# Whole genome re-sequencing reveals recent signatures of selection in three strains of farmed Nile tilapia (*Oreochromis niloticus*)

**DOI:** 10.1038/s41598-020-68064-5

**Published:** 2020-07-13

**Authors:** María I. Cádiz, María E. López, Diego Díaz-Domínguez, Giovanna Cáceres, Grazyella M. Yoshida, Daniel Gomez-Uchida, José M. Yáñez

**Affiliations:** 1 Facultad de Ciencias Veterinarias y Pecuarias, Universidad de Chile, Avenida Santa Rosa 11735, 8820808 La Pintana, Santiago Chile; 2 Programa de Doctorado en Ciencias Silvoagropecuarias y Veterinarias, Campus Sur, Universidad de Chile, Santa Rosa 11315, 8820808 La Pintana, Santiago Chile; 3 Department of Animal Breeding and Genetics, Swedish University of Agriculturall Sciences, Uppsala, Sweden; 4 Departamento de Ciencias de la Computación, Universidad de Chile, Santiago, Chile; 5 Facultad de Ciencias Naturales y Oceanográficas, Universidad de Concepción, Concepción, Chile; 6 Núcleo Milenio INVASAL, Concepción, Chile

**Keywords:** Animal breeding, Genomics

## Abstract

Nile tilapia belongs to the second most cultivated group of fish in the world, mainly because of its favorable characteristics for production. Genetic improvement programs and domestication process of Nile tilapia may have modified the genome through selective pressure, leaving signals that can be detected at the molecular level. In this work, signatures of selection were identified using genome-wide SNP data, by two haplotype-based (*iHS* and *Rsb*) and one *F*_*ST*_ based method. Whole-genome re-sequencing of 326 individuals from three strains (A, B and C) of farmed tilapia maintained in Brazil and Costa Rica was carried out using Illumina HiSeq 2500 technology. After applying conventional SNP-calling and quality-control filters, ~ 1.3 M high-quality SNPs were inferred and used as input for the *iHS*, *Rsb* and *F*_*ST*_ based methods. We detected several candidate genes putatively subjected to selection in each strain. A considerable number of these genes are associated with growth (e.g. *NCAPG, KLF3, TBC1D1, TTN*), early development (e.g. *FGFR3, PFKFB3*), and immunity traits (e.g. *NLRC3*, *PIGR*, *MAP1S*). These candidate genes represent putative genomic landmarks that could be associated to traits of biological and commercial interest in farmed Nile tilapia.

## Introduction

Nile tilapia (*Oreochromis niloticus*) is a teleost fish of the Cichlidae family native to Africa and the Middle East. The geographic range of the species extends from 8°N to 32°N^1^. The first record of domestication is dated around 3,500 years ago as evidenced in paintings at the Theban tombs in Egypt^2^. Nowadays, this species is the second most cultivated group of fish in the world^3^. Favorable characteristics for production include rapid growth, adaptability to different culture conditions, tolerance to high densities, disease resistance, easy reproduction, and tolerance to low concentrations of oxygen^4^.

Genetic improvement programs (GIPs) for Nile tilapia began in 1988 as an approach to counteract the production decrease generated by introgressions with Mozambique tilapia (*Oreochromis mossambicus*)^5,6^. Since then, nearly twenty GIPs have been established for Nile tilapia around the world^7,8^. GIPs aim to improve traits of commercial interest, such as growth rate, disease resistance, cold and salinity tolerance^7^. The GIFT (Genetic Improvement of Farmed Tilapia)^9^ Nile tilapia strain was developed by the ICLARM (International Centre for Living Aquatic Resources Management, now the WorldFish Center), in collaboration with the Norwegian Institute of Aquaculture Research (AKVAFORSK, now NOFIMA Marin)^1^. The implementation of GIPs for the GIFT population has been successful, because growth rate in Nile tilapia has doubled in five generations, showing that this species had a positive response to selection^1^.

Domestication is the process of constant evolutionary and genetic changes in response to captivity^10^. Nile tilapia can be considered to have reached the level of true domestication (level 5), according to the five categories of the domestication process^11,12^. This process may have shaped the genetic diversity of Nile tilapia, leaving signatures in their genomes that can be traced. These signatures can: (1) exhibit increased allele frequencies in favorable adaptive substitutions^13,14^, (2) show strong linkage disequilibrium (LD) in areas surrounding the signature, which decays downstream and upstream of this region^15^, and (3) undergo loss of genetic diversity (selective sweep) in the genome of domestic species compared to the genomes of wild relatives^16^.

Selection signatures can be detected by scanning the genome of sampled individuals in a given population to search for deviations in allele frequency spectrum (Tajima’s D and Fay and Wu’s H scores), higher or lower population differentiation than under neutral expectations (*F*_*ST*_ value), or based on both, measures of LD (*EHH*, *iHS*, *Rsb* methods)^17^ and demographic changes, such as the coalescent three approach^18^. The most suitable method to detect selection signatures depends on the number of populations under study, temporal context scale, and type of selection signatures^17,19^. Thus, more than one approach is often required to capture any signal in the genome^20^. For example, methods derived from *EHH* are used to detect recent positive selection within-population (*iHS*) and between-populations (*Rsb*)^21^, whereas methods based on *F*_*ST*_ are expected to identify older selection events^22^ between-populations^23^.

Several studies of selection signatures have been carried out in aquaculture species^24–28^. Among tilapia and related species, there are only two studies on selection signatures; one based in a comparison of different African cichlid fish lineages^29^ and another one describing whole-genome selection signatures in a total of 47 samples belonging to five tilapia strains^30^. The purpose of the present study was to identify recent signatures of selection in three domestic strains of Nile tilapia from Brazil (strain A) and Costa Rica (strains B and C). We used whole-genome re-sequencing data and applied three statistical approaches to identify genomic regions putatively under selection: (1) *iHS*, (2) *Rsb* and (3) *F*_*ST*_ methods. Finally, the genes under selection were associated with biological functions by performing an enrichment analysis.

## Results

### Quality control

Approximately 76.6 million raw reads (SD = 65.0 million raw reads) per fish were generated for 326 individuals through whole genome re-sequencing. From these, 99.6% were successfully mapped to the reference genome of Nile tilapia. The mean sequencing coverage per individual was 8.7X (SD = 9.9X). Subsequent variant calling yielded a total of 38.45 million variants discovered. From this set, only 1.3 million variants were shared among all three populations and 280 individuals were kept after quality control, which were used for the following analysis (23 individuals with call rate below 80% and 23 with *IBD* > 0.5 were removed).

### Basic statistics and population structure analysis

Observed and expected heterozygosity (*H*_*o*_/*H*_*e*_) obtained were 0.236/0.306, 0.253/0.298 and 0.233/0.299 for A, B and C strains, respectively (Table 1). All these genetic diversity measures were significantly different among populations (*p* < 0.05, Kruskal–Wallis test). The average genome-wide nucleotide diversity (π) within each strain were 8.46 × 10^−4^, 9.39 × 10^−4^, and 8.46 × 10^−4^ for A, B and C populations, respectively (Table 1, Supplementary Fig. S1). We can see that strain B shows a slightly higher level of π than A and C, while these last two present a similar value. The Weir and Cockerham mean (*F*_*ST*_) values among the three strains were low and very similar: A versus B = 0.045 (CI = 0.0445–0.0446), A versus C = 0.045 (CI = 0.0446–0.0449), and B versus C = 0.042 (CI = 0.0413–0.0416).

**Table 1:** Genetic diversity of the three strains of Nile tilapia analyzed in this study. *H*_o_: Observed heterozygosity; *H*_e_: Expected heterozygosity; SD: Standard deviation; CI: Confidence interval.

Strain	Origin	n	H_o_	SD	(CI 95%)	H_e_	SD	(CI 95%)	π
A	Brazil	56	0.236	0.119	0.236–0.237	0.306	0.126	0.306–0.306	8.46 × 10^−4^
B	Costa Rica	100	0.253	0.121	0.253–0.254	0.298	0.124	0.298–0.298	9.39 × 10^−4^
C	Costa Rica	124	0.233	0.112	0.232–0.233	0.299	0.124	0.299–0.299	8.46 × 10^−4^

**Figure 1:**
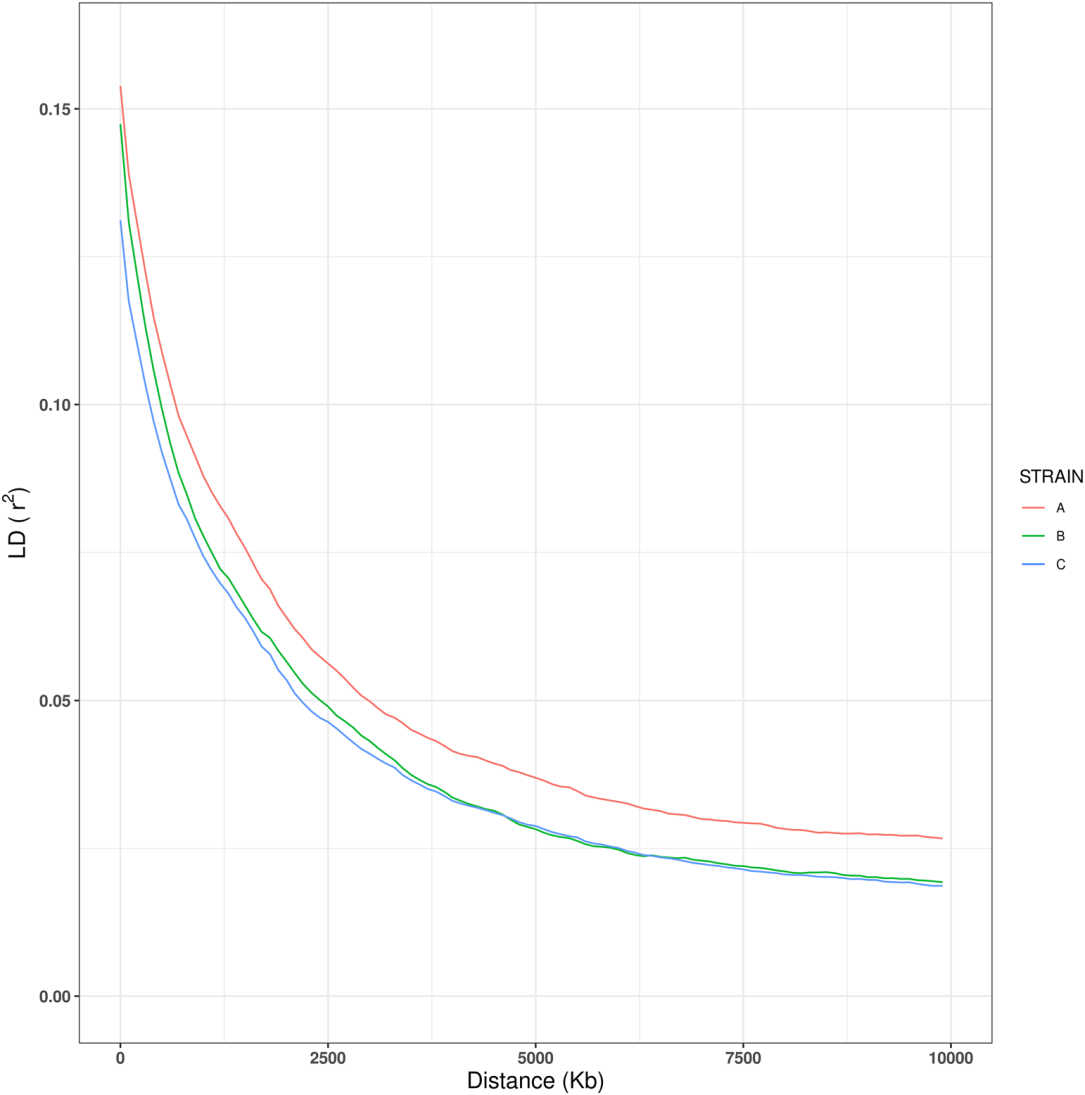
Decay of linkage disequilibrium (*r*^*2*^) over distance across the genome in the three strains of Nile tilapia. Strain A: red, strain B: green, and strain C: blue.

Overall *r*^*2*^ values by strain were plotted against increasing distances (Fig. 1). A rapid decay of LD with increasing distance between markers was observed in all strains of Nile tilapia; however strain A presented a slower LD decay in comparison with strains B and C, which presented similar patterns of LD decay. The values of average LD (*r*^*2*^) in each strain correspond to 0.0486 (strain A), 0.0406 (strain B) and 0.0390 (strain C). Average *r*^*2*^ between adjacent SNPs on each chromosome had some variation in the extent of LD in each strain (Supplementary Figure S2, Supplementary Table S1).

**Figure 2:**
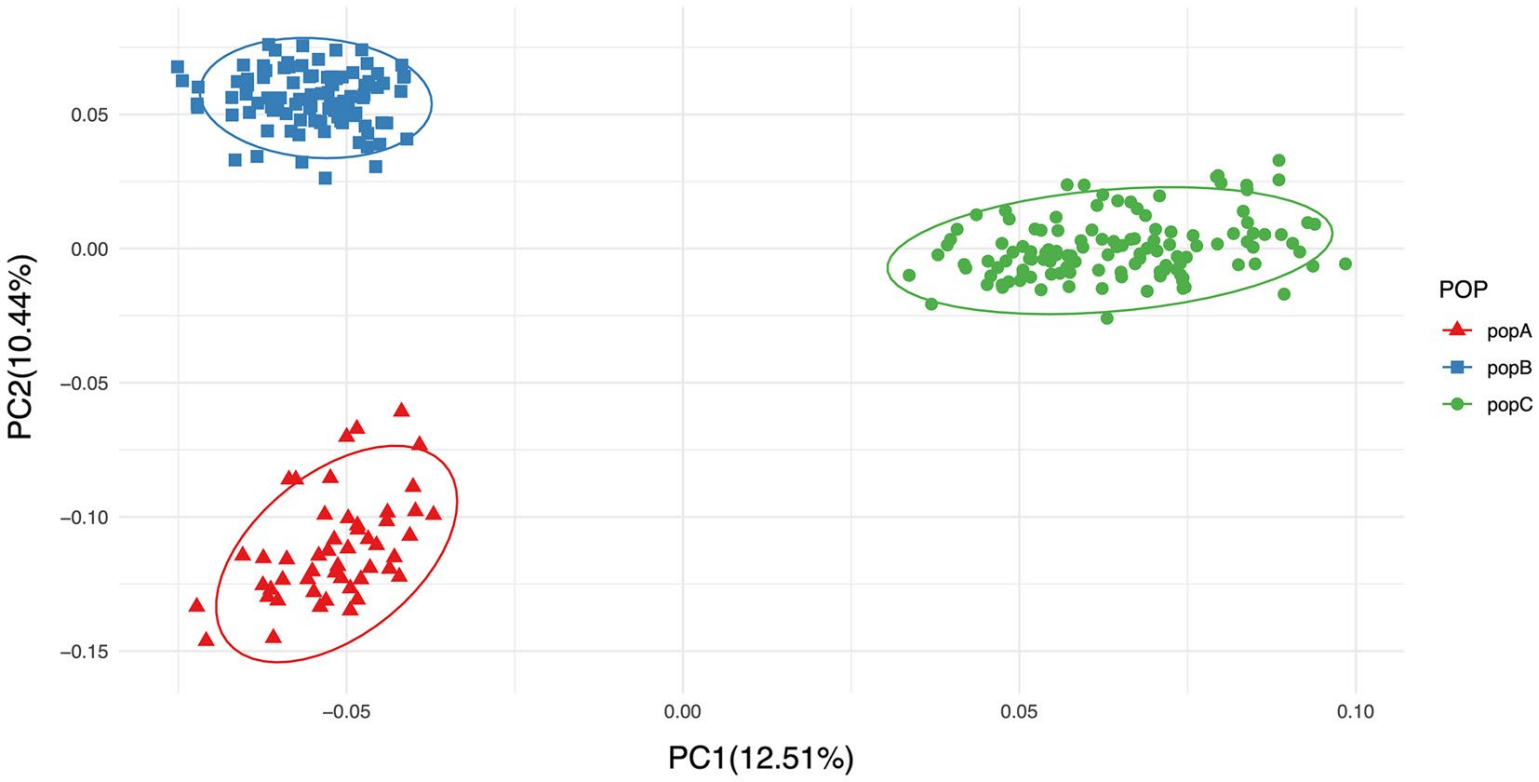
Principal component analysis (PCA) of genetic differentiation among 280 individuals based on ~ 1.3 million SNPs. Each dot represents one individual.

**Figure 3:**
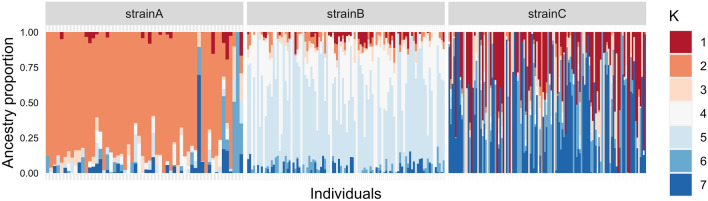
Admixture analysis (K = 7) of the three Nile tilapia populations included in the present study: strain A, strain B and strain C. Each color represents a different theoretical ancestral population and each individual is represented by a vertical bar.

The principal component analysis (PCA) (Fig. 2) shows three distinct clusters corresponding to strain A, B and C of Nile tilapia. The first two eigenvectors together explain 22.45% of the total variance. Based on the first principal component (PC1), the first two clusters correspond to strains A and B, and the third one corresponds to strain C. In addition, admixture analysis revealed that the expected number of ancestral population (K value) is seven (Fig. 3), in agreement with the expected level of admixture for the strains studied here.

### Signatures of selection

**Figure 4:**
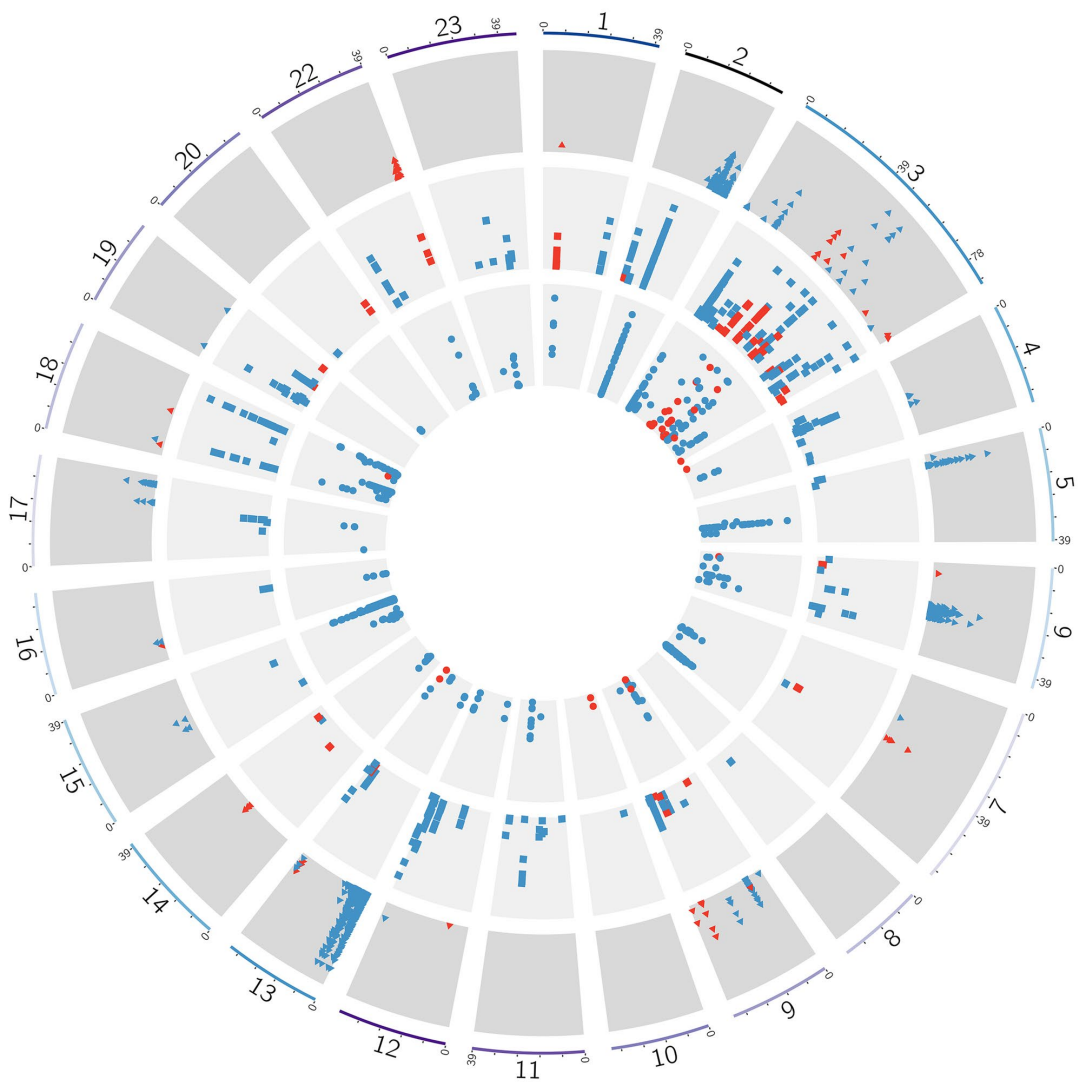
Circos plot of Nile tilapia genome showing signatures of selection in strains A, B, and C. Strain A: outer ring (triangles); strain B: middle ring (squares); and strain C: inner ring (circles). Every dot in the plot represent a particular candidate SNP underlying selection. The y-axis contains the *iHS* values (red) and the *Rsb* scores (blue) over the threshold 7.4 (−log_10_(*p* value), while the x-axis has the chromosome positions.

The *iHS* analysis revealed signatures of selection in the three strains studied (Fig. 4). We found 59, 73 and 30 outlier SNPs indicative of selection for strains A, B and C, respectively (Supplementary Table S2). Annotation of these regions revealed 133, 184 and 73 genes localized in the 250 kb windows harboring each marker in strains A, B and C, respectively. Details of the candidate regions and genes identified can be found in Supplementary Table S3. In LG 3, we found nine candidate genes shared between three strains (A–B–C), 20 candidate genes were overlapped between strains B–C and ten candidates genes were shared between strains A–B and C–A.

The *Rsb* method across the three possible pairs of populations detected several SNPs surpassing the significance threshold (Fig. 4). In the comparison between strains A and B we identified 1,394 SNPs surpassing the threshold, with 980 and 414 SNPs showing evidence of selection in strain A and B, respectively. In the comparison between strains B and C, we identified 839 SNPs surpassing the threshold, with 323 and 516 candidates SNPs in strain B and C, respectively. Finally, in the comparison between strains C and A we detected 1,167 SNPs surpassing the threshold, with 295 and 872 potential SNPs under selection in strains C and A, respectively. In summary, 1,287, 622 and 649 unique candidate SNPs showed evidence of selection in strains A, B and C, respectively (Supplementary Table S2). Associated with these candidates regions we found 559, 765 and 591 genes distributed within a 250 kb windows harboring each marker in strains A, B and C, respectively. Details of the candidate regions and genes can be found in Supplementary Table S4.

Overall, when analyzing *iHS* and *Rsb* results, which approximately follow a normal distribution (See supplementary Fig. S3 and S4), we found overlap in 10, 62 and 21 genes across strains A, B and C, respectively (Fig. 4). Associated with this regions we found several genes potentially linked with the domestication process in these strains of Nile tilapia. For instance, we found genes relevant for growth-traits (*ANKRD46*, *TTN*, *TCD7L1*, *VCAM1* and *KIF1C);* early development (*SYNA*, *GNG7*, *ELAVL1*, *TSPAN3*, *G2E3*, and *PLTP);* immunity traits (*Ladderlectin, FCRL5, HAVCR2, NLRC3, PIGR, MAP1S and TRIM16L);* reproduction (*GPA33, VIPR2* and *CARTPT)* and adaptation to the environment (*DIP2C*). The full list of genes is shown in Supplementary Table S5. Only one common gene in LG3 (Ladderlectin) was detected by both approaches and across all strains. When comparing pairs of populations, we found five (BC), three (AB) and seven (AC) shared genes, by using both approaches.

**Figure 5:**
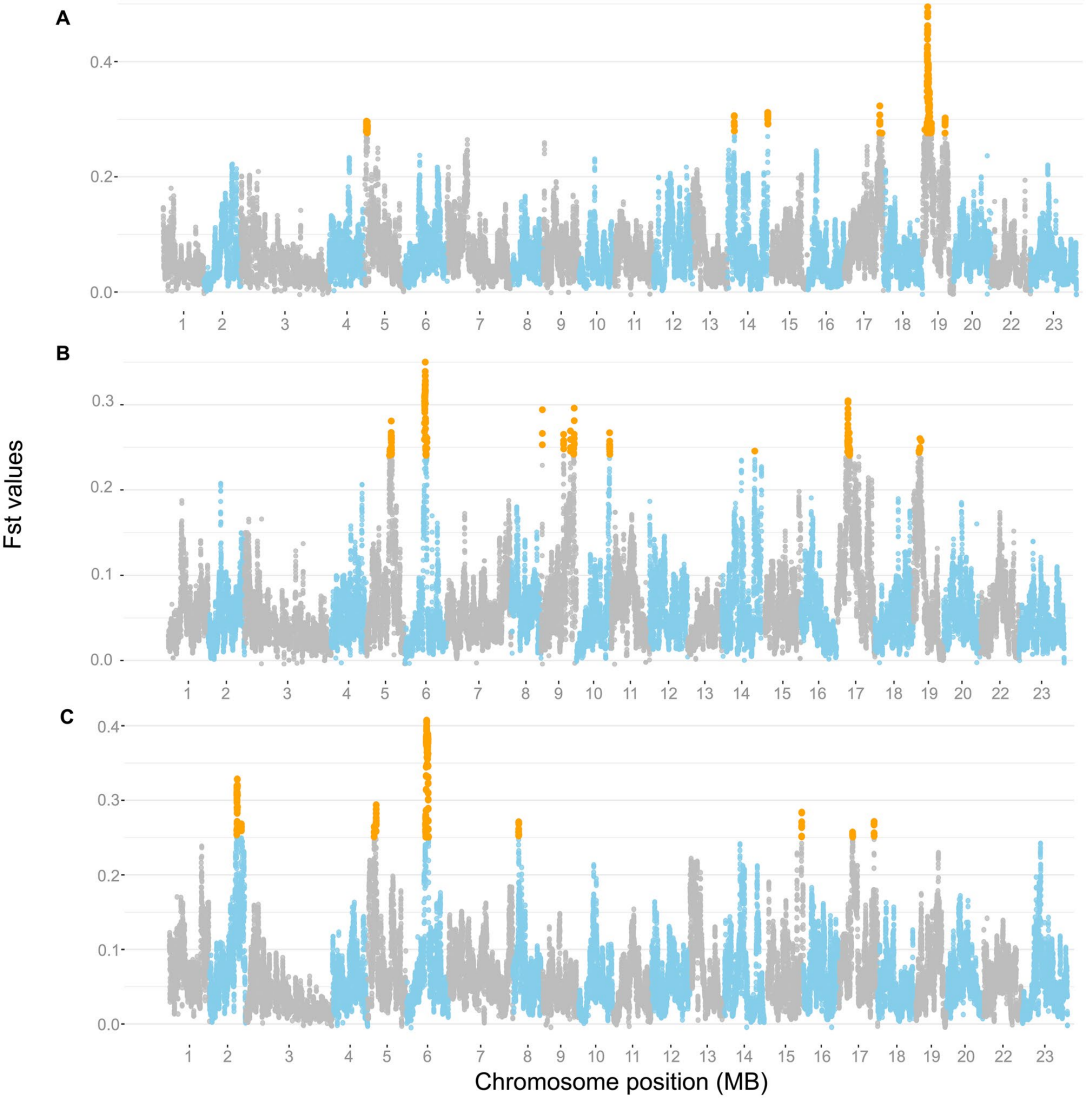
Genome-wide distribution of weighted *F*_*ST*_ values across the three pairwise comparisons between Nile tilapia strains: (**A**) strains AB, (**B**) strains BC and (**C**) strains CA. Orange dots represent outlier values of *F*_*ST*_ (top 0.5%).

By applying the *F*_*ST*_ approach to compare strains AB, BC, and CA, we detected 174 genomic windows over the 0.5% top values in all comparison (Fig. 5). We detected 201 (LG 5, 14, 17 and 19), 231 (LG 5, 6, 9, 10, 14, 17 and 19) and 221 (LG 2, 5, 6, 8, 15 and 17) candidate genes associated with comparisons between strains AB, BC, CA, respectively. We found genes potentially associated with the domestication process. For instance, we found genes relevant for growth-traits *(IL15RA, OPTN, ADRA1D, CISD2, NCAPG, DOC2B, KLF3* and *TBC1D);* early development (*PFKFB3, CAMK1D, AHSA1, FGFR3, LOXL3, SERTAD1, BDH2, METTL14* and *PGM2);* immunity traits (*CDK17*, *MAVS*, *SERPING1*, *LONRF1*, and *PAX5*) and reproduction *(ASMT* and *NANOS1*). The full list of genes is shown in Supplementary Table S5. See details of all regions and genes detected by *F*_*ST*_ method in Supplementary Table S6.

### Comparison of selection signatures between methods

**Figure 6:**
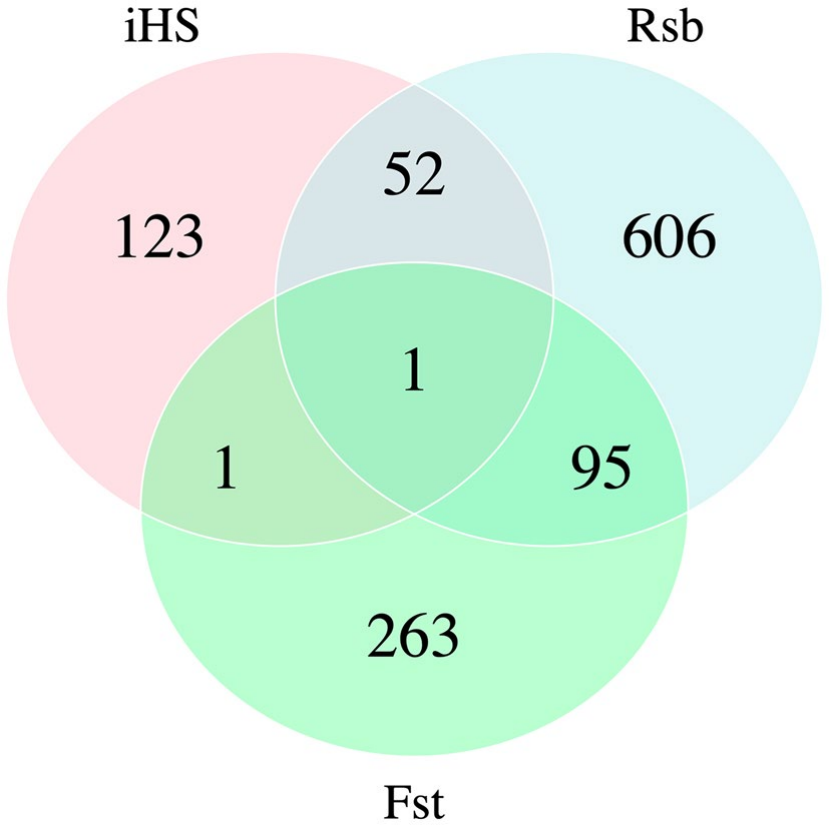
Venn diagram showing shared genes identified between the *iHS*, *Rsb* and *F*_*ST*_ approaches in the three strains of Nile tilapia.

We found 96 candidate genes detected by both *Rsb* and *F*_*ST*_ approaches. 53 candidate genes overlapped between *iHS* and *Rsb*. Between *iHS* and *F*_*ST*_ we found only two common genes. Finally, we identified only one gene detected by all methods (Fig. 6). Based on the SnpEff results, we found that most of the SNPs detected by the three methods were intronic (44.83%) and intergenic (19.05%) variants, and only 2.8% of SNPs were located within exon regions.

### Functional enrichment analysis

The results of enrichment analysis of the total signals of selection detected by both *iHS* and *Rsb* methods are shown in Supplementary Table S7. Overall for strain A, we found a total of 647 genes, which were classified in 53 functional terms, including Biological Processes (BP, 40 terms), Cellular Components (CC, 6 terms), Molecular Function (MF, 2 terms) and the KEGG pathways (5 terms). For strain B, we found 703 genes associated with 28 functional terms, which correspond to BP (15 terms), CC (1 terms), MF (8 terms) and the KEGG pathways (4 terms). Finally, for strain C, we detected 516 genes linked to 61 functional terms, which include BP (46 terms), CC (2 terms), MF (10 terms) and the KEGG pathways (3 terms). Biological terms that were related to domestication processes were further labeled in these categories; (G) growth, (E) early development, (B) behavior and (A) adaptation to environment. Relevant GO categories are presented in Tables 2, 3 and 4 (For detailed results see also Supplementary Figure S5, S6 and S7).

**Table 2:** Enriched GO and KEGG pathway terms for genes related to domestication in regions under selection of strain A of Nile tilapia. Traits are defined as growth (G), early development (E), behavior (B) and adaptation to environment (A).

Code	Term	Genes	*p* value	Trait
*Biological process* (*BP*) 57.0% (369 *genes*)				
GO:0021546	Rhombomere development	5	0.001	E
GO:0048468	Cell development	46	0.004	E
GO:0050890	Cognition	4	0.006	B
GO:0007399	Nervous system development	52	0.007	G
GO:0021593	Rhombomere morphogenesis	4	0.010	E
GO:0048469	Cell maturation	5	0.015	E
GO:0008038	Neuron recognition	4	0.022	B
GO:0030154	Cell differentiation	61	0.023	E
GO:0007417	Central nervous system development	25	0.024	E
GO:0021654	Rhombomere boundary formation	3	0.028	E
GO:0021594	Rhombomere formation	3	0.039	E
GO:0051216	Cartilage development	8	0.043	G
GO:0048666	Neuron development	24	0.043	B
GO:0048859	Formation of anatomical boundary	3	0.045	E
GO:0007612	Learning	3	0.045	B
GO:0061448	Connective tissue development	8	0.051	E
GO:0060113	Inner ear receptor cell differentiation	4	0.052	E
GO:0048731	System development	90	0.053	E
GO:0048514	Blood vessel morphogenesis	15	0.060	E
GO:0002040	Sprouting angiogenesis	6	0.067	E
GO:0048285	Organelle fission	12	0.068	G
GO:0007422	Peripheral nervous system development	5	0.068	E
GO:0030902	Hindbrain development	7	0.081	E
GO:0048599	Oocyte development	3	0.082	R
GO:0022402	Cell cycle process	16	0.083	G
GO:0048477	Oogenesis	4	0.085	R
GO:0009887	Organ morphogenesis	24	0.089	E
GO:0009994	Oocyte differentiation	3	0.090	R
*Celular component* (*CC*) 38.0% (246 *genes*)				
GO:0030018	Z disc	4	0.051	G
GO:0030016	Myofibril	6	0.059	G
GO:0043292	Contractile fiber	6	0.064	G
GO:0031674	I band	4	0.068	G
*Molecular function* (*MF*) 58.9% (381 *genes*)				
GO:0008092	Cytoskeletal protein binding	20	0.071	G
*KEGG* 23.6% (153 *genes*)				
dre04020	Calcium signaling pathway	13	0.061	G
dre04512	ECM-receptor interaction	6	0.077	E

**Table 3:** Enriched GO and KEGG pathway terms for genes related to domestication in regions under selection of strain B of Nile tilapia. Traits are defined as growth (G), early development (E), behavior (B) and adaptation to environment (A).

Code	Term	Genes	*p* value	Trait
*Biological process* (*BP*) 44.8% (315 *genes*)				
GO:0001666	Response to hypoxia	5	0.042	A
GO:0008361	Regulation of cell size	6	0.068	G
GO:0006259	DNA metabolic process	16	0.068	G
GO:0003158	Endothelium development	4	0.084	E
GO:0072080	Nephron tubule development	3	0.086	E
GO:0061326	Renal tubule development	3	0.097	E
*KEGG* 19.1% (134 *genes*)				
dre04514	Cell adhesion molecules (CAMs)	8	0.05	E
dre04810	Regulation of actin cytoskeleton	12	0.069	G
dre04510	Focal adhesion	11	0.084	E

**Table 4:** Enriched GO and KEGG pathway terms for genes related to domestications in regions under selection of strain C of Nile tilapia. Traits are defined as growth (G), early development (E), behavior (B) and adaptation to environment (A).

Code	Term	Genes	*p* value	Trait
*Biological process* (*BP*) 42.8% (221 *genes*)				
GO:0007422	Peripheral nervous system development	6	0.003	E
GO:0002934	Desmosome organization	3	0.003	E
GO:0001666	Response to hypoxia	5	0.013	A
GO:0035270	Endocrine system development	6	0.014	E
GO:0007411	Axon guidance	9	0.015	B
GO:0097485	Neuron projection guidance	9	0.016	E
GO:0006606	Protein import into nucleus	5	0.016	G
GO:0005996	Monosaccharide metabolic process	5	0.022	G
GO:0010001	Glial cell differentiation	5	0.026	E
GO:0042478	Regulation of eye photoreceptor cell development	2	0.033	E
GO:0061564	Axon development	12	0.035	E
GO:0060027	Convergent extension involved in gastrulation	5	0.041	E
GO:0043010	Camera-type eye development	10	0.046	E
GO:0048667	Cell morphogenesis involved in neuron differentiation	11	0.058	E
GO:0060059	Embryonic retina morphogenesis in camera-type eye	3	0.072	E
GO:0000904	Cell morphogenesis involved in differentiation	12	0.075	B
GO:0031175	Neuron projection development	13	0.075	B
GO:0048812	Neuron projection morphogenesis	11	0.080	B
GO:0002009	Morphogenesis of an epithelium	12	0.086	E
GO:0048592	Eye morphogenesis	7	0.089	E
*KEGG* 18.0% (93 *genes*)				
dre04115	P53 signaling pathway	5	0.034	A
dre04110	Cell cycle	6	0.085	G

## Discussion

Previous studies aiming at identifying selection signatures have been performed in different aquaculture species, including Atlantic salmon^24,25,28^ and brown trout^26^. In Nile tilapia, there are only two studies of this kind and both have taken an inter-species approach to detect signals of adaptation and selection in this species. The first one was carried out in the African cichlid lineages, including *O. niloticus* and four other representative species of the cichlid family. The authors found molecular mechanisms shaped the East African cichlid genome, which may have been influential in facilitating subsequent evolutionary diversification^29^. The second study was focused on *O. niloticus*, *O. mossambicus* and their hybrids^30^ and found selection signatures in different genes, including molecules from the Wnt signaling, GnRH receptor and integrin signaling pathways. In this study, we evaluate the presence of selection signatures in three strains of Nile tilapia cultured in Brazil (strain A) and Costa Rica (strains B and C) using data from a whole-genome re-sequencing experiment and three statistical methods (*iHS*, *Rsb* and *F*_*ST*_).

### Basic statistics and genetic structure

Genetic diversity (*H*_*e*_, *H*_*o*_ and π) was low and similar between all strains of Nile tilapia. These results are in agreement with those reported by previous works (*H*_*e*_ ranging from 0.2 to 0.4^30–34^). Low genetic diversity is expected in domesticated populations, compared to their wild conspecifics as these populations can lose genetic diversity due to selective breeding and the absence of gene flow with other populations^35^. The low genetic diversity present in three different populations of farmed Nile tilapia, can be explained by a relatively low effective population size (*N*_*e*_) and the consequent genetic drift and inbreeding^36^. Our results account for a similar rapid LD decay between these strains. Previous studies in this species revealed similar low values of LD; which may have been influenced by recombination rates, effective populations sizes, genetic background and breeding history, including admixture events^34,37^. The results described above are in accordance with those values of *N*_*e*_ reported by Yoshida et al. (2019) (159, 128, 78 for strains A, B and C, respectively). These values are somewhat higher than expected, as domesticated animals typically have values of *N*_*e*_ < 100^38^. Even though these values of *N*_*e*_ are relatively small, they are enough to maintain inbreeding at acceptable rates of accumulation per generation and the necessary levels of diversity in the long-term for these breeding populations^34,39^.

Regarding the genetic structure, the PCA identified three clusters consistent with the three strains of Nile tilapia analyzed here (Fig. 1). As expected, the results of the ancestry analysis (Fig. 2) showed several original lineages (best K = 7), accounting for the multiple origins of the strains, all of them based on GIFT population. The GIFT strain is a synthetic population composed of eight wild and farmed populations of Nile tilapia^1,31^.

### Signatures of selection

As anticipated, our results suggest that domestication and selective breeding have caused changes in the genome of all the strains studied here. Based on our analysis, it was possible to detect several genes involved in biological processes, such as growth, early development, reproduction, immunity traits, behavior and adaptation to environment, which could be under the effect of domestication and directional selection in these strains of Nile tilapia.

None of the candidate regions were found to overlap across all the three analyses. The discrepancies found between methods may be due to the fact that each approach captures a particular signal in the genome^17^ and they may correspond to different types of selection events^40^. The *iHS* test has higher statistical power when selected alleles are at intermediate frequencies^41^. The *Rsb* approach can identify selected alleles which are fixed or close to fixation^42^. Whereas, the *F*_*ST*_ method is sensitive in identifying fixed alleles^43^. When comparing the number of SNPs detected for each method, *Rsb* and *F*_*ST*_ detected a higher number than *iHS* in the three strains (Supplementary Table S2). We suggest that a higher number of selection signatures detected by these methods might be associated with the first stages of domestication and the effect of artificial selection, which may have fixed some favorable mutations in a given population^44^. Hence, a lower number of regions detected by the *iHS* method could be reflecting more recent events of selection in these populations.

Our results are in agreement with the expected effect of domestication and adaptation in a culture system which involves genotypic and phenotypic effects^45^. Aquaculture systems are characterized by less complexity than natural conditions. Thus, they tend to decrease adaptive pressures for many traits (competition for food, shelter, mates and avoidance of predators) and induce selective pressures for other traits^36^. The selection in captive environment tends to accelerate body development through an increased growth rate in fish, and also generate changes in patterns of sexual maturity^45^.

The growth rate is of sizeable economic importance for farmers and easy to record in breeding candidates^46^. The genetic improvement of the synthetic base strain (GIFT) and all of the derived strains studied here, has been focused on growth related traits^1^. In fact, all these Latin American strains (A, B, and C) have been improved for growth-related traits for about ten generations. We found several candidate genes (Supplementary Table S5) and enrichment terms linked to growth-traits (Tables 2, 3, 4). We found genes such as *TTN* that is essential to muscle architecture and signaling in developing and mature striated muscle. Mutations in this gene have been correlated with skeletal muscular dystrophy-like in zebrafish^47^. Furthermore, we identified three genes (*NCAPG, KLF3* and *TBC1D1*) associated with growth traits in livestock animals. The *NCAPG* gene has been linked to the condensation and stabilization of chromosomes during meiosis and mitosis^48^ and to growth traits in cattle^49^, equine^50^, chicken^51^ and sheep^52^. The *KLF3* gene is an essential member of the KLF family and is involved in the regulation of growth, development of muscle and adipose tissue in cattle^53^ and goats^54^. The *TBC1D1* gene corresponds to a critical signaling factor of skeletal muscle substrate utilization^55^ and was correlated with improved muscle mass (chicken^56^, porcine^57^ and rabbits^58^). Additionally, these findings account for the possible polygenic nature of the growth trait^59^, i.e., the growth of fish is controlled by large numbers of small-effect genes^60^. Polygenic dependence is suggested as the growth trait was found linked to several genes and enrichment terms. In addition, we detected several candidate genes (Supplementary Table S5) and enrichment terms (Tables 2, 3, 4) linked to the early development process, which could certainly affect growth. This trait is relevant because myogenesis begins at an earlier development stage in fish embryos than in amniotes such as birds and mammals^61^. Myogenesis corresponds to the formation of muscle fibers involved in the differentiation, fusion, and absorption of myogenic precursor cells to form syncytial fibers^61^.

Additionally, resistance to infectious diseases is an economically relevant trait and is considered a long-term aim because of the consequences of this trait on fish health and growth^62^. In Nile tilapia there are programs that select for disease resistance^7^, but none of the strains used in this study has been artificially selected for disease resistance. However, we suggest that the culture system has commanded natural selection on regions implicated in immunity traits. In fact, we found evidence of selection in several molecules associated with the immunity traits (Supplementary Table S5). Specifically, we found three genes previously associated with defense against bacterial pathogens such as *Streptococcus agalactiae* (*NLRC3*^63^ and *PIGR*^64^) and *Streptococcus iniae* (*MAP1S*^65^) in Nile tilapia. Streptococcosis is an important disease, and the outbreaks affect the advancement of tilapia aquaculture globally. Also, we found the *Ladderlectin* gene, which has been associated with an innate immune response mechanism, that corresponds to plasma pattern recognition for bacterial, fungal and viruses in rainbow trout^66^.

Through captivity, fish populations present changes in behavior-related traits as well, including aggressiveness, foraging, anti-predator and reproductive behavior, which frequently decrease in complexity^36^. We found several genes and enriched terms associated with behavior. For example, we found GO terms related to cognition (GO:0050890) and learning (GO:0007612), traits which have been reported to be impacted by the effect of domestication of fish^67^.

The production of tilapia commonly requires the use of monosex (all-male) populations because they grow about twice as fast as females. We would expect then to detect genes underlying traits related to sexual dimorphism as showing signs of selection. Associated with reproduction processes we found that the *GPA33* gene was previously associated with the early embrionic differentiation of males and females in Nile tilapia^68^. We also found the gene *VIPR2*, which plays a role in the pathway of the follicle growth and maturation in zebrafish^69^. Another relevant gene found by our analysis was *ASMT*, implicated in encoding the second enzyme required for melatonin synthesis^70^, which is in turn involved in growth, gonadal maturity, lipid and protein production in Nile tilapia^71^.

Other interesting genes and GO terms were related with the adaptation to environmental stimuli. Firstly, we found the *DIP2C* gene previously associated with a potential major QTL of salinity tolerance in Nile tilapia^72^. The selection of saline tolerance and superior growth rate is particularly crucial for tilapia production in brackish water areas^72^ and some breeding programs have focused on improving this trait in tilapia^7^. Secondly, in strains B and C, we detected one term associated with response to hypoxia (GO:0001666) (Supplementary Table S7). These characteristics might represent advantageous and functional adaptations for farming systems^45^.

## Conclusion

In this study, we detected several genomic regions putatively underlying selection in three farmed populations of Nile tilapia. These regions harbor interesting candidate genes, which may be associated with the adaptive processes to captivity and traits of economic importance, which have been subjected to artificial directional selection. Also, the result of the enrichment analysis of all candidate genes identified was often linked to production traits, most commonly growth and early development, accounting for the potential effect of genetic improvement in these three strains. Our results may be relevant for a better understanding of genes underlying traits of interest in aquaculture and the effect of domestication in the genome of Nile tilapia.

## Methods

### Fish samples

A total of 326 individuals of farmed Nile tilapia from three commercial strains cultivated in two different countries of Latin America were included in this study (Table 1). Strain A was originally imported from Malaysia to Brazil in 2005, and samples for this study were obtained from the breeding population of AquaAmerica, Brazil. This strain is derived from the GIFT strain, a mixture of four Asian domestic strains from Israel, Singapore, Taiwan and Thailand with four wild populations from Egypt, Senegal, Kenya and Ghana^1^. Strains B and C were introduced from the Philippines (station Carmen Aquafarm) to Costa Rica in 2005, and samples were obtained from the Aquacorporacion Internacional (Costa Rica) breeding population. Strain B is a mixture of an eight-generation GIFT strain, two wild populations from Egypt and Kenya and fish from Strain C, which in turn originated from a mixture of Asian domestic strains from Israel, Singapore, Taiwan and Thailand. Sampling protocols were performed in accordance with *Comité de Bioética Animal, Facultad de Ciencias Veterinarias y Pecuarias, Universidad de Chile, Chile* (certificate Nº19179-VET-UCH).

### Sequence data and quality control

DNA from all individuals was purified from fin-clip samples using a Wizard Genomic DNA purification kit (Promega). The DNA libraries were prepared and sequenced using an Illumina HiSeq 2500 machine (Illumina, USA) as described by Cáceres et al.^33^ and Yáñez et al.^73^. Reads were aligned to the Nile tilapia reference genome (O_niloticus_UMD, GCA_000188235.2) with BWA MEM^74^. The discovery of variants was made with the Genome Analysis Toolkit (GATK) software version 3.5.0. (https://www.broadinstitute.org/gatk/)^75^. Detailed information on variant discovery is fully described in Yáñez et al. [73]. The variant coordinates were updated to the latest version of the genome (O_niloticus_UMD_NMBU, GCA_001858045.3^76^), taking probes of 200 pb and locating them in the new version of the genome.

The variants were filtered using the VCFtools software v0.1.15^77^ and SNPs that did not pass the following quality control (QC) criteria were removed: (1) indels, (2) SNPs with more than two alleles, (3) quality of phred score < 30, (4) SNP call rate < 90%, (5) mitochondrial SNP, (6) SNP deviating from Hardy–Weinberg Equilibrium (HWE, *p* value < 1 × 10^−9^), and (7) minor allele frequency (MAF) < 0.05. Step 6 and 7 were applied on each strain separately. The individuals exhibiting variant call rate below 80% were removed. Closely related individuals may bias estimates of allelic and haplotypic frequencies, and thus they might mask signatures of selection^20^. Related individuals have in common homologous chromosome segments that coalesce in a recent common ancestor^78^. To avoid highly related individuals within samples we performed an analysis of identity by descent (*IBD*) with PLINK v1.09^79^, where one individual from pairs of animals with high values of IBD were excluded. We imputed missing genotypes and inferred haplotypes using BEAGLE v.3^80^ applying default parameters.

### Basic statistics and population structure analysis

Genetic diversity among populations was calculated through observed and expected heterozygosities (*H*_*o*_ and *H*_*e*_) using PLINK v1.09. The nucleotide diversity (π) was characterized over the entire genome using 250 kb genomic bins and a 10 kb step window (–window-pi 250000 –window-pi-step 10000) using VCFtools v0.1.15. We measured genetic differentiation among strains using pairwise Weir and Cockerham's *F*_*ST*_ estimator implemented in StAMPP package for R^81^.

To examine genetic structure among populations, we first performed a PCA implemented in PLINK v1.09. Second, to infer the number of ancestral populations between strains we used the maximum likelihood analysis of individual ancestries by ADMIXTURE software^82^. The number of ancestral populations (K) was set from 1 to 10 and the optimal K was selected based on the lowest cross-validation error and a visual inspection of co-ancestry values.

In addition, we characterized the pairwise linkage disequilibrium (LD) as the Pearson’s squared correlation coefficient (*r*^*2*^) for each strain (A, B and C) and within chromosomes using PLINK v1.09. SNP pairs were located into bins of 100 Kb to calculate mean values of *r*^*2*^ for each bin.

### Signatures of selection

We used three methods to detect signatures of selection: two haplotype-based (*iHS* and *Rsb*) and one *F*_*ST*_ based method. The first two methods are based on extended haplotype homozygosity (*EHH*), which correspond to the probability that two randomly chosen chromosomes carrying the core haplotype are identical by descent^83,84^. The first method is the intra-population standardized integrated haplotype score (*iHS*)^41^; the second is the inter-population standardized log-ratio of integrated *EHH* (*iES*) between pairs of populations (*Rsb*)^42^. Both methods were applied using REHH package^83^.

The *iHS* method compares *EHH* values between alleles within one population, i.e. the area under the curve of the derived and ancestral alleles^84^. This procedure requires the identification of the ancestral allele for each SNP, which is automatically inferred by the REHH package (polarize_vcf = FALSE). Standardized *iHS* was defined as Eq. (1):

1 $$ iHS = \frac{{ln\left( {\frac{{iHH_{A} }}{{iHH_{D} }}} \right) - E_{p} \left[ {ln\left( {\frac{{iHH_{A} }}{{iHH_{D} }}} \right)} \right]}}{{SD_{p} \left[ {ln\left( {\frac{{iHH_{A} }}{{iHH_{D} }}} \right)} \right]}} $$

where *iHH*_*A*_ and *iHH*_*D*_ corresponded to integrated *EHH* score for ancestral (A) and derived (D) core alleles respectively. Expectation (Ep) and standard deviation (SD) of ln (*iHH*_*A*_*/iHH*_*D*_). The *iHS* values were calculated separately within-populations (strains A, B and C) and we used all QC-passed SNPs for each strain.

The *Rsb* method compares *EHH* profiles of the same allele between pairs of populations^42^. This method was defined as the natural logarithm of the ratio between *iESpop1* and *iESpop2*, where *iES* represent the integrated *EHHS* (site-specific *EHH*) for both alleles of each SNP within each population. *Rsb* was calculated between pairs of strains (AB, AC, and BC). This method requires no information of ancestral and derived alleles. Positive values of *Rsb* indicate *iESpop1* is greater than *iESpop2*, i.e., pop1 has longer haplotype than pop2, therefore suggest positive selection in the alternative population (pop1)^25^. Conversely, a negative score suggests positive selection in a reference population (pop2)^25^.

The third method used in this study is based on differences in allele frequencies between two populations by estimating the Fixation index, *F*_*ST*_^85^. This approach was carried out using VCFtools software (version 0.1.15) using overlapping sliding windows (250 kb window size and 25kb step size). The window size was determined based on linkage disequilibrium (LD) decay analysis. We evaluated the same three pairs of strains (AB, AC, and BC). The *F*_*ST*_-based approach does not directly indicate in which population selection is operating. Hence, our results were described in terms of the population pairs.

### Candidate genes to selection

Identifying the causal variant at a site of selection is hard, but if SNPs on a selected haplotype are closely linked to a candidate gene, this information could be used as evidence of a potential sign of selection near that gene^14^. For methods based in *EHH*, candidate regions for selection were defined as those genomic positions containing SNPs with values of *iHS* and *Rsb* above the threshold. The threshold used to set the significance of *iHS* and *Rsb* methods corresponds to 7.4 (− log10(*p* value), accounting for Bonferroni correction). For the *F*_*ST*_ method, the top 0.5% of the windows distribution was chosen as the threshold used to determine SNP candidates for being under selection. In both cases, based on the LD decay previously estimated in these populations of Nile tilapia, we used a range of 250 kb around each SNP to explore for candidate genes under selection. The genes intersecting the candidate regions detected by *iHS*, *Rsb* and *F*_*ST*_ method, were considered a candidate to selection and detected using BEDTools^86^. Finally, the prediction of the functional effects of each SNP candidate to be under selection detected by the three methods (*iHS*, *Rsb* and *F*_*ST*_) in the genome of Nile tilapia was predicted using SnpEff^87^.

Using all candidate genes under selection, detected by both methods (*iHS* and *Rsb*), we performed a BLAST against zebrafish (*Danio rerio*) proteins, using the genome annotations from NCBI of both species. An enrichment analysis was conducted using the online tool David Bioinformatics platform^88^ to detect Gene Ontology (GO) and KEGG (Kyoto Encyclopedia of Genes and Genomes) pathway terms.

### Ethics approval

Nile tilapia sampling procedures were approved by the Comité de Bioética Animal from the Facultad de Ciencias Veterinarias y Pecuarias, Universidad de Chile (certificate No. 19179-VET-UCH).

### Consent for publication

No consent was involved in this publication.

## Supplementary information

Supplementary figure and table legends

Supplementary figures

Supplementary table S1

Supplementary table S2

Supplementary table S3

Supplementary table S4

Supplementary table S5

Supplementary table S6

Supplementary table S7

## Data Availability

The datasets generated during and analyzed during the current study are available at the online digital Sequence Read Archive (SRA), and European Variation Archive (EVA) repositories. The access numbers correspond to PRJNA634901 for the raw sequences (https://www.ncbi.nlm.nih.gov/sra/PRJNA634901) and PRJEB38764 for the polymorphisms (VCF files).
