# Supplementary material for: Whole genome re-sequencing reveals recent signatures of selection in three strains of farmed Nile tilapia (Oreochromis niloticus)
Source: Sci Rep. 2020 Jul 13;10:11514. doi: 10.1038/s41598-020-68064-5 (PMC7359307; doi:10.1038/s41598-020-68064-5)
Supplement: Supplementary file 1 — Supplementary figure and table legends [file 41598_2020_68064_MOESM1_ESM.pdf]

Supplementary information legends

Whole genome re-sequencing reveals recent signatures of selection in three strains of farmed Nile tilapia (*Oreochromis niloticus*)

María I. Cádiz[12], María E. López[31], Diego Díaz-Domínguez[4], Giovanna Cáceres[12], Grazyella M. Yoshida[1], Daniel Gomez-Uchida[5.6], José M. Yáñez[1,6]*.

[1] Facultad de Ciencias Veterinarias y Pecuarias, Universidad de Chile, Avenida Santa Rosa 11735, 8820808, La Pintana, Santiago, Chile

[2] Programa de Doctorado en Ciencias Silvoagropecuarias y Veterinarias, Campus Sur, Universidad de Chile, Santa Rosa 11315, La Pintana, Santiago, Chile. CP: 8820808.

[3] Department of Animal Breeding and Genetics, Swedish University of Agricultural Sciences, Uppsala, Sweden.

[4] Departamento de Ciencias de la Computación, Universidad de Chile.

[5] Facultad de Ciencias Naturales y Oceanográficas, Universidad de Concepción, Concepción, Chile.

[6] Núcleo Milenio INVASAL, Concepción, Chile

*jmayanez@uchile.cl +56-2 29785533 (Corresponding Author).

Supplementary information

Supplementary Table S1. Values of LD in each chromosome

Supplementary Table S2. SNPs detected by iHS, Rsb and F_{ST} by chromosome in each strain.

Supplementary Table S3. List of all genes detected by iHS methods.

Supplementary Table S4. List of all genes detected by Rsb methods.

Supplementary Table S5. Candidate genes for selection detected by iHS, Rsb and F_{ST} methods.

Supplementary Table S6. List of all genes detected by F_{ST} methods.

Supplementary Table S7. List of terms and definitions of enrichment analysis.

Supplementary Figure S1. Nucleotide diversity of strain A (red line), B (green line) and C (blue line) of Nile tilapia.

Supplementary Figure S2. Decay of average linkage disequilibrium (r^2) over distance across in each chromosome of three strain of Nile tilapia.

Supplementary Figure S3. Histograms showing the distribution of the iHS values in the three strain of Nile tilapia (A, B and C).

Supplementary Figure S4. Histograms showing the distribution of the Rsb values in the three strain of Nile tilapia (A, B and C).

Supplementary Figure S5. Enrichment analysis for GO and KEGG pathways term for strain A by DAVID. Each bar represents the -log10(p-value) for term.

Supplementary Figure S6. Enrichment analysis for GO and KEGG pathways term for strain B by DAVID. Each bar represents the -log10(p-value) for term.

Supplementary Figure S7. Enrichment analysis for GO and KEGG pathways term for strain C by DAVID. Each bar represents the -log10(p-value) for term.
