# Supplementary material for: Whole genome re-sequencing reveals recent signatures of selection in three strains of farmed Nile tilapia (Oreochromis niloticus)
Source: Sci Rep. 2020 Jul 13;10:11514. doi: 10.1038/s41598-020-68064-5 (PMC7359307; doi:10.1038/s41598-020-68064-5)
Supplement: Supplementary file 2 — Supplementary figures [file 41598_2020_68064_MOESM2_ESM.pdf]

Supplementary information

Whole genome re-sequencing reveals recent signatures of selection in three strains of farmed Nile tilapia (*Oreochromis niloticus*)

María I. Cádiz[12], María E. López[31], Diego Díaz-Domínguez[4], Giovanna Cáceres[12], Grazyella M. Yoshida[1], Daniel Gomez-Uchida[5.6], José M. Yáñez[1,6]*.

[1] Facultad de Ciencias Veterinarias y Pecuarias, Universidad de Chile, Avenida Santa Rosa 11735, 8820808, La Pintana, Santiago, Chile

[2] Programa de Doctorado en Ciencias Silvoagropecuarias y Veterinarias, Campus Sur, Universidad de Chile, Santa Rosa 11315, La Pintana, Santiago, Chile. CP: 8820808.

[3] Department of Animal Breeding and Genetics, Swedish University of Agricultural Sciences, Uppsala, Sweden.

[4] Departamento de Ciencias de la Computación, Universidad de Chile.

[5] Facultad de Ciencias Naturales y Oceanográficas, Universidad de Concepción, Concepción, Chile.

[6] Núcleo Milenio INVASAL, Concepción, Chile

*jmayanez@uchile.cl +56-2 29785533 (Corresponding Author).

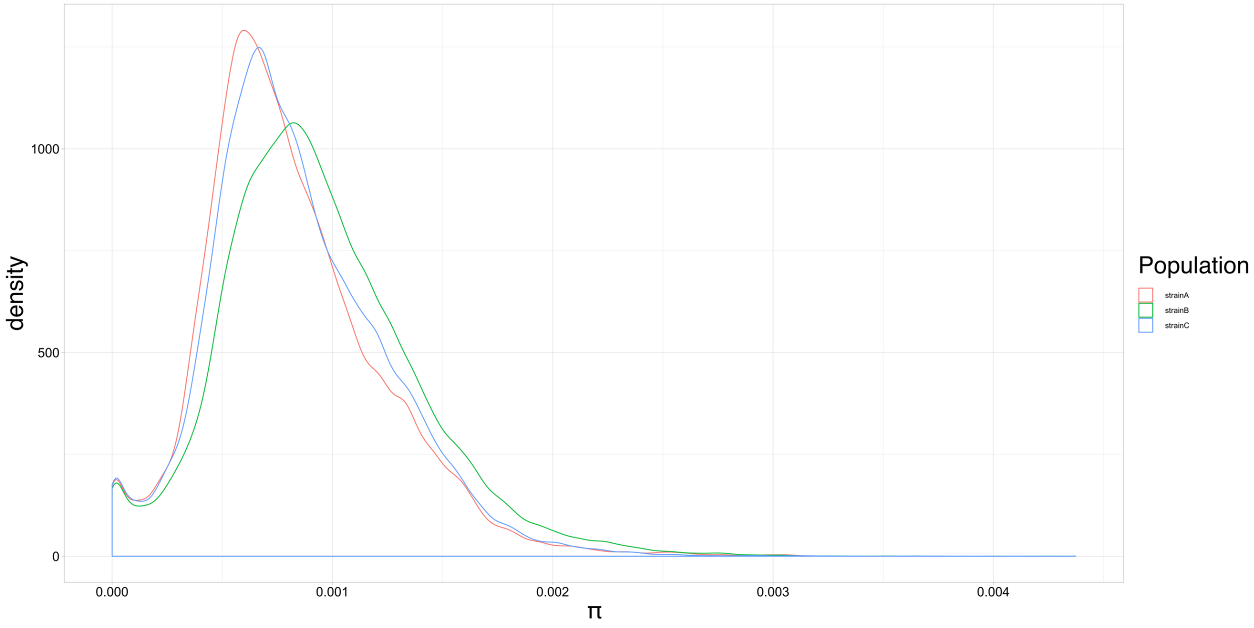

Supplementary Figure S1. Nucleotide diversity of strain A (red line), B (green line) and C (blue line) of Nile tilapia.

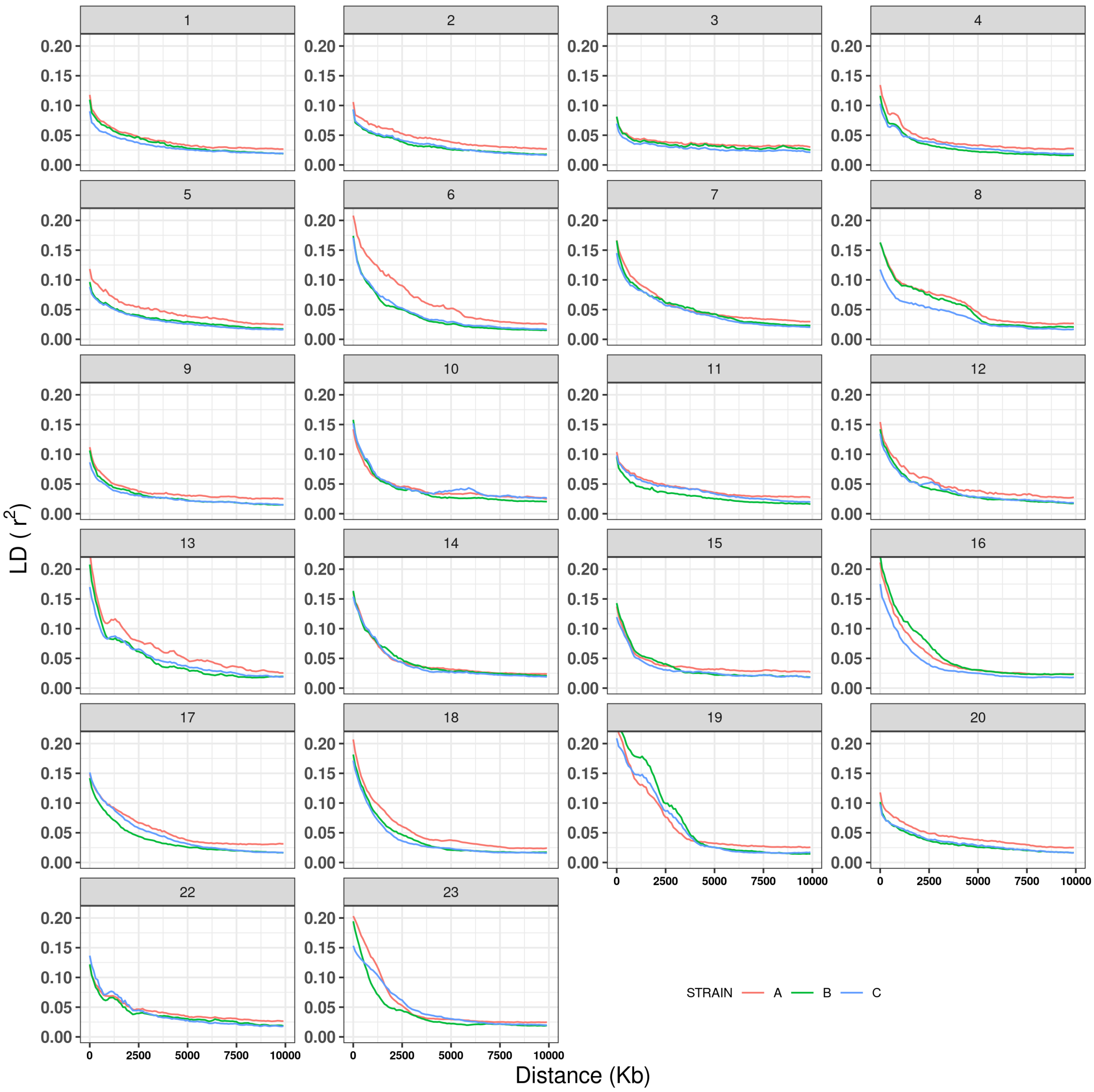

Supplementary Figure S2. Decay of average linkage disequilibrium (r^2) over distance across in each chromosome of three strain of Nile tilapia.

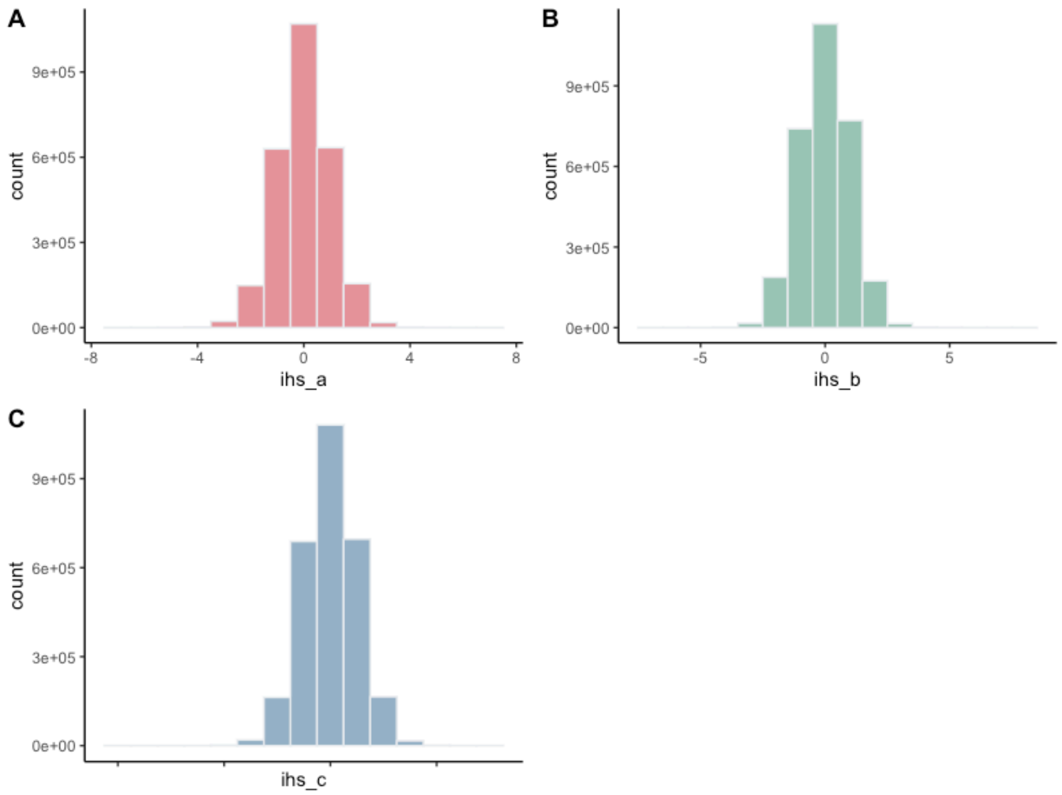

Supplementary Figure S3. Histograms showing the distribution of the iHS values in the three strain of Nile tilapia (A, B and C).

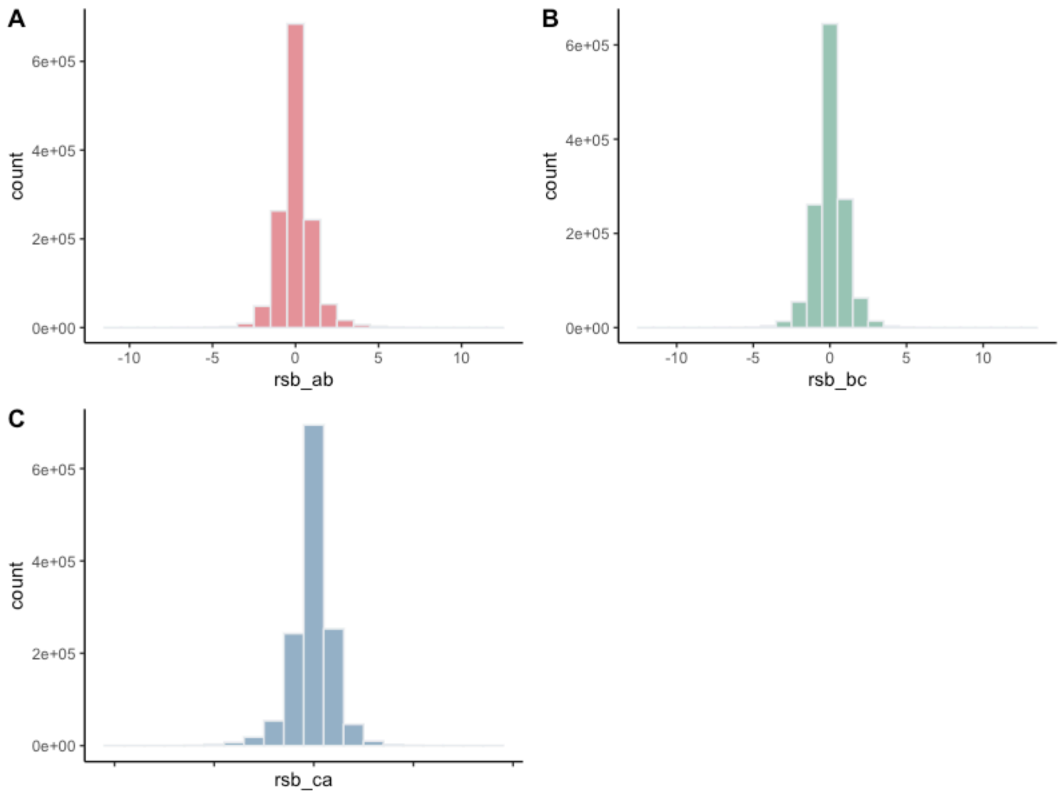

Supplementary Figure S4. Histograms showing the distribution of the Rsb values in the three strain of Nile tilapia (A, B and C).

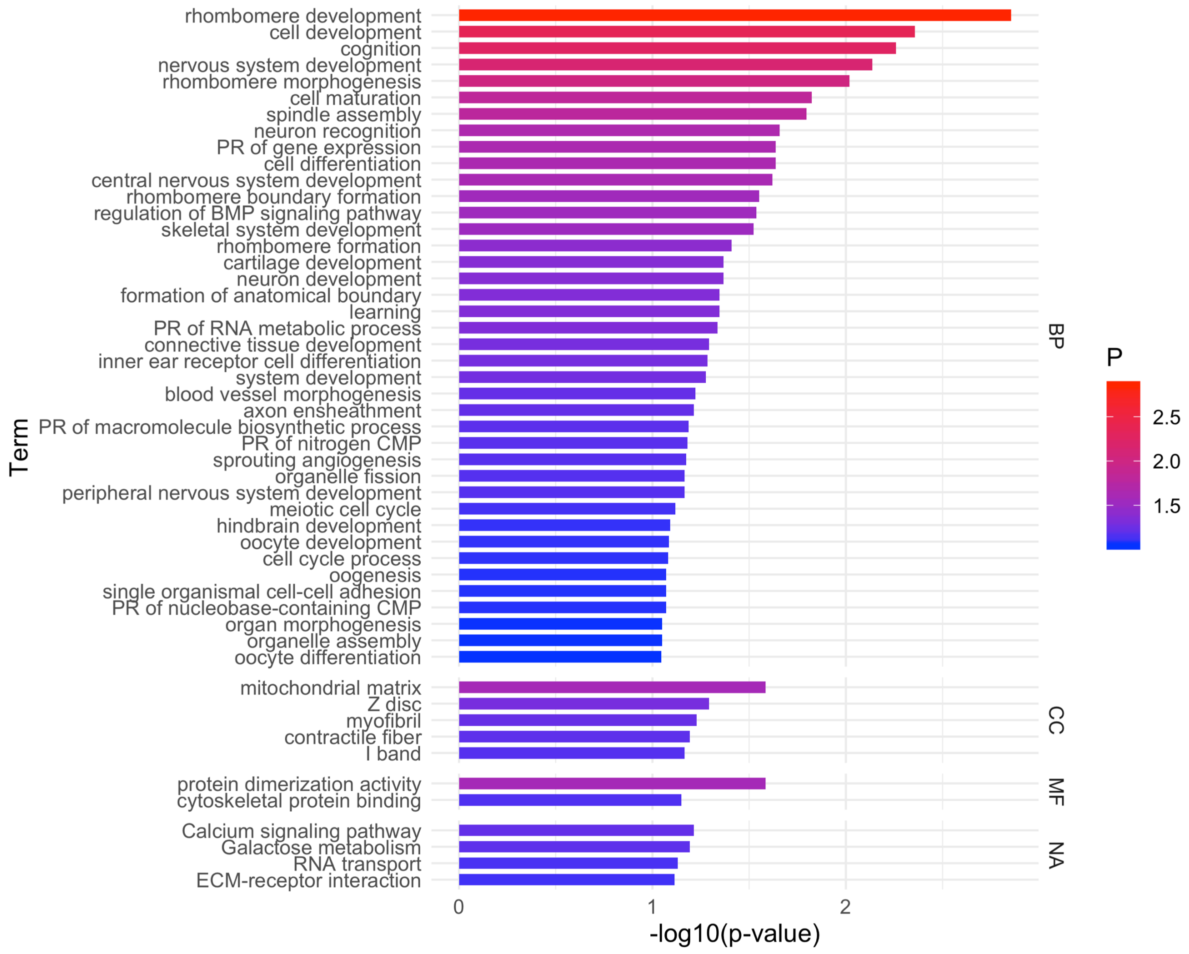

Supplementary Figure S5. Enrichment analysis for GO and KEGG pathways term for strain A by DAVID. Each bar represents the -log10(p-value) for term.

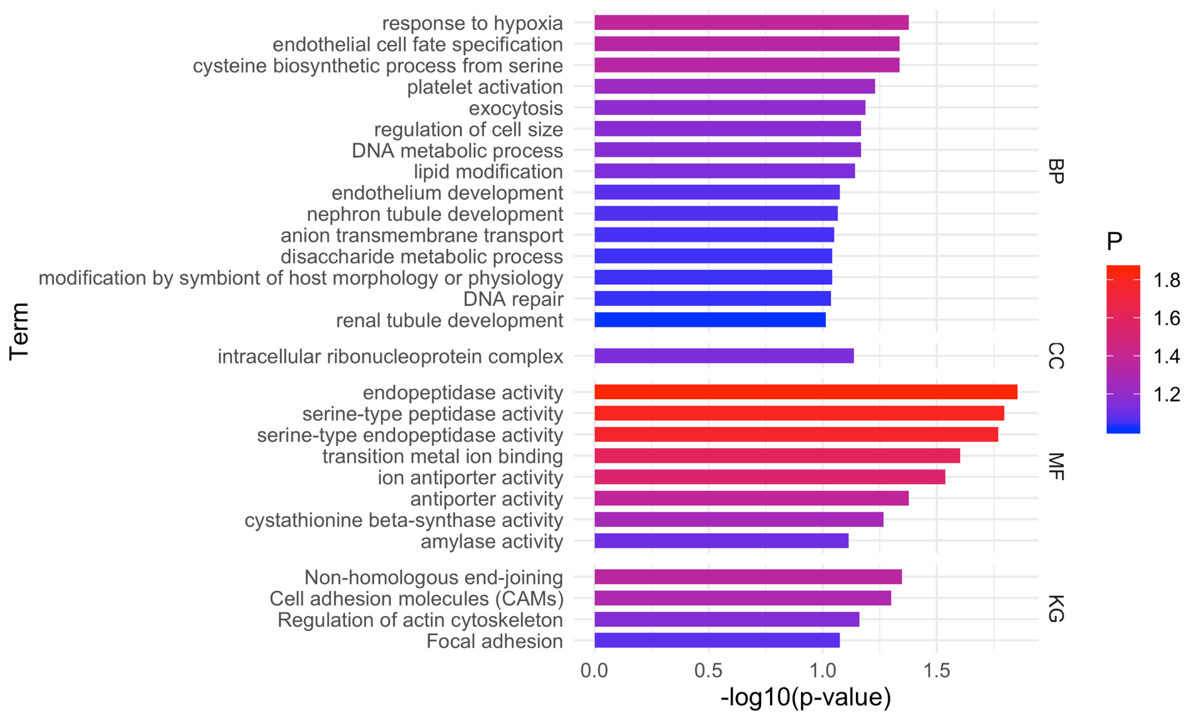

Supplementary Figure S6. Enrichment analysis for GO and KEGG pathways term for strain B by DAVID. Each bar represents the -log10(p-value) for term.

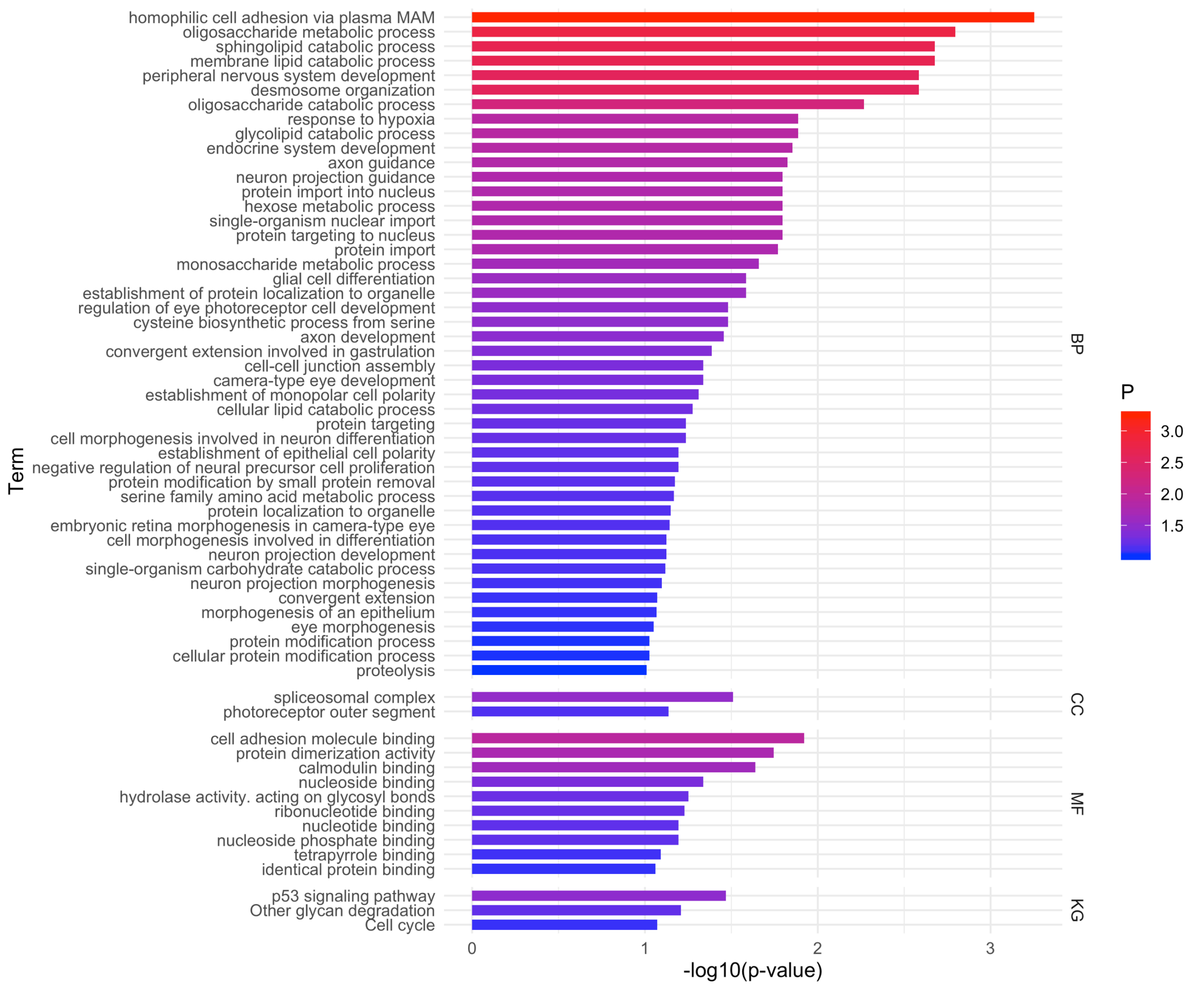

Supplementary Figure S7. Enrichment analysis for GO and KEGG pathways term for strain C by DAVID. Each bar represents the -log10(p-value) for term.
