# Supplementary material for: Whole genome re-sequencing reveals recent signatures of selection in three strains of farmed Nile tilapia (Oreochromis niloticus)
Source: Sci Rep. 2020 Jul 13;10:11514. doi: 10.1038/s41598-020-68064-5 (PMC7359307; doi:10.1038/s41598-020-68064-5)
Supplement: Supplementary file 3 — Supplementary table S1 [file 41598_2020_68064_MOESM3_ESM.pdf]

Supplementary information

Whole genome re-sequencing reveals recent signatures of selection in three strains of farmed Nile tilapia (*Oreochromis niloticus*)

María I. Cádiz[12], María E. López[31], Diego Díaz-Domínguez[4], Giovanna Cáceres[12], Grazyella M. Yoshida[1], Daniel Gomez-Uchida[5,6], José M. Yáñez[1,6]*.

[1] Facultad de Ciencias Veterinarias y Pecuarias, Universidad de Chile, Avenida Santa Rosa 11735, 8820808, La Pintana, Santiago, Chile

[2] Programa de Doctorado en Ciencias Silvoagropecuarias y Veterinarias, Campus Sur, Universidad de Chile, Santa Rosa 11315, La Pintana, Santiago, Chile. CP: 8820808.

[3] Department of Animal Breeding and Genetics, Swedish University of Agricultural Sciences, Uppsala, Sweden.

[4] Departamento de Ciencias de la Computación, Universidad de Chile.

[5] Facultad de Ciencias Naturales y Oceanográficas, Universidad de Concepción, Concepción, Chile.

[6] Núcleo Milenio INVASAL, Concepción, Chile

*jmayanez@uchile.cl +56-2 29785533 (Corresponding Author).

Supplementary Table S1. Values of LD in each chromosome

LG	*LENGHT_CHR*	*Nº SNPs*	*Size (Mb)*	*Strain A (r2)*	*Strain B (r2)*	*Strain C (r2)*
1	40435846	4834	40.44	0.04	0.04	0.03
2	36387328	4554	36.39	0.05	0.03	0.03
3	87460812	12693	87.46	0.04	0.03	0.03
4	35486549	6059	35.49	0.04	0.03	0.03
5	38392377	4700	38.39	0.05	0.04	0.03
6	42413575	7097	42.41	0.07	0.04	0.04
7	64742779	9330	64.74	0.06	0.05	0.05
8	30425071	5682	30.43	0.06	0.06	0.04
9	35732094	4368	35.73	0.04	0.03	0.03
10	34693819	5108	34.69	0.04	0.04	0.05
11	39220051	5119	39.22	0.04	0.03	0.04
12	38544723	6028	38.54	0.05	0.04	0.04
13	34731073	5425	34.73	0.07	0.05	0.05
14	40097775	6531	40.1	0.05	0.05	0.04
15	39687636	4842	39.69	0.04	0.04	0.03
16	35964573	6532	35.96	0.06	0.06	0.04
17	38808393	5736	38.81	0.06	0.04	0.05
18	38606253	6762	38.61	0.06	0.04	0.04
19	30936577	4638	30.94	0.07	0.08	0.07
20	36928077	4552	36.93	0.05	0.03	0.04
22	39180775	6130	39.18	0.04	0.04	0.04
23	45607106	6599	45.61	0.06	0.05	0.05

LG: Linkage group

LENGHT_CHR: chromosome longitude

Nº SNPs: Numer of SNPs

Size (Mb): Size in mega base (Mb)

Strain A (r2): values of r2 to strain A

Strain B (r2): values of r2 to strain B

Strain C (r2): values of r2 to strain C
