# Supplementary material for: Whole genome re-sequencing reveals recent signatures of selection in three strains of farmed Nile tilapia (Oreochromis niloticus)
Source: Sci Rep. 2020 Jul 13;10:11514. doi: 10.1038/s41598-020-68064-5 (PMC7359307; doi:10.1038/s41598-020-68064-5)
Supplement: Supplementary file 4 — Supplementary table S2 [file 41598_2020_68064_MOESM4_ESM.pdf]

Supplementary information

Whole genome re-sequencing reveals recent signatures of selection in three strains of farmed Nile tilapia (*Oreochromis niloticus*)

María I. Cádiz[12], María E. López[31], Diego Díaz-Domínguez[4], Giovanna Cáceres[12], Grazyella M. Yoshida[1], Daniel Gomez-Uchida[5.6], José M. Yáñez[1,6]*.

[1] Facultad de Ciencias Veterinarias y Pecuarias, Universidad de Chile, Avenida Santa Rosa 11735, 8820808, La Pintana, Santiago, Chile

[2] Programa de Doctorado en Ciencias Silvoagropecuarias y Veterinarias, Campus Sur, Universidad de Chile, Santa Rosa 11315, La Pintana, Santiago, Chile. CP: 8820808.

[3] Department of Animal Breeding and Genetics, Swedish University of Agricultural Sciences, Uppsala, Sweden.

[4] Departamento de Ciencias de la Computación, Universidad de Chile.

[5] Facultad de Ciencias Naturales y Oceanográficas, Universidad de Concepción, Concepción, Chile.

[6] Núcleo Milenio INVASAL, Concepción, Chile

*jmayanez@uchile.cl +56-2 29785533 (Corresponding Author).

Supplementary Table S2**.** SNPs detected by iHS, Rsb and FST by chromosome in each strain.

	iHS			***Rsb***			***Fst***					
LG	***A***	***B***	***C***	***A***	***B***	***C***	***AB***		***BC***		***CA***	
							W	***S***	*W*	***S***	*W*	*S*
1	1	7			9	5						
2		2		181	62	45					36	1356
3	11	36	21	36	255	109						
4			1	6	28	5						
5				36	3	43	33	2449	23	1566	12	1021
6	1	2	1	117	15	18			63	4853	104	7214
7	4	3		1	1	125						
8	1				1						7	536
9	8	5	2	28	54	14			23	1626		
10			2		1				11	857		
11					14	9						
12	1			1	49	8						
13	5	9	2	846	11	9						
14	7	2			1	8	15	714	1	96		
15				4	2	141					5	144
16	1			2	2	7						
17				26	11	4	7	487	45	3987	10	1405
18	2		1	1	35	81						
19		2		2	40		119	4704	8	859		
20		2			1	2						
22	17	3			12	7						
23					15	9						
	**59**	**73**	**30**	**1287**	**622**	**649**	**174**	**8354**	**174**	**13844**	**174**	**13844**

LG: Linkage group

W: windows for chromosome

S: SNPs for windows and chromosome
