# Supplementary material for: Whole genome re-sequencing reveals recent signatures of selection in three strains of farmed Nile tilapia (Oreochromis niloticus)
Source: Sci Rep. 2020 Jul 13;10:11514. doi: 10.1038/s41598-020-68064-5 (PMC7359307; doi:10.1038/s41598-020-68064-5)
Supplement: Supplementary file 5 — Supplementary table S3 [file 41598_2020_68064_MOESM5_ESM.pdf]

Supplementary information

Whole genome re-sequencing reveals recent signatures of selection in three strains of farmed Nile tilapia (*Oreochromis niloticus*)

María I. Cádiz[12], María E. López[31], Diego Díaz-Domínguez[4], Giovanna Cáceres[12], Grazyella M. Yoshida[1], Daniel Gomez-Uchida[5.6], José M. Yáñez[1,6]*.

[1] Facultad de Ciencias Veterinarias y Pecuarias, Universidad de Chile, Avenida Santa Rosa 11735, 8820808, La Pintana, Santiago, Chile

[2] Programa de Doctorado en Ciencias Silvoagropecuarias y Veterinarias, Campus Sur, Universidad de Chile, Santa Rosa 11315, La Pintana, Santiago, Chile. CP: 8820808.

[3] Department of Animal Breeding and Genetics, Swedish University of Agricultural Sciences, Uppsala, Sweden.

[4] Departamento de Ciencias de la Computación, Universidad de Chile.

[5] Facultad de Ciencias Naturales y Oceanográficas, Universidad de Concepción, Concepción, Chile.

[6] Núcleo Milenio INVASAL, Concepción, Chile

*jmayanez@uchile.cl +56-2 29785533 (Corresponding Author).

Supplementary Table S3. List of all genes detected by the iHS method in each strain (A, B and C).

LG	*Initial pos.*	*Final pos.*	*iHS*	*Gene symbol*	*Gene name*	*Strain*	*LG3*
NC_031965.2	8285625	8353727	8.076	arnt2	aryl hydrocarbon receptor nuclear translocator 2	A	
NC_031965.2	8320915	8329770	8.076	LOC112842401	syncytin-A-like	A	
NC_031965.2	8354683	8376556	8.076	ctxnd1	cortexin domain containing 1	A	
NC_031965.2	8384336	8403332	8.076	fah	fumarylacetoacetase	A	
NC_031965.2	8438905	8442626	8.076	lto1	LTO1%2C ABCE1 maturation factor	A	
NC_031965.2	8508660	8512284	8.076	fgf3	fibroblast growth factor 3	A	
NC_031965.2	8515727	8525847	8.076	fgf4	fibroblast growth factor 4	A	
NC_031967.2	33138140	33141870	7.448	LOC100695087	tripartite motif-containing protein 16-like	A	
NC_031967.2	33143167	33214249	7.448	col4a5	collagen alpha-5(IV) chain	A	
NC_031967.2	33215777	33358387	7.448	col4a6	collagen alpha-6(IV) chain	A	
NC_031967.2	38541326	38574710	10.129	LOC100706439	kinesin-like protein KIF1C	A	ABC
NC_031967.2	38580689	38603752	10.129	LOC100705911	cysteinyl leukotriene receptor 1-like	A	ABC
NC_031967.2	38621206	38625268	10.129	LOC102075710	myelin-oligodendrocyte glycoprotein	A	ABC
NC_031967.2	38644422	38704304	10.129	LOC102076476	ankyrin repeat domain-containing protein 46	A	ABC
NC_031967.2	43637548	43648417	8.374	LOC102076758	butyrophilin-like protein 8	A	
NC_031967.2	43650400	43657050	8.374	LOC100696502	butyrophilin subfamily 2 member A2	A	
NC_031967.2	43666551	43676574	8.374	LOC100695187	aggrecan core protein	A	
NC_031967.2	43690497	43692491	8.374	LOC100696769	ladderlectin	A	ABC
NC_031967.2	43695767	43705821	8.374	LOC102077851	B-cell receptor CD22	A	
NC_031967.2	43714027	43717786	8.374	LOC109203684	zinc finger MYM-type protein 1-like	A	
NC_031967.2	43718486	43722508	8.374	LOC109194332	zinc finger MYM-type protein 1-like	A	
NC_031967.2	43772297	43788568	8.374	LOC112845607	lamin-A-like	A	
NC_031967.2	46859269	46890096	10.091	LOC102075779	interferon-induced protein 44	A	ABC
NC_031967.2	46940017	46945392	10.091	LOC109196641	C-type lectin BJcuL-like	A	ABC
NC_031967.2	47092303	47093821	10.091	LOC100690229	ladderlectin	A	ABC
NC_031967.2	71908519	71915925	7.898	LOC102079713	low affinity immunoglobulin gamma Fc region receptor II-like	A	
NC_031967.2	71977396	71978581	7.898	LOC109201475	zinc finger BED domain-containing protein 1-like	A	AC
NC_031967.2	72015919	72021730	7.898	LOC109196951	low affinity immunoglobulin gamma Fc region receptor II-like	A	
NC_031967.2	85638752	85640558	8.583	LOC112846044	protein NLRC3-like	A	AB
NC_031967.2	85671063	85688192	8.583	LOC106097519	protein NLRC3-like	A	
NC_031967.2	85787476	85797543	8.583	LOC112846385	protein NLRC5-like	A	
NC_031967.2	85799626	85808562	8.583	LOC109201165	NLR family CARD domain-containing protein 3	A	

NC_031967.2	85830808	85888752	8.583	LOC102082314	protein NLRC3-like	A	
NC_031967.2	85874553	85888752	8.583	LOC102082247	protein NLRC3	A	
NC_031967.2	87041325	87065594	8.445	LOC109197042	NLR family CARD domain-containing protein 3-like	A	ABC
NC_031967.2	87090875	87093871	8.445	LOC109201620	nesprin-2-like	A	ABC
NC_031967.2	87131491	87135794	8.445	LOC102078820	NLR family CARD domain-containing protein 3-like	A	
NC_031971.2	4813492	4822835	7.851	LOC100702690	E3 ubiquitin-protein ligase TRIM41	A	
NC_031971.2	4827387	4846869	7.851	LOC106096490	zinc finger protein 638	A	
NC_031972.2	30836850	30861606	8.363	col27a1	collagen alpha-1(XXVII) chain	A	
NC_031972.2	30862863	31068239	10.471	LOC100700442	voltage-dependent N-type calcium channel subunit alpha-1B	A	
NC_031972.2	31070333	31086850	8.363	ehmt1	euchromatic histone lysine methyltransferase 1%2C	A	
NC_031973.2	7014505	7032261	7.440	LOC100706950	sodium channel protein type 4 subunit alpha A	A	
NC_031973.2	7036299	7040585	7.440	LOC100691830	cilia- and flagella-associated protein 251	A	
NC_031973.2	7041167	7043701	7.440	LOC100692098	redox-regulatory protein FAM213A	A	
NC_031973.2	7062788	7073464	7.440	pdzd7	PDZ domain containing 7%2C	A	
NC_031973.2	7073460	7118741	7.440	LOC100692643	leucine zipper putative tumor suppressor 2 homolog	A	
NC_031973.2	7132920	7138470	7.440	twnk	twinkle mtDNA helicase	A	
NC_031973.2	7138595	7145549	7.440	mrpl43	39S ribosomal protein L43%2C mitochondrial	A	
NC_031973.2	7146014	7202239	7.440	sema4g	semaphorin 4G	A	
NC_031973.2	7205013	7216210	7.440	mfsd13a	major facilitator superfamily domain containing 13A%2C	A	
NC_031973.2	7217209	7217323	7.440	LOC112847734	U5 spliceosomal RNA	A	
NC_031973.2	7218665	7250129	7.440	arhgap27	Rho GTPase activating protein 27%2C	A	
NC_031973.2	7250732	7261023	7.440	vps25	vacuolar protein sorting 25 homolog	A	
NC_031973.2	7261669	7264505	7.440	wnk4	WNK lysine deficient protein kinase 4%2C	A	
NC_031974.2	30021807	30048349	7.676	LOC100711398	glucose-fructose oxidoreductase domain-containing protein 1	A	
NC_031974.2	30059889	30132959	10.668	LOC100706669	solute carrier family 12 member 7	A	
NC_031974.2	30195593	30233398	10.668	LOC100704460	calcium/calmodulin-dependent 3'%2C5'-cyclic nucleotide phosphodiesterase 1A	A	
NC_031974.2	35020664	35023791	7.931	LOC100696490	neurogenic differentiation factor 6-A	A	
NC_031974.2	35029483	35058742	7.931	itprid1	ITPR interacting domain containing 1%2C	A	
NC_031974.2	7530984	7599969	8.507	LOC100707500	disco-interacting protein 2 homolog C	A	
NC_031974.2	7603048	7651893	8.507	LOC100707237	vasoactive intestinal polypeptide receptor 2	A	
NC_031974.2	7656264	7671129	8.507	LOC112847889	zinc finger protein 726-like	A	
NC_031974.2	7672915	7675091	8.507	LOC102080242	vegetative cell wall protein gp1	A	
NC_031974.2	7745546	7762222	8.507	LOC112847955	zinc finger protein 271-like	A	
NC_031974.2	7762268	7767304	8.507	LOC112847891	putative nuclease HARBI1	A	

NC_031977.2	8427812	8439917	7.579	LOC109204494	glutathione hydrolase 5 proenzyme	A	
NC_031977.2	8460704	8474528	7.579	LOC106097761	glutathione hydrolase 5 proenzyme	A	
NC_031977.2	8476951	8480483	7.579	LOC100710238	glutathione hydrolase 5 proenzyme-like	A	
NC_031977.2	8483512	8489627	7.579	asphd2	aspartate beta-hydroxylase domain containing 2%2C	A	
NC_031977.2	8491518	8503048	7.579	LOC100709699	calcium-binding protein 1	A	
NC_031977.2	8504675	8516323	7.579	mlec	malectin	A	
NC_031977.2	8525181	8532017	7.579	hps4	HPS4%2C biogenesis of lysosomal organelles complex 3 subunit 2%2C	A	
NC_031977.2	8540586	8542386	7.579	LOC100709161	C-type lectin domain family 10 member A	A	
NC_031977.2	8542086	8544330	7.579	pla2g1b	phospholipase A2	A	
NC_031977.2	8549629	8551038	7.579	LOC100705146	phospholipase A2%2C minor isoenzyme	A	
NC_031977.2	8558647	8563903	7.579	LOC100704877	5'-AMP-activated protein kinase subunit beta-1	A	
NC_031977.2	8565792	8568962	7.579	LOC100708621	transmembrane protein 233-like	A	
NC_031977.2	8570315	8591206	7.579	msi1	RNA-binding protein Musashi homolog 1	A	
NC_031977.2	8594335	8601645	7.579	hnf1a	HNF1 homeobox A	A	
NC_031977.2	8601754	8605155	7.579	lg12h12orf43	linkage group 12 C12orf43 homolog	A	
NC_031977.2	8606114	8611981	7.579	unc119b	protein unc-119 homolog B	A	
NC_031977.2	8612289	8615760	7.579	pop5	POP5 homolog%2C ribonuclease P/MRP subunit	A	
NC_031977.2	8617336	8624039	7.579	rnf10	RING finger protein 10	A	
NC_031977.2	8624480	8653616	7.579	LOC100707539	calponin homology domain-containing protein DDB_G0272472	A	
NC_031977.2	8653727	8656248	7.579	LOC100707280	beta-crystallin B3	A	
NC_031977.2	8659415	8661124	7.579	LOC100703162	beta-crystallin B2	A	
NC_031977.2	8661255	8662417	7.579	ctu1	cytoplasmic tRNA 2-thiolation protein 1	A	
NC_031978.2	32280754	32281736	8.489	eno4	enolase 4	A	
NC_031978.2	32303810	32404118	8.110	hspa12a	heat shock 70 kDa protein 12A	A	
NC_031978.2	32341331	32342386	8.110	LOC109204836	transcription factor Adf-1-like	A	
NC_031978.2	32432465	32433078	8.110	LOC109199971	probable peroxisomal membrane protein PEX13	A	
NC_031978.2	32504000	32530754	8.489	birc6	baculoviral IAP repeat containing 6%2C	A	
NC_031979.2	22686046	22692260	7.532	ppp1r37	protein phosphatase 1 regulatory subunit 37	A	
NC_031979.2	22694873	22700021	7.532	LOC102081410	zinc homeostasis factor 1	A	
NC_031979.2	22701808	22706656	7.532	mrpl28	39S ribosomal protein L28%2C mitochondrial	A	
NC_031979.2	22720271	22730649	7.532	LOC100712393	galaxin-like	A	
NC_031979.2	22878519	22889213	7.532	LOC102075940	galaxin	A	
NC_031982.2	20389124	20560465	7.754	b4galt2	beta-1%2C4-galactosyltransferase 2	A	
NC_031982.2	20561013	20565403	7.754	LOC100692768	von Willebrand factor C domain-containing protein 2-like	A	

NC_031982.2	20615917	20618751	7.754	LOC100691961	protein THEM6	A	
NC_031982.2	20620553	20623090	7.754	LOC100691691	protein THEM6	A	
NC_031982.2	20630130	20639124	7.754	LOC102082158	tripartite motif-containing protein 47	A	
NC_031982.2	4626677	4685628	7.797	LOC100701150	cadherin-6	A	
NC_031985.2	36097480	36098761	7.682	hsf1	heat shock factor protein 1	A	
NC_031985.2	36108249	36123669	7.502	wdyhv1	WDYHV motif containing 1	A	
NC_031985.2	36124482	36133950	7.502	bop1	block of proliferation 1	A	
NC_031985.2	36135378	36143267	7.502	lrp12	LDL receptor related protein 12	A	
NC_031985.2	36148714	36215398	7.502	LOC100693583	regulating synaptic membrane exocytosis protein 2	A	
NC_031985.2	36249984	36268986	7.502	LOC100694839	large proline-rich protein BAG6	A	
NC_031985.2	36270399	36278928	7.502	lg22h6orf136	linkage group 22 C6orf136 homolog%2C	A	
NC_031985.2	36283411	36300637	7.502	abcf1	ATP binding cassette subfamily F member 1	A	
NC_031985.2	36300633	36303325	7.502	mrps18b	28S ribosomal protein S18b%2C mitochondrial	A	
NC_031985.2	36303466	36314183	7.502	ppp1r10	serine/threonine-protein phosphatase 1 regulatory subunit 10	A	
NC_031985.2	36318174	36320292	7.502	agr2	anterior gradient 2%2C protein disulphide isomerase family member	A	
NC_031985.2	36322837	36335844	7.502	tspan13	tetraspanin 13	A	
NC_031985.2	37622671	37666235	7.454	LOC100698901	glucocorticoid-induced transcript 1 protein	A	
NC_031985.2	37697390	37699229	7.454	LOC112843665	18S ribosomal RNA	A	
NC_031985.2	37699766	37699919	7.454	LOC112843645	5.8S ribosomal RNA	A	
NC_031985.2	37700417	37704343	7.454	LOC112843668	28S ribosomal RNA	A	
NC_031985.2	37802932	37819885	7.454	LOC100691851	SH3 domain-binding protein 5-like	A	
NC_031985.2	37820454	37835623	7.466	gtf2h4	general transcription factor IIH subunit 4	A	
NC_031987.2	6821977	6830507	7.640	LOC109197346	disco-interacting protein 2 homolog C-like	A	
NC_031987.2	6836293	6840245	7.640	LOC109197383	disco-interacting protein 2 homolog C-like	A	
NC_031987.2	6904246	6915158	7.640	LOC100690152	lanosterol synthase	A	
NC_031987.2	6915521	6929088	7.640	mcm3ap	germinal-center associated nuclear protein	A	
NC_031987.2	6929123	6935869	7.640	ybey	endoribonuclease YbeY	A	
NC_031987.2	6936160	6950339	7.640	usp37	ubiquitin carboxyl-terminal hydrolase 37	A	
NC_031987.2	6950402	6958624	7.640	cnot9	CCR4-NOT transcription complex subunit 9	A	
NC_031987.2	6961312	6964435	7.640	asnsd1	asparagine synthetase domain containing 1	A	
NC_031987.2	6964352	6966049	7.640	asdurf	ASNSD1 upstream open reading frame protein	A	
NC_031987.2	6974373	6986653	7.640	slc40a1	solute carrier family 40 member 1	A	
NC_031987.2	6991921	7001141	7.640	wdr75	WD repeat domain 75	A	
NC_031965.2	8285376	8353727	9.299	arnt2	aryl hydrocarbon receptor nuclear translocator 2	B	

NC_031965.2	8320915	8329770	10.831	LOC112842401	syncytin-A-like	B	
NC_031965.2	8354683	8376556	10.831	ctxnd1	cortexin domain containing 1	B	
NC_031965.2	8384336	8403332	10.831	fah	fumarylacetoacetase	B	
NC_031965.2	8438905	8442626	10.831	lto1	LTO1%2C ABCE1 maturation factor	B	
NC_031965.2	8508660	8512284	10.831	fgf3	fibroblast growth factor 3	B	
NC_031965.2	8515727	8525847	10.831	fgf4	fibroblast growth factor 4	B	
NC_031966.2	2474399	2488598	7.522	LOC100711332	ETS-related transcription factor Elf-2	B	
NC_031966.2	2503635	2506656	7.522	ndufc1	NADH dehydrogenase [ubiquinone] 1 subunit C1%2C mitochondrial	B	
NC_031966.2	2506722	2557402	7.522	LOC100695335	N-alpha-acetyltransferase 15%2C NatA auxiliary subunit	B	
NC_031966.2	2565944	2589325	7.522	LOC100711605	malate synthase%2C	B	
NC_031966.2	3074068	3083216	7.462	LOC100696535	rod cGMP-specific 3'%2C5'-cyclic phosphodiesterase subunit alpha	B	
NC_031966.2	3076918	3093565	7.462	slc26a2	solute carrier family 26 member 2%2C	B	
NC_031966.2	3144904	3186616	7.462	LOC100691272	5-hydroxytryptamine receptor 4	B	
NC_031967.2	25526420	25776420	7.611	LOC109194483	titin	B	
NC_031967.2	25565338	25614203	7.611	LOC112846230	Fc receptor-like protein 5	B	
NC_031967.2	25772119	25773679	7.611	LOC106096638	coiled-coil domain-containing protein 106-like	B	
NC_031967.2	29710591	29716904	11.446	LOC109194277	E3 ubiquitin-protein ligase TRIM39-like	B	
NC_031967.2	29732366	29734299	11.446	LOC109199711	erythroid membrane-associated protein	B	
NC_031967.2	29793262	29800581	11.446	LOC112843099	ribonuclease inhibitor-like	B	
NC_031967.2	29804348	29809428	11.446	LOC102082638	E3 ubiquitin-protein ligase TRIM39	B	
NC_031967.2	29830128	29834194	11.446	LOC100695258	CMRF35-like molecule 9	B	
NC_031967.2	29861829	29943994	11.446	LOC102079294	polymeric immunoglobulin receptor	B	
NC_031967.2	29902613	29907759	11.446	LOC109197601	nuclear factor 7%2C ovary-like	B	
NC_031967.2	29942615	29943994	11.446	LOC112843390	polymeric immunoglobulin receptor-like	B	
NC_031967.2	38541326	38574710	10.019	LOC100706439	kinesin-like protein KIF1C	B	ABC
NC_031967.2	38580689	38603752	10.019	LOC100705911	cysteinyl leukotriene receptor 1-like	B	ABC
NC_031967.2	38621206	38625268	10.019	LOC102075710	myelin-oligodendrocyte glycoprotein	B	ABC
NC_031967.2	38644422	38704304	10.019	LOC102076476	ankyrin repeat domain-containing protein 46	B	ABC
NC_031967.2	46859269	46890096	9.168	LOC102075779	interferon-induced protein 44	B	ABC
NC_031967.2	46940017	46945392	9.168	LOC109196641	C-type lectin BJcuL-like	B	ABC
NC_031967.2	47092303	47093821	9.168	LOC100690229	ladderlectin	B	ABC
NC_031967.2	50120446	50149398	9.654	LOC109199757	butyrophilin subfamily 3 member A2-like	B	
NC_031967.2	50168462	50182878	9.654	LOC109199763	centrosomal protein of 290 kDa-like	B	
NC_031967.2	50299669	50307245	9.654	LOC102076565	acidic repeat-containing protein	B	

NC_031967.2	50308981	50315159	9.654	fgb	fibrinogen beta chain	B	
NC_031967.2	50314856	50319673	9.654	LOC102076387	fibrinogen alpha chain	B	
NC_031967.2	50320733	50329322	9.654	LOC100693197	fibrinogen alpha chain	B	
NC_031967.2	50329169	50338243	9.654	LOC102076188	zinc finger and SCAN domain-containing protein 12	B	
NC_031967.2	52409477	52502241	7.946	LOC100700312	deleted in malignant brain tumors 1 protein	B	BC
NC_031967.2	52515236	52518245	7.946	LOC109202644	Fc receptor-like B	B	BC
NC_031967.2	52635907	52639339	7.946	LOC109199313	nuclear factor 7%2C ovary-like	B	BC
NC_031967.2	52648778	52653076	7.946	LOC112846437	hepatitis A virus cellular receptor 2 homolog	B	BC
NC_031967.2	54159054	54202935	11.503	LOC106097159	low affinity immunoglobulin gamma Fc region receptor II	B	BC
NC_031967.2	54251264	54258518	11.503	LOC102081459	butyrophilin subfamily 2 member A2%2C	B	BC
NC_031967.2	54277348	54285214	11.503	LOC109196578	G2/M phase-specific E3 ubiquitin-protein ligase-like	B	BC
NC_031967.2	54365500	54366701	11.503	LOC112844103	putative nuclease HARBI1	B	BC
NC_031967.2	55308862	55313331	9.157	LOC100689971	butyrophilin subfamily 3 member A2	B	BC
NC_031967.2	55379911	55394599	9.157	LOC102079944	phospholipid transfer protein	B	BC
NC_031967.2	55428369	55430188	9.157	LOC102079855	BPI fold-containing family C protein-like	B	BC
NC_031967.2	67447955	67479941	8.211	LOC112846006	ribonuclease inhibitor-like	B	
NC_031967.2	67491169	67519906	8.211	LOC109199432	NACHT%2C LRR and PYD domains-containing protein 12	B	
NC_031967.2	70138229	70143555	9.182	LOC102078736	selection and upkeep of intraepithelial T-cells protein 7	B	
NC_031967.2	70148210	70154875	9.182	LOC100704735	selection and upkeep of intraepithelial T-cells protein 7-like	B	
NC_031967.2	70191359	70259635	9.182	LOC106096754	cell surface A33 antigen	B	
NC_031967.2	70281074	70299684	9.182	LOC106096749	complement C1q-like protein 4	B	
NC_031967.2	70296398	70299184	9.182	LOC106098827	general transcription factor II-I repeat domain-containing protein 2	B	
NC_031967.2	82108222	82358222	8.568	LOC100692238	H-2 class II histocompatibility antigen%2C E-S beta chain	B	
NC_031967.2	82116157	82118821	8.568	LOC100689964	endoplasmic reticulum chaperone BiP-like	B	
NC_031967.2	82161500	82167968	8.568	LOC100711901	H-2 class II histocompatibility antigen%2C E-S beta chain	B	
NC_031967.2	82169297	82172935	8.568	LOC100697318	RLA class II histocompatibility antigen%2C DP alpha-1 chain	B	
NC_031967.2	82183123	82187005	8.568	LOC100706688	endoplasmic reticulum chaperone BiP-like	B	
NC_031967.2	82191956	82196233	8.568	LOC100706959	endoplasmic reticulum chaperone BiP-like	B	
NC_031967.2	82203803	82207746	8.568	LOC100706421	endoplasmic reticulum chaperone BiP	B	
NC_031967.2	84711354	84728422	7.552	LOC100700611	NACHT%2C LRR and PYD domains-containing protein 12-like	B	
NC_031967.2	84750253	84752598	7.552	LOC112846041	protein NLRC3-like	B	AB
NC_031967.2	84759223	84783608	7.552	LOC112846398	ribonuclease inhibitor-like	B	
NC_031967.2	84811887	84816711	7.552	LOC109201227	G2/M phase-specific E3 ubiquitin-protein ligase	B	

NC_031967.2	84832289	84834378	7.552	LOC109201231	piggyBac transposable element-derived protein 4-like	B	
NC_031967.2	84905218	84917610	7.552	LOC109200011	protein NLRC3-like	B	
NC_031967.2	84944820	84951433	7.552	LOC112846387	protein NLRC3-like	B	
NC_031967.2	87041325	87065594	7.635	LOC109197042	NLR family CARD domain-containing protein 3-like	B	ABC
NC_031967.2	87090875	87093871	7.635	LOC109201620	nesprin-2-like	B	ABC
NC_031967.2	87131491	87136083	7.635	LOC102078820	NLR family CARD domain-containing protein 3-like	B	ABC
NC_031971.2	4813785	4822835	7.918	LOC100702690	E3 ubiquitin-protein ligase TRIM41	B	
NC_031971.2	4827387	4846869	7.794	LOC106096490	zinc finger protein 638	B	
NC_031972.2	30836759	30861606	9.270	col27a1	collagen alpha-1(XXVII) chain	B	
NC_031972.2	30862863	31068239	9.228	LOC100700442	voltage-dependent N-type calcium channel subunit alpha-1B	B	
NC_031972.2	31070333	31086759	9.270	ehmt1	euchromatic histone lysine methyltransferase 1%2C	B	
NC_031974.2	27198454	27203231	7.721	LOC100698997	sialoadhesin-like	B	
NC_031974.2	27241913	27245754	7.721	LOC109199459	vascular cell adhesion protein 1-like	B	
NC_031974.2	27247934	27250453	7.721	LOC109199458	zinc finger MYM-type protein 1-like	B	
NC_031974.2	27259862	27273738	7.721	LOC109194184	sialoadhesin	B	
NC_031974.2	27280213	27282796	7.721	LOC109194538	myeloid cell surface antigen CD33	B	
NC_031974.2	27291862	27294371	7.721	LOC109203477	leukocyte elastase inhibitor-like	B	
NC_031974.2	27295802	27299358	7.721	LOC109194146	transcription factor 7-like 1	B	
NC_031974.2	27300025	27309491	7.721	LOC100712479	leukocyte elastase inhibitor	B	
NC_031974.2	27311063	27322214	7.721	snx16	sorting nexin 16%2C	B	
NC_031974.2	27342424	27367965	7.721	LOC100701023	NEDD4-like E3 ubiquitin-protein ligase WWP1	B	
NC_031974.2	27370882	27374809	7.721	rmdn1	regulator of microtubule dynamics 1	B	
NC_031974.2	27374873	27384663	7.721	LOC100701296	copine-3	B	
NC_031974.2	27388133	27401963	7.721	LOC100701568	copine-3	B	
NC_031974.2	27407240	27417818	7.721	LOC100701839	copine-3	B	
NC_031974.2	30021870	30048349	7.500	LOC100711398	glucose-fructose oxidoreductase domain-containing protein 1	B	
NC_031974.2	30059889	30132959	7.500	LOC100706669	solute carrier family 12 member 7	B	
NC_031974.2	30195593	30233398	7.500	LOC100704460	calcium/calmodulin-dependent 3'%2C5'-cyclic nucleotide phosphodiesterase 1A	B	
NC_031974.2	7530530	7599969	7.748	LOC100707500	disco-interacting protein 2 homolog C	B	
NC_031974.2	7603048	7651893	7.748	LOC100707237	vasoactive intestinal polypeptide receptor 2	B	
NC_031974.2	7656264	7671129	7.748	LOC112847889	zinc finger protein 726-like	B	
NC_031974.2	7672915	7675091	7.748	LOC102080242	vegetative cell wall protein gp1	B	
NC_031974.2	7745546	7762222	7.748	LOC112847955	zinc finger protein 271-like	B	
NC_031974.2	7762268	7767304	7.748	LOC112847891	putative nuclease HARBI1	B	

NC_031978.2	32303810	32404118	7.685	hspa12a	heat shock 70 kDa protein 12A	B	
NC_031978.2	32341331	32342386	7.685	LOC109204836	transcription factor Adf-1-like	B	
NC_031978.2	32432465	32433078	7.685	LOC109199971	probable peroxisomal membrane protein PEX13	B	
NC_031978.2	32504000	32532139	8.317	birc6	baculoviral IAP repeat containing 6%2C	B	
NC_031979.2	17202869	17212237	9.466	LOC106097967	secretory phospholipase A2 receptor%2C	B	
NC_031979.2	17242250	17248048	9.466	LOC100702433	cysteine and histidine-rich domain-containing protein 1	B	
NC_031979.2	17247601	17259451	9.466	folh1b	folate hydrolase 1B	B	
NC_031979.2	17261907	17279924	9.466	nox4	NADPH oxidase 4	B	
NC_031979.2	17280474	17285099	9.466	tyr	tyrosinase	B	
NC_031979.2	17291317	17326200	9.466	grm5	glutamate metabotropic receptor 5%2C	B	
NC_031979.2	35186320	35195005	8.271	LOC100703477	DCN1-like protein 5	B	
NC_031979.2	35197070	35221508	8.271	LOC100703753	cullin-5	B	
NC_031979.2	35221459	35231209	8.271	LOC100710181	solute carrier family 35 member F2	B	
NC_031979.2	35231234	35241035	8.271	LOC100710442	rhomboid-related protein 4	B	
NC_031979.2	35367131	35421556	8.271	myo7a	myosin VIIA	B	
NC_031979.2	35421697	35434054	8.271	LOC100692992	glycerophosphodiester phosphodiesterase domain-containing protein 5	B	
NC_031983.2	18042762	18044937	7.607	yju2	YJU2 splicing factor homolog	B	
NC_031983.2	18046326	18047415	7.607	LOC100708247	cocaine- and amphetamine-regulated transcript protein	B	
NC_031983.2	18047873	18059905	7.607	LOC100702708	microtubule-associated protein 1S	B	
NC_031983.2	18060775	18064987	7.607	LOC100702439	interferon-induced protein 44	B	
NC_031983.2	18065486	18067640	7.607	LOC100707977	growth arrest and DNA damage-inducible protein GADD45 beta	B	
NC_031983.2	18069042	18077829	7.607	gng7	G protein subunit gamma 7%2C	B	
NC_031983.2	18080709	18096175	7.607	LOC100707435	GTP-binding protein Di-Ras1	B	
NC_031983.2	18098614	18106610	7.607	LOC100707170	receptor expression-enhancing protein 5	B	
NC_031983.2	18108470	18155340	7.607	LOC100701627	Ig kappa chain V region Mem5-like	B	
NC_031983.2	18111001	18144919	7.607	LOC112843186	Ig kappa chain V-III region MOPC 63-like	B	
NC_031983.2	18113529	18122395	7.607	LOC100701901	excitatory amino acid transporter 5	B	
NC_031983.2	18123215	18133028	7.607	LOC100706107	ELAV-like protein 1	B	
NC_031983.2	18135766	18140750	7.607	LOC102076783	tetraspanin-3	B	
NC_031983.2	18145239	18146054	7.607	LOC102077194	Ig kappa chain V region 120-like	B	
NC_031983.2	18147703	18194358	7.607	LOC109195905	Ig lambda-1 chain C region-like	B	
NC_031983.2	18152985	18154113	7.607	LOC109195891	immunoglobulin kappa variable 6D-21-like	B	
NC_031983.2	18156107	18163874	7.607	LOC109195882	Ig kappa chain V region 3381-like	B	
NC_031983.2	18159852	18160519	7.607	LOC109195815	immunoglobulin kappa variable 1D-16-like	B	

NC_031983.2	18168721	18169470	7.607	LOC102076682	immunoglobulin kappa variable 4-1-like	B	
NC_031983.2	18169887	18171264	7.607	LOC109195879	Ig lambda chain C region-like	B	
NC_031983.2	18174438	18175024	7.607	LOC109195902	Ig kappa chain V region 3381-like	B	
NC_031983.2	18183234	18184138	7.607	LOC102076501	Ig kappa chain V region 3381-like	B	
NC_031983.2	18191789	18192491	7.607	LOC109195894	Ig kappa chain V-VI region NQ2-48.2.2-like	B	
NC_031983.2	18192523	18205351	7.607	LOC106098051	Ig lambda chain C region-like	B	
NC_031983.2	18195419	18196154	7.607	LOC109195885	Ig kappa chain V region 4135-like	B	
NC_031983.2	18202072	18202716	7.607	LOC112843190	Ig kappa chain V-VI region NQ2-48.2.2-like	B	
NC_031983.2	18209569	18227968	7.607	LOC109195812	Ig kappa chain C region%2C B allele-like	B	
NC_031983.2	18212931	18213590	7.607	LOC109195896	Ig kappa chain V region BS-5-like	B	
NC_031983.2	18218162	18218744	7.607	LOC109195895	Ig kappa chain V region BS-5-like	B	
NC_031983.2	18225637	18226496	7.607	LOC109195901	Ig kappa chain V region BS-5-like	B	
NC_031983.2	18228958	18229841	7.607	LOC102075657	immunoglobulin kappa variable 4-1-like	B	
NC_031983.2	18230190	18239551	7.607	LOC109195906	Ig kappa chain C region%2C B allele-like	B	
NC_031983.2	18233073	18234249	7.607	LOC109195811	Ig kappa chain V region 3547-like	B	
NC_031983.2	18236821	18237411	7.607	LOC109195883	immunoglobulin kappa variable 4-1-like	B	
NC_031983.2	18243311	18244028	7.607	LOC109195893	Ig kappa chain V region BS-5-like	B	
NC_031983.2	18245267	18245593	7.607	LOC109195904	Ig lambda chain C region-like	B	
NC_031983.2	18250139	18251779	7.607	LOC102083289	Ig kappa-b4 chain C region-like	B	
NC_031983.2	18254275	18254808	7.607	LOC109195810	Ig kappa chain V region 3381-like	B	
NC_031983.2	18258226	18260068	7.607	LOC106098056	Ig kappa chain C region%2C B allele-like	B	
NC_031983.2	18262196	18263833	7.607	LOC109195880	Ig kappa chain C region%2C B allele-like	B	
NC_031983.2	18267088	18267935	7.607	LOC109195890	Ig kappa chain V region BS-5-like	B	
NC_031983.2	18268407	18290954	7.607	LOC100699464	Ig kappa chain C region%2C B allele-like	B	
NC_031983.2	18276460	18277979	7.607	LOC109195876	Ig lambda chain C region-like	B	
NC_031983.2	18280161	18280899	7.607	LOC109195903	immunoglobulin kappa variable 1-6-like	B	
NC_031983.2	18283236	18284231	7.607	LOC109195888	Ig kappa chain V region 3381-like	B	
NC_031983.2	18286016	18289149	7.607	LOC109195900	Ig kappa chain V region 4135-like	B	
NC_031983.2	30496957	30572334	7.908	myt1l	myelin transcription factor 1 like%2C	B	
NC_031983.2	30576884	30616320	7.908	pxdn	peroxidasin homolog	B	
NC_031983.2	30626945	30634063	7.908	LOC109195824	C-C chemokine receptor type 6	B	
NC_031984.2	36468693	36500844	9.803	LOC100695531	voltage-gated potassium channel subunit beta-2	B	
NC_031984.2	36595424	36657743	8.816	LOC100706982	ephrin type-B receptor 2	B	
NC_031984.2	36670657	36680808	8.816	LOC100707689	putative beta-lactamase-like 1	B	

NC_031984.2	36683225	36690992	8.816	LOC100707247	trafficking protein particle complex subunit 6B	B	
NC_031984.2	36707613	36718693	9.803	foxp3	forkhead box P3	B	
NC_031985.2	36097326	36098761	11.372	hsf1	heat shock factor protein 1	B	
NC_031985.2	36108249	36123669	10.422	wdyhv1	WDYHV motif containing 1	B	
NC_031985.2	36124482	36133950	10.422	bop1	block of proliferation 1	B	
NC_031985.2	36135378	36143267	10.422	lrp12	LDL receptor related protein 12	B	
NC_031985.2	36148714	36215398	10.422	LOC100693583	regulating synaptic membrane exocytosis protein 2	B	
NC_031985.2	36249984	36268986	10.422	LOC100694839	large proline-rich protein BAG6	B	
NC_031985.2	36270399	36278928	10.422	lg22h6orf136	linkage group 22 C6orf136 homolog%2C	B	
NC_031985.2	36283411	36300637	10.422	abcf1	ATP binding cassette subfamily F member 1	B	
NC_031985.2	36300633	36303325	10.422	mrps18b	28S ribosomal protein S18b%2C mitochondrial	B	
NC_031985.2	36303466	36314183	10.422	ppp1r10	serine/threonine-protein phosphatase 1 regulatory subunit 10	B	
NC_031985.2	36318174	36320292	10.422	agr2	anterior gradient 2%2C protein disulphide isomerase family member	B	
NC_031985.2	36322837	36335844	10.422	tspan13	tetraspanin 13	B	
NC_031967.2	33563988	33567997	7.448	LOC102078702	ankyrin repeat domain-containing protein 46-like	C	
NC_031967.2	33694728	33698069	7.448	LOC112843770	SCAN domain-containing protein 3-like	C	
NC_031967.2	33709945	33725339	7.448	LOC112845466	mucin-2-like	C	
NC_031967.2	33774596	33781082	7.448	LOC102078973	endonuclease domain-containing 1 protein-like	C	
NC_031967.2	36330485	36333895	7.470	LOC106098676	polymeric immunoglobulin receptor	C	
NC_031967.2	36371345	36372048	7.470	LOC100710332	histone H2B 1/2	C	
NC_031967.2	36373133	36374523	7.470	LOC109198952	histone H1-like	C	
NC_031967.2	36376344	36376827	7.470	LOC109198962	histone H2A	C	
NC_031967.2	36377072	36377582	7.470	LOC109198959	histone H3	C	
NC_031967.2	36382136	36383158	7.470	LOC109198966	histone H4	C	
NC_031967.2	36385021	36387414	7.470	LOC109198939	zinc finger BED domain-containing protein 1-like	C	AC
NC_031967.2	36440905	36451340	7.470	LOC109196860	zinc finger protein 883-like	C	
NC_031967.2	36502238	36511552	7.470	LOC109194461	zinc finger protein 665	C	
NC_031967.2	36555030	36570634	7.470	LOC102080278	gastrula zinc finger protein XlCGF26.1	C	
NC_031967.2	38541326	38574710	11.419	LOC100706439	kinesin-like protein KIF1C	C	ABC
NC_031967.2	38580689	38603752	11.419	LOC100705911	cysteinyl leukotriene receptor 1-like	C	ABC
NC_031967.2	38621206	38625268	11.419	LOC102075710	myelin-oligodendrocyte glycoprotein	C	ABC
NC_031967.2	38644422	38704304	11.419	LOC102076476	ankyrin repeat domain-containing protein 46	C	ABC
NC_031967.2	46859269	46890096	8.027	LOC102075779	interferon-induced protein 44	C	ABC
NC_031967.2	46940017	46945392	8.027	LOC109196641	C-type lectin BJcuL-like	C	ABC

NC_031967.2	47092303	47093821	8.027	LOC100690229	ladderlectin	C	ABC
NC_031967.2	52412778	52502241	7.754	LOC100700312	deleted in malignant brain tumors 1 protein	C	BC
NC_031967.2	52515236	52518245	7.722	LOC109202644	Fc receptor-like B	C	BC
NC_031967.2	52635907	52639339	7.722	LOC109199313	nuclear factor 7%2C ovary-like	C	BC
NC_031967.2	52648778	52653076	7.722	LOC112846437	hepatitis A virus cellular receptor 2 homolog	C	BC
NC_031967.2	52659901	52662778	7.754	LOC109199340	V-set and immunoglobulin domain-containing protein 1	C	
NC_031967.2	54159054	54202935	12.088	LOC106097159	low affinity immunoglobulin gamma Fc region receptor II	C	BC
NC_031967.2	54251264	54258518	12.088	LOC102081459	butyrophilin subfamily 2 member A2%2C	C	BC
NC_031967.2	54277348	54285214	12.088	LOC109196578	G2/M phase-specific E3 ubiquitin-protein ligase-like	C	BC
NC_031967.2	54365500	54366701	12.088	LOC112844103	putative nuclease HARBI1	C	BC
NC_031967.2	55308862	55313331	7.794	LOC100689971	butyrophilin subfamily 3 member A2	C	BC
NC_031967.2	55379911	55394599	7.794	LOC102079944	phospholipid transfer protein	C	BC
NC_031967.2	55428369	55430277	7.794	LOC102079855	BPI fold-containing family C protein-like	C	BC
NC_031967.2	62497031	62546070	8.401	LOC102077300	hemicentin-2	C	
NC_031967.2	87041325	87065594	7.509	LOC109197042	NLR family CARD domain-containing protein 3-like	C	ABC
NC_031967.2	87090875	87093871	7.509	LOC109201620	nesprin-2-like	C	ABC
NC_031967.2	87131491	87136076	7.509	LOC102078820	NLR family CARD domain-containing protein 3-like	C	
NC_031969.2	1102005	1111693	7.645	ube2z	ubiquitin conjugating enzyme E2 Z	C	
NC_031969.2	1140706	1145800	7.645	LOC100692587	ATP synthase F(0) complex subunit C2%2C mitochondrial	C	
NC_031969.2	1240172	1350997	7.645	LOC100701331	junction plakoglobin	C	
NC_031969.2	1314908	1317753	7.645	LOC109201795	polycystic kidney disease protein 1-like 3	C	
NC_031971.2	4813492	4822835	9.285	LOC100702690	E3 ubiquitin-protein ligase TRIM41	C	
NC_031971.2	4827387	4846869	9.285	LOC106096490	zinc finger protein 638	C	
NC_031974.2	7530734	7599969	8.620	LOC100707500	disco-interacting protein 2 homolog C	C	
NC_031974.2	7603048	7651893	7.424	LOC100707237	vasoactive intestinal polypeptide receptor 2	C	
NC_031974.2	7656264	7671129	7.424	LOC112847889	zinc finger protein 726-like	C	
NC_031974.2	7672915	7675091	7.424	LOC102080242	vegetative cell wall protein gp1	C	
NC_031974.2	7745546	7762222	7.424	LOC112847955	zinc finger protein 271-like	C	
NC_031974.2	7762268	7767304	7.424	LOC112847891	putative nuclease HARBI1	C	
NC_031975.2	5636743	5687786	7.674	LOC100703630	opioid-binding protein/cell adhesion molecule homolog	C	
NC_031975.2	5804258	5826632	7.674	LOC102082136	E3 ubiquitin-protein ligase CBL-B	C	
NC_031975.2	5830164	5879748	7.674	LOC100694738	CD166 antigen homolog	C	
NC_031978.2	33013032	33041036	7.418	LOC100696947	sodium/potassium/calcium exchanger 3	C	
NC_031978.2	33013032	33263032	7.418	ttc27	tetratricopeptide repeat domain 27%2C	C	

NC_031978.2	33225005	33242969	7.418	LOC100698617	pituitary homeobox 3	C	
NC_031982.2	32125312	32134587	8.448	LOC100710992	NLR family CARD domain-containing protein 3-like	C	
NC_031982.2	32145208	32169506	8.448	LOC102076273	NLR family CARD domain-containing protein 3	C	
NC_031982.2	32209102	32211167	8.448	LOC109195560	tripartite motif-containing protein 16-like	C	
NC_031982.2	32218177	32221426	8.448	LOC109195431	zinc finger protein 665-like	C	
NC_031982.2	32287341	32287501	8.448	LOC112843009	U1 spliceosomal RNA	C	
NC_031982.2	32287681	32287841	8.448	LOC112842998	U1 spliceosomal RNA	C	
NC_031982.2	32288953	32289124	8.448	LOC112843017	U1 spliceosomal RNA	C	
NC_031982.2	32289305	32289465	8.448	LOC112843008	U1 spliceosomal RNA	C	
NC_031982.2	32289647	32289808	8.448	LOC112842997	U1 spliceosomal RNA	C	
NC_031982.2	32290920	32291091	8.448	LOC112843012	U1 spliceosomal RNA	C	
NC_031982.2	32291272	32291433	8.448	LOC112843002	U1 spliceosomal RNA	C	
NC_031982.2	32291615	32291776	8.448	LOC112843007	U1 spliceosomal RNA	C	
NC_031982.2	32291958	32292121	8.448	LOC112843028	U1 spliceosomal RNA	C	
NC_031982.2	32293236	32293407	8.448	LOC112843018	U1 spliceosomal RNA	C	
NC_031982.2	32293588	32293751	8.448	LOC112843001	U1 spliceosomal RNA	C	
NC_031982.2	32293932	32294095	8.448	LOC112842990	U1 spliceosomal RNA	C	
NC_031982.2	32337832	32345694	8.448	znf622	zinc finger protein 622	C	
NC_031982.2	32352026	32365729	8.448	march11	E3 ubiquitin-protein ligase MARCH11	C	

LG: Linkage group
Initial pos: initial position
Final pos: final position
LG3: Genes shared between A, B and C strains in linkage group (LG)3
