# Supplementary material for: Whole genome re-sequencing reveals recent signatures of selection in three strains of farmed Nile tilapia (Oreochromis niloticus)
Source: Sci Rep. 2020 Jul 13;10:11514. doi: 10.1038/s41598-020-68064-5 (PMC7359307; doi:10.1038/s41598-020-68064-5)
Supplement: Supplementary file 6 — Supplementary table S4 [file 41598_2020_68064_MOESM6_ESM.pdf]

Supplementary information

Whole genome re-sequencing reveals recent signatures of selection in three strains of farmed Nile tilapia (*Oreochromis niloticus*)

María I. Cádiz[12], María E. López[31], Diego Díaz-Domínguez[4], Giovanna Cáceres[12], Grazyella M. Yoshida[1], Daniel Gomez-Uchida[5.6], José M. Yáñez[1,6]*.

[1] Facultad de Ciencias Veterinarias y Pecuarias, Universidad de Chile, Avenida Santa Rosa 11735, 8820808, La Pintana, Santiago, Chile

[2] Programa de Doctorado en Ciencias Silvoagropecuarias y Veterinarias, Campus Sur, Universidad de Chile, Santa Rosa 11315, La Pintana, Santiago, Chile. CP: 8820808.

[3] Department of Animal Breeding and Genetics, Swedish University of Agricultural Sciences, Uppsala, Sweden.

[4] Departamento de Ciencias de la Computación, Universidad de Chile.

[5] Facultad de Ciencias Naturales y Oceanográficas, Universidad de Concepción, Concepción, Chile.

[6] Núcleo Milenio INVASAL, Concepción, Chile

*jmayanez@uchile.cl +56-2 29785533 (Corresponding Author).

Supplementary Table S4 **.** List of all genes detected by the Rsb method in each strain (A, B and C).

LG	***Initial pos.***	***Final pos.***	***Rsb***	***Gene symbol***	***Gene name***	Strain
NC_031966.2	25187737	25191737	8.214	LOC102076834	hepatocyte cell adhesion molecule	A
NC_031966.2	25203024	25209031	8.214	LOC100695871	homeobox protein CDX-1	A
NC_031966.2	25214060	25242032	8.214	pdgfrb	platelet derived growth factor receptor beta	A
NC_031966.2	25242407	25254146	8.214	csf1r	colony stimulating factor 1 receptor%2C	A
NC_031966.2	25256867	25270532	8.214	hmgxb3	HMG domain-containing protein 3	A
NC_031966.2	25270906	25273231	8.214	LOC100695072	SLC35A4 upstream open reading frame protein	A
NC_031966.2	25273393	25274795	8.214	slc35a4	probable UDP-sugar transporter protein SLC35A4	A
NC_031966.2	25274305	25282954	8.214	LOC102077825	chromosome-associated kinesin KIF4	A
NC_031966.2	25285410	25290149	8.214	cd74	CD74 molecule	A
NC_031966.2	25292892	25338932	8.214	LOC100694812	bifunctional heparan sulfate N-deacetylase/N-sulfotransferase 1	A
NC_031966.2	25341163	25347045	8.214	rbm22	RNA binding motif protein 22	A
NC_031966.2	25346824	25353459	8.214	LOC100709366	myozenin-2	A
NC_031966.2	25365724	25369786	8.214	LOC100709097	ADP-ribosylation factor-like protein 3	A
NC_031966.2	25374659	25389771	8.214	LOC100694276	regulator of G-protein signaling 14	A
NC_031966.2	25397885	25414037	8.214	LOC100693999	sodium-dependent phosphate transport protein 2A	A
NC_031966.2	27352226	27356686	8.407	LOC109194874	trichohyalin	A
NC_031966.2	27359154	27362531	8.407	LOC112842384	spindle and centriole-associated protein 1-like	A
NC_031966.2	27375983	27392629	8.407	LOC109194891	glucosamine-6-phosphate isomerase 1	A
NC_031966.2	27446216	27532538	8.407	frmpd3	FERM and PDZ domain containing 3%2C	A
NC_031966.2	27543111	27546975	7.515	prps1	phosphoribosyl pyrophosphate synthetase 1%2C	A
NC_031966.2	27549757	27553073	7.515	slc25a53	solute carrier family 25 member 53	A
NC_031966.2	27561471	27571751	7.515	LOC100690556	SLAIN motif-containing protein-like	A
NC_031966.2	27572162	27578728	7.515	znf711	zinc finger protein 711	A
NC_031966.2	27584022	27587703	8.407	LOC106096447	BTB/POZ domain-containing protein KCTD12	A
NC_031966.2	27593581	27601295	7.515	LOC100690288	proline-rich receptor-like protein kinase PERK13	A
NC_031966.2	27640196	27643956	7.515	LOC100698667	rho-related GTP-binding protein RhoG	A
NC_031966.2	27649260	27659385	7.515	LOC102076457	FH2 domain-containing protein 1	A
NC_031966.2	27681739	27694605	7.515	ogt	O-linked N-acetylglucosamine (GlcNAc) transferase%2C	A
NC_031966.2	27695706	27705835	7.515	LOC100690020	acidic repeat-containing protein	A
NC_031966.2	27709051	27712606	7.515	LOC112841944	olfactory receptor 2T3-like	A
NC_031966.2	27712726	27715748	7.515	LOC100689752	caltractin	A
NC_031966.2	27717292	27721220	7.613	nsdhl	NAD(P) dependent steroid dehydrogenase-like	A

NC_031966.2	27742012	27748387	7.658	fut11	alpha-(1%2C3)-fucosyltransferase 11	A
NC_031966.2	27747535	27751837	7.658	LOC100697336	ras-related protein Rab-9B	A
NC_031966.2	27752465	27763025	7.658	LOC100697062	myelin proteolipid protein	A
NC_031966.2	27764513	27767799	7.658	LOC102077653	protocadherin-20	A
NC_031966.2	27768903	27772663	7.658	LOC100696796	regulator of cell cycle RGCC	A
NC_031966.2	27787345	27836396	8.088	nlgn3	neuroligin 3%2C	A
NC_031966.2	28028735	28116472	7.765	LOC100695922	glutamate receptor 3	A
NC_031966.2	28157911	28170303	7.765	eda	ectodysplasin A%2C	A
NC_031966.2	28170578	28173314	7.765	LOC100695394	tumor necrosis factor ligand superfamily member 13B	A
NC_031966.2	28173428	28176381	7.765	LOC100695129	leucine-rich repeat-containing protein 32	A
NC_031966.2	28184872	28188597	7.765	LOC102079731	gap junction beta-1 protein	A
NC_031966.2	28188961	28192214	7.765	LOC100694863	gap junction alpha-3 protein	A
NC_031966.2	28197602	28199689	7.765	vma21	VMA21%2C vacuolar ATPase assembly factor	A
NC_031966.2	28213049	28223814	7.765	LOC100694598	melatonin receptor type 1B	A
NC_031966.2	28231079	28259780	7.765	neurl1b	E3 ubiquitin-protein ligase NEURL1B	A
NC_031966.2	28269649	28271494	7.765	dusp1	dual specificity phosphatase 1	A
NC_031966.2	28397686	28400606	8.495	ddx41	DEAD-box helicase 41	A
NC_031966.2	28400739	28406143	8.495	dok3	docking protein 3	A
NC_031966.2	28406149	28407761	8.495	LOC100710497	neurogenin-2	A
NC_031966.2	28566252	28647686	8.495	unc5a	netrin receptor UNC5A	A
NC_031966.2	29450452	29462088	11.106	sncb	beta-synuclein	A
NC_031966.2	29464721	29469059	11.106	LOC109203106	neurofilament medium polypeptide	A
NC_031966.2	29468848	29482397	11.106	cdhr2	cadherin related family member 2%2C	A
NC_031966.2	29486392	29500530	11.106	rnf44	RING finger protein 44	A
NC_031966.2	29500708	29506930	11.106	faf2	FAS-associated factor 2	A
NC_031966.2	29507899	29511944	11.106	pin4	peptidyl-prolyl cis-trans isomerase NIMA-interacting 4	A
NC_031966.2	29511931	29527776	11.106	LOC106096426	serine/threonine-protein kinase Nek6	A
NC_031966.2	29531505	29537083	11.106	LOC100708081	D(1) dopamine receptor	A
NC_031966.2	29554104	29562025	11.106	LOC100710401	homeobox protein MSX-2	A
NC_031966.2	29572486	29583336	11.106	LOC100709874	cytoplasmic polyadenylation element-binding protein 4	A
NC_031966.2	29589118	29591455	9.164	stc2	stanniocalcin 2	A
NC_031966.2	30293447	30305593	7.984	LOC100704611	LON peptidase N-terminal domain and RING finger protein 1	A
NC_031966.2	30306198	30309397	7.984	med7	mediator complex subunit 7%2C	A
NC_031966.2	30311335	30318792	7.984	itk	IL2 inducible T cell kinase	A

NC_031966.2	30321628	30347186	7.984	LOC100704422	cytoplasmic FMR1-interacting protein 2	A
NC_031966.2	30351593	30359046	7.984	hmmr	hyaluronan mediated motility receptor	A
NC_031966.2	30359303	30361882	7.984	LOC100704156	insulin	A
NC_031966.2	30692287	30698315	7.754	LOC100703886	teneurin-2	A
NC_031966.2	30820508	30822699	10.046	LOC109194822	sentrin-specific protease 2-like	A
NC_031966.2	30918442	30918747	8.253	cystm1	cysteine rich transmembrane module containing 1	A
NC_031966.2	30931753	30935469	7.444	LOC100703341	eukaryotic translation initiation factor 4E-binding protein 3-like	A
NC_031966.2	30940462	30950025	7.444	acsl4	acyl-CoA synthetase long chain family member 4%2C	A
NC_031966.2	30950285	30953246	7.444	nxt2	NTF2-related export protein 2	A
NC_031966.2	30953383	30958772	7.444	psmd10	26S proteasome non-ATPase regulatory subunit 10	A
NC_031966.2	30960487	30965431	7.444	xiap	E3 ubiquitin-protein ligase XIAP	A
NC_031966.2	30966123	30988098	7.444	LOC100703806	cohesin subunit SA-2	A
NC_031966.2	31000108	31028571	7.584	LOC100703532	ephrin-B1	A
NC_031966.2	31092898	31102732	7.678	ara	androgen receptor alpha	A
NC_031966.2	31104024	31118427	7.678	LOC100702004	moesin	A
NC_031966.2	31117478	31127668	7.678	zc4h2	zinc finger C4H2 domain-containing protein	A
NC_031966.2	31129891	31133827	7.678	asb12	ankyrin repeat and SOCS box containing 12%2C	A
NC_031966.2	31133964	31164751	8.267	arhgef9	Cdc42 guanine nucleotide exchange factor 9%2C	A
NC_031966.2	31285060	31335764	7.757	LOC100702720	teneurin-1	A
NC_031966.2	32640755	32648430	7.743	fnta	farnesyltransferase%2C CAAX box%2C alpha	A
NC_031966.2	32657157	32659012	7.743	LOC102078310	transmembrane protein 271	A
NC_031966.2	32684943	32769091	7.743	pigg	GPI ethanolamine phosphate transferase 2	A
NC_031966.2	32771440	32786990	7.743	guf1	GUF1 homolog%2C GTPase%2C	A
NC_031966.2	32792340	32834293	7.743	gabra2	gamma-aminobutyric acid receptor subunit alpha-2	A
NC_031966.2	32839389	32871826	7.418	gabra4	gamma-aminobutyric acid receptor subunit alpha-4	A
NC_031966.2	32902824	32908859	7.418	cfap100	cilia and flagella associated protein 100%2C	A
NC_031966.2	32909882	32921694	7.418	ap1ar	AP-1 complex-associated regulatory protein	A
NC_031966.2	32925162	32935851	7.418	LOC100706145	mothers against decapentaplegic homolog 5	A
NC_031966.2	32936720	32946623	7.418	syvn1	E3 ubiquitin-protein ligase synoviolin	A
NC_031966.2	32947695	32953370	7.418	LOC100700492	integral membrane protein GPR137	A
NC_031966.2	32953412	32963565	7.418	LOC100700758	phospholipase D3	A
NC_031966.2	32963714	32967344	7.418	smim19	small integral membrane protein 19	A
NC_031966.2	32968573	32978683	7.418	fam199x	family with sequence similarity 199%2C X-linked%2C	A
NC_031966.2	32978738	32983334	7.418	commd5	COMM domain containing 5	A

NC_031966.2	32987366	33000318	7.418	lonrf3	LON peptidase N-terminal domain and RING finger protein 3	A
NC_031966.2	33060636	33063739	7.418	dcps	decapping enzyme%2C scavenger	A
NC_031966.2	33064849	33074938	7.418	ids	iduronate 2-sulfatase	A
NC_031966.2	33094108	33138305	7.908	aff2	AF4/FMR2 family member 2	A
NC_031966.2	33289858	33310450	7.830	fmr1	fragile X mental retardation 1	A
NC_031966.2	33312163	33326242	7.830	LOC100702116	ras-related protein Rab-33A	A
NC_031966.2	33328841	33336695	7.830	LOC100702380	matrix-remodeling-associated protein 5	A
NC_031966.2	33336667	33370892	7.830	LOC109195092	immunoglobulin superfamily member 10	A
NC_031966.2	33373168	33378628	7.830	hdac3	histone deacetylase 3	A
NC_031966.2	33379155	33417372	7.830	myot	myotilin	A
NC_031966.2	33422513	33480144	7.849	LOC100703189	TRAF2 and NCK-interacting protein kinase	A
NC_031966.2	33495597	33501363	7.849	LOC100708648	beta-1%2C3-galactosyltransferase 2	A
NC_031967.2	10036077	10049870	7.467	rcor2	REST corepressor 2	A
NC_031967.2	10064092	10073644	7.467	naa40	N(alpha)-acetyltransferase 40%2C NatD catalytic subunit%2C	A
NC_031967.2	10074318	10079462	7.467	b3gat3	beta-1%2C3-glucuronyltransferase 3	A
NC_031967.2	10095237	10095301	7.467	LOC112846588	small nucleolar RNA SNORD29	A
NC_031967.2	10095501	10095571	7.467	LOC112846589	small nucleolar RNA SNORD30	A
NC_031967.2	10095815	10095940	7.467	LOC112846592	small nucleolar RNA SNORD22	A
NC_031967.2	10096716	10096787	7.467	LOC112846590	small nucleolar RNA SNORD31	A
NC_031967.2	10096989	10097060	7.467	LOC112846591	small nucleolar RNA SNORD31	A
NC_031967.2	10097672	10100033	7.467	arl2	ADP ribosylation factor like GTPase 2	A
NC_031967.2	10102569	10112763	7.467	LOC102075936	putative protein TPRXL	A
NC_031967.2	10115823	10165986	7.467	ppp2r5b	protein phosphatase 2 regulatory subunit B'beta	A
NC_031967.2	10169443	10172560	7.467	LOC100707302	glycoprotein hormone beta-5	A
NC_031967.2	10178786	10183263	7.467	gpha2	glycoprotein hormone alpha 2	A
NC_031967.2	10182817	10194753	7.467	majin	membrane anchored junction protein%2C	A
NC_031967.2	10194876	10197402	7.467	LOC100712401	glyoxal reductase%2C	A
NC_031967.2	10197446	10202008	7.467	bad	BCL2 associated agonist of cell death	A
NC_031967.2	15950514	15964402	10.479	LOC100702854	calpain-5	A
NC_031967.2	15965295	15968805	10.479	cnpy4	canopy FGF signaling regulator 4	A
NC_031967.2	15977632	15997578	10.479	LOC100703122	netrin-1	A
NC_031967.2	16001948	16021471	10.479	LOC102077238	active breakpoint cluster region-related protein	A
NC_031967.2	16030678	16042411	10.479	LOC106097031	cornifelin homolog B	A
NC_031967.2	16044950	16057787	10.479	LOC109200085	cornifelin homolog B-like	A

NC_031967.2	16129814	16134352	10.479	LOC100700423	cornifelin homolog B%2C	A
NC_031967.2	16138745	16145420	10.479	LOC106097030	cornifelin homolog B-like	A
NC_031967.2	46859269	46890096	11.230	LOC102075779	interferon-induced protein 44	A
NC_031967.2	46940017	46945392	11.230	LOC109196641	C-type lectin BJcuL-like	A
NC_031967.2	47092303	47093821	11.230	LOC100690229	ladderlectin	A
NC_031967.2	52412950	52502241	8.853	LOC100700312	deleted in malignant brain tumors 1 protein	A
NC_031967.2	52515236	52518245	8.853	LOC109202644	Fc receptor-like B	A
NC_031967.2	52635907	52639339	8.853	LOC109199313	nuclear factor 7%2C ovary-like	A
NC_031967.2	52648778	52653076	8.853	LOC112846437	hepatitis A virus cellular receptor 2 homolog	A
NC_031967.2	52659901	52662950	8.853	LOC109199340	V-set and immunoglobulin domain-containing protein 1	A
NC_031967.2	53707615	53711900	7.843	LOC109201334	zinc finger MYM-type protein 1	A
NC_031967.2	53808345	53810754	7.843	LOC109201337	tripartite motif-containing protein 16-like	A
NC_031967.2	53866123	53874374	7.843	LOC102082344	retrovirus-related Pol polyprotein from transposon 412%2C	A
NC_031967.2	53877225	53949317	7.843	LOC109201351	low affinity immunoglobulin gamma Fc region receptor II	A
NC_031967.2	53905553	53920952	7.843	LOC112845985	zinc finger protein 883-like	A
NC_031967.2	53915858	53931177	7.843	LOC109201348	low affinity immunoglobulin gamma Fc region receptor II	A
NC_031967.2	54159054	54202935	14.955	LOC106097159	low affinity immunoglobulin gamma Fc region receptor II	A
NC_031967.2	54251264	54258518	14.955	LOC102081459	butyrophilin subfamily 2 member A2%2C	A
NC_031967.2	54277348	54285214	14.955	LOC109196578	G2/M phase-specific E3 ubiquitin-protein ligase-like	A
NC_031967.2	54365500	54366701	14.955	LOC112844103	putative nuclease HARBI1	A
NC_031967.2	55308862	55313331	11.385	LOC100689971	butyrophilin subfamily 3 member A2	A
NC_031967.2	55379911	55394599	11.385	LOC102079944	phospholipid transfer protein	A
NC_031967.2	55428369	55430194	11.385	LOC102079855	BPI fold-containing family C protein-like	A
NC_031967.2	61070126	61320126	8.261	LOC100703578	E3 ubiquitin-protein ligase TRIM39	A
NC_031967.2	765110	846479	7.476	LOC100696152	ankyrin-2	A
NC_031967.2	78818818	78838523	7.427	LOC109201126	beta-1%2C4 N-acetylgalactosaminyltransferase 2-like	A
NC_031967.2	78914120	78917532	7.427	LOC112841649	programmed cell death 1 ligand 1-like	A
NC_031967.2	849299	945570	7.476	LOC100695450	calcium/calmodulin-dependent protein kinase type II delta 1 chain	A
NC_031967.2	851067	892890	7.476	LOC112846512	calcium/calmodulin-dependent protein kinase type II delta chain-like	A
NC_031967.2	9957292	9965075	7.467	hspa12b	heat shock 70 kDa protein 12B	A
NC_031967.2	9965197	9970564	7.467	cct7	T-complex protein 1 subunit eta	A
NC_031967.2	9971902	9978896	7.467	LOC100708373	RING finger protein B	A
NC_031967.2	9983989	10034340	7.467	mark2	microtubule affinity regulating kinase 2%2C	A
NC_031969.2	17151548	17157394	7.785	LOC100701484	zona pellucida sperm-binding protein 3	A

NC_031969.2	17163313	17212064	7.633	LOC100701757	trinucleotide repeat-containing gene 6C protein	A
NC_031969.2	17249854	17328680	7.633	LOC100702031	neuronal-specific septin-3	A
NC_031969.2	17337180	17355805	7.633	LOC106098794	T-cell antigen CD7	A
NC_031969.2	17345598	17350030	7.633	LOC109201947	T-cell antigen CD7	A
NC_031969.2	17359397	17368721	7.633	jpt1	Jupiter microtubule associated homolog 1	A
NC_031969.2	17370813	17376701	7.633	sumo2	small ubiquitin-like modifier 2	A
NC_031969.2	17376843	17383686	7.633	nup85	nuclear pore complex protein Nup85	A
NC_031969.2	17383743	17394683	7.633	gga3	ADP-ribosylation factor-binding protein GGA3	A
NC_031969.2	17393822	17397185	7.633	mrps7	28S ribosomal protein S7%2C mitochondrial	A
NC_031969.2	17397278	17401548	7.785	mif4gd	MIF4G domain containing	A
NC_031969.2	20984951	21015585	7.543	rnf213	E3 ubiquitin-protein ligase RNF213	A
NC_031969.2	21016259	21020569	7.543	LOC109201825	B-cell receptor CD22-like	A
NC_031969.2	21021725	21025329	7.543	LOC102082641	B-cell receptor CD22-like	A
NC_031969.2	21027137	21028448	7.543	LOC100700387	neuropeptide B	A
NC_031969.2	21028672	21039645	7.543	LOC100700658	guanine nucleotide-binding protein G(o) subunit alpha	A
NC_031969.2	21040266	21050865	7.543	LOC100707635	ethanolamine-phosphate cytidylyltransferase	A
NC_031969.2	21053035	21068413	7.543	LOC100700923	transcription factor MafG	A
NC_031969.2	21068593	21071896	7.543	LOC100701200	pyrroline-5-carboxylate reductase 1%2C mitochondrial	A
NC_031969.2	21073387	21075355	7.543	LOC100707908	myeloid-associated differentiation marker-like protein 2	A
NC_031969.2	21077278	21084416	7.543	LOC100708180	notum%2C palmitoleoyl-protein carboxylesterase	A
NC_031970.2	2018833	2025568	8.197	LOC109201209	C-type lectin lectoxin-Lio3-like	A
NC_031970.2	2091314	2103114	8.197	nicn1	nicolin 1	A
NC_031970.2	2173449	2177759	8.197	LOC109196940	E3 ubiquitin-protein ligase TRIM21-like	A
NC_031970.2	4702891	4738828	7.538	LOC100703093	angiopoietin-4	A
NC_031970.2	4764244	4769887	10.007	LOC102080461	coxsackievirus and adenovirus receptor homolog	A
NC_031970.2	4786930	4793877	10.007	LOC102080365	polymeric immunoglobulin receptor-like	A
NC_031970.2	4847268	4866754	10.007	LOC100694792	cullin-associated NEDD8-dissociated protein 1	A
NC_031970.2	4868840	4878343	10.007	sec13	SEC13 homolog%2C nuclear pore and COPII coat complex component	A
NC_031970.2	4879605	4894911	10.007	LOC109194281	deoxyribonuclease-1	A
NC_031970.2	4914008	4915485	10.007	LOC100702560	granzyme B	A
NC_031970.2	4931470	4945346	10.007	LOC100702290	deoxyribonuclease-1	A
NC_031970.2	5002826	5061770	10.260	LOC100701753	granzyme B-like	A
NC_031970.2	5099114	5100702	10.260	LOC109194506	anionic trypsin-1-like	A
NC_031970.2	5114496	5119647	10.260	LOC102076094	deoxyribonuclease-1-like	A

NC_031970.2	5121186	5129749	10.260	LOC100700928	deoxyribonuclease-1	A
NC_031970.2	5131125	5166160	10.260	LOC109194541	deoxyribonuclease-1	A
NC_031970.2	5142734	5144527	10.260	LOC100701205	granzyme B(G%2CH)	A
NC_031970.2	5166548	5168054	10.260	LOC106098728	granzyme B-like	A
NC_031970.2	5180256	5181654	10.260	LOC100534529	granzyme-1	A
NC_031970.2	5185253	5194810	10.260	LOC100700119	keratin%2C type II cytoskeletal 8	A
NC_031970.2	5196203	5214460	10.260	rbl1	RB transcriptional corepressor like 1	A
NC_031970.2	5217848	5228706	10.260	LOC100699587	transmembrane protein 74B	A
NC_031970.2	5242815	5255349	12.403	psmf1	proteasome inhibitor PI31 subunit	A
NC_031970.2	5256940	5261549	7.938	pigu	phosphatidylinositol glycan anchor biosynthesis class U	A
NC_031970.2	5269816	5271363	7.412	LOC100699316	tripartite motif-containing protein 35-like	A
NC_031970.2	5275154	5278246	7.412	LOC100699045	zinc-binding protein A33-like	A
NC_031971.2	19002433	19036451	7.603	rnf24	RING finger protein 24	A
NC_031971.2	19041863	19043364	7.434	ccdc86	coiled-coil domain containing 86%2C	A
NC_031971.2	19045941	19055914	7.434	glb1	beta-galactosidase	A
NC_031971.2	19059072	19063106	7.434	LOC100697970	ecto-ADP-ribosyltransferase 5	A
NC_031971.2	19065274	19067991	7.434	LOC102076348	ecto-ADP-ribosyltransferase 5%2C	A
NC_031971.2	19068368	19085110	7.434	LOC100698242	ecto-ADP-ribosyltransferase 4	A
NC_031971.2	19087017	19091634	7.434	LOC102075943	ecto-ADP-ribosyltransferase 4	A
NC_031971.2	19093623	19098438	7.434	LOC100709264	claudin domain-containing protein 1	A
NC_031971.2	19099229	19106317	7.434	LOC100698506	N-acyl-aromatic-L-amino acid amidohydrolase (carboxylate-forming) B	A
NC_031971.2	19112139	19117047	7.421	tacr3b	neuromedin-K receptor-like	A
NC_031971.2	19119421	19121908	7.421	LOC100709532	ecto-ADP-ribosyltransferase 4	A
NC_031971.2	19123000	19128461	7.421	primpol	DNA-directed primase/polymerase protein	A
NC_031971.2	19128542	19147977	7.421	LOC100709803	long-chain-fatty-acid--CoA ligase 1	A
NC_031971.2	19153935	19156329	7.421	helt	hairy and enhancer of split-related protein HELT	A
NC_031971.2	19182706	19184955	7.421	slc25a4	ADP/ATP translocase 1	A
NC_031971.2	19185343	19194057	7.421	cfap97	cilia and flagella associated protein 97%2C	A
NC_031971.2	19195822	19201851	7.421	ufsp2	UFM1 specific peptidase 2%2C	A
NC_031971.2	19210632	19221243	7.421	gucy1a1	guanylate cyclase 1 soluble subunit alpha 1	A
NC_031971.2	19222091	19241452	7.421	gucy1b1	guanylate cyclase 1 soluble subunit beta 1	A
NC_031971.2	19245641	19246781	7.421	fabp2	fatty acid binding protein 2	A
NC_031971.2	19246879	19251788	7.603	LOC100699853	inactive ubiquitin carboxyl-terminal hydrolase 53	A
NC_031971.2	19276647	19280883	10.043	LOC100711322	myozenin-2	A

NC_031971.2	19281766	19291401	10.043	LOC102079281	synaptopodin-2	A
NC_031971.2	19298783	19303338	10.043	LOC100700121	solute carrier family 22 member 7	A
NC_031971.2	19303405	19314624	10.043	LOC100700394	solute carrier family 22 member 7	A
NC_031971.2	19316471	19321073	10.043	LOC102078086	solute carrier family 22 member 7	A
NC_031971.2	19321323	19326566	10.043	LOC109202510	pre-mRNA-splicing factor ATP-dependent RNA helicase DHX15	A
NC_031971.2	19327076	19328073	10.043	LOC109202456	solute carrier family 22 member 7-like	A
NC_031971.2	19329911	19335004	10.043	LOC106098904	solute carrier family 22 member 7	A
NC_031971.2	19335142	19345204	10.043	LOC100706682	pre-mRNA-splicing factor ATP-dependent RNA helicase DHX15	A
NC_031971.2	19346822	19348350	10.043	sod3	extracellular superoxide dismutase [Cu-Zn]	A
NC_031971.2	19347872	19357850	10.043	LOC100691566	coiled-coil domain-containing protein 149	A
NC_031971.2	19358931	19359699	7.421	LOC100691835	leucine-rich repeat LGI family member 2	A
NC_031971.2	19393293	19407595	10.043	mttp	microsomal triglyceride transfer protein	A
NC_031971.2	19413256	19424483	10.043	LOC100707215	tetraspanin-5	A
NC_031971.2	19426821	19442596	10.043	acer2	alkaline ceramidase 2	A
NC_031971.2	19444583	19449023	10.043	rps6	40S ribosomal protein S6	A
NC_031971.2	19454131	19456861	10.043	LOC100692918	perilipin-2	A
NC_031971.2	19456977	19470318	10.043	LOC100707927	WD40 repeat-containing protein SMU1	A
NC_031971.2	19470146	19478753	10.043	LOC100708200	dnaJ homolog subfamily A member 1	A
NC_031971.2	19478657	19482546	10.043	aptx	aprataxin	A
NC_031971.2	19484825	19487834	10.043	LOC100693190	dentin sialophosphoprotein	A
NC_031971.2	19491147	19498409	10.043	LOC100693466	SPARC-like protein 1	A
NC_031971.2	19503630	19506471	10.043	LOC100693995	class E vacuolar protein-sorting machinery protein HSE1-like	A
NC_031971.2	19614181	19615065	9.136	LOC109202643	spore coat protein SP96-like	A
NC_031971.2	19620281	19621195	9.136	LOC102079162	spore coat protein SP65-like	A
NC_031971.2	19660606	19664058	9.136	LOC109202454	proline-rich protein LAS17-like	A
NC_031971.2	20260717	20262821	7.580	LOC106098902	zinc finger BED domain-containing protein 4	A
NC_031971.2	20352880	20447114	7.580	ctnna2	catenin alpha 2%2C	A
NC_031971.2	20474025	20478490	7.498	lrrtm1	leucine rich repeat transmembrane neuronal 1	A
NC_031971.2	20785493	20795315	10.245	adra1d	adrenoceptor alpha 1D%2C	A
NC_031971.2	20795490	20806055	10.245	LOC100695334	5-hydroxytryptamine receptor 4-like	A
NC_031971.2	20844300	20858425	10.245	mettl14	N6-adenosine-methyltransferase non-catalytic subunit	A
NC_031971.2	20859537	20873159	9.080	prss12	neurotrypsin	A
NC_031971.2	20884433	20910356	7.454	LOC100695867	bifunctional heparan sulfate N-deacetylase/N-sulfotransferase 4	A
NC_031971.2	21015585	21041096	7.742	ugt8	2-hydroxyacylsphingosine 1-beta-galactosyltransferase	A

NC_031971.2	21051108	21067997	7.536	spock3	SPARC (osteonectin)%2C cwcv and kazal like domains proteoglycan 3%2C	A
NC_031971.2	21073685	21084057	7.449	LOC100689789	choline transporter-like protein 1	A
NC_031971.2	21093704	21100540	8.282	LOC100696392	rho GTPase-activating protein 7	A
NC_031971.2	21186101	21198674	10.023	lonrf1	LON peptidase N-terminal domain and RING finger protein 1	A
NC_031971.2	21197768	21227528	9.369	LOC100696923	calcium-binding protein 2	A
NC_031971.2	21227195	21227528	9.369	LOC100690058	double C2-like domain-containing protein beta	A
NC_031971.2	21282453	21287967	10.023	serping1	C1 inhibitor precursor	A
NC_031971.2	21290835	21294476	9.485	tmem134	transmembrane protein 134	A
NC_031971.2	21302754	21307181	10.023	aip	AH receptor-interacting protein	A
NC_031971.2	21307514	21312310	10.023	cdk2ap2	cyclin dependent kinase 2 associated protein 2	A
NC_031971.2	21342210	21345677	10.023	LOC109202440	serine/threonine-protein kinase pim-1	A
NC_031971.2	21355114	21358601	10.023	LOC109202441	serine/threonine-protein kinase pim-2-like	A
NC_031971.2	21365778	21368852	10.023	LOC109202442	serine/threonine-protein kinase pim-1-like	A
NC_031971.2	21376425	21381586	7.950	LOC106098749	serine/threonine-protein kinase pim-1	A
NC_031971.2	21385836	21385945	8.551	LOC102080289	serine/threonine-protein kinase pim-1	A
NC_031971.2	21394596	21430135	10.637	gpr83	G protein-coupled receptor 83	A
NC_031971.2	21442605	21452098	10.637	ankrd49	ankyrin repeat domain 49%2C	A
NC_031971.2	21481064	21503762	10.637	LOC109194342	nucleoporin NUP159	A
NC_031971.2	21530812	21538855	10.637	LOC102079525	E3 ubiquitin-protein ligase ZFP91	A
NC_031971.2	21542912	21543908	8.286	rbm47	RNA-binding protein 47	A
NC_031971.2	21594744	21615501	7.551	LOC102081304	putative methyltransferase NSUN7	A
NC_031971.2	21616038	21671667	7.551	apbb2	amyloid beta precursor protein binding family B member 2%2C	A
NC_031971.2	21624239	21627762	7.551	LOC102078215	trichohyalin-like	A
NC_031971.2	21674917	21682757	7.636	haspin	histone H3 associated protein kinase%2C	A
NC_031971.2	21687943	21721454	7.551	LOC100695321	protein FAM184B	A
NC_031971.2	21723740	21728416	8.551	ncapg	condensin complex subunit 3	A
NC_031971.2	21742358	21746333	7.551	lcorl	ligand dependent nuclear receptor corepressor like%2C	A
NC_031971.2	21883711	21982582	7.454	slit2	slit homolog 2 protein	A
NC_031971.2	21983356	21995190	7.454	LOC100694526	intelectin	A
NC_031971.2	21997670	22003957	7.454	LOC100693983	intelectin	A
NC_031971.2	22010686	22013722	7.454	LOC100693716	D(1A) dopamine receptor-like	A
NC_031971.2	22021056	22029675	7.454	LOC100693455	neuronal acetylcholine receptor subunit alpha-9-I	A
NC_031971.2	22032577	22038104	7.454	rhoh	ras homolog family member H	A
NC_031971.2	22040317	22049251	9.867	n4bp2	NEDD4 binding protein 2%2C	A

NC_031971.2	22098822	22102175	7.797	smim14	small integral membrane protein 14	A
NC_031971.2	22102343	22110003	7.403	map9	microtubule associated protein 9%2C	A
NC_031971.2	22113114	22120147	7.403	ugdh	UDP-glucose 6-dehydrogenase	A
NC_031971.2	22120200	22126829	7.403	lias	lipoic acid synthetase	A
NC_031971.2	22127440	22133221	7.403	rpl9	60S ribosomal protein L9	A
NC_031971.2	22134879	22145004	7.403	rfc1	replication factor C subunit 1	A
NC_031971.2	22146022	22161013	7.403	wdr19	WD repeat domain 19%2C	A
NC_031971.2	22165227	22227871	7.403	klhl5	kelch like family member 5%2C	A
NC_031971.2	22236220	22248803	7.403	fam114a1	family with sequence similarity 114 member A1	A
NC_031971.2	22253504	22260203	7.403	klf3	Krueppel-like factor 3	A
NC_031971.2	22300061	22346230	7.403	LOC100690481	TBC1 domain family member 1	A
NC_031971.2	22346708	22348822	7.797	pgm2	phosphoglucomutase 2%2C	A
NC_031971.2	22360647	22376848	7.445	zcchc7	zinc finger CCHC domain-containing protein 7	A
NC_031971.2	22422003	22424941	7.838	pax5	paired box 5%2C	A
NC_031971.2	22488720	22493142	8.437	melk	maternal embryonic leucine zipper kinase	A
NC_031971.2	22502804	22513722	7.763	LOC100712566	thrombospondin type-1 domain-containing protein 4	A
NC_031971.2	22514352	22523619	7.763	LOC100712301	tyrosine-protein phosphatase non-receptor type 9	A
NC_031971.2	22525221	22543431	7.763	polq	DNA polymerase theta	A
NC_031971.2	22542008	22553813	7.763	uba6	ubiquitin like modifier activating enzyme 6%2C	A
NC_031971.2	22554928	22557844	7.763	grhpr	glyoxylate and hydroxypyruvate reductase	A
NC_031971.2	22557630	22567549	7.763	zbtb5	zinc finger and BTB domain containing 5%2C	A
NC_031971.2	22567748	22572723	7.763	polr1e	DNA-directed RNA polymerase I subunit RPA49	A
NC_031971.2	22572904	22579825	7.763	fbxo10	F-box only protein 10	A
NC_031971.2	22593677	22596051	7.763	LOC100698610	C-X-C motif chemokine 10	A
NC_031971.2	22602022	22603115	7.763	il-8	interleukin-8	A
NC_031971.2	22604553	22605565	7.763	LOC109202588	growth-regulated protein homolog gamma	A
NC_031971.2	22608356	22614513	9.344	LOC100698346	calcium/calmodulin-dependent protein kinase type II delta 2 chain	A
NC_031971.2	22651272	22688404	7.904	LOC100710955	ankyrin-2	A
NC_031971.2	24188613	24212012	7.596	LOC100709169	glutamate receptor ionotropic%2C NMDA 2B	A
NC_031971.2	24230131	24261245	7.596	atf7ip	activating transcription factor 7 interacting protein%2C	A
NC_031971.2	24282917	24300857	7.570	sept3	neuronal-specific septin-3	A
NC_031971.2	24334134	24345354	7.570	LOC100710246	serine hydrolase-like protein	A
NC_031971.2	24348658	24350765	7.570	Mb	myoglobin	A
NC_031971.2	24358666	24365289	7.570	cenpm	centromere protein M	A

NC_031971.2	24367756	24375620	7.570	LOC100697878	putative RNA-binding protein Luc7-like 2	A
NC_031971.2	24377419	24379108	7.570	LOC100698144	essential MCU regulator%2C mitochondrial	A
NC_031971.2	24378956	24394583	7.570	LOC102077678	TRIO and F-actin-binding protein	A
NC_031971.2	24406331	24418211	7.570	LOC100710503	inward rectifier potassium channel 4	A
NC_031971.2	24430916	24435108	12.100	LOC100698408	transcription factor Sox-10	A
NC_031971.2	24438662	24447292	12.100	rnaseh2a	ribonuclease H2 subunit A	A
NC_031971.2	24462553	24505169	7.570	LOC100710771	microtubule-associated serine/threonine-protein kinase 1	A
NC_031971.2	24543235	24552095	12.100	atg4d	autophagy related 4D cysteine peptidase	A
NC_031971.2	24553009	24570028	12.100	LOC100711303	transcription activator BRG1	A
NC_031971.2	24578041	24601879	12.100	LOC100711575	low-density lipoprotein receptor	A
NC_031971.2	24611231	24619111	12.100	LOC100698945	AP-1 complex subunit mu-2	A
NC_031971.2	24619458	24622098	12.100	cdkn2d	cyclin dependent kinase inhibitor 2D	A
NC_031971.2	24626371	24634220	12.100	LOC100699482	volume-regulated anion channel subunit LRRC8C	A
NC_031971.2	24635742	24650368	12.100	keap1	kelch like ECH associated protein 1	A
NC_031971.2	24653604	24656929	12.100	LOC100712110	sphingosine 1-phosphate receptor 1	A
NC_031971.2	24690091	24690167	7.423	fdx2	ferredoxin 2	A
NC_031971.2	24693173	24693608	8.679	zglp1	GATA-type zinc finger protein 1	A
NC_031971.2	24925044	24976922	7.667	LOC100690838	nuclear factor 1 X-type	A
NC_031971.2	24996867	24999656	10.389	LOC100691100	DAN domain family member 5-like	A
NC_031971.2	25004691	25009822	10.389	LOC100701102	tetraspanin-1	A
NC_031971.2	25011100	25020962	10.389	LOC100701373	ras-related protein Rab-3D	A
NC_031971.2	25022274	25028141	10.389	pld6	mitochondrial cardiolipin hydrolase	A
NC_031971.2	25027641	25031415	10.389	LOC100701647	transcription elongation factor 1 homolog	A
NC_031971.2	25046092	25109647	10.389	LOC100691640	phospholipid phosphatase-related protein type 5	A
NC_031971.2	25109735	25143359	10.389	LOC102077827	paralemmin-1	A
NC_031972.2	20068875	20103548	8.048	pappa	pappalysin 1%2C	A
NC_031972.2	20122177	20166676	8.048	LOC100697547	zinc finger protein 618	A
NC_031972.2	20206975	20318875	8.048	rgs3	regulator of G protein signaling 3%2C	A
NC_031974.2	1231013	1232111	7.692	LOC109196898	zinc finger protein 883-like	A
NC_031974.2	1245558	1247642	7.692	LOC112847924	zinc finger protein 239-like	A
NC_031974.2	1255768	1279566	7.692	LOC112847859	zinc finger protein 665-like	A
NC_031974.2	1358045	1363779	7.692	LOC102083260	zinc finger protein 708-like	A
NC_031974.2	1448802	1481013	7.692	LOC102080698	zinc finger protein 100	A
NC_031974.2	18322555	18365708	10.274	vipr1	vasoactive intestinal peptide receptor 1	A

NC_031974.2	18401604	18413372	10.274	LOC100709670	eukaryotic translation initiation factor 5B	A
NC_031974.2	18425120	18426275	10.274	LOC100708859	nanos homolog 1-like	A
NC_031974.2	18427405	18434877	10.274	kpna1	importin subunit alpha-5	A
NC_031974.2	18439336	18442908	10.274	LOC100698117	protein FAM162B	A
NC_031974.2	18443169	18447831	10.274	ccdc58	coiled-coil domain containing 58%2C	A
NC_031974.2	18451568	18452409	10.274	csta	cystatin A	A
NC_031974.2	18456939	18457778	10.274	LOC100697579	cystatin-B-like	A
NC_031974.2	18472303	18474121	10.274	LOC100697316	cystatin-B-like	A
NC_031974.2	18489822	18492141	10.274	LOC109203484	trichohyalin-like	A
NC_031974.2	18493855	18497890	10.274	LOC109203569	extracellular matrix protein FRAS1	A
NC_031974.2	18497803	18500585	10.274	LOC106097837	cystatin-B	A
NC_031974.2	18501318	18503115	10.274	LOC100690818	cystatin-B	A
NC_031974.2	18503258	18511279	10.274	LOC109203571	cystatin-B	A
NC_031974.2	18512185	18515908	10.274	LOC109203572	cystatin-B	A
NC_031974.2	18519150	18522221	10.274	LOC109203567	trichohyalin-like	A
NC_031974.2	18522878	18572555	10.274	LOC102082737	extracellular matrix protein FRAS1	A
NC_031974.2	18532113	18535014	10.274	LOC100710659	cystatin-B	A
NC_031974.2	18541778	18543040	10.274	LOC100695024	cystatin-B	A
NC_031974.2	18557922	18563668	10.274	LOC100696082	thiosulfate:glutathione sulfurtransferase	A
NC_031974.2	7526989	7599969	7.455	LOC100707500	disco-interacting protein 2 homolog C	A
NC_031974.2	7603048	7651893	10.172	LOC100707237	vasoactive intestinal polypeptide receptor 2	A
NC_031974.2	7656264	7671129	10.172	LOC112847889	zinc finger protein 726-like	A
NC_031974.2	7672915	7675091	10.172	LOC102080242	vegetative cell wall protein gp1	A
NC_031974.2	7745546	7762222	10.172	LOC112847955	zinc finger protein 271-like	A
NC_031974.2	7762268	7767304	10.172	LOC112847891	putative nuclease HARBI1	A
NC_031977.2	36455896	36473897	9.060	LOC102079812	GTPase IMAP family member 2	A
NC_031977.2	36470115	36472183	9.060	LOC106097401	zinc finger MYM-type protein 1	A
NC_031977.2	36488180	36489356	9.060	LOC100695451	poly [ADP-ribose] polymerase 14	A
NC_031977.2	36494296	36506319	9.060	LOC100695189	poly [ADP-ribose] polymerase 14	A
NC_031977.2	36516610	36526494	9.060	LOC100694918	GTPase IMAP family member 8-like	A
NC_031977.2	36542175	36566833	9.060	LOC100694656	poly [ADP-ribose] polymerase 14	A
NC_031977.2	36591695	36602072	9.060	LOC102080448	GTPase IMAP family member 8	A
NC_031977.2	36620684	36639479	9.060	LOC100694118	GTPase IMAP family member 8	A
NC_031977.2	36643253	36678724	9.060	LOC109204452	zinc finger BED domain-containing protein 4-like	A

NC_031978.2	1007340	1015747	10.015	LOC100705715	cytochrome P450 26B1	A
NC_031978.2	1077872	1101992	10.015	LOC109194243	TBC1 domain family member 12	A
NC_031978.2	1121179	1121281	9.388	LOC102077403	CD48 antigen	A
NC_031978.2	1135087	1135273	10.034	LOC102077498	pregnancy-specific beta-1-glycoprotein 8-like	A
NC_031978.2	1141810	1146333	7.545	LOC100704647	solute carrier family 22 member 15-like	A
NC_031978.2	11521348	11560700	7.637	LOC100691860	RAC-gamma serine/threonine-protein kinase	A
NC_031978.2	1158710	1160847	10.015	LOC112841916	titin-like	A
NC_031978.2	11587395	11597757	7.637	zbtb18	zinc finger and BTB domain containing 18%2C	A
NC_031978.2	11619668	11651415	7.637	LOC100691320	adenylosuccinate synthetase isozyme 2	A
NC_031978.2	11654659	11658031	7.637	LOC102080800	zinc finger protein 37	A
NC_031978.2	1165526	1165964	7.720	kif11	kinesin family member 11%2C	A
NC_031978.2	11665010	11708881	7.637	LOC100691053	equilibrative nucleoside transporter 1	A
NC_031978.2	11710988	11731269	7.637	LOC100690786	atlastin-2	A
NC_031978.2	1193799	1194284	8.562	LOC100712147	early growth response protein 2b	A
NC_031978.2	1198497	1199761	8.812	LOC100711878	2-aminoethanethiol dioxygenase	A
NC_031978.2	1218517	1222226	8.820	LOC100708019	protein ZNF365	A
NC_031978.2	1230301	1237709	10.084	sult6b1	sulfotransferase 6B1	A
NC_031978.2	1249298	1279834	8.480	slc30a6	solute carrier family 30 member 6	A
NC_031978.2	1388906	1389038	7.402	LOC100711336	forkhead box protein G1	A
NC_031978.2	1420854	1421137	10.152	LOC100710803	acylphosphatase-2	A
NC_031978.2	1432790	1433603	10.357	LOC100711069	spectrin beta chain%2C non-erythrocytic 1	A
NC_031978.2	152040	175856	7.794	LOC100691573	bone morphogenetic protein receptor type-1A	A
NC_031978.2	1538155	1543259	8.522	LOC100709102	echinoderm microtubule-associated protein-like 6	A
NC_031978.2	1624658	1650911	7.612	LOC100710178	reticulon-1	A
NC_031978.2	1691756	1703212	7.525	LOC106096524	deleted in malignant brain tumors 1 protein	A
NC_031978.2	1725702	1736520	7.525	LOC102080493	scavenger receptor cysteine-rich type 1 protein M130	A
NC_031978.2	177025	179795	7.794	rps27a	ribosomal protein S27a	A
NC_031978.2	1794844	1826048	7.525	LOC109194525	inactive ubiquitin carboxyl-terminal hydrolase 54	A
NC_031978.2	179932	195925	7.794	clhc1	clathrin heavy chain linker domain containing 1%2C	A
NC_031978.2	1826019	1828807	7.525	LOC100710888	extensin	A
NC_031978.2	1830199	1836531	7.525	mmrn2	multimerin 2%2C	A
NC_031978.2	196549	198429	7.794	LOC102079711	deleted in malignant brain tumors 1 protein	A
NC_031978.2	310650	374512	10.072	kif20b	kinesin family member 20B%2C	A
NC_031978.2	33021031	33041036	7.802	LOC100696947	sodium/potassium/calcium exchanger 3	A

NC_031978.2	33021031	33271031	7.802	ttc27	tetratricopeptide repeat domain 27%2C	A
NC_031978.2	33225005	33242969	7.802	LOC100698617	pituitary homeobox 3	A
NC_031978.2	3678035	3706398	10.003	LOC100693660	pyridoxal kinase	A
NC_031978.2	3717816	3768928	10.003	itsn1	intersectin 1%2C	A
NC_031978.2	3769663	3776087	10.003	cryzl1	crystallin zeta like 1%2C	A
NC_031978.2	3776081	3783664	10.003	setd4	SET domain containing 4%2C	A
NC_031978.2	3782989	3785749	7.525	LOC100534432	carbonyl reductase-like 20beta-hydroxysteroid dehydrogenase	A
NC_031978.2	3788653	3792822	10.003	LOC100694993	carbonyl reductase [NADPH] 1	A
NC_031978.2	3812066	3819309	8.555	LOC100694734	carbonyl reductase [NADPH] 1-like	A
NC_031978.2	3831014	3832636	10.098	LOC100694465	carbonyl reductase [NADPH] 1	A
NC_031978.2	384296	386073	10.029	LOC109196090	nascent polypeptide-associated complex subunit alpha%2C muscle-specific form-like	A
NC_031978.2	3918753	3958847	8.767	LOC102080562	pro-neuregulin-3%2C membrane-bound	A
NC_031978.2	4558762	4579211	10.150	ccdc172	coiled-coil domain containing 172%2C	A
NC_031978.2	4582719	4686475	10.150	gfra1	GDNF family receptor alpha 1%2C	A
NC_031978.2	4699412	4709455	8.784	LOC100692310	attractin-like protein 1	A
NC_031978.2	5048394	5058046	7.438	LOC100692040	protein kinase C epsilon type	A
NC_031978.2	5146272	5188158	7.592	epas1	endothelial PAS domain protein 1%2C	A
NC_031978.2	5213428	5220995	7.703	LOC100694200	solute carrier family 22 member 7	A
NC_031978.2	5229460	5232057	8.890	LOC100693926	cytochrome c oxidase assembly protein COX20%2C mitochondrial	A
NC_031978.2	5233419	5236091	8.890	LOC100534501	gonadotropin subunit beta-2	A
NC_031978.2	5243628	5259627	7.403	plekha3	pleckstrin homology domain containing A3	A
NC_031978.2	5257408	5282642	7.403	ppp1r1c	protein phosphatase 1 regulatory inhibitor subunit 1C%2C	A
NC_031978.2	5283359	5321398	7.403	nckap1	NCK associated protein 1%2C	A
NC_031978.2	5320339	5333957	8.890	LOC100691233	dual specificity protein phosphatase 19	A
NC_031978.2	543641	553064	8.934	htr7	5-hydroxytryptamine receptor 7	A
NC_031978.2	5562156	5575465	10.170	zc3h15	zinc finger CCCH domain-containing protein 15	A
NC_031978.2	5576218	5583262	10.170	rbm45	RNA binding motif protein 45%2C	A
NC_031978.2	5585087	5589370	10.170	LOC100692041	cytochrome c	A
NC_031978.2	558571	562826	9.446	arfgef3	ARFGEF family member 3%2C	A
NC_031978.2	5738320	5740287	10.949	LOC106097796	zinc finger MYM-type protein 1	A
NC_031978.2	5756415	5758864	10.949	LOC109204767	protein Daple-like	A
NC_031978.2	5870611	5880868	10.949	LOC100690703	gap junction alpha-5 protein	A
NC_031978.2	5885613	5893198	10.949	LOC100690426	follistatin-related protein 1	A
NC_031978.2	5893299	5898300	10.949	LOC102076404	runt-related transcription factor 3	A

NC_031978.2	5895785	5906167	10.949	supt3h	SPT3 homolog%2C SAGA and STAGA complex component%2C	A
NC_031978.2	5906174	5913877	10.949	cdc5l	cell division cycle 5 like	A
NC_031978.2	5919204	5929908	10.949	LOC100691502	beta-soluble NSF attachment protein	A
NC_031978.2	5934750	5957229	7.469	LOC100689896	1-phosphatidylinositol 4%2C5-bisphosphate phosphodiesterase beta-1	A
NC_031978.2	5981763	5987955	8.531	crls1	cardiolipin synthase (CMP-forming)	A
NC_031978.2	5988218	5997651	8.531	mcm8	DNA helicase MCM8	A
NC_031978.2	654112	655017	7.558	ankrd1	ankyrin repeat domain 1	A
NC_031978.2	694348	702402	7.830	pcgf5	polycomb group RING finger protein 5	A
NC_031978.2	7177234	7257847	8.300	LOC100710372	contactin-associated protein-like 4	A
NC_031978.2	7334892	7365569	8.300	LOC102081706	protein FAM161A	A
NC_031978.2	7392611	7407185	8.300	b3gnt2	N-acetyllactosaminide beta-1%2C3-N-acetylglucosaminyltransferase 2	A
NC_031978.2	7611323	7614028	7.815	LOC100712246	homeobox protein OTX1 B	A
NC_031978.2	7622143	7706814	7.486	wdpcp	WD repeat containing planar cell polarity effector%2C	A
NC_031978.2	770479	812467	7.428	hectd2	HECT domain E3 ubiquitin protein ligase 2	A
NC_031978.2	7706955	7713146	7.486	LOC100711978	malate dehydrogenase%2C cytoplasmic	A
NC_031978.2	7713574	7752736	7.486	ugp2	UDP-glucose pyrophosphorylase 2%2C	A
NC_031978.2	7753670	7806447	7.486	LOC100711439	E3 ubiquitin-protein ligase pellino homolog 1	A
NC_031978.2	7825783	7834432	7.486	LOC109204856	ABC transporter G family member 20	A
NC_031978.2	7859877	7861323	7.815	LOC100704123	ABC transporter G family member 23-like	A
NC_031978.2	886814	889224	10.034	ppp1r3c	protein phosphatase 1 regulatory subunit 3C	A
NC_031978.2	920154	927195	10.034	fgfbp3	fibroblast growth factor binding protein 3	A
NC_031978.2	940762	945175	10.034	LOC100705974	D(1) dopamine receptor	A
NC_031978.2	9433965	9568380	7.521	slc8a1	sodium/calcium exchanger 1	A
NC_031978.2	958002	958545	7.475	LOC100709639	SLAM family member 9-like	A
NC_031978.2	959470	960157	8.276	LOC102076678	solute carrier family 22 member 15	A
NC_031978.2	967160	968476	10.034	LOC100709371	D(1C) dopamine receptor-like	A
NC_031978.2	9735552	9774410	7.484	mrps5	28S ribosomal protein S5%2C mitochondrial	A
NC_031978.2	9782317	9789230	7.484	LOC100708039	mal%2C T cell differentiation protein like	A
NC_031978.2	9790812	9798702	7.484	LOC100706613	myelin and lymphocyte protein	A
NC_031978.2	9805574	9817747	7.484	nphp1	nephrocystin 1%2C	A
NC_031978.2	9818432	9821838	7.484	LOC100706081	C-factor	A
NC_031978.2	9825156	9850122	7.484	LOC100705818	pantothenate kinase 1	A
NC_031978.2	9851982	9864029	7.484	LOC100705554	monocarboxylate transporter 12-B	A
NC_031978.2	9890853	9921002	7.484	bcl2l11	BCL2 like 11	A

NC_031978.2	989089	994868	10.015	LOC109204770	C-type lectin domain family 12 member B-like	A
NC_031980.2	16956687	16957974	8.160	atp5mpl	6.8 kDa mitochondrial proteolipid	A
NC_031980.2	16958071	16975575	8.160	garem2	GRB2 associated regulator of MAPK1 subtype 2%2C	A
NC_031980.2	16975972	16985075	8.160	LOC100704158	trifunctional enzyme subunit alpha%2C mitochondrial	A
NC_031980.2	16985268	16992894	8.160	hadhb	hydroxyacyl-CoA dehydrogenase trifunctional multienzyme complex subunit beta	A
NC_031980.2	17095382	17111990	8.160	tfap2d	transcription factor AP-2 delta	A
NC_031980.2	17120619	17132720	8.160	tfap2b	transcription factor AP-2 beta%2C	A
NC_031980.2	21160046	21391442	8.168	LOC106097546	mucin-3A	A
NC_031980.2	21300755	21309022	8.168	LOC109194794	MAP kinase kinase kinase wis4-like	A
NC_031981.2	26244066	26248963	7.430	LOC109200413	probable cyclin-dependent serine/threonine-protein kinase DDB_G0292550	A
NC_031981.2	26248995	26249981	7.430	LOC109200418	phospholipase A2 inhibitor and Ly6/PLAUR domain-containing protein-like	A
NC_031981.2	26276333	26280885	7.430	LOC109200414	nascent polypeptide-associated complex subunit alpha%2C muscle-specific form	A
NC_031981.2	26314163	26316101	7.430	LOC112842621	mucin-5AC-like	A
NC_031981.2	26316092	26318043	7.430	LOC112842694	mucin-2-like	A
NC_031981.2	26423641	26431499	7.430	LOC109195048	integumentary mucin C.1	A
NC_031981.2	26431516	26433923	7.430	LOC109195022	integumentary mucin C.1-like	A
NC_031981.2	26436023	26453598	7.430	LOC109195023	mucin-5AC-like	A
NC_031981.2	26461077	26463397	7.430	LOC109195024	cell wall protein DAN4-like	A
NC_031981.2	26465183	26469849	7.430	LOC109195025	integumentary mucin C.1-like	A
NC_031981.2	26476538	26493367	9.151	LOC109195026	mucin-5AC-like	A
NC_031981.2	34464763	34468966	7.478	LOC102077602	amphoterin-induced protein 2	A
NC_031981.2	34504934	34539735	10.591	LOC100693734	sodium-coupled neutral amino acid transporter 4	A
NC_031981.2	34578873	34589764	10.591	LOC100700135	sodium-coupled neutral amino acid transporter 2	A
NC_031981.2	34592644	34616802	10.591	scaf11	SR-related CTD associated factor 11%2C	A
NC_031981.2	34622099	34661644	10.591	arid2	AT-rich interaction domain 2	A
NC_031981.2	34664115	34693829	10.591	gas2l3	GAS2-like protein 3	A
NC_031981.2	34702190	34705900	7.478	tm7sf3	transmembrane 7 superfamily member 3	A
NC_031981.2	34716661	34718181	8.647	fgfr1op2	FGFR1 oncogene partner 2	A
NC_031981.2	34720607	34721104	7.980	med21	mediator complex subunit 21%2C	A
NC_031981.2	34730845	34731750	7.598	stk38l	serine/threonine kinase 38 like	A
NC_031981.2	34752526	34763123	8.200	LOC100698791	aryl hydrocarbon receptor nuclear translocator-like protein 2	A
NC_031981.2	34779552	34792966	8.186	LOC100698522	sushi domain-containing protein 3	A
NC_031981.2	34814001	34839650	7.787	slc13a4	solute carrier family 13 member 4	A
NC_031981.2	34838236	34841147	7.787	LOC100692481	cytochrome c oxidase assembly factor 6 homolog	A

NC_031981.2	34851274	34912696	7.787	LOC100697991	receptor-type tyrosine-protein phosphatase zeta	A
NC_031981.2	34913060	34922958	7.787	LOC100697729	HMG box-containing protein 1	A
NC_031981.2	34924432	34943576	7.787	pik3cg	phosphatidylinositol 4%2C5-bisphosphate 3-kinase catalytic subunit gamma	A
NC_031982.2	6379878	6398229	8.434	LOC100690141	protein-glutamine gamma-glutamyltransferase K	A
NC_031982.2	6406669	6417661	8.434	nop9	NOP9 nucleolar protein%2C	A
NC_031982.2	6435146	6467795	8.434	rem2	GTP-binding protein REM 2	A
NC_031982.2	6506297	6600814	8.434	LOC102076135	capping protein%2C Arp2/3 and myosin-I linker protein 3	A
NC_031983.2	24392900	24568821	7.461	alk	ALK receptor tyrosine kinase%2C	A
NC_031983.2	24581013	24625990	7.461	clip4	CAP-Gly domain containing linker protein family member 4%2C	A
NC_031983.2	24628312	24629826	7.461	lg19h2orf50	linkage group 19 C2orf50 homolog	A
NC_031983.2	24630900	24642142	7.461	LOC100710049	tripartite motif-containing protein 16-like	A
NC_031983.2	5470107	5558763	7.468	kcnh5	potassium voltage-gated channel subfamily H member 5	A
NC_031983.2	5555297	5562573	7.468	gphb5	glycoprotein hormone beta 5	A
NC_031983.2	5648495	5705672	7.468	syne2	nesprin-2	A
NC_031983.2	5694519	5720107	7.468	esr2	estrogen receptor 2	A
NC_031987.2	8447273	8457533	7.657	arglu1	arginine and glutamate rich 1	A
NC_031987.2	8462447	8488968	7.657	efnb2	ephrin B2%2C	A
NC_031965.2	36014559	36028806	11.587	LOC109202808	adhesion G-protein coupled receptor G1	B
NC_031965.2	36064206	36121241	11.587	LOC100706608	adhesion G-protein coupled receptor G2	B
NC_031965.2	36132394	36146665	11.587	LOC109202805	adhesion G-protein coupled receptor G5-like	B
NC_031965.2	36217883	36228361	11.587	LOC109202809	adhesion G-protein coupled receptor G5-like	B
NC_031965.2	36258289	36261138	8.637	mmp15	matrix metallopeptidase 15	B
NC_031966.2	15664833	15667148	10.151	LOC106097356	signal-regulatory protein beta-2-like	B
NC_031966.2	15696796	15704813	10.151	LOC109203601	signal-regulatory protein beta-2-like	B
NC_031966.2	15752281	15754034	10.151	LOC100702355	twitchin-like	B
NC_031966.2	15867302	15872163	10.151	LOC100703432	T-cell surface glycoprotein CD4-like	B
NC_031966.2	2466983	2470276	10.534	enpp6	ectonucleotide pyrophosphatase/phosphodiesterase 6	B
NC_031966.2	2474399	2488598	10.534	LOC100711332	ETS-related transcription factor Elf-2	B
NC_031966.2	2503635	2506656	10.534	ndufc1	NADH dehydrogenase [ubiquinone] 1 subunit C1%2C mitochondrial	B
NC_031966.2	2506722	2557402	10.534	LOC100695335	N-alpha-acetyltransferase 15%2C NatA auxiliary subunit	B
NC_031966.2	2565944	2589325	10.534	LOC100711605	malate synthase%2C	B
NC_031967.2	11078268	11143476	7.426	nfib	nuclear factor 1 B-type	B
NC_031967.2	11153931	11160186	10.012	zdhhc21	palmitoyltransferase ZDHHC21	B
NC_031967.2	11160855	11166281	10.012	plaa	phospholipase A-2-activating protein	B
NC_031967.2	11167346	11175697	10.012	haus6	HAUS augmin like complex subunit 6	B
NC_031967.2	11173623	11173753	10.012	LOC112846595	small Cajal body-specific RNA 8	B

NC_031967.2	11177143	11217449	10.012	dennd4c	DENN domain containing 4C%2C	B
NC_031967.2	11262788	11265268	10.012	LOC109195697	borealin-2	B
NC_031967.2	11265858	11269498	10.012	plin2	perilipin 2	B
NC_031967.2	11282861	11296573	7.727	adamtsl1	ADAMTS like 1%2C	B
NC_031967.2	11453323	11560118	7.558	col25a1	collagen alpha-1(XXV) chain	B
NC_031967.2	11561543	11570699	10.095	etnppl	ethanolamine-phosphate phospho-lyase	B
NC_031967.2	11570519	11572038	10.095	ostc	oligosaccharyltransferase complex non-catalytic subunit	B
NC_031967.2	11584176	11584889	10.095	LOC109195714	perilipin-2	B
NC_031967.2	11585217	11587758	10.095	LOC102078792	borealin-2	B
NC_031967.2	11590754	11593707	10.095	rpl34	60S ribosomal protein L34	B
NC_031967.2	11603076	11653003	10.095	slc24a2	sodium/potassium/calcium exchanger 2	B
NC_031967.2	11682319	11689614	10.010	lgi2	leucine rich repeat LGI family member 2	B
NC_031967.2	11731818	11745561	10.010	LOC112841621	serine/threonine-protein kinase/endoribonuclease IRE1-like	B
NC_031967.2	11759294	11765161	10.010	LOC106096440	serine/threonine-protein kinase/endoribonuclease IRE1a	B
NC_031967.2	11766348	11780968	10.010	LOC100709990	coiled-coil domain-containing protein 149	B
NC_031967.2	11792004	11798441	8.983	LOC100709718	pre-mRNA-splicing factor ATP-dependent RNA helicase DHX15	B
NC_031967.2	11826210	11892269	8.339	ppargc1a	PPARG coactivator 1 alpha%2C	B
NC_031967.2	15905323	15918964	7.586	capg	capping actin protein%2C gelsolin like%2C	B
NC_031967.2	15950514	15964402	7.586	LOC100702854	calpain-5	B
NC_031967.2	15965295	15968805	7.586	cnpy4	canopy FGF signaling regulator 4	B
NC_031967.2	15977632	15997578	7.586	LOC100703122	netrin-1	B
NC_031967.2	16001948	16021471	7.586	LOC102077238	active breakpoint cluster region-related protein	B
NC_031967.2	16030678	16042411	7.586	LOC106097031	cornifelin homolog B	B
NC_031967.2	16044950	16057787	7.586	LOC109200085	cornifelin homolog B-like	B
NC_031967.2	16129814	16134352	7.586	LOC100700423	cornifelin homolog B%2C	B
NC_031967.2	16138745	16145420	7.586	LOC106097030	cornifelin homolog B-like	B
NC_031967.2	20008770	20028263	7.837	LOC100692450	diamine acetyltransferase 2	B
NC_031967.2	20036995	20038984	7.837	LOC112845256	diamine acetyltransferase 2-like	B
NC_031967.2	20054121	20062402	7.837	LOC112841625	diamine acetyltransferase 2-like	B
NC_031967.2	20067014	20071587	7.837	LOC100704805	ubiquitin carboxyl-terminal hydrolase 2%2C	B
NC_031967.2	20079970	20083365	7.837	LOC100692720	putative E3 ubiquitin-protein ligase ARI6	B
NC_031967.2	20083628	20084703	7.837	LOC100692994	polyubiquitin	B
NC_031967.2	20088578	20097044	7.837	LOC100705341	ubiquitin carboxyl-terminal hydrolase 19	B
NC_031967.2	20117205	20120008	7.837	LOC100693263	probable E3 ubiquitin-protein ligase ARI5	B
NC_031967.2	20121311	20129993	7.837	LOC102081244	ubiquitin carboxyl-terminal hydrolase 17-like protein B	B
NC_031967.2	20133161	20139627	7.837	LOC102081408	ubiquitin carboxyl-terminal hydrolase 17-like protein D	B
NC_031967.2	20256342	20258770	7.837	LOC100706136	probable E3 ubiquitin-protein ligase ARI8	B

NC_031967.2	20694409	20705750	10.365	LOC109196528	leukocyte immunoglobulin-like receptor subfamily B member 3A	B
NC_031967.2	20694409	20944409	10.365	LOC109198185	low affinity immunoglobulin gamma Fc region receptor II-b	B
NC_031967.2	20861718	20944409	10.365	LOC109201287	obscurin	B
NC_031967.2	21085069	21102494	7.403	LOC109196517	peroxidasin homolog	B
NC_031967.2	21142593	21153220	7.403	LOC109201316	high affinity immunoglobulin epsilon receptor subunit alpha-like	B
NC_031967.2	25806994	25926777	10.489	LOC109194483	titin	B
NC_031967.2	25912866	26056994	10.489	LOC112846236	low affinity immunoglobulin gamma Fc region receptor III-like	B
NC_031967.2	25961005	25970564	10.489	LOC106097472	Fc receptor-like protein 5	B
NC_031967.2	25998095	26015209	10.489	LOC112845320	Fc receptor-like protein 5	B
NC_031967.2	26045240	26056611	10.489	LOC112845322	Fc receptor-like protein 5	B
NC_031967.2	29710591	29716904	16.654	LOC109194277	E3 ubiquitin-protein ligase TRIM39-like	B
NC_031967.2	29732366	29734299	16.654	LOC109199711	erythroid membrane-associated protein	B
NC_031967.2	29793262	29800581	16.654	LOC112843099	ribonuclease inhibitor-like	B
NC_031967.2	29804348	29809428	16.654	LOC102082638	E3 ubiquitin-protein ligase TRIM39	B
NC_031967.2	29830128	29834194	16.654	LOC100695258	CMRF35-like molecule 9	B
NC_031967.2	29861829	29944100	16.654	LOC102079294	polymeric immunoglobulin receptor	B
NC_031967.2	29902613	29907759	16.654	LOC109197601	nuclear factor 7%2C ovary-like	B
NC_031967.2	29942615	29944100	16.654	LOC112843390	polymeric immunoglobulin receptor-like	B
NC_031967.2	38541326	38574710	12.897	LOC100706439	kinesin-like protein KIF1C	B
NC_031967.2	38580689	38603752	12.897	LOC100705911	cysteinyl leukotriene receptor 1-like	B
NC_031967.2	38621206	38625268	12.897	LOC102075710	myelin-oligodendrocyte glycoprotein	B
NC_031967.2	38644422	38704304	12.897	LOC102076476	ankyrin repeat domain-containing protein 46	B
NC_031967.2	41213962	41221945	9.042	LOC100700412	protein phosphatase 1K%2C mitochondrial	B
NC_031967.2	41239402	41241948	9.042	LOC112845553	G protein-regulated inducer of neurite outgrowth 1-like	B
NC_031967.2	41252678	41260897	9.042	LOC109203507	toll-like receptor 13	B
NC_031967.2	41260990	41273020	9.042	LOC100700682	toll-like receptor 13	B
NC_031967.2	41271270	41285218	9.042	LOC102080834	toll-like receptor 13	B
NC_031967.2	41291148	41297452	9.042	LOC100700947	toll-like receptor 13	B
NC_031967.2	41302152	41332447	9.042	LOC100701224	RING finger protein 150	B
NC_031967.2	41335861	41338982	9.042	LOC109203648	tripartite motif-containing protein 16-like	B
NC_031967.2	41357038	41358945	9.042	LOC100701496	tripartite motif-containing protein 16-like	B
NC_031967.2	41385659	41387651	9.042	LOC100701769	tripartite motif-containing protein 16	B
NC_031967.2	41403394	41408430	9.042	LOC100702042	tripartite motif-containing protein 16-like	B
NC_031967.2	41435257	41460058	9.042	LOC100702844	type II inositol 3%2C4-bisphosphate 4-phosphatase	B
NC_031967.2	41441609	41444948	9.042	LOC109203650	tripartite motif-containing protein 16-like	B
NC_031967.2	46859269	46890096	10.004	LOC102075779	interferon-induced protein 44	B
NC_031967.2	46940017	46945392	10.004	LOC109196641	C-type lectin BJcuL-like	B

NC_031967.2	47092303	47093821	10.377	LOC100690229	ladderlectin	B
NC_031967.2	52412227	52502241	10.151	LOC100700312	deleted in malignant brain tumors 1 protein	B
NC_031967.2	52515236	52518245	10.151	LOC109202644	Fc receptor-like B	B
NC_031967.2	52635907	52639339	10.151	LOC109199313	nuclear factor 7%2C ovary-like	B
NC_031967.2	52648778	52653076	10.151	LOC112846437	hepatitis A virus cellular receptor 2 homolog	B
NC_031967.2	52659901	52662227	10.151	LOC109199340	V-set and immunoglobulin domain-containing protein 1	B
NC_031967.2	56510457	56543827	9.820	LOC109201101	NACHT%2C LRR and PYD domains-containing protein 12	B
NC_031967.2	56521062	56523318	8.192	LOC102081849	zinc finger BED domain-containing protein 1	B
NC_031967.2	56548292	56556883	8.192	LOC109196839	dynein heavy chain 5%2C axonemal-like	B
NC_031967.2	56603427	56605414	8.192	LOC100690147	lysozyme C	B
NC_031967.2	56618767	56641214	8.192	LOC112845987	NACHT%2C LRR and PYD domains-containing protein 12-like	B
NC_031967.2	56656336	56668905	8.192	LOC102077195	E-selectin-like	B
NC_031967.2	56697907	56702851	8.192	LOC109196842	cell surface A33 antigen-like	B
NC_031967.2	56716429	56760457	9.820	LOC109201128	myelin-oligodendrocyte glycoprotein	B
NC_031967.2	67447955	67479941	16.955	LOC112846006	ribonuclease inhibitor-like	B
NC_031967.2	67491169	67519906	16.955	LOC109199432	NACHT%2C LRR and PYD domains-containing protein 12	B
NC_031967.2	67934645	67934745	7.443	LOC112846587	U6 spliceosomal RNA	B
NC_031967.2	69110258	69122775	8.133	LOC100707056	protein NLRC3	B
NC_031967.2	69143068	69148487	8.133	LOC106097136	CD226 antigen%2C	B
NC_031967.2	69188280	69202330	8.133	LOC112846058	CD226 antigen-like	B
NC_031967.2	69234981	69239084	8.133	LOC102081137	programmed cell death 1 ligand 1	B
NC_031967.2	69262966	69263630	8.133	LOC112846010	Fc receptor-like B	B
NC_031967.2	69265900	69270756	8.133	LOC102080842	myelin protein zero-like protein 2	B
NC_031967.2	70235232	70259635	8.243	LOC106096754	cell surface A33 antigen	B
NC_031967.2	70281074	70303517	10.006	LOC106096749	complement C1q-like protein 4	B
NC_031967.2	70296398	70299184	10.006	LOC106098827	general transcription factor II-I repeat domain-containing protein 2	B
NC_031967.2	70428327	70444580	10.006	LOC109197662	NLR family CARD domain-containing protein 3-like	B
NC_031967.2	77187106	77205828	8.177	nfxl1	NF-X1-type zinc finger protein NFXL1	B
NC_031967.2	77209085	77236061	10.419	corin	atrial natriuretic peptide-converting enzyme	B
NC_031967.2	77243585	77250425	10.419	LOC100703673	sodium/hydrogen exchanger 9B2	B
NC_031967.2	77255116	77269156	10.419	LOC109194306	sodium/hydrogen exchanger 9B2	B
NC_031967.2	77312361	77315916	10.419	LOC102076883	programmed cell death 1 ligand 1	B
NC_031967.2	77328243	77333043	10.419	LOC100700236	matrix remodeling-associated protein 8-like	B
NC_031967.2	77334805	77337967	10.419	LOC102076784	CD226 antigen-like	B
NC_031967.2	77349617	77356941	10.419	LOC102075832	coxsackievirus and adenovirus receptor homolog	B
NC_031967.2	79906945	79934021	11.496	LOC112846023	NLR family CARD domain-containing protein 3-like	B
NC_031967.2	79977610	79982108	11.496	LOC100706428	cytosolic sulfotransferase 3	B

NC_031967.2	80014118	80015688	11.496	LOC109196936	putative nuclease HARBI1	B
NC_031967.2	87041325	87065594	14.654	LOC109197042	NLR family CARD domain-containing protein 3-like	B
NC_031967.2	87090875	87093871	14.654	LOC109201620	nesprin-2-like	B
NC_031967.2	87131491	87136087	16.955	LOC102078820	NLR family CARD domain-containing protein 3-like	B
NC_031967.2	9594124	9597620	7.440	LOC109195644	ribonuclease inhibitor-like	B
NC_031967.2	9699946	9702303	7.440	LOC109195645	zinc finger BED domain-containing protein 1	B
NC_031967.2	9717955	9774980	7.440	LOC100709727	semaphorin-4F	B
NC_031967.2	9835150	9843430	7.440	m1ap	meiosis 1 arrest protein	B
NC_031969.2	11175145	11187322	7.944	LOC102077620	nuclear body protein SP140-like protein	B
NC_031969.2	11194578	11218758	7.525	LOC106098071	GTPase IMAP family member 7	B
NC_031969.2	11236501	11240748	7.525	LOC109201990	GTPase IMAP family member 7	B
NC_031969.2	11265774	11271165	7.525	LOC112841654	GTPase IMAP family member 7-like	B
NC_031969.2	11275159	11424911	7.525	LOC102081056	calponin homology domain-containing protein DDB_G0272472	B
NC_031969.2	11384173	11386612	7.525	LOC100703859	coiled-coil domain-containing protein 97	B
NC_031969.2	12456995	12461569	10.604	ciita	MHC class II transactivator	B
NC_031969.2	12463007	12480225	10.604	nubp1	cytosolic Fe-S cluster assembly factor NUBP1	B
NC_031969.2	12497307	12508340	10.604	emp2	epithelial membrane protein 2	B
NC_031969.2	12509214	12529422	10.604	atf7ip2	activating transcription factor 7 interacting protein 2%2C	B
NC_031969.2	12533300	12550125	10.604	LOC100711957	NACHT%2C LRR and PYD domains-containing protein 12	B
NC_031969.2	12610765	12623423	10.604	LOC102082628	NACHT%2C LRR and PYD domains-containing protein 12	B
NC_031969.2	13212660	13246841	8.027	LOC100690405	GRB2-related adapter protein	B
NC_031969.2	13252458	13270524	8.027	LOC100690681	mucin-12	B
NC_031969.2	13304902	13308191	8.027	LOC112846723	phospholipase A2 inhibitor and Ly6/PLAUR domain-containing protein-like	B
NC_031969.2	13312034	13312981	8.027	LOC112846660	mucin-5AC-like	B
NC_031969.2	13313700	13317238	8.027	LOC112846724	phospholipase A2 inhibitor and Ly6/PLAUR domain-containing protein-like	B
NC_031969.2	13318788	13330725	8.027	LOC112846725	mucin-5AC-like	B
NC_031969.2	13366418	13385895	8.027	LOC100708657	sphingosine kinase 1	B
NC_031969.2	13387394	13387465	8.027	LOC112846803	small nucleolar RNA SNORD49	B
NC_031969.2	13387636	13387705	8.027	LOC112846802	small nucleolar RNA R38	B
NC_031969.2	13387900	13400738	8.027	fbf1	Fas binding factor 1%2C	B
NC_031969.2	13408741	13414301	8.027	LOC100700939	cytoglobin-1	B
NC_031969.2	13415613	13427779	8.027	LOC100700674	phosphoribosyl pyrophosphate synthase-associated protein 1	B
NC_031969.2	13431533	13433115	8.027	LOC100700404	ras-related protein Rap-2a-like	B
NC_031969.2	19252185	19268479	7.541	gpr139	G protein-coupled receptor 139	B
NC_031969.2	19277503	19285479	7.408	LOC100700322	coagulation factor X	B
NC_031969.2	19295755	19312279	7.408	gprc5b	G protein-coupled receptor class C group 5 member B%2C	B
NC_031969.2	19315004	19332023	7.408	iqck	IQ domain-containing protein K	B

NC_031969.2	19333540	19362214	7.408	LOC100710707	SUN domain-containing protein 1	B
NC_031969.2	19366609	19375605	7.408	ccp110	centriolar coiled-coil protein 110	B
NC_031969.2	19431573	19449957	7.408	LOC102081276	nuclear GTPase SLIP-GC	B
NC_031969.2	19473666	19486995	7.408	LOC102082475	nuclear GTPase SLIP-GC	B
NC_031969.2	29042483	29054825	7.833	ccdc47	coiled-coil domain containing 47%2C	B
NC_031969.2	29058909	29081860	7.833	setd1a	SET domain containing 1A%2C	B
NC_031969.2	29086797	29095599	7.833	hsd3b7	3 beta-hydroxysteroid dehydrogenase type 7	B
NC_031969.2	29099941	29111709	7.833	tbc1d10b	TBC1 domain family member 10B	B
NC_031969.2	29111970	29115997	7.833	mylpf	myosin light chain%2C phosphorylatable%2C fast skeletal muscle	B
NC_031969.2	29120266	29125558	7.833	LOC100702252	sesquipedalian-1	B
NC_031969.2	29125049	29131612	7.833	cd2bp2	CD2 antigen cytoplasmic tail-binding protein 2	B
NC_031969.2	29157822	29162115	7.833	lg4h16orf58	RUS1 family protein C16orf58 homolog	B
NC_031969.2	29173288	29178842	7.833	LOC100692966	elongin-B	B
NC_031969.2	29186102	29195451	7.833	armc5	armadillo repeat containing 5	B
NC_031969.2	29195292	29196590	7.833	LOC100701173	cytochrome c oxidase subunit 6A%2C mitochondrial	B
NC_031969.2	29208791	29226307	7.833	LOC100692692	fibroin heavy chain	B
NC_031969.2	29226486	29233427	7.833	LOC100700894	perforin-1	B
NC_031969.2	29234083	29237335	7.833	LOC109202044	perforin-1	B
NC_031969.2	29237809	29244985	7.833	LOC109201769	perforin-1-like	B
NC_031969.2	29247070	29251736	7.833	LOC100692421	perforin-1	B
NC_031969.2	29259747	29271486	7.833	LOC100692151	progestin and adipoQ receptor family member 4	B
NC_031969.2	32495068	32504419	7.751	LOC102076996	probable basic-leucine zipper transcription factor H	B
NC_031969.2	32504353	32524092	7.751	LOC100700268	tetratricopeptide repeat protein 30A	B
NC_031969.2	32523868	32532497	7.751	lg4h16orf89	UPF0764 protein C16orf89 homolog	B
NC_031969.2	32533916	32540333	7.751	alg1	ALG1%2C chitobiosyldiphosphodolichol beta-mannosyltransferase	B
NC_031969.2	32539823	32542945	7.751	eef2kmt	eukaryotic elongation factor 2 lysine methyltransferase%2C	B
NC_031969.2	32543817	32557247	7.751	pigq	phosphatidylinositol N-acetylglucosaminyltransferase subunit Q	B
NC_031969.2	32560076	32585772	7.751	rab40c	RAB40C%2C member RAS oncogene family%2C	B
NC_031969.2	32587087	32592425	7.751	LOC102077529	gastrula zinc finger protein XlCGF57.1	B
NC_031969.2	32595543	32600936	7.751	dnal1	dynein axonemal light chain 1%2C	B
NC_031969.2	32638109	32679938	7.751	LOC100703873	xylosyltransferase 1	B
NC_031969.2	32683134	32688339	7.751	kat8	histone acetyltransferase KAT8	B
NC_031969.2	32689403	32695330	7.751	rnf25	E3 ubiquitin-protein ligase RNF25	B
NC_031969.2	32701327	32708980	7.751	anapc2	anaphase-promoting complex subunit 2	B
NC_031969.2	32709273	32719420	7.751	LOC100703328	ras-related protein Rab-35	B
NC_031969.2	32722204	32725940	7.751	LOC100698395	serine/arginine repetitive matrix protein 2	B
NC_031969.2	6301873	6307161	8.097	eci1	enoyl-CoA delta isomerase 1%2C mitochondrial	B

NC_031969.2	6308568	6314558	8.097	LOC109202009	deoxyribonuclease-1-like	B
NC_031969.2	6331016	6334974	8.097	LOC102083068	zinc finger protein 525	B
NC_031969.2	6339959	6351771	8.097	LOC100700560	deoxyribonuclease-1	B
NC_031969.2	6353097	6356684	8.097	LOC100707017	putative zinc finger protein 876	B
NC_031969.2	6359847	6363633	8.097	naa60	N(alpha)-acetyltransferase 60%2C NatF catalytic subunit%2C	B
NC_031969.2	6369373	6396719	8.097	carhsp1	calcium regulated heat stable protein 1	B
NC_031969.2	6420785	6457789	8.097	rrn3	RNA polymerase I-specific transcription initiation factor RRN3	B
NC_031969.2	9533698	9543296	7.698	LOC100699375	heat-stable enterotoxin receptor	B
NC_031969.2	9546219	9549602	7.698	LOC109201995	piggyBac transposable element-derived protein 2	B
NC_031969.2	9552030	9558053	7.698	LOC100699108	phospholipase B-like 1	B
NC_031969.2	9574655	9631050	7.698	LOC100698838	phospholipase B-like 1	B
NC_031969.2	9636676	9637439	7.698	LOC112846759	pollen-specific leucine-rich repeat extensin-like protein 2	B
NC_031969.2	9641778	9649802	7.698	LOC100698040	phospholipase B-like 1	B
NC_031969.2	9650319	9660692	7.698	pick1	PRKCA-binding protein	B
NC_031969.2	9658339	9662627	7.698	LOC100695382	galectin-2	B
NC_031969.2	9668307	9681980	7.698	LOC102080457	interferon-induced very large GTPase 1	B
NC_031969.2	9706194	9710903	7.698	LOC102080551	interferon-induced very large GTPase 1-like	B
NC_031969.2	9724040	9725787	7.698	LOC100694673	microsomal glutathione S-transferase 1	B
NC_031969.2	9729720	9732180	7.698	LOC109194535	microsomal glutathione S-transferase 1-like	B
NC_031969.2	9741764	9752746	7.698	LOC102078595	interferon-induced very large GTPase 1	B
NC_031969.2	9772256	9783698	7.698	LOC102080640	interferon-induced very large GTPase 1-like	B
NC_031970.2	3637735	3765853	7.642	LOC100707136	dedicator of cytokinesis protein 3	B
NC_031970.2	3697255	3703302	7.642	LOC102081134	butyrophilin subfamily 3 member A2	B
NC_031970.2	3770176	3781937	7.642	LOC102081399	GTPase IMAP family member 8	B
NC_031970.2	3839140	3862031	7.642	LOC109200464	probable polypeptide N-acetylgalactosaminyltransferase 8	B
NC_031970.2	3883158	3887735	7.642	LOC100700530	probable polypeptide N-acetylgalactosaminyltransferase 8	B
NC_031970.2	602792	625724	8.018	LOC102078228	methylglutaconyl-CoA hydratase%2C mitochondrial-like	B
NC_031970.2	625058	627182	8.018	LOC102078049	pre-mRNA-splicing factor 38A	B
NC_031970.2	665690	668444	8.018	LOC109200456	zinc finger BED domain-containing protein 1-like	B
NC_031971.2	1544037	1549461	8.535	LOC109202617	NLR family CARD domain-containing protein 3-like	B
NC_031971.2	1589320	1634831	8.535	LOC100695682	heterogeneous nuclear ribonucleoprotein L-like	B
NC_031971.2	19428868	19442596	8.782	acer2	alkaline ceramidase 2	B
NC_031971.2	19444583	19449023	10.712	rps6	40S ribosomal protein S6	B
NC_031971.2	19454131	19456861	10.712	LOC100692918	perilipin-2	B
NC_031971.2	19456977	19470318	10.712	LOC100707927	WD40 repeat-containing protein SMU1	B
NC_031971.2	19470146	19478753	10.712	LOC100708200	dnaJ homolog subfamily A member 1	B
NC_031971.2	19478657	19482546	10.712	aptx	aprataxin	B

NC_031971.2	19484825	19487834	10.712	LOC100693190	dentin sialophosphoprotein	B
NC_031971.2	19491147	19498409	10.712	LOC100693466	SPARC-like protein 1	B
NC_031971.2	19503630	19506471	10.712	LOC100693995	class E vacuolar protein-sorting machinery protein HSE1-like	B
NC_031971.2	19614181	19615065	10.712	LOC109202643	spore coat protein SP96-like	B
NC_031971.2	19620281	19621195	10.712	LOC102079162	spore coat protein SP65-like	B
NC_031971.2	19660606	19664058	10.712	LOC109202454	proline-rich protein LAS17-like	B
NC_031971.2	31689470	31713471	11.177	LOC100691651	complement C3	B
NC_031971.2	31716141	31747992	11.177	LOC100691917	complement C3	B
NC_031971.2	31751455	31773251	11.177	LOC100700974	complement C3	B
NC_031971.2	31752187	31800704	11.177	LOC100692186	complement C3	B
NC_031971.2	31805495	31820232	11.177	LOC100692725	DENN domain-containing protein 1B	B
NC_031971.2	31820970	31824330	11.177	LOC100703003	tubulin beta-4B chain	B
NC_031971.2	31851458	31882789	7.613	LOC100692999	glucagon receptor	B
NC_031971.2	35727930	35736079	8.321	pcdh18	protocadherin 18%2C	B
NC_031971.2	8372546	8404922	7.707	LOC106096648	voltage-dependent T-type calcium channel subunit alpha-1I	B
NC_031971.2	8435821	8449424	7.707	LOC100710443	GRB2-related adapter protein 2	B
NC_031971.2	8451344	8465170	7.707	LOC100710709	protein FAM83F	B
NC_031971.2	8467364	8479719	7.707	LOC100708025	cytohesin-4	B
NC_031971.2	8486643	8497634	7.707	LOC100708296	ras-related C3 botulinum toxin substrate 2	B
NC_031971.2	8519793	8522112	7.707	LOC102081650	cationic trypsin-like	B
NC_031971.2	8555116	8560206	7.707	LOC109202641	thrombin-like enzyme acutin	B
NC_031972.2	32240603	32285456	7.864	vldlr	very low density lipoprotein receptor%2C	B
NC_031972.2	32289071	32296744	7.864	LOC100696692	potassium voltage-gated channel subfamily V member 2	B
NC_031972.2	32296403	32307678	7.864	pum3	pumilio RNA binding family member 3	B
NC_031972.2	32307920	32334224	7.864	cfap44	cilia and flagella associated protein 44%2C	B
NC_031972.2	32340927	32344730	7.864	LOC100703139	E3 ubiquitin/ISG15 ligase TRIM25-like	B
NC_031972.2	32356299	32357380	7.864	LOC109202988	endonuclease domain-containing 1 protein-like	B
NC_031972.2	32357693	32360561	7.864	LOC112847249	paraneoplastic antigen Ma6E-like	B
NC_031972.2	32364056	32366512	7.864	LOC100695370	tripartite motif-containing protein 16-like	B
NC_031972.2	32376470	32379000	7.864	LOC100695106	tripartite motif-containing protein 16-like	B
NC_031972.2	32381860	32383384	7.864	brcc3	BRCA1/BRCA2-containing complex subunit 3	B
NC_031972.2	32386463	32388157	7.864	LOC100694844	E3 ubiquitin/ISG15 ligase TRIM25-like	B
NC_031972.2	32400329	32462199	7.864	rab27b	RAB27B%2C member RAS oncogene family%2C	B
NC_031973.2	17610165	17860165	8.733	ptprh	protein tyrosine phosphatase%2C receptor type H%2C	B
NC_031973.2	17620978	17622823	8.733	LOC100706346	vitamin K epoxide reductase complex subunit 1	B
NC_031973.2	17622968	17633168	8.733	LOC102082809	mitogen-activated protein kinase 7	B
NC_031973.2	17635315	17637795	8.733	LOC100706181	serine protease 27	B

NC_031973.2	17639244	17649866	8.733	LOC100705912	paired box protein Pax-6	B
NC_031973.2	17653809	17668029	8.733	LOC100706082	matrix metalloproteinase-25	B
NC_031973.2	17669077	17681235	8.733	LOC100705648	carbonic anhydrase 4	B
NC_031973.2	17733207	17740975	8.733	LOC100705383	putative carbonic anhydrase 3	B
NC_031973.2	17749461	17754436	8.733	LOC100705115	sarcoplasmic reticulum histidine-rich calcium-binding protein-like	B
NC_031973.2	17763443	17766507	8.733	LOC100704847	carbonic anhydrase 4	B
NC_031973.2	17813276	17816417	8.733	LOC100704578	carbonic anhydrase 4-like	B
NC_031973.2	17829230	17835249	8.733	LOC100704310	sarcoplasmic reticulum histidine-rich calcium-binding protein	B
NC_031973.2	17836154	17860165	8.733	LOC102075974	transient receptor potential cation channel subfamily M member 4	B
NC_031974.2	15471120	15478589	9.599	LOC100711263	delta-type opioid receptor	B
NC_031974.2	15480357	15483600	9.599	LOC100710999	charged multivesicular body protein 5	B
NC_031974.2	15483616	15488044	9.599	LOC100710732	FAST kinase domain-containing protein 3%2C mitochondrial	B
NC_031974.2	15488709	15492174	9.599	LOC100710463	myosin regulatory light chain 2%2C smooth muscle minor	B
NC_031974.2	15496567	15526423	9.599	myom1	myomesin 1%2C	B
NC_031974.2	15528415	15547169	9.599	lpin2	lipin 2	B
NC_031974.2	15546579	15559597	9.599	emilin2	EMILIN-2	B
NC_031974.2	15559727	15595780	9.599	smchd1	structural maintenance of chromosomes flexible hinge domain containing 1%2C	B
NC_031974.2	15595826	15617270	9.599	LOC100697483	methyltransferase-like protein 4	B
NC_031974.2	15621115	15636753	9.599	LOC102077272	structural maintenance of chromosomes flexible hinge domain-containing protein 1	B
NC_031974.2	24885964	24899840	7.753	LOC109203599	sialic acid-binding Ig-like lectin 7	B
NC_031974.2	24920457	24922053	7.672	LOC109203494	vascular cell adhesion protein 1-like	B
NC_031974.2	24928636	24930189	7.672	LOC109203495	vascular cell adhesion protein 1	B
NC_031974.2	24936006	24940242	7.672	LOC100691759	sialic acid-binding Ig-like lectin 7	B
NC_031974.2	24942470	24944984	7.672	LOC100690693	sialoadhesin	B
NC_031974.2	24958521	24961187	7.672	LOC100690149	sialic acid-binding Ig-like lectin 12	B
NC_031974.2	24994630	24996879	7.672	LOC109203607	vascular cell adhesion protein 1	B
NC_031974.2	25004848	25006929	7.672	LOC109203496	myelin-associated glycoprotein-like	B
NC_031974.2	25024988	25029085	7.672	LOC109194360	myelin-associated glycoprotein	B
NC_031974.2	25052297	25056217	7.672	LOC109203497	B-cell receptor CD22	B
NC_031974.2	25058664	25063443	7.672	LOC109200393	deleted in malignant brain tumors 1 protein	B
NC_031974.2	25095812	25100442	7.672	LOC109200396	deleted in malignant brain tumors 1 protein	B
NC_031974.2	25109683	25119823	7.672	LOC102077673	leukocyte elastase inhibitor%2C	B
NC_031974.2	25136169	25138775	7.908	LOC109200390	general transcription factor II-I repeat domain-containing protein 2-like	B
NC_031974.2	27198454	27203231	8.532	LOC100698997	sialoadhesin-like	B
NC_031974.2	27241913	27245754	8.532	LOC109199459	vascular cell adhesion protein 1-like	B
NC_031974.2	27247934	27250453	8.532	LOC109199458	zinc finger MYM-type protein 1-like	B
NC_031974.2	27259862	27273738	8.532	LOC109194184	sialoadhesin	B

NC_031974.2	27280213	27282796	8.532	LOC109194538	myeloid cell surface antigen CD33	B
NC_031974.2	27291862	27294371	8.532	LOC109203477	leukocyte elastase inhibitor-like	B
NC_031974.2	27295802	27299358	8.532	LOC109194146	transcription factor 7-like 1	B
NC_031974.2	27300025	27309491	8.532	LOC100712479	leukocyte elastase inhibitor	B
NC_031974.2	27311063	27322214	8.532	snx16	sorting nexin 16%2C	B
NC_031974.2	27342424	27367965	8.532	LOC100701023	NEDD4-like E3 ubiquitin-protein ligase WWP1	B
NC_031974.2	27370882	27374809	8.532	rmdn1	regulator of microtubule dynamics 1	B
NC_031974.2	27374873	27384663	8.532	LOC100701296	copine-3	B
NC_031974.2	27388133	27401963	8.532	LOC100701568	copine-3	B
NC_031974.2	27407240	27417818	8.532	LOC100701839	copine-3	B
NC_031974.2	27428417	27434437	8.532	idi1	isopentenyl-diphosphate Delta-isomerase 1	B
NC_031974.2	27433923	27440701	8.532	LOC100707204	WD repeat-containing protein 37	B
NC_031974.2	33333874	33340985	7.509	LOC100711676	microtubule-associated protein 4	B
NC_031974.2	33393400	33395673	10.013	LOC109203478	proline-%2C glutamic acid- and leucine-rich protein 1-like	B
NC_031974.2	33429532	33433090	10.013	LOC109203463	zinc finger MYM-type protein 1	B
NC_031974.2	33436050	33439229	10.013	LOC109203479	proline-rich protein 36-like	B
NC_031974.2	33447003	33583874	7.509	LOC112847800	syncytin-A-like	B
NC_031974.2	33456730	33486303	10.013	LOC106098735	NACHT%2C LRR and PYD domains-containing protein 4E-like	B
NC_031974.2	33510576	33565936	10.013	LOC100690049	phospholipid phosphatase-related protein type 4	B
NC_031974.2	33587517	33612989	9.015	plppr5	phospholipid phosphatase related 5%2C	B
NC_031974.2	34152196	34153670	7.733	LOC109200251	protein ANTAGONIST OF LIKE HETEROCHROMATIN PROTEIN 1	B
NC_031975.2	6988695	7042527	8.041	srrm3	serine/arginine repetitive matrix 3%2C	B
NC_031975.2	7044142	7063079	8.041	LOC102080620	carbohydrate-responsive element-binding protein	B
NC_031975.2	7061998	7100541	8.041	ar	androgen receptor	B
NC_031975.2	7117418	7122847	8.041	LOC100710618	N-acetyllactosaminide beta-1%2C3-N-acetylglucosaminyltransferase 3	B
NC_031975.2	7141662	7158683	8.041	LOC100710883	moesin	B
NC_031975.2	7165795	7179121	8.041	zc3h12b	probable ribonuclease ZC3H12B	B
NC_031975.2	7180121	7191251	8.041	LOC102079444	APC membrane recruitment protein 1	B
NC_031975.2	7191732	7200934	8.041	gab3	GRB2 associated binding protein 3%2C	B
NC_031975.2	7208327	7223462	8.041	aifm1	apoptosis inducing factor mitochondria associated 1%2C	B
NC_031975.2	7224389	7233739	8.041	LOC100704648	glycine receptor subunit alpha-2	B
NC_031975.2	7237957	7238695	8.041	nufip2	NUFIP2%2C FMR1 interacting protein 2	B
NC_031976.2	13698560	13711755	8.744	LOC100704602	plectin	B
NC_031976.2	13734628	13735355	8.744	LOC102075500	hepcidin	B
NC_031976.2	13748968	13750248	8.744	LOC109204099	hepcidin-like	B
NC_031976.2	13750451	13751256	8.744	LOC109204043	hepcidin-like	B
NC_031976.2	13756685	13757482	8.744	LOC109204286	hepcidin-like	B

NC_031976.2	13762863	13763721	8.744	LOC109204285	hepcidin-like	B
NC_031976.2	13778687	13780491	8.744	LOC109204256	hepcidin	B
NC_031976.2	13796449	13797284	8.744	LOC109204092	hepcidin-like	B
NC_031976.2	13800694	13801429	8.744	LOC109204255	hepcidin-like	B
NC_031976.2	13818291	13821669	8.744	LOC100534415	hepcidin	B
NC_031976.2	13834861	13896333	8.744	rspo2	R-spondin 2%2C	B
NC_031976.2	13902418	13930128	8.744	LOC100691937	eukaryotic translation initiation factor 3 subunit E-A	B
NC_031976.2	13938516	13948560	8.744	emc2	ER membrane protein complex subunit 2	B
NC_031976.2	14987188	15052784	8.990	LOC102079407	furin-like protease kpc-1	B
NC_031976.2	15052745	15070450	7.574	mms22l	MMS22 like%2C DNA repair protein	B
NC_031976.2	15070166	15102434	7.574	klhl32	kelch like family member 32%2C	B
NC_031976.2	15103183	15115806	7.574	fzd6	frizzled class receptor 6%2C	B
NC_031976.2	15115849	15121915	7.574	cthrc1	collagen triple helix repeat containing 1%2C	B
NC_031976.2	15122005	15126971	7.574	slc25a32	mitochondrial folate transporter/carrier	B
NC_031976.2	15127477	15141801	7.574	dcaf13	DDB1 and CUL4 associated factor 13	B
NC_031976.2	15187776	15237188	8.990	rims2	regulating synaptic membrane exocytosis protein 2	B
NC_031976.2	16738840	16747294	8.533	LOC102076445	myelin-associated glycoprotein	B
NC_031976.2	16754897	16759214	8.533	LOC109194180	sialoadhesin	B
NC_031976.2	16825654	16830812	8.533	LOC100703576	C3a anaphylatoxin chemotactic receptor-like	B
NC_031976.2	16837460	16838273	8.533	LOC100703305	chemokine-like receptor 1	B
NC_031976.2	16842937	16850336	8.533	LOC100703034	chemokine-like receptor 1	B
NC_031976.2	16868614	16872465	8.533	LOC100702766	C5a anaphylatoxin chemotactic receptor 1-like	B
NC_031976.2	16917267	16931359	8.533	LOC100709210	chemokine-like receptor 1	B
NC_031976.2	16933306	16940496	8.533	LOC100708939	chemokine-like receptor 1	B
NC_031976.2	16958843	16971779	9.354	LOC100708394	chemokine-like receptor 1	B
NC_031976.2	16990062	16991111	9.354	LOC100708124	chemokine-like receptor 1	B
NC_031976.2	17013985	17015598	9.354	LOC109204401	chemokine-like receptor 1	B
NC_031976.2	17029783	17030855	9.354	LOC102076817	chemokine-like receptor 1	B
NC_031976.2	17040823	17041943	9.354	LOC100707854	chemokine-like receptor 1	B
NC_031976.2	17055651	17061147	9.354	LOC100707054	chemokine-like receptor 1	B
NC_031976.2	2423185	2539915	7.428	cdk14	cyclin dependent kinase 14%2C	B
NC_031976.2	25422171	25426769	11.898	bcl3	B cell CLL/lymphoma 3%2C	B
NC_031976.2	25428302	25439262	11.898	LOC100698984	probable ATP-dependent RNA helicase ddx6	B
NC_031976.2	25441400	25450426	11.898	LOC100696843	mitochondrial import receptor subunit TOM40 homolog	B
NC_031976.2	2545891	2561889	7.428	LOC100706383	pituitary tumor-transforming gene 1 protein-interacting protein	B
NC_031976.2	25463046	25479378	11.898	LOC102080243	meprin A subunit beta-like	B
NC_031976.2	25601760	25610416	11.898	LOC109204053	hemicentin-2	B

NC_031976.2	26194413	26223508	7.462	LOC100701297	forkhead box protein O1-B	B
NC_031976.2	26228196	26231943	7.462	med18	mediator complex subunit 18%2C	B
NC_031976.2	26238088	26244657	7.462	LOC100694213	cytochrome P450 4B1	B
NC_031976.2	26256248	26262381	7.462	LOC102079868	cytochrome P450 4B1	B
NC_031976.2	26273148	26278243	7.462	LOC100701569	cytochrome P450 4B1	B
NC_031976.2	26281802	26289158	7.462	ppp1r8	nuclear inhibitor of protein phosphatase 1	B
NC_031976.2	26288234	26288364	7.462	LOC112848293	small Cajal body-specific RNA 1	B
NC_031976.2	26290272	26296441	7.462	themis2	protein THEMIS2	B
NC_031976.2	26296849	26303380	7.462	rpa2	replication protein A 32 kDa subunit	B
NC_031976.2	26304186	26308226	7.462	smpdl3b	acid sphingomyelinase-like phosphodiesterase 3b	B
NC_031976.2	26307980	26311448	7.462	mecr	enoyl-[acyl-carrier-protein] reductase%2C mitochondrial	B
NC_031976.2	26312593	26327048	7.462	LOC100692547	protein lin-28 homolog A-like	B
NC_031976.2	26327679	26340000	7.462	maneal	glycoprotein endo-alpha-1%2C2-mannosidase-like protein	B
NC_031976.2	26339890	26344759	7.462	yrdc	yrdC N6-threonylcarbamoyltransferase domain containing	B
NC_031976.2	26350314	26363755	7.462	mtf1	metal regulatory transcription factor 1	B
NC_031976.2	26363894	26383847	7.462	inpp5b	inositol polyphosphate-5-phosphatase B%2C	B
NC_031976.2	26388815	26391679	7.462	LOC102083321	probable cyclin-dependent serine/threonine-protein kinase DDB_G0292550	B
NC_031976.2	35776767	35796984	7.503	LOC102080704	putative ferric-chelate reductase 1	B
NC_031976.2	35826416	35845744	7.503	LOC100698420	membrane-spanning 4-domains subfamily A member 8	B
NC_031976.2	35854376	35864948	7.503	LOC100707639	B-lymphocyte antigen CD20	B
NC_031976.2	35924940	35931609	7.503	LOC100707375	high affinity immunoglobulin epsilon receptor subunit beta	B
NC_031976.2	35932499	35937482	7.503	tmem176b	transmembrane protein 176B	B
NC_031976.2	35946048	35949957	7.503	lamtor2	late endosomal/lysosomal adaptor%2C MAPK and MTOR activator 2	B
NC_031976.2	35951962	35966753	7.503	rab25	RAB25%2C member RAS oncogene family	B
NC_031976.2	35970770	35985097	7.503	LOC100706302	ras-related protein Rab-11A	B
NC_031976.2	35985404	35998447	7.503	LOC100697624	RNA-binding protein MEX3A	B
NC_031976.2	36005629	36024156	7.503	lmna	lamin	B
NC_031976.2	36859374	36879999	9.434	LOC102083175	semaphorin-4B	B
NC_031976.2	36882771	36912872	9.434	adam15	ADAM metallopeptidase domain 15	B
NC_031976.2	36916434	36925342	9.434	dcst1	DC-STAMP domain containing 1%2C	B
NC_031976.2	36924720	36943495	9.434	dcst2	DC-STAMP domain containing 2	B
NC_031976.2	37002716	37014323	9.434	LOC112848161	C-type mannose receptor 2-like	B
NC_031976.2	37044076	37093727	9.434	nek11	NIMA related kinase 11	B
NC_031977.2	16471284	16476982	7.939	edil3	EGF like repeats and discoidin domains 3%2C	B
NC_031977.2	16542693	16550167	7.939	LOC100706323	hyaluronan and proteoglycan link protein 1	B
NC_031977.2	16552047	16572922	7.939	LOC100706060	versican core protein	B
NC_031977.2	16583441	16605952	7.483	LOC109204414	versican core protein-like	B

NC_031977.2	16615767	16642477	7.483	xrcc4	DNA repair protein XRCC4	B
NC_031977.2	16642347	16648247	7.483	tmem167a	protein kish-A	B
NC_031977.2	16666054	16668099	7.483	LOC106096593	piggyBac transposable element-derived protein 4-like	B
NC_031977.2	16672789	16679496	7.483	LOC102081075	arrestin domain-containing protein 3-like	B
NC_031977.2	16690213	16693839	7.483	LOC100705535	arrestin domain-containing protein 3-like	B
NC_031977.2	16694134	16701581	7.483	LOC100705267	arrestin domain-containing protein 2	B
NC_031977.2	16708067	16710751	7.483	LOC100705000	arrestin domain-containing protein 3	B
NC_031977.2	16712744	16715303	7.483	LOC100704729	arrestin domain-containing protein 3	B
NC_031977.2	16742908	16800678	7.483	ssbp2	single stranded DNA binding protein 2	B
NC_031977.2	30703915	30720511	8.826	LOC100693557	sushi domain-containing protein 2	B
NC_031977.2	30720585	30723365	10.135	LOC100693286	retinol dehydrogenase 14	B
NC_031977.2	30729064	30731663	10.135	LOC106098900	tripartite motif-containing protein 35-like	B
NC_031977.2	30759083	30760673	10.135	LOC102060415	nuclear factor 7%2C brain-like	B
NC_031977.2	30765600	30766739	10.135	LOC109204424	zinc-binding protein A33-like	B
NC_031977.2	30773098	30781630	10.135	LOC106096969	tripartite motif-containing protein 35-like	B
NC_031977.2	30786021	30788293	10.135	LOC106096970	nuclear factor 7%2C brain	B
NC_031977.2	30795853	30813170	10.135	LOC109204381	nuclear factor 7%2C brain-like	B
NC_031977.2	30795986	30798495	10.135	LOC106096968	nuclear factor 7%2C brain-like	B
NC_031977.2	30818094	30819597	10.135	LOC109204497	nuclear factor 7%2C brain-like	B
NC_031977.2	30825595	30827997	10.135	LOC109204384	nuclear factor 7%2C brain-like	B
NC_031977.2	30851263	30853750	10.135	LOC109204572	nuclear factor 7%2C brain-like	B
NC_031977.2	30854586	30934735	10.135	LOC100695100	calcium/calmodulin-dependent protein kinase type II delta chain	B
NC_031977.2	30939773	30952709	10.135	LOC102077389	protein transport protein Sec24C	B
NC_031977.2	30966435	30968086	7.678	LOC106097377	nuclear factor 7%2C brain	B
NC_031977.2	30974489	30976817	7.678	LOC109204649	nuclear factor 7%2C brain-like	B
NC_031977.2	30998768	31000804	7.678	LOC100698454	tripartite motif-containing protein 35-like	B
NC_031977.2	35912770	35919707	13.284	LOC100703707	fructose-1%2C6-bisphosphatase isozyme 2	B
NC_031977.2	35920443	35924647	10.390	LOC100703974	cathepsin L1	B
NC_031977.2	35934494	36013715	10.390	dapk1	death associated protein kinase 1%2C	B
NC_031977.2	36051868	36056303	10.390	LOC100700363	granzyme K	B
NC_031977.2	36078534	36082239	10.390	LOC100700638	granzyme K-like	B
NC_031977.2	36100428	36103106	10.390	LOC100700901	granzyme K	B
NC_031977.2	36111883	36116613	10.390	LOC100701180	granzyme K-like	B
NC_031977.2	36133356	36135690	10.390	LOC106098174	granzyme K-like	B
NC_031977.2	36455896	36473897	7.412	LOC102079812	GTPase IMAP family member 2	B
NC_031977.2	36470115	36472183	7.412	LOC106097401	zinc finger MYM-type protein 1	B
NC_031977.2	36488180	36489356	7.412	LOC100695451	poly [ADP-ribose] polymerase 14	B

NC_031977.2	36494296	36506319	7.412	LOC100695189	poly [ADP-ribose] polymerase 14	B
NC_031977.2	36516610	36526494	7.412	LOC100694918	GTPase IMAP family member 8-like	B
NC_031977.2	36542175	36566833	7.412	LOC100694656	poly [ADP-ribose] polymerase 14	B
NC_031977.2	36591695	36602072	7.412	LOC102080448	GTPase IMAP family member 8	B
NC_031977.2	36620684	36639479	7.412	LOC100694118	GTPase IMAP family member 8	B
NC_031977.2	36643253	36678724	7.412	LOC109204452	zinc finger BED domain-containing protein 4-like	B
NC_031977.2	36723081	36774584	7.456	LOC100703505	GTPase IMAP family member 8	B
NC_031977.2	36827640	36843223	7.456	LOC100711088	GTPase IMAP family member 8-like	B
NC_031977.2	36946047	36965842	7.521	LOC100712315	rap guanine nucleotide exchange factor 1	B
NC_031977.2	38359316	38361636	11.776	srp19	signal recognition particle 19	B
NC_031977.2	38483521	38503954	11.146	LOC100710015	phosphatidylinositol 3-kinase regulatory subunit alpha	B
NC_031977.2	38504630	38519479	11.146	LOC100711619	shieldin complex subunit 3	B
NC_031977.2	38521154	38535204	11.146	LOC100710281	serine/threonine-protein phosphatase 2A catalytic subunit beta	B
NC_031978.2	30129806	30168798	10.128	fancl	E3 ubiquitin-protein ligase FANCL	B
NC_031978.2	30167514	30198759	10.128	vrk2	serine/threonine-protein kinase VRK2	B
NC_031978.2	30266838	30269445	9.825	LOC100708994	cationic amino acid transporter 2	B
NC_031978.2	33021031	33041036	8.330	LOC100696947	sodium/potassium/calcium exchanger 3	B
NC_031978.2	33021031	33271031	8.330	ttc27	tetratricopeptide repeat domain 27%2C	B
NC_031978.2	33225005	33242969	7.492	LOC100698617	pituitary homeobox 3	B
NC_031978.2	34311695	34326714	8.511	LOC100697919	blastomere cadherin	B
NC_031979.2	32948523	32955681	8.173	treh	trehalase	B
NC_031979.2	32955748	32967255	8.173	ddx6	DEAD-box helicase 6%2C	B
NC_031979.2	32970731	33034214	8.173	cxcr5	C-X-C chemokine receptor type 5	B
NC_031979.2	32984173	33012437	8.173	bcl9l	B cell CLL/lymphoma 9 like%2C	B
NC_031979.2	33034163	33060601	8.173	cdon	cell adhesion associated%2C oncogene regulated%2C	B
NC_031980.2	27831308	27900477	9.473	LOC100710023	opsin-5	B
NC_031980.2	9226106	9232784	7.541	LOC100690064	steroid hormone receptor ERR2	B
NC_031980.2	9249837	9278342	7.541	ylpm1	YLP motif containing 1%2C	B
NC_031980.2	9272065	9281200	7.541	LOC102082270	prospero homeobox protein 2	B
NC_031980.2	9281254	9289326	7.541	LOC100689795	dihydrolipoyllysine-residue succinyltransferase component of 2-oxoglutarate dehydrogenase complex%	B
NC_031980.2	9288834	9298680	7.541	LOC102081508	leucine-rich repeat-containing protein 74A	B
NC_031980.2	9301207	9311359	7.541	LOC100712416	jun dimerization protein 2	B
NC_031980.2	9321707	9325129	7.541	LOC100709467	proto-oncogene c-Fos	B
NC_031980.2	9328987	9331524	7.541	LOC100709203	proto-oncogene c-Fos	B
NC_031980.2	9339920	9345325	7.541	tmed10	transmembrane emp24 domain-containing protein 10	B
NC_031980.2	9349169	9352294	7.541	eif2b2	eukaryotic translation initiation factor 2B subunit beta	B
NC_031980.2	9437097	9443030	7.541	LOC100712477	visual system homeobox 2	B

NC_031980.2	9444889	9457538	7.541	LOC100712210	acetyl-coenzyme A synthetase 2-like%2C mitochondrial	B
NC_031980.2	9457705	9467534	7.541	abcd4	ATP binding cassette subfamily D member 4	B
NC_031980.2	9468849	9476106	7.541	LOC100711669	sphingosine-1-phosphate phosphatase 1	B
NC_031981.2	15557237	15706900	7.911	ppfia2	PTPRF interacting protein alpha 2%2C	B
NC_031981.2	15706894	15756616	7.911	acss3	acyl-CoA synthetase short chain family member 3%2C	B
NC_031981.2	15763304	15805768	7.911	lin7a	lin-7 homolog A%2C crumbs cell polarity complex component%2C	B
NC_031981.2	21203001	21205306	7.435	LOC106097885	zinc finger BED domain-containing protein 1	B
NC_031981.2	21214008	21242474	7.435	cpm	carboxypeptidase M	B
NC_031981.2	21245464	21250319	7.435	LOC102082896	overexpressed in colon carcinoma 1 protein	B
NC_031981.2	21258619	21265220	7.435	dclre1c	DNA cross-link repair 1C%2C	B
NC_031981.2	21265947	21274576	7.435	tmem243	transmembrane protein 243	B
NC_031981.2	21275056	21285135	7.435	dmtf1	cyclin-D-binding Myb-like transcription factor 1	B
NC_031981.2	21285803	21294636	7.435	cwf19l1	CWF19 like cell cycle control factor 1	B
NC_031981.2	21294917	21317348	7.435	LOC100690342	CD9 antigen	B
NC_031981.2	21319564	21335045	7.435	lg17h12orf56	linkage group 17 C12orf56 homolog%2C	B
NC_031981.2	21336482	21347270	7.435	LOC100693577	acyl-coenzyme A thioesterase 1	B
NC_031981.2	21355768	21370776	7.435	LOC100693306	protein NLRC3	B
NC_031981.2	21372898	21381927	7.435	LOC100693038	tetraspanin-8	B
NC_031981.2	21384654	21409634	7.435	LOC102075864	tetraspanin-33	B
NC_031981.2	21420299	21425955	7.435	LOC100692765	NACHT%2C LRR and PYD domains-containing protein 12	B
NC_031981.2	22677843	22685076	10.089	ncaph2	condensin-2 complex subunit H2	B
NC_031981.2	22684370	22687602	10.089	LOC100698329	protein SCO2 homolog%2C mitochondrial	B
NC_031981.2	22690146	22712528	10.089	LOC102077806	coiled-coil domain-containing protein 136	B
NC_031981.2	22714354	22768867	10.089	LOC100698591	filamin-C	B
NC_031981.2	22770301	22776178	10.089	socs2	suppressor of cytokine signaling 2	B
NC_031981.2	22780935	22787578	10.089	LOC100698862	death domain-containing protein CRADD	B
NC_031981.2	22792087	22837921	10.089	ptpro	protein tyrosine phosphatase%2C receptor type O%2C	B
NC_031981.2	22842145	22874679	8.112	LOC100699396	epidermal growth factor receptor kinase substrate 8	B
NC_031981.2	22885298	22898302	7.961	dusp16	dual specificity protein phosphatase 16	B
NC_031981.2	22903617	22907961	7.961	crebl2	cAMP responsive element binding protein like 2	B
NC_031981.2	22908928	22915864	7.961	gpr19	G protein-coupled receptor 19%2C	B
NC_031982.2	23871683	23909094	8.730	LOC100691642	netrin-G1	B
NC_031982.2	23975981	23983276	8.730	LOC109194289	pancreatic alpha-amylase	B
NC_031982.2	23999277	24003712	8.730	LOC100534494	pancreatic alpha-amylase	B
NC_031982.2	24010564	24015425	8.730	LOC100701014	pancreatic alpha-amylase	B
NC_031982.2	24030513	24035545	8.730	LOC109195663	pancreatic alpha-amylase-like	B
NC_031982.2	24043624	24050119	8.730	LOC109195662	pancreatic alpha-amylase	B

NC_031982.2	24098019	24121683	8.730	LOC100700290	collagen alpha-1(XI) chain	B
NC_031982.2	32125312	32134587	10.060	LOC100710992	NLR family CARD domain-containing protein 3-like	B
NC_031982.2	32145208	32169506	10.060	LOC102076273	NLR family CARD domain-containing protein 3	B
NC_031982.2	32209102	32211167	10.060	LOC109195560	tripartite motif-containing protein 16-like	B
NC_031982.2	32218177	32221426	10.060	LOC109195431	zinc finger protein 665-like	B
NC_031982.2	32287341	32287501	10.060	LOC112843009	U1 spliceosomal RNA	B
NC_031982.2	32287681	32287841	10.060	LOC112842998	U1 spliceosomal RNA	B
NC_031982.2	32288953	32289124	10.060	LOC112843017	U1 spliceosomal RNA	B
NC_031982.2	32289305	32289465	10.060	LOC112843008	U1 spliceosomal RNA	B
NC_031982.2	32289647	32289808	10.060	LOC112842997	U1 spliceosomal RNA	B
NC_031982.2	32290920	32291091	10.060	LOC112843012	U1 spliceosomal RNA	B
NC_031982.2	32291272	32291433	10.060	LOC112843002	U1 spliceosomal RNA	B
NC_031982.2	32291615	32291776	10.060	LOC112843007	U1 spliceosomal RNA	B
NC_031982.2	32291958	32292121	10.060	LOC112843028	U1 spliceosomal RNA	B
NC_031982.2	32293236	32293407	10.060	LOC112843018	U1 spliceosomal RNA	B
NC_031982.2	32293588	32293751	10.060	LOC112843001	U1 spliceosomal RNA	B
NC_031982.2	32293932	32294095	10.060	LOC112842990	U1 spliceosomal RNA	B
NC_031982.2	32337832	32345694	10.060	znf622	zinc finger protein 622	B
NC_031982.2	32352026	32359919	8.090	march11	E3 ubiquitin-protein ligase MARCH11	B
NC_031982.2	7563488	7563550	13.824	mcm6	DNA replication licensing factor MCM6	B
NC_031982.2	7567296	7577797	10.893	LOC100701870	protein lifeguard 3	B
NC_031982.2	7579262	7589952	10.893	LOC100702139	caspase-8	B
NC_031982.2	7589059	7593534	10.893	catip	ciliogenesis associated TTC17 interacting protein%2C	B
NC_031982.2	7595310	7597386	10.893	LOC100702676	caspase-8	B
NC_031982.2	7634989	7640370	10.893	LOC102079913	caspase-8	B
NC_031982.2	7650302	7655687	10.893	LOC106097537	retrovirus-related Pol polyprotein from transposon 412	B
NC_031982.2	7688213	7691174	10.893	LOC100702924	GTPase IMAP family member 4-like	B
NC_031982.2	7712737	7716058	10.893	LOC109195365	zinc finger MYM-type protein 1-like	B
NC_031982.2	7748687	7752816	10.893	LOC100701212	GTPase IMAP family member 4-like	B
NC_031982.2	7775403	7782285	10.893	LOC100700669	GTPase IMAP family member 4	B
NC_031982.2	7785691	7788603	10.893	LOC109194579	GTPase IMAP family member 4-like	B
NC_031982.2	7805588	7808624	10.893	LOC100699857	GTPase IMAP family member 4-like	B
NC_031983.2	18042301	18044937	7.926	yju2	YJU2 splicing factor homolog	B
NC_031983.2	18046326	18047415	7.766	LOC100708247	cocaine- and amphetamine-regulated transcript protein	B
NC_031983.2	18047873	18059905	7.766	LOC100702708	microtubule-associated protein 1S	B
NC_031983.2	18060775	18064987	7.766	LOC100702439	interferon-induced protein 44	B
NC_031983.2	18065486	18067640	7.766	LOC100707977	growth arrest and DNA damage-inducible protein GADD45 beta	B

NC_031983.2	18069042	18077829	7.766	gng7	G protein subunit gamma 7%2C	B
NC_031983.2	18080709	18096175	7.766	LOC100707435	GTP-binding protein Di-Ras1	B
NC_031983.2	18098614	18106610	7.766	LOC100707170	receptor expression-enhancing protein 5	B
NC_031983.2	18108470	18155340	7.766	LOC100701627	Ig kappa chain V region Mem5-like	B
NC_031983.2	18111001	18144919	7.766	LOC112843186	Ig kappa chain V-III region MOPC 63-like	B
NC_031983.2	18113529	18122395	7.766	LOC100701901	excitatory amino acid transporter 5	B
NC_031983.2	18123215	18133028	7.766	LOC100706107	ELAV-like protein 1	B
NC_031983.2	18135766	18140750	7.766	LOC102076783	tetraspanin-3	B
NC_031983.2	18145239	18146054	7.766	LOC102077194	Ig kappa chain V region 120-like	B
NC_031983.2	18147703	18194358	7.766	LOC109195905	Ig lambda-1 chain C region-like	B
NC_031983.2	18152985	18154113	7.766	LOC109195891	immunoglobulin kappa variable 6D-21-like	B
NC_031983.2	18156107	18163874	7.766	LOC109195882	Ig kappa chain V region 3381-like	B
NC_031983.2	18159852	18160519	7.766	LOC109195815	immunoglobulin kappa variable 1D-16-like	B
NC_031983.2	18168721	18169470	7.766	LOC102076682	immunoglobulin kappa variable 4-1-like	B
NC_031983.2	18169887	18171264	7.766	LOC109195879	Ig lambda chain C region-like	B
NC_031983.2	18174438	18175024	7.766	LOC109195902	Ig kappa chain V region 3381-like	B
NC_031983.2	18183234	18184138	7.766	LOC102076501	Ig kappa chain V region 3381-like	B
NC_031983.2	18191789	18192491	7.766	LOC109195894	Ig kappa chain V-VI region NQ2-48.2.2-like	B
NC_031983.2	18192523	18205351	7.766	LOC106098051	Ig lambda chain C region-like	B
NC_031983.2	18195419	18196154	7.766	LOC109195885	Ig kappa chain V region 4135-like	B
NC_031983.2	18202072	18202716	7.766	LOC112843190	Ig kappa chain V-VI region NQ2-48.2.2-like	B
NC_031983.2	18209569	18227968	7.766	LOC109195812	Ig kappa chain C region%2C B allele-like	B
NC_031983.2	18212931	18213590	7.766	LOC109195896	Ig kappa chain V region BS-5-like	B
NC_031983.2	18218162	18218744	7.766	LOC109195895	Ig kappa chain V region BS-5-like	B
NC_031983.2	18225637	18226496	7.766	LOC109195901	Ig kappa chain V region BS-5-like	B
NC_031983.2	18228958	18229841	7.766	LOC102075657	immunoglobulin kappa variable 4-1-like	B
NC_031983.2	18230190	18239551	7.766	LOC109195906	Ig kappa chain C region%2C B allele-like	B
NC_031983.2	18233073	18234249	7.766	LOC109195811	Ig kappa chain V region 3547-like	B
NC_031983.2	18236821	18237411	7.766	LOC109195883	immunoglobulin kappa variable 4-1-like	B
NC_031983.2	18243311	18244028	7.766	LOC109195893	Ig kappa chain V region BS-5-like	B
NC_031983.2	18245267	18245593	7.766	LOC109195904	Ig lambda chain C region-like	B
NC_031983.2	18250139	18251779	7.766	LOC102083289	Ig kappa-b4 chain C region-like	B
NC_031983.2	18254275	18254808	7.766	LOC109195810	Ig kappa chain V region 3381-like	B
NC_031983.2	18258226	18260068	7.766	LOC106098056	Ig kappa chain C region%2C B allele-like	B
NC_031983.2	18262196	18263833	7.766	LOC109195880	Ig kappa chain C region%2C B allele-like	B
NC_031983.2	18267088	18267935	7.766	LOC109195890	Ig kappa chain V region BS-5-like	B
NC_031983.2	18268407	18290954	7.766	LOC100699464	Ig kappa chain C region%2C B allele-like	B

NC_031983.2	18276460	18277979	7.766	LOC109195876	Ig lambda chain C region-like	B
NC_031983.2	18280161	18280899	7.766	LOC109195903	immunoglobulin kappa variable 1-6-like	B
NC_031983.2	18283236	18284231	7.766	LOC109195888	Ig kappa chain V region 3381-like	B
NC_031983.2	18286016	18289149	7.766	LOC109195900	Ig kappa chain V region 4135-like	B
NC_031983.2	20075428	20086275	7.805	stag1	cohesin subunit SA-1	B
NC_031983.2	20093592	20098913	7.805	LOC100690726	solute carrier family 35 member G2	B
NC_031983.2	20101463	20113723	7.805	LOC109194286	endophilin-B1	B
NC_031983.2	20145312	20164134	7.805	atr	ATR serine/threonine kinase%2C	B
NC_031983.2	20165066	20202934	7.805	LOC100711732	plastin-1	B
NC_031983.2	20205991	20215904	7.805	LOC100711459	glycogenin-1	B
NC_031983.2	20216778	20231495	7.805	hltf	helicase like transcription factor%2C	B
NC_031983.2	20232586	20248903	7.805	LOC100710923	serine/threonine-protein kinase PAK 2	B
NC_031983.2	20251792	20273169	7.805	LOC100710657	otoferlin	B
NC_031983.2	20274821	20281153	7.805	LOC102078875	ensconsin	B
NC_031983.2	20280997	20290553	7.805	LOC102078694	vacuolar protein 8	B
NC_031983.2	20291140	20310442	7.805	ahi1	Abelson helper integration site 1%2C	B
NC_031983.2	20301298	20309034	7.805	LOC100710131	MFS-type transporter SLC18B1	B
NC_031983.2	20310283	20318232	7.805	LOC100709864	MFS-type transporter SLC18B1	B
NC_031983.2	20319559	20325428	7.805	LOC100698304	tubby-related protein 4	B
NC_031983.2	4990258	5036883	8.291	akap6	A-kinase anchor protein 6	B
NC_031983.2	5039268	5090984	8.291	arhgap5	Rho GTPase activating protein 5%2C	B
NC_031983.2	5128702	5136207	8.291	nubpl	iron-sulfur protein NUBPL	B
NC_031983.2	5137326	5148536	8.291	LOC100692408	histone-lysine N-methyltransferase 2B	B
NC_031983.2	5183108	5187102	8.291	yipf6	Yip1 domain family member 6	B
NC_031983.2	5187516	5191369	8.291	snapc1	small nuclear RNA activating complex polypeptide 1	B
NC_031983.2	5192658	5240258	8.291	syt16	synaptotagmin 16%2C	B
NC_031983.2	5248720	5267501	9.795	sgpp1	sphingosine-1-phosphate phosphatase 1	B
NC_031983.2	5271626	5274116	9.795	wdr89	WD repeat-containing protein 89	B
NC_031983.2	5278950	5330039	9.795	ppp2r5e	protein phosphatase 2 regulatory subunit B'epsilon%2C	B
NC_031983.2	5359054	5498720	9.795	kcnh5	potassium voltage-gated channel subfamily H member 5	B
NC_031983.2	6144111	6209210	8.293	LOC100698875	transcription factor IIIB 90 kDa subunit	B
NC_031983.2	6589056	6589500	7.412	LOC109195942	tumor necrosis factor alpha-induced protein 2	B
NC_031983.2	6589056	6602942	7.412	LOC100693617	tumor necrosis factor alpha-induced protein 2	B
NC_031983.2	6604770	6615481	11.475	LOC106098437	tumor necrosis factor alpha-induced protein 2	B
NC_031983.2	6628629	6660247	11.475	LOC100692806	exocyst complex component 3-like protein 4	B
NC_031983.2	6662650	6682401	10.058	LOC109195939	tumor necrosis factor alpha-induced protein 2	B
NC_031983.2	6683759	6715745	10.058	LOC100692537	tumor necrosis factor alpha-induced protein 2	B

NC_031983.2	6722433	6742062	10.058	LOC100692266	tumor necrosis factor alpha-induced protein 2	B
NC_031983.2	6750156	6752582	10.058	LOC109195821	exocyst complex component 3-like protein 2	B
NC_031983.2	6767479	6784106	10.058	LOC109195941	tumor necrosis factor alpha-induced protein 2	B
NC_031983.2	6793445	6822741	10.058	LOC109195940	tumor necrosis factor alpha-induced protein 2	B
NC_031983.2	6851676	6865280	10.058	LOC100691460	protein LBH	B
NC_031983.2	6866677	6894204	10.058	LOC100691190	protein AHNAK2	B
NC_031983.2	6974793	6980075	8.049	pgrmc2	membrane-associated progesterone receptor component 2	B
NC_031983.2	6981947	6990344	8.049	LOC100692897	acetylserotonin O-methyltransferase	B
NC_031983.2	6991156	7005586	8.049	LOC100690654	protein Jade-1	B
NC_031983.2	7007189	7021874	8.049	nsd2	histone-lysine N-methyltransferase NSD2	B
NC_031983.2	7023979	7048379	8.049	letm1	leucine zipper and EF-hand containing transmembrane protein 1%2C	B
NC_031983.2	7108812	7176666	8.049	fgfr3	fibroblast growth factor receptor 3	B
NC_031983.2	7315333	7317930	8.398	LOC100692627	HRAS-like suppressor 3	B
NC_031983.2	7332472	7335092	10.094	foxi3	forkhead box protein I3	B
NC_031983.2	7369889	7371736	10.094	LOC100692083	complement factor D	B
NC_031983.2	7371398	7376514	10.094	LOC102082980	GTPase IMAP family member 8	B
NC_031983.2	7376658	7401153	10.094	lg19h20orf194	linkage group 19 C20orf194 homolog	B
NC_031983.2	7402978	7409121	10.094	ky	kyphoscoliosis peptidase	B
NC_031983.2	7413479	7513281	10.094	slc4a11	sodium bicarbonate transporter-like protein 11	B
NC_031983.2	7551046	7552523	9.932	rpia	ribose 5-phosphate isomerase A	B
NC_031983.2	7558908	7574820	7.743	eif2ak3	eukaryotic translation initiation factor 2 alpha kinase 3	B
NC_031983.2	8350364	8370547	8.034	rab3gap2	rab3 GTPase-activating protein non-catalytic subunit	B
NC_031983.2	8391694	8395345	8.034	LOC102079967	interferon alpha-inducible protein 27-like protein 2A	B
NC_031983.2	8396692	8399704	8.034	LOC106098440	interferon alpha-inducible protein 27-like protein 2A	B
NC_031983.2	8400478	8401709	8.034	LOC100690202	interferon alpha-inducible protein 27-like protein 2A	B
NC_031983.2	8402636	8404434	8.034	LOC100689935	interferon alpha-inducible protein 27-like protein 2A	B
NC_031983.2	8405892	8412076	8.034	LOC102079820	interferon alpha-inducible protein 27-like protein 1	B
NC_031983.2	8419420	8426749	8.034	LOC100712553	interferon alpha-inducible protein 27%2C mitochondrial-like	B
NC_031983.2	8425937	8429261	8.034	LOC109195965	interferon alpha-inducible protein 27-like protein 2A	B
NC_031983.2	8429852	8430872	8.034	LOC109195964	interferon alpha-inducible protein 27-like protein 2A	B
NC_031983.2	8436444	8438098	8.034	LOC109195779	interferon alpha-inducible protein 27-like protein 2	B
NC_031983.2	8438695	8439648	8.034	LOC102082289	interferon alpha-inducible protein 27-like protein 2A	B
NC_031983.2	8442357	8450566	8.034	LOC109194347	interferon alpha-inducible protein 27-like protein 2	B
NC_031983.2	8452562	8453519	8.034	LOC102079370	interferon alpha-inducible protein 27-like protein 2A	B
NC_031983.2	8460285	8469899	8.034	LOC100708436	cytosolic 5'-nucleotidase 1A	B
NC_031983.2	8480042	8483392	8.034	LOC100712021	protein L-Myc-1b	B
NC_031983.2	8493605	8505005	8.034	LOC100711753	sodium-dependent lysophosphatidylcholine symporter 1-B	B

NC_031983.2	8551599	8555072	8.034	LOC100707898	syncoilin	B
NC_031983.2	8554822	8559519	8.034	LOC100711479	histone-binding protein RBBP4	B
NC_031983.2	8559573	8565831	8.034	zbtb8os	protein archease	B
NC_031984.2	3790685	3808236	7.767	LOC109196279	plexin A3	B
NC_031984.2	3874603	3887494	7.767	LOC100698542	interferon-inducible GTPase 5	B
NC_031984.2	4013481	4018998	7.767	LOC100698012	GTPase IMAP family member 7-like	B
NC_031985.2	12602912	12611070	8.173	LOC100692985	myelin basic protein	B
NC_031985.2	12614692	12621219	8.173	LOC102080389	zinc finger and BTB domain-containing protein 45	B
NC_031985.2	12621735	12632687	8.173	scn1b	sodium channel subunit beta-1	B
NC_031985.2	12633695	12638384	8.173	naxe	NAD(P)H-hydrate epimerase	B
NC_031985.2	12639157	12648852	8.173	LOC102079654	lamin-A%2C	B
NC_031985.2	12649465	12658067	8.173	LOC100691402	upstream stimulatory factor 2	B
NC_031985.2	12658583	12665916	8.173	tekt2	tektin 2%2C	B
NC_031985.2	12666476	12669325	8.173	LOC100709165	free fatty acid receptor 3-like	B
NC_031985.2	12670703	12676680	8.173	LOC100708895	free fatty acid receptor 3-like	B
NC_031985.2	12677113	12700688	8.173	LOC100692441	protein argonaute-3	B
NC_031985.2	12705312	12724652	8.173	LOC100708625	sodium/hydrogen exchanger 3	B
NC_031985.2	12749359	12753924	8.173	LOC109196462	cytotoxic T-lymphocyte protein 4	B
NC_031985.2	12754007	12769281	8.173	LOC100692172	sodium/hydrogen exchanger 3	B
NC_031985.2	12789969	12793995	8.173	olah	S-acyl fatty acid synthase thioesterase%2C medium chain	B
NC_031985.2	12793955	12808035	8.173	LOC100708352	sodium/hydrogen exchanger 3	B
NC_031985.2	12819966	12837289	8.173	LOC100691637	sodium/hydrogen exchanger 3	B
NC_031985.2	6928883	6942827	8.016	LOC100710548	major histocompatibility complex class I-related gene protein	B
NC_031985.2	6957333	6962170	10.291	LOC100710819	major histocompatibility complex class I-related gene protein-like	B
NC_031985.2	6965332	6970852	10.291	LOC100711084	major histocompatibility complex class I-related gene protein	B
NC_031985.2	6975798	6987223	10.291	LOC100711353	major histocompatibility complex class I-related gene protein-like	B
NC_031985.2	7040251	7079158	10.291	LOC102079388	major histocompatibility complex class I-related gene protein	B
NC_031985.2	7067860	7071361	10.291	LOC109196531	zinc finger MYM-type protein 1-like	B
NC_031985.2	7096398	7099047	10.291	LOC112843547	major histocompatibility complex class I-related gene protein-like	B
NC_031985.2	7104708	7109942	10.291	LOC100696328	major histocompatibility complex class I-related gene protein-like	B
NC_031985.2	7112959	7116672	10.291	LOC109196375	major histocompatibility complex class I-related gene protein-like	B
NC_031985.2	7132431	7141722	10.291	LOC109196374	major histocompatibility complex class I-related gene protein-like	B
NC_031985.2	7153256	7165286	10.291	LOC100712435	major histocompatibility complex class I-related gene protein	B
NC_031986.2	14730155	14731258	8.417	LOC102080405	leukotriene-B(4) omega-hydroxylase 2	B
NC_031986.2	14771642	14773126	8.417	LOC112843912	zinc finger BED domain-containing protein 4-like	B
NC_031986.2	14776342	14777236	8.417	LOC112843780	OX-2 membrane glycoprotein-like	B
NC_031986.2	14785799	14788112	8.417	LOC106096586	OX-2 membrane glycoprotein%2C	B

NC_031986.2	14795994	14799175	8.417	LOC106096584	OX-2 membrane glycoprotein%2C	B
NC_031986.2	14803010	14825256	8.417	LOC100710881	OX-2 membrane glycoprotein	B
NC_031986.2	14831018	14833432	8.417	LOC106096583	B- and T-lymphocyte attenuator	B
NC_031986.2	14837887	14853424	8.417	LOC109197094	B- and T-lymphocyte attenuator	B
NC_031986.2	14854308	14860274	8.417	LOC102081783	B- and T-lymphocyte attenuator-like	B
NC_031986.2	14860437	14871074	8.417	LOC100708021	splicing factor U2AF 35 kDa subunit	B
NC_031986.2	14872701	14883131	8.417	LOC100707752	cystathionine beta-synthase	B
NC_031986.2	14907296	14926142	8.417	LOC100710616	vascular endothelial growth factor C	B
NC_031986.2	14937017	14938528	8.417	LOC100707486	trace amine-associated receptor 13c-like	B
NC_031986.2	14949624	14950601	8.417	LOC100707222	trace amine-associated receptor 13c-like	B
NC_031986.2	14958014	14958991	8.417	LOC109196918	trace amine-associated receptor 13c-like	B
NC_031986.2	14960813	14962401	8.417	LOC100706960	trace amine-associated receptor 13c-like	B
NC_031986.2	14974390	14975367	8.417	LOC100706689	trace amine-associated receptor 13c-like	B
NC_031986.2	23875032	23900870	13.082	LOC100691162	DNA repair protein RAD50	B
NC_031986.2	23907163	23939081	13.082	LOC112843900	nucleotide-binding oligomerization domain-containing protein 1-like	B
NC_031986.2	24025421	24048419	13.082	LOC100690327	prolactin receptor	B
NC_031986.2	33631911	33634328	8.386	oca2	OCA2 melanosomal transmembrane protein%2C	B
NC_031986.2	33639386	33691841	10.799	herc2	E3 ubiquitin-protein ligase HERC2	B
NC_031986.2	33691986	33698572	10.799	nipa1	NIPA magnesium transporter 1	B
NC_031986.2	33701274	33706512	10.799	nipa2	NIPA magnesium transporter 2%2C	B
NC_031986.2	33706514	33746188	10.799	cyfip1	cytoplasmic FMR1 interacting protein 1%2C	B
NC_031986.2	33714686	33719711	10.799	fndc9	fibronectin type III domain containing 9	B
NC_031986.2	33757031	33759019	10.799	LOC109197062	trypsin	B
NC_031986.2	33807180	33808607	10.799	LOC109197061	trypsin	B
NC_031986.2	33815920	33817521	10.799	LOC100703283	mast cell protease 1A	B
NC_031986.2	33818487	33823619	10.799	LOC100711131	spindlin-1	B
NC_031986.2	33822513	33826079	10.799	lg23h19orf70	MICOS complex subunit MIC13	B
NC_031986.2	33826324	33833218	10.799	LOC100703009	hydroxysteroid 11-beta-dehydrogenase 1-like protein	B
NC_031986.2	33844263	33849637	10.799	LOC100710599	hydroxysteroid 11-beta-dehydrogenase 1-like protein	B
NC_031986.2	35302429	35304427	8.497	LOC100694966	mast cell protease 4	B
NC_031986.2	35314353	35325078	8.497	LOC100694707	granzyme B(G%2CH)	B
NC_031986.2	35349034	35364900	8.497	LOC100694171	ras-related protein Rab-3B	B
NC_031986.2	35366081	35386053	8.497	LOC100693897	nardilysin	B
NC_031986.2	35415013	35430230	8.497	LOC102082277	sterile alpha motif domain-containing protein 9	B
NC_031986.2	35432564	35442833	8.497	LOC100693634	granzyme G	B
NC_031986.2	35444786	35446473	8.497	LOC102082461	mast cell protease 1A	B
NC_031986.2	35455067	35501542	8.497	LOC100693369	FRAS1-related extracellular matrix protein 1	B

NC_031987.2	24264801	24267930	7.489	LOC102078189	CD209 antigen-like protein E	B
NC_031987.2	24326456	24340948	7.489	LOC102080016	CD209 antigen-like protein A	B
NC_031987.2	24347190	24349587	7.489	LOC102075958	zinc finger MYM-type protein 1	B
NC_031987.2	24381005	24397191	7.489	LOC106097441	C-type lectin domain family 4 member E-like	B
NC_031987.2	24413966	24416310	7.489	LOC112844124	CD209 antigen-like protein A	B
NC_031987.2	24451234	24459635	7.489	LOC109197657	CD209 antigen-like protein E	B
NC_031965.2	8284950	8353727	11.355	arnt2	aryl hydrocarbon receptor nuclear translocator 2	C
NC_031965.2	8320915	8329770	10.697	LOC112842401	syncytin-A-like	C
NC_031965.2	8354683	8376556	10.697	ctxnd1	cortexin domain containing 1	C
NC_031965.2	8384336	8403332	10.697	fah	fumarylacetoacetase	C
NC_031965.2	8438905	8442626	10.697	lto1	LTO1%2C ABCE1 maturation factor	C
NC_031965.2	8508660	8512284	10.697	fgf3	fibroblast growth factor 3	C
NC_031965.2	8515727	8525847	10.697	fgf4	fibroblast growth factor 4	C
NC_031966.2	15656476	15657343	7.648	LOC106097355	Ig kappa chain V region K29-213-like	C
NC_031966.2	15664833	15667148	10.044	LOC106097356	signal-regulatory protein beta-2-like	C
NC_031966.2	15696796	15704813	10.044	LOC109203601	signal-regulatory protein beta-2-like	C
NC_031966.2	15752281	15754034	10.044	LOC100702355	twitchin-like	C
NC_031966.2	15867302	15872163	10.044	LOC100703432	T-cell surface glycoprotein CD4-like	C
NC_031967.2	11092295	11143476	7.993	nfib	nuclear factor 1 B-type	C
NC_031967.2	11153931	11160186	7.705	zdhhc21	palmitoyltransferase ZDHHC21	C
NC_031967.2	11160855	11166281	7.705	plaa	phospholipase A-2-activating protein	C
NC_031967.2	11167346	11175697	7.705	haus6	HAUS augmin like complex subunit 6	C
NC_031967.2	11173623	11173753	7.705	LOC112846595	small Cajal body-specific RNA 8	C
NC_031967.2	11177143	11217449	7.705	dennd4c	DENN domain containing 4C%2C	C
NC_031967.2	11262788	11265268	7.705	LOC109195697	borealin-2	C
NC_031967.2	11265858	11269498	7.705	plin2	perilipin 2	C
NC_031967.2	11282861	11342295	7.993	adamtsl1	ADAMTS like 1%2C	C
NC_031967.2	21001975	21251975	9.165	LOC109198185	low affinity immunoglobulin gamma Fc region receptor II-b	C
NC_031967.2	21001975	21251975	9.165	LOC109201287	obscurin	C
NC_031967.2	21085069	21102494	9.165	LOC109196517	peroxidasin homolog	C
NC_031967.2	21142593	21153220	9.165	LOC109201316	high affinity immunoglobulin epsilon receptor subunit alpha-like	C
NC_031967.2	29710591	29716904	13.307	LOC109194277	E3 ubiquitin-protein ligase TRIM39-like	C
NC_031967.2	29732366	29734299	13.307	LOC109199711	erythroid membrane-associated protein	C
NC_031967.2	29793262	29800581	13.307	LOC112843099	ribonuclease inhibitor-like	C
NC_031967.2	29804348	29809428	13.307	LOC102082638	E3 ubiquitin-protein ligase TRIM39	C

NC_031967.2	29830128	29834194	13.307	LOC100695258	CMRF35-like molecule 9	C
NC_031967.2	29861829	29944100	16.955	LOC102079294	polymeric immunoglobulin receptor	C
NC_031967.2	29902613	29907759	13.307	LOC109197601	nuclear factor 7%2C ovary-like	C
NC_031967.2	29942615	29944100	16.955	LOC112843390	polymeric immunoglobulin receptor-like	C
NC_031967.2	38541326	38574710	14.574	LOC100706439	kinesin-like protein KIF1C	C
NC_031967.2	38580689	38603752	14.574	LOC100705911	cysteinyl leukotriene receptor 1-like	C
NC_031967.2	38621206	38625268	14.574	LOC102075710	myelin-oligodendrocyte glycoprotein	C
NC_031967.2	38644422	38704304	14.574	LOC102076476	ankyrin repeat domain-containing protein 46	C
NC_031967.2	43637548	43648417	12.264	LOC102076758	butyrophilin-like protein 8	C
NC_031967.2	43650400	43657050	12.264	LOC100696502	butyrophilin subfamily 2 member A2	C
NC_031967.2	43666551	43676574	12.264	LOC100695187	aggrecan core protein	C
NC_031967.2	43690497	43692491	12.264	LOC100696769	ladderlectin	C
NC_031967.2	43695767	43705821	12.264	LOC102077851	B-cell receptor CD22	C
NC_031967.2	43714027	43717786	12.264	LOC109203684	zinc finger MYM-type protein 1-like	C
NC_031967.2	43718486	43722508	12.264	LOC109194332	zinc finger MYM-type protein 1-like	C
NC_031967.2	43772297	43788568	12.264	LOC112845607	lamin-A-like	C
NC_031967.2	4563350	4617887	7.547	sorcs2	VPS10 domain-containing receptor SorCS2	C
NC_031967.2	4634427	4648704	10.230	grpel1	GrpE like 1%2C mitochondrial	C
NC_031967.2	4649851	4656112	10.230	tada2b	transcriptional adapter 2-beta	C
NC_031967.2	4656276	4661124	10.230	LOC102079040	myosin-4	C
NC_031967.2	4689521	4690958	10.230	LOC100704474	eotaxin	C
NC_031967.2	4697137	4698163	10.230	LOC100704742	C-C motif chemokine 3	C
NC_031967.2	4699386	4742223	10.230	tbc1d14	TBC1 domain family member 14	C
NC_031967.2	4746606	4759672	10.230	kiaa0232	KIAA0232 ortholog%2C	C
NC_031967.2	4776120	4776947	10.230	LOC106096483	protein S100-P	C
NC_031967.2	4777380	4778338	10.230	LOC100705545	protein S100-G	C
NC_031967.2	53707615	53711900	7.717	LOC109201334	zinc finger MYM-type protein 1	C
NC_031967.2	53808345	53810754	7.717	LOC109201337	tripartite motif-containing protein 16-like	C
NC_031967.2	53866123	53874374	7.717	LOC102082344	retrovirus-related Pol polyprotein from transposon 412%2C	C
NC_031967.2	53877225	53950329	8.972	LOC109201351	low affinity immunoglobulin gamma Fc region receptor II	C
NC_031967.2	53905553	53920952	7.717	LOC112845985	zinc finger protein 883-like	C
NC_031967.2	53915858	53931177	7.717	LOC109201348	low affinity immunoglobulin gamma Fc region receptor II	C
NC_031967.2	54159054	54202935	11.607	LOC106097159	low affinity immunoglobulin gamma Fc region receptor II	C
NC_031967.2	54251264	54258518	11.607	LOC102081459	butyrophilin subfamily 2 member A2%2C	C

NC_031967.2	54277348	54285214	11.607	LOC109196578	G2/M phase-specific E3 ubiquitin-protein ligase-like	C
NC_031967.2	54365500	54366701	11.607	LOC112844103	putative nuclease HARBI1	C
NC_031967.2	55308862	55313331	16.955	LOC100689971	butyrophilin subfamily 3 member A2	C
NC_031967.2	55379911	55394599	16.955	LOC102079944	phospholipid transfer protein	C
NC_031967.2	55428369	55430194	16.955	LOC102079855	BPI fold-containing family C protein-like	C
NC_031967.2	59627941	59649297	10.181	LOC109201331	programmed cell death 1 ligand 1-like	C
NC_031967.2	59663066	59672498	10.181	LOC102081053	interferon-induced protein with tetratricopeptide repeats 5	C
NC_031967.2	59680409	59685902	10.181	LOC100702506	interferon-induced protein with tetratricopeptide repeats 5	C
NC_031967.2	59744528	59753934	10.181	LOC109194473	interferon-induced protein with tetratricopeptide repeats 1	C
NC_031967.2	59761387	59765743	10.181	LOC102081141	butyrophilin-like protein 8	C
NC_031967.2	59776051	59806319	13.584	LOC102081234	interferon-induced protein with tetratricopeptide repeats 5	C
NC_031967.2	59800083	59806319	13.584	LOC109194232	interferon-induced protein with tetratricopeptide repeats 1	C
NC_031967.2	71826216	71849543	7.838	LOC100696307	ephrin type-B receptor 4	C
NC_031967.2	71908519	71915925	7.838	LOC102079713	low affinity immunoglobulin gamma Fc region receptor II-like	C
NC_031967.2	71977396	71978581	7.838	LOC109201475	zinc finger BED domain-containing protein 1-like	C
NC_031967.2	72015919	72021730	7.838	LOC109196951	low affinity immunoglobulin gamma Fc region receptor II-like	C
NC_031967.2	72187657	72191728	8.824	LOC112846013	NLR family CARD domain-containing protein 3-like	C
NC_031967.2	72210094	72265658	8.824	LOC102076415	butyrophilin-like protein 2	C
NC_031967.2	72279632	72353138	8.824	LOC109196955	polymeric immunoglobulin receptor-like	C
NC_031967.2	77312361	77315916	10.251	LOC102076883	programmed cell death 1 ligand 1	C
NC_031967.2	77328243	77333043	10.251	LOC100700236	matrix remodeling-associated protein 8-like	C
NC_031967.2	77334805	77337967	10.251	LOC102076784	CD226 antigen-like	C
NC_031967.2	77349617	77356941	10.251	LOC102075832	coxsackievirus and adenovirus receptor homolog	C
NC_031967.2	77504011	77508044	10.251	LOC112843799	protein translocase subunit SecA-like	C
NC_031969.2	15672960	15872654	10.309	LOC106096470	immunoglobulin mu heavy chain-like	C
NC_031969.2	15686289	15692772	10.309	LOC106096474	Ig mu chain C region membrane-bound form-like	C
NC_031969.2	15774379	15778031	10.309	LOC109202053	Ig gamma-2B chain C region-like	C
NC_031969.2	15815743	15825355	10.309	LOC100700584	G2/M phase-specific E3 ubiquitin-protein ligase	C
NC_031969.2	15910562	15922960	10.309	LOC100693985	nucleus accumbens-associated protein 1	C
NC_031970.2	20683800	20694519	11.337	LOC100694025	voltage-dependent calcium channel subunit alpha-2/delta-3	C
NC_031970.2	23828959	23831213	7.889	LOC100703692	class E basic helix-loop-helix protein 40	C
NC_031970.2	23834724	23909251	7.889	LOC100704142	inositol 1%2C4%2C5-trisphosphate receptor type 1	C
NC_031970.2	23909540	23918054	10.877	sumf1	formylglycine-generating enzyme	C
NC_031970.2	23918351	23928557	10.877	LOC100704594	leucine-rich repeat neuronal protein 1	C

NC_031970.2	23957536	23964205	10.877	LOC100705573	sorting nexin-6	C
NC_031970.2	23964385	23966265	10.877	LOC100704861	dnaJ homolog subfamily B member 9	C
NC_031970.2	23966328	23970358	10.877	zc3h10	zinc finger CCCH domain-containing protein 10	C
NC_031970.2	23972101	23990139	10.877	LOC100706099	extended synaptotagmin-1	C
NC_031970.2	23994749	24008490	10.877	LOC100706363	probable E3 ubiquitin-protein ligase bre1	C
NC_031970.2	24024296	24078959	7.889	LOC100706902	low-density lipoprotein receptor-related protein 1	C
NC_031970.2	24045497	24049008	10.877	LOC102075530	delta-type opioid receptor-like	C
NC_031970.2	24135307	24153721	7.648	LOC100707164	RNA-binding motif%2C single-stranded-interacting protein 2	C
NC_031970.2	24165978	24169656	7.612	LOC100707431	protein lifeguard 2	C
NC_031970.2	24174918	24176622	7.624	LOC100707699	nuclear receptor subfamily 1 group D member 1	C
NC_031970.2	30345029	30359314	8.362	dnah1	dynein axonemal heavy chain 1	C
NC_031970.2	30359990	30375402	7.676	mapkapk3	MAP kinase-activated protein kinase 3	C
NC_031970.2	30378720	30394363	7.676	LOC100703249	6-phosphofructo-2-kinase/fructose-2%2C6-bisphosphatase 4	C
NC_031970.2	30395512	30398106	7.676	LOC100702706	actin-related protein 2/3 complex subunit 4	C
NC_031970.2	30398387	30402214	7.676	tada3	transcriptional adapter 3	C
NC_031970.2	30415773	30425204	7.676	LOC100702167	RNA-binding protein 38	C
NC_031970.2	30426687	30433681	7.676	rae1	mRNA export factor	C
NC_031970.2	30435393	30444568	7.676	LOC100701625	bone morphogenetic protein 7	C
NC_031970.2	30453238	30466697	7.676	LOC102076859	dysbindin	C
NC_031970.2	30474511	30483754	7.676	LOC100702977	cadherin-like protein 26	C
NC_031970.2	30487388	30495124	7.676	LOC102075564	cadherin-like protein 26	C
NC_031970.2	30505787	30514088	7.676	LOC100701354	cadherin-like protein 26	C
NC_031970.2	30514202	30516266	7.676	LOC100702707	protein LSM14 homolog B	C
NC_031970.2	30518436	30531793	7.676	LOC102076274	transcription initiation factor TFIID subunit 4	C
NC_031970.2	30540744	30595029	8.362	LOC100702438	cadherin-4	C
NC_031971.2	16284398	16288794	7.666	lsm6	LSM6 homolog%2C U6 small nuclear RNA and mRNA degradation associated%2C	C
NC_031971.2	16303566	16378150	7.666	znf827	zinc finger protein 827	C
NC_031971.2	16395124	16408062	7.666	mmaa	methylmalonic aciduria (cobalamin deficiency) cblA type%2C	C
NC_031971.2	16410051	16428025	7.666	smad1	SMAD family member 1	C
NC_031971.2	16447484	16458554	7.666	LOC102076376	nephronectin	C
NC_031971.2	16475602	16504914	7.666	hhip	hedgehog interacting protein%2C	C
NC_031971.2	24647663	24650368	10.576	keap1	kelch like ECH associated protein 1	C
NC_031971.2	24653604	24656929	10.154	LOC100712110	sphingosine 1-phosphate receptor 1	C
NC_031971.2	24690091	24692954	10.154	fdx2	ferredoxin 2	C

NC_031971.2	24693173	24700238	10.154	zglp1	GATA-type zinc finger protein 1	C
NC_031971.2	24701513	24702708	10.154	LOC100712378	forkhead box protein D3-B	C
NC_031971.2	24705964	24707535	10.154	LOC100689757	sialidase-4	C
NC_031971.2	24712272	24718019	10.154	LOC106098442	sialidase-3-like	C
NC_031971.2	24716370	24728467	10.154	LOC100690027	sialidase-4-like	C
NC_031971.2	24730466	24736543	10.154	LOC102075532	ubiquitin carboxyl-terminal hydrolase 26	C
NC_031971.2	24743047	24744293	10.154	LOC109202509	ubiquitin carboxyl-terminal hydrolase 29-like	C
NC_031971.2	24744289	24752350	10.154	LOC109202437	ubiquitin carboxyl-terminal hydrolase 37-like	C
NC_031971.2	24756056	24761745	10.154	LOC102080914	ubiquitin carboxyl-terminal hydrolase 26-like	C
NC_031971.2	24763764	24797318	10.154	LOC102075878	ubiquitin carboxyl-terminal hydrolase 26	C
NC_031971.2	24765881	24771109	10.154	LOC109202416	ubiquitin carboxyl-terminal hydrolase 26-like	C
NC_031971.2	24774095	24776729	10.154	neu3e	sialidase-3-like	C
NC_031971.2	24801817	24806700	10.154	LOC112847074	ubiquitin carboxyl-terminal hydrolase 37-like	C
NC_031971.2	24817468	24824167	10.154	LOC100700016	T-cell acute lymphocytic leukemia protein 1 homolog	C
NC_031971.2	24842065	24844185	10.154	LOC100700287	heme oxygenase	C
NC_031971.2	24845951	24850133	10.154	LOC100700565	RING finger protein 11	C
NC_031971.2	24851195	24856911	10.154	LOC100700831	PWWP domain-containing protein 2A	C
NC_031971.2	24858136	24897663	10.576	LOC100690838	nuclear factor 1 X-type	C
NC_031971.2	31689470	31713471	10.324	LOC100691651	complement C3	C
NC_031971.2	31716141	31747992	10.324	LOC100691917	complement C3	C
NC_031971.2	31751455	31773251	10.324	LOC100700974	complement C3	C
NC_031971.2	31752187	31800704	10.324	LOC100692186	complement C3	C
NC_031971.2	31805495	31820232	10.324	LOC100692725	DENN domain-containing protein 1B	C
NC_031971.2	31820970	31824330	10.324	LOC100703003	tubulin beta-4B chain	C
NC_031971.2	31851458	31901547	12.003	LOC100692999	glucagon receptor	C
NC_031971.2	6274425	6283320	7.962	LOC100707118	hsp70-Hsp90 organizing protein 2	C
NC_031971.2	6291028	6334825	7.962	LOC102079796	B-cell receptor CD22	C
NC_031971.2	6337671	6413297	7.962	LOC102083017	B-cell receptor CD22-like	C
NC_031971.2	6418813	6429347	7.962	LOC102080295	B-cell receptor CD22	C
NC_031971.2	6440886	6493903	7.962	LOC100707654	zinc finger protein 665%2C	C
NC_031971.2	6487217	6491295	7.962	LOC112847099	G2/M phase-specific E3 ubiquitin-protein ligase-like	C
NC_031971.2	6522793	6524425	7.962	LOC109202532	hemicentin-1-like	C
NC_031972.2	35058365	35062848	8.471	slc25a46	solute carrier family 25 member 46	C
NC_031972.2	35062662	35067633	7.748	LOC100703784	protein asteroid homolog 1-like	C

NC_031972.2	35090963	35095997	7.748	LOC109202854	protein asteroid homolog 1-like	C
NC_031972.2	35099004	35105408	7.748	LOC100703239	protein asteroid homolog 1-like	C
NC_031972.2	35118664	35148326	7.748	LOC100702966	cell division control protein 3	C
NC_031972.2	35120086	35120195	7.748	LOC112847600	U6 spliceosomal RNA	C
NC_031972.2	35133692	35136284	7.748	LOC112847250	proline-rich protein 36-like	C
NC_031972.2	35223501	35233072	7.748	LOC102076965	protein asteroid homolog 1	C
NC_031972.2	35259317	35268837	7.748	wdr36	WD repeat domain 36	C
NC_031972.2	35270436	35289502	7.748	LOC102081444	alpha-mannosidase 2	C
NC_031972.2	36746111	36750328	10.424	ppp1r3b	protein phosphatase 1 regulatory subunit 3B	C
NC_031972.2	36768087	36783371	10.424	LOC100700854	NACHT%2C LRR and PYD domains-containing protein 3-like	C
NC_031972.2	36843770	36984060	10.424	LOC102081282	scavenger receptor cysteine-rich type 1 protein M160	C
NC_031972.2	36882401	36886139	10.424	LOC102076350	scavenger receptor cysteine-rich type 1 protein M130	C
NC_031972.2	45612776	45625859	8.070	LOC102077728	collectin-11	C
NC_031972.2	45625946	45644978	8.070	LOC112847533	pulmonary surfactant-associated protein D-like	C
NC_031972.2	45649945	45663937	8.070	LOC109202924	pulmonary surfactant-associated protein D-like	C
NC_031972.2	45678820	45690907	8.070	LOC100690010	collectin-43	C
NC_031972.2	45699095	45701821	8.070	LOC109194373	mannose-binding protein C	C
NC_031972.2	45704228	45707226	8.070	LOC109202923	mannose-binding protein C	C
NC_031972.2	45727348	45729873	8.070	LOC106098139	mannose-binding protein C	C
NC_031972.2	45731881	45734030	8.070	LOC102077308	mannose-binding protein C	C
NC_031972.2	45742547	45745482	8.070	ifitm5	interferon induced transmembrane protein 5	C
NC_031972.2	60529872	60531291	8.015	mterf2	mitochondrial transcription termination factor 2	C
NC_031972.2	60535544	60541199	8.015	LOC100703984	dihydrofolate reductase	C
NC_031972.2	60541345	60557162	8.015	LOC100703716	cryptochrome-1	C
NC_031972.2	60561843	60623977	8.015	LOC100703440	ankyrin repeat and BTB/POZ domain-containing protein BTBD11-B	C
NC_031972.2	60626777	60634848	8.015	mlc1	megalencephalic leukoencephalopathy with subcortical cysts 1%2C	C
NC_031972.2	60636150	60643975	8.015	panx2	pannexin 2	C
NC_031972.2	60644264	60651377	8.015	trabd	TraB domain containing	C
NC_031972.2	60652010	60660183	8.015	selenoo	selenoprotein O	C
NC_031972.2	60660922	60669063	8.015	LOC100702632	mitogen-activated protein kinase 12	C
NC_031972.2	60677801	60701136	8.015	LOC100697954	MTSS1-like protein	C
NC_031972.2	60705347	60779872	8.015	LOC106098375	neural-cadherin	C
NC_031972.2	61028407	61034850	7.801	parp12	poly [ADP-ribose] polymerase 12	C
NC_031972.2	61034356	61044776	7.801	LOC100695569	thromboxane-A synthase	C

NC_031972.2	61045377	61053350	7.497	LOC102078896	thromboxane-A synthase	C
NC_031972.2	61053743	61059938	7.497	LOC100695304	thromboxane-A synthase	C
NC_031972.2	61123542	61190264	11.156	LOC100701827	homeodomain-interacting protein kinase 2	C
NC_031972.2	61191488	61229459	10.925	LOC102079498	UPF0606 protein KIAA1549	C
NC_031972.2	61229592	61275777	8.042	LOC100695036	protein-methionine sulfoxide oxidase mical3a	C
NC_031972.2	61308824	61318760	10.315	ttc38	tetratricopeptide repeat domain 38	C
NC_031972.2	61318569	61331138	9.809	LOC100694777	tetraspanin-33-like	C
NC_031972.2	61335640	61355288	8.200	LOC100701010	tetraspanin-9	C
NC_031972.2	61558831	61571128	8.404	LOC100700740	protein arginine N-methyltransferase 8-B	C
NC_031972.2	61585963	61586130	7.945	LOC100694510	ras and EF-hand domain-containing protein homolog	C
NC_031972.2	61646174	61683405	7.783	LOC100694244	transmembrane and TPR repeat-containing protein 2	C
NC_031972.2	61785236	61788481	10.418	LOC100693969	protein FAM180A	C
NC_031972.2	61788462	61793491	8.061	LOC100700474	solute carrier family 23 member 1	C
NC_031972.2	61802302	61803444	8.065	LOC102080922	intestine-specific homeobox	C
NC_031972.2	61806444	61816944	8.067	LOC100693700	protein bicaudal D homolog 1	C
NC_031972.2	61844067	61844545	7.851	LOC100693441	FYVE%2C RhoGEF and PH domain-containing protein 4	C
NC_031972.2	61889819	61905170	7.499	nup205	nuclear pore complex protein Nup205	C
NC_031972.2	61905402	61912198	7.499	agk	acylglycerol kinase	C
NC_031972.2	61912679	61916777	8.523	LOC100692895	protein LCHN	C
NC_031972.2	61918570	61923081	7.499	LOC100692625	wee1-like protein kinase 2	C
NC_031972.2	61926215	61939407	7.499	LOC100692352	bcl-2-like protein 13	C
NC_031972.2	62108063	62114605	7.497	LOC100690199	probable polypeptide N-acetylgalactosaminyltransferase 8	C
NC_031972.2	62119357	62123362	7.497	LOC102075693	probable polypeptide N-acetylgalactosaminyltransferase 8	C
NC_031972.2	62145475	62152339	7.497	LOC100689932	probable polypeptide N-acetylgalactosaminyltransferase 8	C
NC_031972.2	62156895	62163221	7.497	LOC100712550	probable polypeptide N-acetylgalactosaminyltransferase 8	C
NC_031972.2	62163556	62179593	7.497	LOC100712284	probable polypeptide N-acetylgalactosaminyltransferase 8	C
NC_031972.2	62186116	62192336	7.497	ndufa9	NADH dehydrogenase [ubiquinone] 1 alpha subcomplex subunit 9%2C mitochondrial	C
NC_031972.2	62192872	62216747	7.497	dyrk4	dual specificity tyrosine phosphorylation regulated kinase 4%2C	C
NC_031972.2	62217876	62228936	7.497	wfdc1	WAP four-disulfide core domain 1%2C	C
NC_031972.2	62247095	62262864	7.497	LOC100711474	monocarboxylate transporter 12	C
NC_031972.2	62278196	62302716	7.497	kcng4	potassium voltage-gated channel modifier subfamily G member 4%2C	C
NC_031972.2	62539319	62731079	7.536	cdh13	cadherin 13%2C	C
NC_031972.2	62707377	62711100	10.262	LOC112847384	THAP domain-containing protein 5-like	C
NC_031972.2	62991020	62998385	8.003	LOC100698323	G1/S-specific cyclin-D2	C

NC_031974.2	18322564	18365708	8.212	vipr1	vasoactive intestinal peptide receptor 1	C
NC_031974.2	18401604	18413372	8.212	LOC100709670	eukaryotic translation initiation factor 5B	C
NC_031974.2	18425120	18426275	8.212	LOC100708859	nanos homolog 1-like	C
NC_031974.2	18427405	18434877	8.212	kpna1	importin subunit alpha-5	C
NC_031974.2	18439336	18442908	8.212	LOC100698117	protein FAM162B	C
NC_031974.2	18443169	18447831	8.212	ccdc58	coiled-coil domain containing 58%2C	C
NC_031974.2	18451568	18452409	8.212	csta	cystatin A	C
NC_031974.2	18456939	18457778	8.212	LOC100697579	cystatin-B-like	C
NC_031974.2	18472303	18474121	8.212	LOC100697316	cystatin-B-like	C
NC_031974.2	18489822	18492141	8.212	LOC109203484	trichohyalin-like	C
NC_031974.2	18493855	18497890	8.212	LOC109203569	extracellular matrix protein FRAS1	C
NC_031974.2	18497803	18500585	8.212	LOC106097837	cystatin-B	C
NC_031974.2	18501318	18503115	8.212	LOC100690818	cystatin-B	C
NC_031974.2	18503258	18511279	8.212	LOC109203571	cystatin-B	C
NC_031974.2	18512185	18515908	8.212	LOC109203572	cystatin-B	C
NC_031974.2	18519150	18522221	8.212	LOC109203567	trichohyalin-like	C
NC_031974.2	18522878	18572564	8.212	LOC102082737	extracellular matrix protein FRAS1	C
NC_031974.2	18532113	18535014	8.212	LOC100710659	cystatin-B	C
NC_031974.2	18541778	18543040	8.212	LOC100695024	cystatin-B	C
NC_031974.2	18557922	18563668	8.212	LOC100696082	thiosulfate:glutathione sulfurtransferase	C
NC_031974.2	4096784	4097929	10.364	LOC106097626	GTPase IMAP family member 7-like	C
NC_031974.2	4099092	4099980	10.364	LOC100695536	GTPase IMAP family member 4-like	C
NC_031974.2	4119801	4121623	10.364	LOC100708574	GTPase IMAP family member 3-like	C
NC_031974.2	4125809	4127152	10.364	LOC100708304	GTPase IMAP family member 7-like	C
NC_031974.2	4139808	4141494	10.364	LOC100708033	immune-associated nucleotide-binding protein 9-like	C
NC_031974.2	4141562	4142847	10.364	LOC100707765	GTPase IMAP family member 7-like	C
NC_031974.2	4149979	4151990	10.364	LOC100703489	GTPase IMAP family member 7-like	C
NC_031974.2	4163611	4167644	10.364	LOC100707496	GTPase IMAP family member 6	C
NC_031974.2	4169892	4171248	10.364	LOC100707234	GTPase IMAP family member 7-like	C
NC_031974.2	4196064	4220909	10.364	LOC102077850	GTPase IMAP family member 7-like	C
NC_031974.2	4201171	4203073	10.364	LOC100706432	GTPase IMAP family member 7-like	C
NC_031974.2	4209110	4213348	10.364	LOC100706173	GTPase IMAP family member 3	C
NC_031974.2	4219331	4220604	10.364	LOC100705904	GTPase IMAP family member 7-like	C
NC_031974.2	4220949	4222412	10.364	LOC112841629	GTPase IMAP family member 7-like	C

NC_031974.2	4221001	4295785	10.364	LOC100709743	GTPase IMAP family member 7	C
NC_031974.2	4240074	4250626	10.364	LOC100705375	GTPase IMAP family member 8-like	C
NC_031974.2	4265851	4270271	10.364	LOC109197604	NACHT%2C LRR and PYD domains-containing protein 1-like	C
NC_031974.2	4284822	4289986	10.364	LOC109197603	NACHT%2C LRR and PYD domains-containing protein 1b allele 3-like	C
NC_031974.2	4290013	4292085	10.364	LOC106097025	GTPase IMAP family member 7-like	C
NC_031974.2	7528517	7599969	7.688	LOC100707500	disco-interacting protein 2 homolog C	C
NC_031974.2	7603048	7651893	10.205	LOC100707237	vasoactive intestinal polypeptide receptor 2	C
NC_031974.2	7656264	7671129	10.205	LOC112847889	zinc finger protein 726-like	C
NC_031974.2	7672915	7675091	10.205	LOC102080242	vegetative cell wall protein gp1	C
NC_031974.2	7745546	7762222	10.205	LOC112847955	zinc finger protein 271-like	C
NC_031974.2	7762268	7767304	10.205	LOC112847891	putative nuclease HARBI1	C
NC_031976.2	16738840	16747294	8.988	LOC102076445	myelin-associated glycoprotein	C
NC_031976.2	16754897	16759214	8.988	LOC109194180	sialoadhesin	C
NC_031976.2	16825654	16830812	8.988	LOC100703576	C3a anaphylatoxin chemotactic receptor-like	C
NC_031976.2	16837460	16838273	8.988	LOC100703305	chemokine-like receptor 1	C
NC_031976.2	16842937	16850336	8.988	LOC100703034	chemokine-like receptor 1	C
NC_031976.2	16868614	16872465	8.988	LOC100702766	C5a anaphylatoxin chemotactic receptor 1-like	C
NC_031976.2	16917267	16931359	8.988	LOC100709210	chemokine-like receptor 1	C
NC_031976.2	16933306	16940496	8.988	LOC100708939	chemokine-like receptor 1	C
NC_031976.2	26194413	26223508	10.121	LOC100701297	forkhead box protein O1-B	C
NC_031976.2	26228196	26231943	10.121	med18	mediator complex subunit 18%2C	C
NC_031976.2	26238088	26244657	10.121	LOC100694213	cytochrome P450 4B1	C
NC_031976.2	26256248	26262381	10.121	LOC102079868	cytochrome P450 4B1	C
NC_031976.2	26273148	26278243	10.121	LOC100701569	cytochrome P450 4B1	C
NC_031976.2	26281802	26289158	10.121	ppp1r8	nuclear inhibitor of protein phosphatase 1	C
NC_031976.2	26288234	26288364	10.121	LOC112848293	small Cajal body-specific RNA 1	C
NC_031976.2	26290272	26296441	10.121	themis2	protein THEMIS2	C
NC_031976.2	26296849	26303380	10.121	rpa2	replication protein A 32 kDa subunit	C
NC_031976.2	26304186	26308226	10.121	smpdl3b	acid sphingomyelinase-like phosphodiesterase 3b	C
NC_031976.2	26307980	26311448	10.121	mecr	enoyl-[acyl-carrier-protein] reductase%2C mitochondrial	C
NC_031976.2	26312593	26327048	10.121	LOC100692547	protein lin-28 homolog A-like	C
NC_031976.2	26327679	26340000	10.121	maneal	glycoprotein endo-alpha-1%2C2-mannosidase-like protein	C
NC_031976.2	26339890	26344759	10.121	yrdc	yrdC N6-threonylcarbamoyltransferase domain containing	C
NC_031976.2	26350314	26363755	10.121	mtf1	metal regulatory transcription factor 1	C

NC_031976.2	26363894	26383847	10.121	inpp5b	inositol polyphosphate-5-phosphatase B%2C	C
NC_031976.2	26388815	26391667	11.150	LOC102083321	probable cyclin-dependent serine/threonine-protein kinase DDB_G0292550	C
NC_031977.2	35912770	35919707	8.422	LOC100703707	fructose-1%2C6-bisphosphatase isozyme 2	C
NC_031977.2	35920443	35924647	7.735	LOC100703974	cathepsin L1	C
NC_031977.2	35934494	36013715	7.735	dapk1	death associated protein kinase 1%2C	C
NC_031977.2	36051868	36056303	7.735	LOC100700363	granzyme K	C
NC_031977.2	36078534	36082239	7.735	LOC100700638	granzyme K-like	C
NC_031977.2	36100428	36103106	7.735	LOC100700901	granzyme K	C
NC_031977.2	36111883	36116613	7.735	LOC100701180	granzyme K-like	C
NC_031977.2	36133356	36135690	7.735	LOC106098174	granzyme K-like	C
NC_031977.2	4299745	4446883	8.023	abca2	ATP binding cassette subfamily A member 2%2C	C
NC_031977.2	4525949	4527216	8.023	LOC100697646	3-hydroxy-3-methylglutaryl-coenzyme A reductase	C
NC_031978.2	1586557	1621509	8.969	LOC100709102	echinoderm microtubule-associated protein-like 6	C
NC_031978.2	1624658	1657934	8.826	LOC100710178	reticulon-1	C
NC_031978.2	1691756	1703212	8.826	LOC106096524	deleted in malignant brain tumors 1 protein	C
NC_031978.2	1725702	1736520	8.826	LOC102080493	scavenger receptor cysteine-rich type 1 protein M130	C
NC_031978.2	1794844	1826048	8.826	LOC109194525	inactive ubiquitin carboxyl-terminal hydrolase 54	C
NC_031978.2	1826019	1828807	8.826	LOC100710888	extensin	C
NC_031978.2	1830199	1836557	8.969	mmrn2	multimerin 2%2C	C
NC_031978.2	23343164	23356407	8.204	LOC100691069	visual pigment-like receptor peropsin	C
NC_031978.2	23357635	23378227	7.718	vit	vitrin	C
NC_031978.2	23379657	23405601	7.718	strn	striatin	C
NC_031978.2	23405773	23425690	7.718	heatr5b	HEAT repeat containing 5B%2C	C
NC_031978.2	23425798	23428493	7.718	gpatch11	G patch domain-containing protein 11	C
NC_031978.2	23429395	23437767	7.718	LOC100691607	interferon-induced%2C double-stranded RNA-activated protein kinase	C
NC_031978.2	23438185	23443713	7.718	LOC100700343	interferon-induced%2C double-stranded RNA-activated protein kinase	C
NC_031978.2	23497879	23526891	7.718	LOC100692960	interferon-induced%2C double-stranded RNA-activated protein kinase	C
NC_031978.2	34311726	34326714	11.164	LOC100697919	blastomere cadherin	C
NC_031979.2	12349255	12352338	7.746	LOC100702079	rhodopsin kinase 2	C
NC_031979.2	12353257	12354340	7.418	rnf7	RING-box protein 2	C
NC_031979.2	12354330	12371054	7.418	LOC100701807	transmembrane protein 255B	C
NC_031979.2	12372624	12405028	7.418	rasa2	RAS p21 protein activator 2%2C	C
NC_031979.2	12407051	12417471	7.418	zbtb38	zinc finger and BTB domain containing 38%2C	C
NC_031979.2	12418173	12421440	7.418	LOC100694132	F-box only protein 36	C

NC_031979.2	12420426	12446658	7.418	trip12	E3 ubiquitin-protein ligase TRIP12	C
NC_031979.2	12449919	12517825	7.418	dner	delta and Notch-like epidermal growth factor-related receptor	C
NC_031979.2	12521343	12553786	7.418	pid1	PTB-containing%2C cubilin and LRP1-interacting protein	C
NC_031979.2	12640658	12648031	8.270	sphkap	A-kinase anchor protein SPHKAP	C
NC_031979.2	14454870	14458890	7.661	tubd1	tubulin delta 1	C
NC_031979.2	14459685	14480310	7.661	LOC100691442	vacuole membrane protein 1	C
NC_031979.2	14480992	14491504	7.661	LOC100694582	protein NPAT	C
NC_031979.2	14491768	14517182	7.661	atm	ATM serine/threonine kinase	C
NC_031979.2	14517384	14527007	7.661	LOC102076746	transmembrane protease serine 2	C
NC_031979.2	14526937	14539415	7.661	LOC100694039	beta-secretase 2	C
NC_031979.2	14558996	14684313	7.661	LOC100693769	Down syndrome cell adhesion molecule	C
NC_031979.2	14700320	14704848	7.661	pcp4	Purkinje cell protein 4	C
NC_031980.2	18919673	19021848	7.958	man1a1	mannosidase alpha class 1A member 1	C
NC_031980.2	19026774	19029524	7.958	LOC112842378	uveal autoantigen with coiled-coil domains and ankyrin repeats-like	C
NC_031980.2	19079227	19082137	7.958	LOC112842351	macrophage mannose receptor 1-like	C
NC_031980.2	19088733	19094219	7.958	LOC102077903	macrophage mannose receptor 1	C
NC_031980.2	19107524	19110034	7.958	LOC112842495	protein ANTAGONIST OF LIKE HETEROCHROMATIN PROTEIN 1-like	C
NC_031980.2	19110575	19113443	7.958	LOC102078167	putative C-type lectin domain family 20 member A	C
NC_031980.2	19139773	19143664	7.958	ccdc25	coiled-coil domain containing 25%2C	C
NC_031980.2	19143887	19152727	7.958	esco2	N-acetyltransferase ESCO2	C
NC_031980.2	19154497	19161645	7.958	pbk	PDZ binding kinase	C
NC_031980.2	20624040	20629574	7.430	LOC102077771	proline-rich nuclear receptor coactivator 2	C
NC_031980.2	20631423	20642395	7.430	LOC100704743	cysteine-rich protein 2	C
NC_031980.2	20677007	20680976	7.430	LOC109194904	location of vulva defective 1-like	C
NC_031980.2	20707900	20711705	7.430	LOC109194905	integumentary mucin C.1-like	C
NC_031980.2	20749294	20752431	7.430	LOC102077608	cell wall integrity and stress response component 3	C
NC_031980.2	23231187	23242201	7.831	LOC100706214	paired box protein Pax-1	C
NC_031980.2	23250367	23252630	7.831	LOC100706476	homeobox protein Nkx-2.2a	C
NC_031980.2	23271591	23275668	7.831	nkx2-4	NK2 homeobox 4%2C	C
NC_031980.2	23275444	23320453	7.831	xrn2	5'-3' exoribonuclease 2	C
NC_031980.2	23334318	23340408	7.831	LOC106098344	bifunctional protein GlmU	C
NC_031980.2	23391068	23395075	10.544	insm1	INSM transcriptional repressor 1	C
NC_031980.2	23396087	23436403	10.544	cfap61	cilia and flagella associated protein 61%2C	C
NC_031980.2	23436669	23444771	10.544	crnkl1	crooked neck pre-mRNA splicing factor 1	C

NC_031980.2	23444710	23448046	10.544	naa20	N(alpha)-acetyltransferase 20%2C NatB catalytic subunit	C
NC_031980.2	23454734	23456225	7.831	LOC100707543	collagen alpha-1(XII) chain	C
NC_031980.2	27831308	27900477	7.531	LOC100710023	opsin-5	C
NC_031980.2	37198071	37206588	7.401	LOC100696294	receptor expression-enhancing protein 6	C
NC_031980.2	37207383	37210480	7.401	lg15h19orf25	UPF0449 protein C19orf25 homolog	C
NC_031980.2	37210850	37252997	7.401	apc2	APC2%2C WNT signaling pathway regulator%2C	C
NC_031980.2	37226554	37230905	7.401	LOC102080923	probable E3 ubiquitin-protein ligase TRIML1	C
NC_031980.2	37258167	37271875	7.401	LOC100706493	GTP-binding protein Di-Ras1	C
NC_031980.2	37373679	37375349	7.401	gadd45b	growth arrest and DNA damage inducible beta	C
NC_031980.2	37389709	37390967	7.401	LOC100695767	growth arrest and DNA damage-inducible protein GADD45 gamma	C
NC_031980.2	37392491	37393420	7.401	LOC100703831	growth arrest and DNA damage-inducible protein GADD45 beta-like	C
NC_031980.2	37398875	37409480	7.401	LOC102080291	survival motor neuron protein	C
NC_031980.2	37409613	37412896	7.401	LOC100701394	cocaine- and amphetamine-regulated transcript protein	C
NC_031980.2	37422522	37436595	7.401	LOC100695503	Ig kappa chain V-III region PC 2485/PC 4039-like	C
NC_031980.2	37442215	37448071	7.401	camk4	calcium/calmodulin dependent protein kinase IV%2C	C
NC_031980.2	37466551	37469839	7.786	lg15h19orf24	linkage group 15 C19orf24 homolog	C
NC_031980.2	37474554	37477260	7.786	LOC102079526	T-cell acute lymphocytic leukemia protein 1 homolog	C
NC_031980.2	37482503	37502695	7.786	kdm4a	lysine demethylase 4A	C
NC_031980.2	37506277	37694426	7.786	LOC100694711	receptor-type tyrosine-protein phosphatase F	C
NC_031980.2	37901403	37915605	10.064	hyi	hydroxypyruvate isomerase (putative)%2C	C
NC_031980.2	37920137	37929753	10.064	LOC109194886	KICSTOR complex protein SZT2	C
NC_031980.2	37981140	37982211	9.800	LOC109199849	coiled-coil domain-containing protein 106-like	C
NC_031980.2	37996761	37998823	9.346	LOC100703129	sorting nexin-9	C
NC_031980.2	39102596	39113676	7.432	vps16	VPS16%2C CORVET/HOPS core subunit	C
NC_031980.2	39192893	39284261	10.251	LOC100694175	KICSTOR complex protein SZT2	C
NC_031980.2	39286276	39290755	10.251	ddx10	DEAD-box helicase 10	C
NC_031980.2	39292511	39302961	10.251	LOC102080084	transcription initiation factor TFIID subunit 4	C
NC_031980.2	39304712	39318213	10.251	LOC100693637	BTB/POZ domain-containing protein KCTD1	C
NC_031980.2	39333399	39341657	10.251	LOC100698963	aquaporin-4	C
NC_031980.2	39461229	39461384	7.883	LOC112842543	5.8S ribosomal RNA	C
NC_031981.2	1494880	1505282	9.626	nek7	NIMA related kinase 7%2C	C
NC_031981.2	1613746	1729461	9.626	LOC112842638	receptor-type tyrosine-protein phosphatase C-like	C
NC_031981.2	23246385	23255402	10.735	tnpo3	transportin 3	C
NC_031981.2	23259248	23261101	10.695	opn1sw	opsin 1%2C short wave sensitive	C

NC_031981.2	23261103	23264301	10.695	calu	calumenin	C
NC_031981.2	23271112	23332634	10.695	LOC100705337	ephrin-A2	C
NC_031981.2	23353722	23369650	10.695	LOC109195251	desmoglein-2	C
NC_031981.2	23398864	23418340	10.695	LOC102083034	desmoglein-2	C
NC_031981.2	23439507	23455417	10.695	LOC102083206	desmoglein-2	C
NC_031981.2	23460879	23477825	10.695	LOC102083127	desmoglein-2	C
NC_031981.2	23485529	23490054	10.695	commd2	COMM domain containing 2	C
NC_031981.2	23490424	23496385	10.735	trappc8	trafficking protein particle complex 8%2C	C
NC_031982.2	13820066	13822543	7.655	LOC106097911	putative nuclease HARBI1	C
NC_031982.2	13830947	13839943	7.655	LOC112842904	deleted in malignant brain tumors 1 protein-like	C
NC_031982.2	13845094	13858081	7.655	LOC106098023	deleted in malignant brain tumors 1 protein	C
NC_031982.2	13861952	13908000	7.655	LOC102078004	deleted in malignant brain tumors 1 protein	C
NC_031982.2	13942124	13954204	7.655	aknad1	AKNA domain containing 1%2C	C
NC_031982.2	13954376	13965007	7.655	gpsm2	G protein signaling modulator 2%2C	C
NC_031982.2	13965085	13978559	7.655	wdr47	WD repeat domain 47%2C	C
NC_031982.2	13980672	14013610	7.655	LOC100701512	calmodulin-regulated spectrin-associated protein 2	C
NC_031982.2	14014870	14018549	7.655	LOC100701239	TIR domain-containing adapter molecule 1	C
NC_031982.2	14044748	14045937	7.655	LOC100693396	C-C motif chemokine 20	C
NC_031982.2	14052553	14053245	7.655	LOC109195725	C-C motif chemokine 20-like	C
NC_031982.2	17278196	17297923	7.895	cdh2	cadherin 2	C
NC_031982.2	17398919	17407010	10.015	LOC100707499	desmocollin-2	C
NC_031982.2	17412241	17441936	10.015	LOC112842746	desmocollin-2-like	C
NC_031982.2	17447507	17457220	10.015	LOC102081358	desmoglein-2	C
NC_031982.2	17459544	17464161	10.015	LOC100702414	tubulin beta chain	C
NC_031982.2	17464862	17504418	10.015	LOC100707236	solute carrier family 22 member 23	C
NC_031982.2	17509648	17525595	10.015	dsp	desmoplakin	C
NC_031982.2	17526269	17528196	7.895	tns3	tensin 3%2C	C
NC_031982.2	29981098	29983250	7.685	LOC109195492	tripartite motif-containing protein 16-like	C
NC_031982.2	30003624	30017683	7.685	LOC109195498	E3 ubiquitin/ISG15 ligase TRIM25-like	C
NC_031982.2	30014117	30016425	7.685	LOC109195493	tripartite motif-containing protein 16-like	C
NC_031982.2	30024335	30046144	7.685	LOC109195491	E3 ubiquitin/ISG15 ligase TRIM25-like	C
NC_031982.2	30054693	30059547	7.685	LOC109195500	serine/threonine-protein kinase pim-2-like	C
NC_031982.2	30063428	30064598	7.685	LOC109195445	differentially expressed in FDCP 6 homolog	C
NC_031982.2	30067740	30069809	7.685	LOC109195504	tripartite motif-containing protein 16-like protein	C

NC_031982.2	30087506	30088603	7.685	LOC100697853	E3 ubiquitin/ISG15 ligase TRIM25-like	C
NC_031982.2	30092757	30096250	7.685	LOC109195497	serine/threonine-protein kinase pim-2	C
NC_031982.2	30106594	30108837	7.685	LOC109195495	tripartite motif-containing protein 16-like	C
NC_031982.2	30148432	30150765	7.685	LOC109195494	tripartite motif-containing protein 16-like	C
NC_031982.2	30161973	30162567	7.685	LOC109195502	malonyl-CoA-acyl carrier protein transacylase%2C mitochondrial-like	C
NC_031982.2	30178553	30181731	7.685	LOC109195529	differentially expressed in FDCP 6 homolog	C
NC_031982.2	30202461	30204622	7.685	LOC106097803	tripartite motif-containing protein 16	C
NC_031982.2	35491943	35503283	11.407	LOC100703857	prohibitin-2	C
NC_031982.2	35508194	35523031	11.407	LOC100704126	protein FAM49B	C
NC_031982.2	35525676	35690594	7.614	LOC100704393	zinc finger protein 2	C
NC_031982.2	35553902	35557463	11.407	LOC100703314	transcriptional regulator Myc-2	C
NC_031982.2	37032845	37039557	10.225	LOC100692678	gypsy retrotransposon integrase-like protein 1%2C	C
NC_031982.2	37052249	37052957	10.225	LOC112842974	T cell receptor alpha variable 18-like	C
NC_031982.2	37063894	37065480	10.225	LOC100692951	T cell receptor alpha variable 38-1-like	C
NC_031982.2	37104541	37105276	10.225	LOC102080414	T cell receptor alpha variable 18-like	C
NC_031982.2	37112364	37113032	10.225	LOC112842817	T cell receptor alpha variable 21-like	C
NC_031982.2	37114696	37116203	10.225	LOC109201758	T cell receptor alpha variable 38-1-like	C
NC_031982.2	37132368	37133092	10.225	LOC109201759	T cell receptor alpha variable 19-like	C
NC_031982.2	37143962	37144699	10.225	LOC109201848	T cell receptor alpha variable 38-2/delta variable 8-like	C
NC_031982.2	37160034	37160704	10.225	LOC109201849	T cell receptor alpha variable 18-like	C
NC_031982.2	37201991	37202751	10.225	LOC112842800	T cell receptor alpha variable 3-like	C
NC_031982.2	37206849	37207589	10.225	LOC109201760	T cell receptor alpha variable 38-2/delta variable 8-like	C
NC_031982.2	37234314	37234974	10.225	LOC109201850	T cell receptor alpha variable 18-like	C
NC_031982.2	37251440	37252265	10.225	LOC112842799	T cell receptor alpha variable 39-like	C
NC_031982.2	37412922	37413934	10.942	LOC112842814	T cell receptor alpha variable 18-like	C
NC_031982.2	37417228	37425144	10.942	LOC106097252	zinc finger protein 2 homolog	C
NC_031982.2	37436937	37443909	10.942	LOC102079485	golgin subfamily A member 6-like protein 6	C
NC_031982.2	37446067	37453441	10.942	iqub	IQ and ubiquitin-like domain-containing protein	C
NC_031982.2	37462409	37463245	10.942	LOC112842811	T cell receptor alpha variable 36/delta variable 7-like	C
NC_031982.2	37470175	37513190	10.942	LOC102079576	zinc finger and SCAN domain-containing protein 2	C
NC_031982.2	37480700	37485194	10.942	LOC109203498	zinc finger protein 664-like	C
NC_031982.2	37494373	37554807	7.528	LOC106097251	golgin subfamily A member 6-like protein 6	C
NC_031982.2	37519110	37520000	10.942	LOC100711528	T cell receptor alpha variable 38-2/delta variable 8-like	C
NC_031982.2	37528599	37529468	10.942	LOC112842815	T cell receptor alpha variable 9-1-like	C

NC_031982.2	37533170	37535309	10.942	LOC112842895	T cell receptor alpha variable 3-like	C
NC_031982.2	37537857	37538534	10.942	LOC112842818	T cell receptor alpha variable 19-like	C
NC_031982.2	37549992	37554807	7.528	LOC102079862	caspase-1-like	C
NC_031982.2	37599420	37600717	9.218	LOC112842816	T cell receptor alpha variable 38-1-like	C
NC_031982.2	37639086	37639841	9.218	LOC112842797	T cell receptor alpha variable 18-like	C
NC_031982.2	37649220	37656464	9.218	LOC106097647	gastrula zinc finger protein XlCGF8.2DB	C
NC_031982.2	37673167	37673856	9.218	LOC112842805	T cell receptor alpha variable 38-2/delta variable 8-like	C
NC_031982.2	37682219	37687702	9.218	LOC100690514	zinc finger protein with KRAB and SCAN domains 8-like	C
NC_031982.2	37695648	37696519	9.218	LOC109203499	T cell receptor alpha variable 18-like	C
NC_031982.2	38185408	38194750	10.015	LOC109195407	zinc finger protein 2 homolog	C
NC_031982.2	38229543	38230261	10.015	LOC112842859	T cell receptor alpha variable 3-like	C
NC_031982.2	38238272	38239007	10.015	LOC109195401	T cell receptor alpha variable 38-2/delta variable 8-like	C
NC_031982.2	38264057	38265006	10.015	LOC112842978	T cell receptor alpha variable 3-like	C
NC_031982.2	38271798	38272772	10.015	LOC112842856	T cell receptor alpha variable 3-like	C
NC_031982.2	38280570	38289321	10.015	LOC100705385	zinc finger protein 260	C
NC_031982.2	38305865	38307243	10.015	LOC102081108	T cell receptor alpha variable 40-like	C
NC_031982.2	38346220	38347470	10.015	LOC109195402	T cell receptor alpha variable 18-like	C
NC_031982.2	38367906	38371187	10.015	LOC112842854	zinc finger BED domain-containing protein 4-like	C
NC_031982.2	38393491	38431197	10.015	LOC112842855	T cell receptor alpha variable 3-like	C
NC_031982.2	38407408	38408140	10.015	LOC112842857	T cell receptor alpha variable 38-2/delta variable 8-like	C
NC_031982.2	7563488	7563550	15.176	mcm6	DNA replication licensing factor MCM6	C
NC_031982.2	7567296	7577797	11.603	LOC100701870	protein lifeguard 3	C
NC_031982.2	7579262	7589952	11.603	LOC100702139	caspase-8	C
NC_031982.2	7589059	7593534	11.603	catip	ciliogenesis associated TTC17 interacting protein%2C	C
NC_031982.2	7595310	7597386	11.603	LOC100702676	caspase-8	C
NC_031982.2	7634989	7640370	11.603	LOC102079913	caspase-8	C
NC_031982.2	7650302	7655687	11.603	LOC106097537	retrovirus-related Pol polyprotein from transposon 412	C
NC_031982.2	7688213	7691174	11.603	LOC100702924	GTPase IMAP family member 4-like	C
NC_031982.2	7712737	7716058	11.603	LOC109195365	zinc finger MYM-type protein 1-like	C
NC_031982.2	7748687	7752816	11.603	LOC100701212	GTPase IMAP family member 4-like	C
NC_031982.2	7775403	7782285	11.603	LOC100700669	GTPase IMAP family member 4	C
NC_031982.2	7785691	7788603	11.603	LOC109194579	GTPase IMAP family member 4-like	C
NC_031982.2	7805588	7808624	11.603	LOC100699857	GTPase IMAP family member 4-like	C
NC_031984.2	3790433	3808236	8.292	LOC109196279	plexin A3	C

NC_031984.2	3874603	3887494	8.021	LOC100698542	interferon-inducible GTPase 5	C
NC_031984.2	4013481	4018998	8.021	LOC100698012	GTPase IMAP family member 7-like	C
NC_031985.2	23476095	23477739	7.713	LOC109196405	lactose-binding lectin l-2-like	C
NC_031985.2	23522459	23523569	7.713	LOC100692342	lactose-binding lectin l-2	C
NC_031985.2	23527774	23529265	7.713	LOC109196397	golgin subfamily A member 6-like protein 22	C
NC_031985.2	23536921	23538527	7.713	LOC100712526	lactose-binding lectin l-2-like	C
NC_031985.2	23542555	23543525	7.713	LOC112843465	lactose-binding lectin l-2-like	C
NC_031985.2	23552691	23554339	7.713	LOC100691892	lactose-binding lectin l-2	C
NC_031985.2	23563096	23564483	7.713	LOC109196403	lactose-binding lectin l-2	C
NC_031985.2	23571211	23575549	7.713	LOC100694396	lactose-binding lectin l-2-like	C
NC_031985.2	23578114	23583882	7.713	LOC106097545	lactose-binding lectin l-2-like	C
NC_031985.2	23587563	23589037	7.713	LOC109196400	lactose-binding lectin l-2-like	C
NC_031985.2	23589923	23591265	7.713	LOC112843475	galactose-specific lectin nattectin-like	C
NC_031985.2	23593167	23594920	7.713	LOC109196402	lactose-binding lectin l-2	C
NC_031985.2	23597839	23599083	7.713	LOC102078271	lactose-binding lectin l-2	C
NC_031985.2	23605099	23606419	7.713	LOC109196404	lactose-binding lectin l-2-like	C
NC_031985.2	23679201	23681172	7.713	LOC109196463	lactose-binding lectin l-2-like	C
NC_031985.2	23682919	23715150	7.713	LOC100706258	galactose-specific lectin nattectin	C
NC_031985.2	23700291	23702004	7.713	LOC112841631	lactose-binding lectin l-2-like	C
NC_031985.2	28537955	28537966	8.022	topaz1	protein TOPAZ1	C
NC_031985.2	28564552	28569061	12.594	LOC100709024	1-acylglycerol-3-phosphate O-acyltransferase ABHD5	C
NC_031985.2	28579169	28596542	12.594	LOC100709297	SNF-related serine/threonine-protein kinase	C
NC_031985.2	28599849	28603410	12.594	rpl14	60S ribosomal protein L14	C
NC_031985.2	28612836	28617275	12.594	LOC100708135	leukocyte elastase inhibitor-like	C
NC_031986.2	14730155	14731258	9.260	LOC102080405	leukotriene-B(4) omega-hydroxylase 2	C
NC_031986.2	14771642	14773126	9.260	LOC112843912	zinc finger BED domain-containing protein 4-like	C
NC_031986.2	14776342	14777236	9.260	LOC112843780	OX-2 membrane glycoprotein-like	C
NC_031986.2	14785799	14788112	9.260	LOC106096586	OX-2 membrane glycoprotein%2C	C
NC_031986.2	14795994	14799175	9.260	LOC106096584	OX-2 membrane glycoprotein%2C	C
NC_031986.2	14803010	14825256	9.260	LOC100710881	OX-2 membrane glycoprotein	C
NC_031986.2	14831018	14833432	9.260	LOC106096583	B- and T-lymphocyte attenuator	C
NC_031986.2	14837887	14853424	9.260	LOC109197094	B- and T-lymphocyte attenuator	C
NC_031986.2	14854308	14860274	9.260	LOC102081783	B- and T-lymphocyte attenuator-like	C
NC_031986.2	14860437	14871074	9.260	LOC100708021	splicing factor U2AF 35 kDa subunit	C

NC_031986.2	14872701	14883131	9.260	LOC100707752	cystathionine beta-synthase		C
NC_031986.2	14907296	14926142	9.260	LOC100710616	vascular endothelial growth factor C		C
NC_031986.2	14937017	14938528	9.260	LOC100707486	trace amine-associated receptor 13c-like		C
NC_031986.2	14949624	14950601	9.260	LOC100707222	trace amine-associated receptor 13c-like		C
NC_031986.2	14958014	14958991	9.260	LOC109196918	trace amine-associated receptor 13c-like		C
NC_031986.2	14960813	14962401	9.260	LOC100706960	trace amine-associated receptor 13c-like		C
NC_031986.2	14974390	14975367	9.260	LOC100706689	trace amine-associated receptor 13c-like		C
NC_031986.2	26602543	26613000	9.710	LOC109204420	nucleotide-binding oligomerization domain-containing protein 1-like		C
NC_031986.2	26661494	26740281	10.456	LOC100701774	NLR family CARD domain-containing protein 3		C
NC_031986.2	26751195	26760194	10.456	LOC106096908	protein NLRC3		C
NC_031986.2	26766208	26777059	10.456	LOC100709649	protein NLRC3		C
NC_031986.2	26778968	26782846	10.456	LOC100709380	ribonuclease inhibitor		C
NC_031986.2	26804207	26818322	10.456	LOC102081256	protein NLRC3-like		C
NC_031986.2	26825027	26844209	10.456	LOC100708839	protein NLRC3		C
NC_031986.2	26845361	26852543	9.710	LOC106096905	protein NLRC3		C
NC_031986.2	27118861	27128584	7.479	LOC100706698	kalirin		C
NC_031986.2	27130905	27137322	10.735	iqcb1	IQ calmodulin-binding motif-containing protein 1		C
NC_031986.2	27137452	27155935	10.735	LOC100706430	protein EFR3 homolog B		C
NC_031986.2	27157409	27159040	10.735	pomc	pro-opiomelanocortin		C
NC_031986.2	27165657	27176682	10.735	LOC100706171	protein NLRC3		C
NC_031986.2	27180807	27200655	10.735	LOC102080998	protein NLRC3		C
NC_031986.2	27192753	27196098	10.735	LOC112843957	neoverrucotoxin subunit alpha-like		C
NC_031986.2	27208075	27209718	10.735	LOC102080901	E3 ubiquitin-protein ligase TRIM39-like		C
NC_031986.2	27247382	27251732	10.735	LOC109204585	protein NLRC3-like		C
NC_031986.2	27285074	27291568	10.735	LOC109204633	stonustoxin subunit beta-like		C
NC_031986.2	27305357	27312681	10.735	LOC100705638	NLR family CARD domain-containing protein 3-like		C
NC_031986.2	27314922	27330778	10.735	LOC100705372	protein NLRC3-like		C
NC_031986.2	28170383	28190713	7.407	LOC102077510	adhesion G-protein coupled receptor G7		C
NC_031986.2	28196519	28205210	7.407	ftcd	formimidoyltransferase-cyclodeaminase		C
NC_031986.2	28210714	28225591	7.407	LOC100710713	cyclin-dependent kinase 15		C
NC_031986.2	28239954	28243886	7.407	LOC100710446	frizzled-7-like		C
NC_031986.2	28268056	28270139	7.407	LOC100710187	putative claudin-24		C
NC_031986.2	28304203	28308618	7.407	LOC109196789	adhesion G-protein coupled receptor G7-like		C
NC_031986.2	28391149	28401834	7.407	LOC106098147	NLR family CARD domain-containing protein 3		C

NC_031986.2	28403744	28415508	7.407	LOC102080577	adhesion G-protein coupled receptor G7	C
NC_031987.2	19358159	19379864	7.458	LOC102078547	nectin-4	C
NC_031987.2	19408352	19428980	7.458	LOC102079924	nectin-3	C
NC_031987.2	19433621	19525482	7.458	LOC102079988	nectin-2	C
NC_031987.2	19470296	19487635	7.458	LOC102079645	nectin-2	C
NC_031987.2	19487868	19509392	7.458	LOC109194266	nectin-4	C
NC_031987.2	19531838	19559823	7.458	LOC102078979	nectin-2	C

LG: Linkage group
initial pos: initial position
final pos: final position
