# Supplementary material for: Whole genome re-sequencing reveals recent signatures of selection in three strains of farmed Nile tilapia (Oreochromis niloticus)
Source: Sci Rep. 2020 Jul 13;10:11514. doi: 10.1038/s41598-020-68064-5 (PMC7359307; doi:10.1038/s41598-020-68064-5)
Supplement: Supplementary file 7 — Supplementary table S5 [file 41598_2020_68064_MOESM7_ESM.pdf]

Supplementary information

Whole genome re-sequencing reveals recent signatures of selection in three strains of farmed Nile tilapia (*Oreochromis niloticus*)

María I. Cádiz[12], María E. López[31], Diego Díaz-Domínguez[4], Giovanna Cáceres[12], Grazyella M. Yoshida[1], Daniel Gomez-Uchida[5.6], José M. Yáñez[1,6]*.

[1] Facultad de Ciencias Veterinarias y Pecuarias, Universidad de Chile, Avenida Santa Rosa 11735, 8820808, La Pintana, Santiago, Chile

[2] Programa de Doctorado en Ciencias Silvoagropecuarias y Veterinarias, Campus Sur, Universidad de Chile, Santa Rosa 11315, La Pintana, Santiago, Chile. CP: 8820808.

[3] Department of Animal Breeding and Genetics, Swedish University of Agricultural Sciences, Uppsala, Sweden.

[4] Departamento de Ciencias de la Computación, Universidad de Chile.

[5] Facultad de Ciencias Naturales y Oceanográficas, Universidad de Concepción, Concepción, Chile.

[6] Núcleo Milenio INVASAL, Concepción, Chile

*jmayanez@uchile.cl +56-2 29785533 (Corresponding Author).

Table S5. Candidate genes for selection detected by iHS, Rsb and FST methods.

Gene	*Description/Function*	*Trait[1]*	*iHS*	*Rsb*	F_{ST}
ANKRD46	This gene belongs to the ankyrin gene family, which regulates gene expression in pathways controlling growth, myogenesis and innate immunity [1]. It has been associated with meat quality traits in chicken[2]	G	X	X	
TTN	It is essential to muscle architecture and signaling in developing and mature striated muscle. Mutations in this gene have been correlated with skeletal muscular dystrophy-like on zebrafish[3]	G	X	X	
TCD7L1	Involved in the canonical Wnt signaling pathway[4]	G	X	X	
VCAM	It has been reported to have a specific role in myotubes formation and fusion[5]	G	X	X	
KIF1C	Linked to the regulation of the cytoskeletal organization and osteoclastic bone resorption[6]	G	X	X	
NCAPG	Involved in the condensation and stabilization of chromosomes during meiosis and mitosis[7] and to growth traits in cattle[8], equine[9], chicken[10] and sheep[11]	G			X
KLF3	Essential member of the KLFs family. It is involved in the growth and development of muscle and adipose tissue in cattle[12] and goat[13]	G			X
TBC1D1	Critical signaling factor of skeletal muscle substrate utilization[14]. It was correlated with improved muscle mass (chicken[15], porcine[16] and rabbits[17])	G			X
IL15RA	Associated to osteoblast function, bone mineralization[18], signaling in muscle oxidative metabolism, and adaptation to exercise in mouse[19]	G			X
OPTN	Regulator of bone resorption and the reductions in its expression predispose to Paget's disease of bone[20].	G			X
ADRA1D	Contribute to cell survival and maintenance of skeletal muscle[21]	G			X
DOC2B	Insulin responsiveness of skeletal muscle[22]	G			X
CISD2	Aging of skeletal muscle[23]	G			X
ELAVL1	Putative role in embryonic erythropoiesis in zebrafish[24]	E	X	X	
SYNA	Cell fusion and differentiation involved in placental development[25]	E	X	X	
GNG752	Putative role in placental development in the mouse[26]	E	X	X	
TSPAN3	Related to embryogenesis in amphibia[27]	E	X	X	
G2E3	Related to embryogenesis in mouse[28]	E	X	X	
PLTP	Odontogenesis in mice and gar fish[29]	E	X	X	
FGFR3	Early embryogenesis in zebrafish[30]	E			X
PFKFB3	Angiogenic process in zebrafish[31]	E			X
AHSA1	Severe craniofacial phenotypes in zebrafish[32]	E			X
BDH2	Mitochondrial dysfunction and delays erythroid maturation in zebrafish[33]	E			X
METTL14	Early embryogenesis in mouse[34]	E			X
LOXL3	Palatogenesis and vertebral column development[35]	E			X

SERTAD1	Cardiogenesis in mouse[36]	E			X
PGM2	Embryonic lethality in mouse[37]	E			X
CAMK1D	Involved in calmodulin-dependent signaling, a pathway for the activation of myogenesis[38] and embryonic development[39]	E			X
NLRC3	Associated to the defense against bacterial pathogens *Streptococcus agalactiae* in Nile tilapia[40]	I	X	X	
PIGR	Involved in the defense against the *Streptococcus agalactiae* and *Aeromonas hydrophila* in Nile tilapia[41]	I	X	X	
MAP1S	Associated to the defense against *Streptococcus iniae* (MAP1S [42]) in Nile tilapia	I	X	X	
Ladderlectin	Associated with an innate immune response mechanism, which corresponds to plasma pattern recognition for bacterial, fungal and viral hemorrhagic septicemia in rainbow trout[43]	I	X	X	
HAVCR2	Role in the immune system of *Oryzias latipes* [44]	I	X	X	
FCRL5	Role in the immune system of *Miichthys miiuy*[45]	I	X	X	
TRIM16L	Role in the immune system of *Epinephelus coioides*[46]	I	X	X	
PAX5	Possibly involved in B cell activation in Nile tilapia[47]	I			X
MAVS	Involved in innate antibacterial immunity in Nile tilapia[48]	I			X
SERPING1	Associated with a response against Grass Carp Reovirus infection of *Ctenopharyngodon idella*[49]	I			X
LONRF1	Role against outbreaks of the Porcine Reproductive and Respiratory Syndrome Virus (PRRSV) pathogen in pigs[50]	I			X
CDK17	Role against outbreaks of the PRRSV pathogen in pigs[51]	I			X
IFI44L	Linked to the social average daily gain in pigs[52]	B	X	X	
GTF2IRD1	The disruption of this gene was correlated with aggression, natural fear answer, and enhanced social interaction in mouse[53]	B	X	X	
BTN3A2	Linked to risk of schizophrenia in mouse[54]	B	X	X	
LRRTM1	Associated with claustrophobia-like behavior in mouse[55]	B			X
CTNNA2	Implicated in obsessive-compulsive disorder in canine[56]	B			X
UGT8	Linked to mood disorders in humans[57]	B			X
SPOCK3	Attention-deficit/hyperactivity disorder and personality disorders in humans[58]	B			X
GPA33	Sexual dimorphism on Nile tilapia[59].	R	X	X	
VIPR2	Role in the pathway of the follicle growth and maturation in zebrafish[60]	R	X	X	
CARTPT	Putative role in dominant follicle selection in cattle[61]	R	X	X	
ASMT	Implicated in encoding the second enzyme required for melatonin synthesis[62]. This hormone engaged in changes in growth, gonadal maturity, lipid and protein production in Nile Tilapia[63]	R			X
NANOS1	Linked to the maintenance of oocyte production in zebrafish[64]	R			X
DIP2C	Associated to major QTL of salinity tolerance in Nile tilapia[65]	A	X	X	

[1] Trait, trait associated with domestication: (G) Growth, (E) Early development, (B) Behavior, (R) Reproduction, (A) Adaptation to environment and (I) Immune system. iHS, Rsb, F_{ST}: method by genes were detected.

References

1. Johnston, I. A., Bower, N. I. & Macqueen, D. J. Growth and the regulation of myotomal muscle mass in teleost fish. *J. Exp. Biol.* **214**, 1617–1628 (2011).
2. Zhang, T. *et al.* Genome-wide association study of meat quality traits in chicken. *Genet. Mol. Res.* **14**, 10452–10460 (2015).
3. Steffen, L. S. *et al.* The zebrafish runzel muscular dystrophy is linked to the titin gene. *Dev. Biol.* **309**, 180–192 (2007).
4. Ye, B., Haodong, L. L. X. & Faqian, C. Y. L. Opposing Roles of Tcf7/Lef1 and Tcf7l2 in Cyclin D2 and Bmp4 Expression and Cardiomyocyte Cell Cycle Control during Late Heart Development. *Lab. Investig.* **99**, 807–818 (2019).
5. Gentile, A. *et al.* Human epicardium-derived cells fuse with high efficiency with skeletal myotubes and differentiate toward the skeletal muscle phenotype: A comparison study with stromal and endothelial cells. *Mol. Biol. Cell* **22**, 581–592 (2011).
6. Kobayakawa, M. *et al.* Kif1c regulates osteoclastic bone resorption as a downstream molecule of p130Cas. *Cell Biochem. Funct.* (2019) doi:10.1002/cbf.3476.
7. Al-Mamun, H. A. *et al.* Genome-wide association study of body weight in Australian Merino sheep reveals an orthologous region on OAR6 to human and bovine genomic regions affecting height and weight. *Genet. Sel. Evol.* **47**, 1–11 (2015).
8. Smith, J. L. *et al.* Genome-wide association and genotype by environment interactions for growth traits in U.S. Gelbvieh cattle. *BMC Genomics* **20**, 1–13 (2019).
9. Grilz-Seger, G. *et al.* Analysis of ROH patterns in the Noriker horse breed reveals signatures of selection for coat color and body size. *Anim. Genet.* **50**, 334–346 (2019).
10. Lyu, S., Arends, D., Nassar, M. K. & Brockmann, G. A. Fine mapping of a distal chromosome 4 QTL affecting growth and muscle mass in a chicken advanced intercross line. *Anim. Genet.* **48**, 295–302 (2017).
11. Signer-Hasler, H., Burren, A., Ammann, P., Drögemüller, C. & Flury, C. Runs of homozygosity and signatures of selection: a comparison among eight local Swiss sheep breeds. *Anim. Genet.* **50**, 512–525 (2019).
12. Xu, J. W. *et al.* Novel copy number variation of the KLF3 gene is associated with growth traits in beef cattle. *Gene* **680**, 99–104 (2019).
13. Xu, Q. *et al.* Fibroblast growth factor 21 regulates lipid accumulation and adipogenesis in goat intramuscular adipocyte. *Anim. Biotechnol.* (2019) doi:doi.org/10.1080/10495398.2019.1691010 Fibroblast.
14. Espelage, L., Al-Hasani, H. & Chadt, A. RabGAPs TBC1D1 and TBC1D4 in skeletal muscle function and exercise. *J. Mol. Endocrinol.* (2019) doi:10.1530/jme-19-0143.
15. Rubin, C. J. *et al.* Whole-genome resequencing reveals loci under selection during chicken domestication. *Nature* **464**, 587–591 (2010).
16. Fontanesi, L. *et al.* Identification and association analysis of several hundred single nucleotide polymorphisms within candidate genes for back fat thickness in Italian large white pigs using a selective genotyping approach. *J. Anim. Sci.* **90**, 2450–2464 (2012).
17. Yang, Z. J. *et al.* Identification and association of SNPs in TBC1D1 gene with growth traits in two rabbit breeds. *Asian-Australasian J. Anim. Sci.* **26**, 1529–1535 (2013).
18. Loro, E. *et al.* IL15RA is required for osteoblast function and bone mineralization. *Bone* **103**, 20–30 (2017).

19. Loro, E., Bisetto, S. & Khurana, T. S. Mitochondrial ultrastructural adaptations in fast muscles of mice lacking IL15RA. *J. Cell Sci.* **131**, (2018).
20. Obaid, R. *et al.* Optineurin Negatively Regulates Osteoclast Differentiation by Modulating NF-κB and Interferon Signaling: Implications for Paget's Disease. *Cell Rep.* **13**, 1096–1102 (2015).
21. Saini, A., Al-Shanti, N. & Stewart, C. C2 skeletal myoblast survival, death, proliferation and differentiation: Regulation by adra1d. *Cell. Physiol. Biochem.* **25**, 253–262 (2010).
22. Zhang, J. *et al.* DOC2B promotes insulin sensitivity in mice via a novel KLC1-dependent mechanism in skeletal muscle. *Diabetologia* **62**, 845–859 (2019).
23. Huang, Y. L. *et al.* Comparative proteomic profiling reveals a role for Cisd2 in skeletal muscle aging. *Aging Cell* **17**, (2018).
24. Li, X. *et al.* Elavl1a regulates zebrafish erythropoiesis via posttranscriptional control of gata1. *Blood* **123**, 1384–1392 (2014).
25. Gong, R. *et al.* Syncytin-A mediates the formation of syncytiotrophoblast involved in mouse placental development. *Cell. Physiol. Biochem.* **20**, 517–526 (2007).
26. Lai, W. S. & Ding, Y. L. GNG7 silencing promotes the proliferation and differentiation of placental cytotrophoblasts in preeclampsia rats through activation of the mTOR signaling pathway. *Int. J. Mol. Med.* **43**, 1939–1950 (2019).
27. Kashef, J., Diana, T., Oelgeschläger, M. & Nazarenko, I. Expression of the tetraspanin family members Tspan3, Tspan4, Tspan5 and Tspan7 during Xenopus laevis embryonic development. *Gene Expr. Patterns* **13**, 1–11 (2013).
28. Brooks, W. S. *et al.* G2E3 is a dual function ubiquitin ligase required for early embryonic development. *J. Biol. Chem.* **283**, 22304–22315 (2008).
29. Rostampour, N., Appelt, C., Abid, A. & Boughner, J. Expression of new genes in vertebrate tooth development and p63 signaling. *Dev. Dyn.* **248**, 744–755 (2019).
30. Ota, S. *et al.* FGF receptor gene expression and its regulation by FGF signaling during early zebrafish development. *Genesis* **48**, 707–716 (2010).
31. Wade, S. M. *et al.* Dysregulated miR-125a promotes angiogenesis through enhanced glycolysis. *EBioMedicine* **47**, 402–413 (2019).
32. Sheehan-Rooney, K., Swartz, M. E., Zhao, F., Liu, D. & Eberhart, J. K. Ahsa1 and Hsp90 activity confers more severe craniofacial phenotypes in a zebrafish model of hypoparathyroidism, sensorineural deafness and renal dysplasia (HDR). *DMM Dis. Model. Mech.* **6**, 1285–1291 (2013).
33. Davuluri, G. *et al.* Inactivation of 3-hydroxybutyrate dehydrogenase 2 delays zebrafish erythroid maturation by conferring premature mitophagy. *Proc. Natl. Acad. Sci. U. S. A.* **113**, E1460–E1469 (2016).
34. Meng, T.-G. *et al.* Mettl14 is required for mouse postimplantation development by facilitating epiblast maturation. *FASEB J.* **33**, 1179–1187 (2019).
35. Zhang, J. *et al.* Loss of lysyl oxidase-like 3 causes cleft palate and spinal deformity in mice. *Hum. Mol. Genet.* **24**, 6174–6185 (2015).
36. Peng, Y., Zhao, S., Song, L., Wang, M. & Jiao, K. Sertad1 Encodes a Novel Transcriptional co-Activator of SMAD1 in Mouse Embryonic Hearts. *Biochem Biosphy Res Commun* **29**, 4 (2014).
37. Balakrishnan, B. *et al.* A novel phosphoglucomutase-deficient mouse model reveals aberrant glycosylation and early embryonic lethality. *J. Inherit. Metab. Dis.* **42**, 998–1007 (2019).
38. Olson, E. N. & Williams, R. S. Remodeling muscles with calcineurin. *BioEssays* **22**, 510–519 (2000).

39. White, R. & Ziman, M. Genome-wide discovery of Pax7 target genes during development Robert. *Physiol Genomics* **33**, 41–49 (2008).
40. Gao, F. ying *et al.* Molecular characterization, expression and functional analysis of NOD1, NOD2 and NLRC3 in Nile tilapia (Oreochromis niloticus). *Fish Shellfish Immunol.* **73**, 207–219 (2018).
41. Liu, S. *et al.* Expression and functional analysis of polymeric immunoglobulin receptor in Nile tilapia (Oreochromis niloticus). *Aquaculture* **500**, 41–49 (2019).
42. Qiang, J. *et al.* Effects of exposure to Streptococcus iniae on microRNA expression in the head kidney of genetically improved farmed tilapia (Oreochromis niloticus). *BMC Genomics* **18**, 1–11 (2017).
43. Reid, A., Young, K. M. & Lumsden, J. S. Rainbow trout Oncorhynchus mykiss ladderlectin, but not intelectin, binds viral hemorrhagic septicemia virus IVb. *Dis. Aquat. Org.* **95**, 137–143 (2011).
44. Nibona, E. *et al.* Identification, characterization, expression profiles of OlHavcr2 in medaka (Oryzias latipes). *Gen. Comp. Endocrinol.* **277**, 30–37 (2019).
45. Xu, T. J., Meng, F. X., Sun, Y. N., Shi, G. & Wang, R. X. Identification of immune genes of the miiuy croaker (Miichthys miiuy) by sequencing and bioinformatic analysis of ESTs. *Fish Shellfish Immunol.* **29**, 1099–1105 (2010).
46. Yu, Y. *et al.* Fish TRIM16L exerts negative regulation on antiviral immune response against grouper iridoviruses. *Fish Shellfish Immunol.* **59**, 256–267 (2016).
47. Wu, L. *et al.* Molecular characterization and transcriptional expression of a B cell transcription factor Pax5 in Nile tilapia (Oreochromis niloticus). *Fish Shellfish Immunol.* **90**, 165–172 (2019).
48. Gao, F. Y. *et al.* Molecular characterization and function analysis of three RIG-I-like receptor signaling pathway genes (MDA5, LGP2 and MAVS) in Oreochromis niloticus. *Fish Shellfish Immunol.* **82**, 101–114 (2018).
49. Chen, L. *et al.* Cloning of six serpin genes and their responses to GCRV infection in grass carp (Ctenopharyngodon idella). *Fish Shellfish Immunol.* **86**, 93–100 (2019).
50. Walker, L. R. *et al.* Genome-wide association analysis for porcine reproductive and respiratory syndrome virus susceptibility traits in two genetic populations of pigs. *J. Anim. Sci.* **97**, 3253–3261 (2019).
51. Bai, J. *et al.* A high-throughput screen for genes essential for PRRSV infection using a piggyBac-based system. *Virology* **531**, 19–30 (2019).
52. Hong, J. K. *et al.* A genome-wide association study of social genetic effects in Landrace pigs. **31**, 784–790 (2018).
53. Young, E. J. *et al.* Reduced fear and aggression and altered serotonin metabolism in Gtf2ird1-targeted mice. *GENES BRAIN Behav.* **7**, 224–234 (2008).
54. Wu, Y. *et al.* Identification of the primate-specific gene BTN3A2 as an additional schizophrenia risk gene in the MHC loci. *EBioMedicine* **44**, 530–541 (2019).
55. Voikar, V. *et al.* LRRTM1-deficient mice show a rare phenotype of avoiding small enclosures-A tentative mouse model for claustrophobia-like behaviour. *Behav. Brain Res.* **238**, 69–78 (2013).
56. Tang, R. *et al.* Candidate genes and functional noncoding variants identified in a canine model of obsessive-compulsive disorder. *Genome Biol.*

15, (2014).
57. Le-Niculescu, H. *et al.* Identifying blood biomarkers for mood disorders using convergent functional genomics. *Mol. Psychiatry* **14**, 156–174 (2009).
58. Weber, H. *et al.* SPOCK3, a risk gene for adult ADHD and personality disorders. *Eur. Arch. Psychiatry Clin. Neurosci.* **264**, 409–421 (2014).
59. Eshel, O. *et al.* Identification of male-specific amh duplication, sexually differentially expressed genes and microRNAs at early embryonic development of Nile tilapia (Oreochromis niloticus). *BMC Genomics* **15**, (2014).
60. Zhou, R., Tsang, A. H. K., Lau, S.-W. & Ge, W. Pituitary Adenylate Cyclase-Activating Polypeptide (PACAP) and Its Receptors in the Zebrafish Ovary: Evidence for Potentially Dual Roles of PACAP in Controlling Final Oocyte Maturation. *Biol. Reprod.* **85**, 615–625 (2011).
61. Smith, G. W., Sen, A., Folger, J. K. & Ireland, J. J. Putative role of cocaine- and amphetamine-regulated transcript (CARTPT) in dominant follicle selection in cattle. *Soc. Reprod. Fertil. Suppl.* **67**, 105–17 (2010).
62. Huelsmann, M. *et al.* Genes lost during the transition from land to water in cetaceans highlight genomic changes associated with aquatic adaptations. *Sci. Adv.* **5**, (2019).
63. Singh, R., Singh, A. K. & Madhu Tripathi. Melatonin Induced Changes in Specific Growth Rate, Gonadal Maturity, Lipid and Protein Production in Nile Tilapia Oreochromis niloticus (Linnaeus 1758). *Asian-Australasian J. Anim. Sci.* **25**, 37–43 (2012).
64. Draper, B. W., McCallum, C. M. & Moens, C. B. Nanos1 Is Required To Maintain Oocyte Production in Adult Zebrafish. *Dev. Biol.* **305**, 589–598 (2007).
65. Gu, X. H. *et al.* Identifying a Major QTL Associated with Salinity Tolerance in Nile Tilapia Using QTL-Seq. *Mar. Biotechnol.* **20**, 98–107 (2018).
