# Supplementary material for: Whole genome re-sequencing reveals recent signatures of selection in three strains of farmed Nile tilapia (Oreochromis niloticus)
Source: Sci Rep. 2020 Jul 13;10:11514. doi: 10.1038/s41598-020-68064-5 (PMC7359307; doi:10.1038/s41598-020-68064-5)
Supplement: Supplementary file 8 — Supplementary table S6 [file 41598_2020_68064_MOESM8_ESM.pdf]

Supplementary information

Whole genome re-sequencing reveals recent signatures of selection in three strains of farmed Nile tilapia (*Oreochromis niloticus*)

María I. Cádiz[12], María E. López[31], Diego Díaz-Domínguez[4], Giovanna Cáceres[12], Grazyella M. Yoshida[1], Daniel Gomez-Uchida[5.6], José M. Yáñez[1,6]*.

[1] Facultad de Ciencias Veterinarias y Pecuarias, Universidad de Chile, Avenida Santa Rosa 11735, 8820808, La Pintana, Santiago, Chile

[2] Programa de Doctorado en Ciencias Silvoagropecuarias y Veterinarias, Campus Sur, Universidad de Chile, Santa Rosa 11315, La Pintana, Santiago, Chile. CP: 8820808.

[3] Department of Animal Breeding and Genetics, Swedish University of Agricultural Sciences, Uppsala, Sweden.

[4] Departamento de Ciencias de la Computación, Universidad de Chile.

[5] Facultad de Ciencias Naturales y Oceanográficas, Universidad de Concepción, Concepción, Chile.

[6] Núcleo Milenio INVASAL, Concepción, Chile

*jmayanez@uchile.cl +56-2 29785533 (Corresponding Author).

Supplementary Table S6. List of all genes detected by FST method.

LG	*Initial pos.*	*Final pos.*	*Fst*	*Gene symbol*	*Gene name*	*Fst pair*
NC_031970.2	1450001	1456151	0.278567	aldh4a1	aldehyde dehydrogenase 4 family member A1	AB
NC_031970.2	1457073	1573914	0.278567	rnf123	E3 ubiquitin-protein ligase RNF123	AB
NC_031970.2	1500334	1511978	0.278567	amigo3	adhesion molecule with Ig like domain 3	AB
NC_031970.2	1577939	1581356	0.278567	LOC100704836	nuclear factor 7%2C brain-like	AB
NC_031970.2	1619478	1700000	0.278567	LOC100705106	metabotropic glutamate receptor 7	AB
NC_031970.2	1811582	1813889	0.278551	LOC100705371	nuclear factor 7%2C ovary-like	AB
NC_031970.2	2018833	2025568	0.279528	LOC109201209	C-type lectin lectoxin-Lio3-like	AB
NC_031970.2	2091314	2100000	0.28043	nicn1	nicolin 1	AB
NC_031970.2	2173449	2175000	0.289934	LOC109196940	E3 ubiquitin-protein ligase TRIM21-like	AB
NC_031970.2	2432598	2475000	0.280473	dag1	dystroglycan	AB
NC_031970.2	2709137	2725000	0.288342	LOC100707129	poly(rC)-binding protein 4	AB
NC_031979.2	39800001	39810365	0.29983	LOC100694109	mannan-binding lectin serine protease 1	AB
NC_031979.2	39812093	39816625	0.29983	LOC100694381	coagulation factor IX	AB
NC_031979.2	39819137	39836154	0.29983	abcf3	ATP binding cassette subfamily F member 3	AB
NC_031979.2	39838716	39858311	0.29983	lrch3	leucine rich repeats and calponin homology domain containing 3%2C	AB
NC_031979.2	39860915	39864953	0.29983	rpl35a	60S ribosomal protein L35a	AB
NC_031979.2	39877706	39897917	0.29983	abcc5	ATP binding cassette subfamily C member 5%2C	AB
NC_031979.2	39901373	39909014	0.29983	LOC100695440	aminopeptidase RNPEPL1	AB
NC_031979.2	39909132	39936159	0.29983	LOC100695702	segment polarity protein dishevelled homolog DVL-3	AB
NC_031979.2	39945101	39964366	0.29983	LOC100696228	matrix metalloproteinase-23	AB
NC_031979.2	39967639	39969647	0.29983	LOC100711345	stress-associated endoplasmic reticulum protein 1	AB
NC_031979.2	39971189	39979524	0.29983	eif2a	eukaryotic translation initiation factor 2A	AB
NC_031979.2	39983942	39987015	0.291507	LOC112842090	translation initiation factor IF-2-like	AB
NC_031979.2	39998789	40006829	0.291507	LOC102076535	P2Y purinoceptor 14	AB
NC_031979.2	40013885	40016120	0.291507	LOC100696762	dual specificity protein phosphatase 14	AB
NC_031979.2	40016000	40023944	0.291507	polr2d	DNA-directed RNA polymerase II subunit RPB4	AB
NC_031979.2	40045898	40047694	0.291507	LOC102078165	olfactory receptor 10A6-like	AB
NC_031979.2	40059391	40060317	0.291507	LOC100697300	olfactory receptor 490-like	AB
NC_031979.2	40091339	40093756	0.291507	LOC100697563	olfactory receptor 142-like	AB
NC_031979.2	40097174	40098151	0.291507	LOC100697835	olfactory receptor 10A3-like	AB
NC_031979.2	40119684	40125000	0.308242	LOC100699398	E3 ubiquitin-protein ligase rififylin	AB
NC_031979.2	40131048	40141628	0.291507	eif4h	eukaryotic translation initiation factor 4H	AB
NC_031979.2	40143954	40150000	0.301402	LOC100699668	anoctamin-7	AB
NC_031979.2	40177319	40198758	0.291507	LOC102076712	scavenger receptor cysteine-rich type 1 protein M130	AB
NC_031979.2	40210824	40215365	0.291507	LOC100699935	P2Y purinoceptor 14	AB

NC_031979.2	40243498	40250000	0.29275	ece2	endothelin converting enzyme 2%2C	AB
NC_031979.2	6608493	6644659	0.294713	eif4g1	eukaryotic translation initiation factor 4 gamma 1	AB
NC_031979.2	6617810	6617876	0.294713	LOC112842299	small nucleolar RNA SNORD66	AB
NC_031979.2	6645206	6658230	0.294713	psmd2	26S proteasome non-ATPase regulatory subunit 2	AB
NC_031979.2	6658669	6670188	0.294713	LOC100705895	putative serine protease K12H4.7	AB
NC_031979.2	6671853	6678815	0.294713	serpine2	glia-derived nexin	AB
NC_031979.2	6739630	6750383	0.279557	wdfy1	WD repeat and FYVE domain containing 1	AB
NC_031979.2	6750883	6769041	0.279557	LOC100699243	dehydrogenase/reductase SDR family member 12	AB
NC_031979.2	6770093	6776142	0.279557	ap1s3	AP-1 complex subunit sigma-3	AB
NC_031979.2	6775867	6779753	0.279557	scg2	secretogranin II	AB
NC_031979.2	6822709	6824825	0.279557	kcne4	potassium voltage-gated channel subfamily E member 4	AB
NC_031979.2	6827694	6850000	0.294713	acsl3	acyl-CoA synthetase long chain family member 3%2C	AB
NC_031979.2	6895408	6900000	0.305999	sgpp2	sphingosine-1-phosphate phosphatase 2	AB
NC_031979.2	6920185	6925000	0.28951	pax3	paired box 3%2C	AB
NC_031981.2	35226398	35233993	0.276478	pfkfb3	6-phosphofructo-2-kinase/fructose-2%2C6-biphosphatase 3%2C	AB
NC_031981.2	35245614	35261911	0.276478	rbm17	RNA binding motif protein 17%2C	AB
NC_031981.2	35261940	35273651	0.276478	il15ra	interleukin 15 receptor subunit alpha%2C	AB
NC_031981.2	35278687	35305539	0.276478	LOC100696399	interleukin-15 receptor subunit alpha	AB
NC_031981.2	35311528	35342572	0.276478	fbh1	F-box DNA helicase 1	AB
NC_031981.2	35343139	35352812	0.276478	nudt5	ADP-sugar pyrophosphatase	AB
NC_031981.2	35351683	35365465	0.276478	cdc123	cell division cycle 123%2C	AB
NC_031981.2	35368251	35400000	0.276478	camk1d	calcium/calmodulin dependent protein kinase ID	AB
NC_031981.2	35462612	35472896	0.291135	ccdc3	coiled-coil domain containing 3	AB
NC_031981.2	35475279	35482056	0.291135	optn	optineurin	AB
NC_031981.2	35482523	35494353	0.291135	mcm10	minichromosome maintenance 10 replication initiation factor%2C	AB
NC_031981.2	35494821	35500000	0.323052	ucma	unique cartilage matrix-associated protein	AB
NC_031981.2	35509896	35516113	0.291135	phyh	phytanoyl-CoA 2-hydroxylase	AB
NC_031981.2	35517049	35525000	0.29638	LOC100690600	cyclin-dependent kinase 17	AB
NC_031981.2	37325001	37334486	0.275763	aldh1l2	aldehyde dehydrogenase 1 family member L2%2C	AB
NC_031981.2	37334646	37338968	0.275763	lg17h12orf45	linkage group 17 C12orf45 homolog	AB
NC_031981.2	37346949	37383399	0.275763	slc41a2	solute carrier family 41 member 2	AB
NC_031981.2	37404844	37490445	0.275763	chst11	carbohydrate sulfotransferase 11	AB
NC_031981.2	37502746	37513626	0.275763	txnrd1	thioredoxin reductase 1%2C	AB
NC_031981.2	37511743	37522324	0.275763	nfyb	nuclear transcription factor Y subunit beta	AB
NC_031981.2	37514048	37517165	0.275763	LOC102079836	zinc finger BED domain-containing protein 1-like	AB
NC_031981.2	37535090	37537188	0.275763	LOC100534419	inward rectifier potassium channel 13	AB
NC_031981.2	37571597	37575000	0.275763	podxl	podocalyxin	AB

NC_031983.2	22464784	22481556	0.275871	dcaf5	DDB1 and CUL4 associated factor 5%2C	AB
NC_031983.2	22483925	22548125	0.275871	pacs2	phosphofurin acidic cluster sorting protein 2	AB
NC_031983.2	22551593	22557364	0.275871	srsf5	serine and arginine rich splicing factor 5%2C	AB
NC_031983.2	22559248	22569804	0.275871	LOC100707081	deubiquitinase DESI2	AB
NC_031983.2	22570741	22574316	0.275871	synj2bp	synaptojanin 2 binding protein%2C	AB
NC_031983.2	22575546	22582657	0.275871	LOC100706816	galectin-3	AB
NC_031983.2	22582444	22591044	0.275871	LOC100706545	glia maturation factor beta	AB
NC_031983.2	22594332	22600883	0.275871	cnih1	cornichon family AMPA receptor auxiliary protein 1%2C	AB
NC_031983.2	22602093	22608094	0.275871	cdkn3	cyclin dependent kinase inhibitor 3	AB
NC_031983.2	22617728	22627429	0.275871	slc39a8	solute carrier family 39 member 8	AB
NC_031983.2	22628094	22630565	0.275871	nat8	N-acetyltransferase 8	AB
NC_031983.2	22647591	22667007	0.275871	LOC100706014	dynactin subunit 1	AB
NC_031983.2	22668234	22682136	0.275871	alms1	ALMS1%2C centrosome and basal body associated protein%2C	AB
NC_031983.2	22691926	22700000	0.275871	kcnk5	potassium channel subfamily K member 5	AB
NC_031983.2	22713809	22717109	0.289561	LOC109195789	mucin-19	AB
NC_031983.2	22738815	22750000	0.293762	LOC100705492	potassium channel subfamily K member 17	AB
NC_031983.2	22755852	22759525	0.295094	LOC100705223	potassium channel subfamily K member 16-like	AB
NC_031983.2	22763945	22775000	0.295094	kif6	kinesin family member 6%2C	AB
NC_031983.2	22865516	22875000	0.299584	LOC100694327	disheveled-associated activator of morphogenesis 2	AB
NC_031983.2	2525001	2542605	0.281726	adck1	aarF domain containing kinase 1%2C	AB
NC_031983.2	2549120	2557224	0.281726	snx6	sorting nexin 6	AB
NC_031983.2	2558670	2567177	0.281726	psma3	proteasome subunit alpha 3	AB
NC_031983.2	2566216	2566295	0.281726	LOC112843212	small nucleolar RNA SNORD53/SNORD92	AB
NC_031983.2	2568288	2607768	0.281726	arid4a	AT-rich interaction domain 4A%2C	AB
NC_031983.2	2697537	2704009	0.281726	LOC100699342	cofilin-2	AB
NC_031983.2	2708809	2743734	0.281726	baz1a	bromodomain adjacent to zinc finger domain 1A%2C	AB
NC_031983.2	2750350	2764217	0.281726	jkamp	JNK1/MAPK8 associated membrane protein	AB
NC_031983.2	2768198	2775000	0.281726	fam241a	family with sequence similarity 241 member A	AB
NC_031983.2	4600001	4793539	0.289155	npas3	neuronal PAS domain protein 3%2C	AB
NC_031983.2	4818030	4850000	0.289155	akap6	A-kinase anchor protein 6	AB
NC_031983.2	5039268	5050000	0.358247	arhgap5	Rho GTPase activating protein 5%2C	AB
NC_031983.2	5128702	5136207	0.39945	nubpl	iron-sulfur protein NUBPL	AB
NC_031983.2	5137326	5148536	0.39945	LOC100692408	histone-lysine N-methyltransferase 2B	AB
NC_031983.2	5183108	5187102	0.39945	yipf6	Yip1 domain family member 6	AB
NC_031983.2	5187516	5191369	0.39945	snapc1	small nuclear RNA activating complex polypeptide 1	AB
NC_031983.2	5192658	5200000	0.414881	syt16	synaptotagmin 16%2C	AB
NC_031983.2	5241818	5250000	0.400526	sgpp1	sphingosine-1-phosphate phosphatase 1	AB

NC_031983.2	5271626	5274116	0.404111	wdr89	WD repeat-containing protein 89	AB
NC_031983.2	5278950	5300000	0.426801	ppp2r5e	protein phosphatase 2 regulatory subunit B'epsilon%2C	AB
NC_031983.2	5359054	5375000	0.438692	kcnh5	potassium voltage-gated channel subfamily H member 5	AB
NC_031983.2	5555297	5562573	0.300539	gphb5	glycoprotein hormone beta 5	AB
NC_031983.2	5648495	5650000	0.389543	syne2	nesprin-2	AB
NC_031983.2	5694519	5700000	0.377338	esr2	estrogen receptor 2	AB
NC_031983.2	5903562	5925000	0.350431	LOC100699141	protein jagged-2	AB
NC_031983.2	6144111	6150000	0.386448	LOC100698875	transcription factor IIIB 90 kDa subunit	AB
NC_031983.2	6233175	6237557	0.315885	LOC100694622	BTB/POZ domain-containing protein 6-B	AB
NC_031983.2	6286840	6297551	0.314018	LOC109196000	transcription factor IIIB 90 kDa subunit	AB
NC_031983.2	6340720	6350000	0.34119	zfyve21	zinc finger FYVE domain-containing protein 21	AB
NC_031983.2	6353066	6375000	0.319421	klc1	kinesin light chain 1	AB
NC_031983.2	6397364	6400000	0.315885	apopt1	apoptogenic 1%2C mitochondrial	AB
NC_031983.2	6401222	6416626	0.290669	bag5	BAG family molecular chaperone regulator 5	AB
NC_031983.2	6456681	6461654	0.290669	ckb	creatine kinase B%2C	AB
NC_031983.2	6462322	6475000	0.321522	mark3	MAP/microtubule affinity-regulating kinase 3	AB
NC_031983.2	6510522	6519626	0.290669	eif5	eukaryotic translation initiation factor 5	AB
NC_031983.2	6519638	6524760	0.290669	vps29	VPS29%2C retromer complex component%2C	AB
NC_031983.2	6526329	6533583	0.290669	LOC100694421	protein FAM216A	AB
NC_031983.2	6546982	6550000	0.306183	LOC100693880	tumor necrosis factor alpha-induced protein 2	AB
NC_031983.2	6568125	6574917	0.290669	LOC109195943	tumor necrosis factor alpha-induced protein 2	AB
NC_031983.2	6577583	6589500	0.290669	LOC109195942	tumor necrosis factor alpha-induced protein 2	AB
NC_031983.2	6577583	6600000	0.290669	LOC100693617	tumor necrosis factor alpha-induced protein 2	AB
NC_031983.2	6800001	6822741	0.28451	LOC109195940	tumor necrosis factor alpha-induced protein 2	AB
NC_031983.2	6851676	6865280	0.28451	LOC100691460	protein LBH	AB
NC_031983.2	6866677	6894204	0.28451	LOC100691190	protein AHNAK2	AB
NC_031983.2	6905527	6910380	0.28451	sertad1	SERTA domain containing 1%2C	AB
NC_031983.2	6915335	6921744	0.28451	cdca4	cell division cycle associated 4%2C	AB
NC_031983.2	6927520	6932522	0.28451	LOC100693443	activator of 90 kDa heat shock protein ATPase homolog 1	AB
NC_031983.2	6932144	6938851	0.28451	LOC102077187	dr1-associated corepressor homolog	AB
NC_031983.2	6942574	6967751	0.28451	LOC100690925	la-related protein 1B	AB
NC_031983.2	6972302	6980075	0.28451	pgrmc2	membrane-associated progesterone receptor component 2	AB
NC_031983.2	6981947	6990344	0.28451	LOC100692897	acetylserotonin O-methyltransferase	AB
NC_031983.2	6991156	7005586	0.28451	LOC100690654	protein Jade-1	AB
NC_031983.2	7007189	7021874	0.28451	nsd2	histone-lysine N-methyltransferase NSD2	AB
NC_031983.2	7023979	7048379	0.28451	letm1	leucine zipper and EF-hand containing transmembrane protein 1%2C	AB
NC_031983.2	7108812	7125000	0.34465	fgfr3	fibroblast growth factor receptor 3	AB

NC_031983.2	7315333	7317930	0.277684	LOC100692627	HRAS-like suppressor 3	AB
NC_031983.2	7332472	7335092	0.277684	foxi3	forkhead box protein I3	AB
NC_031983.2	7369889	7371736	0.277684	LOC100692083	complement factor D	AB
NC_031983.2	7371398	7375000	0.290961	LOC102082980	GTPase IMAP family member 8	AB
NC_031983.2	7376658	7400000	0.287481	lg19h20orf194	linkage group 19 C20orf194 homolog	AB
NC_031983.2	7402978	7409121	0.277684	ky	kyphoscoliosis peptidase	AB
NC_031983.2	7413479	7425000	0.277684	slc4a11	sodium bicarbonate transporter-like protein 11	AB
NC_031983.2	7575001	7590233	0.281043	eif2ak3	eukaryotic translation initiation factor 2 alpha kinase 3	AB
NC_031983.2	7593408	7724317	0.281043	atrn	attractin	AB
NC_031983.2	7734189	7825000	0.281043	gfra4	GDNF family receptor alpha 4%2C	AB
NC_031983.2	8075486	8080062	0.275953	ap5s1	AP-5 complex subunit sigma-1	AB
NC_031983.2	8089420	8096583	0.275953	cdc25b	M-phase inducer phosphatase 2	AB
NC_031983.2	8120161	8121185	0.275953	LOC100710855	homeobox protein notochord-like	AB
NC_031983.2	8124634	8125000	0.281575	LOC100691274	homeobox protein EMX1	AB
NC_031983.2	8136960	8162853	0.275953	sfxn5	sideroflexin 5%2C	AB
NC_031983.2	8163809	8175000	0.275953	LOC100710586	rab11 family-interacting protein 1	AB
NC_031983.2	8230297	8231343	0.291761	LOC102082380	probable G-protein coupled receptor 151	AB
NC_031983.2	8254099	8261535	0.291761	mavs	mitochondrial antiviral signaling protein	AB
NC_031983.2	8262300	8268435	0.291761	LOC100710057	pantothenate kinase 3	AB
NC_031983.2	8269860	8299958	0.291761	loxl3	lysyl oxidase homolog 3	AB
NC_031983.2	8302601	8304329	0.291761	mrps26	28S ribosomal protein S26%2C mitochondrial	AB
NC_031983.2	8305903	8308928	0.291761	LOC102080515	neurofilament light polypeptide	AB
NC_031983.2	8312083	8313897	0.291761	LOC100690744	nanos homolog 1	AB
NC_031983.2	8350364	8370547	0.291761	rab3gap2	rab3 GTPase-activating protein non-catalytic subunit	AB
NC_031983.2	8625001	8631016	0.27632	mthfd1	C-1-tetrahydrofolate synthase%2C cytoplasmic	AB
NC_031983.2	8633885	8634949	0.27632	LOC102077281	putative glycine-rich cell wall structural protein 1	AB
NC_031983.2	8672691	8686268	0.27632	ccdc9b	coiled-coil domain containing 9B%2C	AB
NC_031983.2	8689492	8692150	0.27632	inafm2	InaF motif containing 2	AB
NC_031983.2	8692349	8716539	0.27632	plcb2	1-phosphatidylinositol 4%2C5-bisphosphate phosphodiesterase beta-2	AB
NC_031983.2	8727034	8729210	0.27632	ankrd63	ankyrin repeat domain 63	AB
NC_031983.2	8729276	8746894	0.27632	pak6	p21 (RAC1) activated kinase 6%2C	AB
NC_031983.2	8750172	8754935	0.27632	bub1b	BUB1 mitotic checkpoint serine/threonine kinase B	AB
NC_031983.2	8754951	8759237	0.27632	zfyve19	abscission/NoCut checkpoint regulator	AB
NC_031983.2	8758967	8766425	0.27632	LOC100709168	protein phosphatase 1 regulatory subunit 14B	AB
NC_031983.2	8765099	8778695	0.27632	spint1	kunitz-type protease inhibitor 1	AB
NC_031983.2	8782932	8813783	0.27632	LOC102075671	pleckstrin homology domain-containing family G member 3	AB
NC_031983.2	8815260	8823173	0.27632	LOC100708898	neuroglobin	AB

NC_031983.2	8823028	8825247	0.27632	srp14	signal recognition particle 14	AB
NC_031983.2	8826552	8831437	0.27632	pld4	phospholipase D family member 4%2C	AB
NC_031983.2	8831535	8850087	0.27632	LOC100705500	centrosomal protein of 170 kDa protein B	AB
NC_031983.2	8853917	8875000	0.27632	akt1	AKT serine/threonine kinase 1%2C	AB
NC_031983.2	8891487	8898582	0.278362	LOC100704967	zinc finger and BTB domain-containing protein 18.2	AB
NC_031983.2	8901841	8905751	0.278362	siva1	apoptosis regulatory protein Siva	AB
NC_031983.2	8906016	8910153	0.278362	adssl1	adenylosuccinate synthase like 1	AB
NC_031983.2	8910373	8918968	0.278362	LOC100704696	inverted formin-2	AB
NC_031983.2	8918830	8924770	0.278362	LOC102082255	inverted formin-2	AB
NC_031983.2	8929742	8950000	0.278371	dnajc17	DnaJ heat shock protein family (Hsp40) member C17	AB
NC_031983.2	8952422	8956080	0.278362	LOC102081895	cdc42 effector protein 3	AB
NC_031983.2	8971587	8973902	0.278362	gchfr	GTP cyclohydrolase 1 feedback regulatory protein	AB
NC_031983.2	9005604	9007794	0.278362	cyp1c1	cytochrome P450 1C1	AB
NC_031983.2	9026964	9044735	0.278362	LOC112843138	adhesion G-protein coupled receptor G5-like	AB
NC_031983.2	9048024	9050000	0.278362	LOC106098435	adhesion G-protein coupled receptor G5	AB
NC_031983.2	9075667	9080943	0.279823	LOC102082013	adhesion G-protein coupled receptor G5	AB
NC_031983.2	9103359	9115260	0.279823	bmf	Bcl2 modifying factor%2C	AB
NC_031983.2	9169778	9175000	0.292969	LOC100704430	cysteine-rich motor neuron 1 protein	AB
NC_031970.2	23625001	23625090	0.240534	pfdn5	prefoldin subunit 5	BC
NC_031970.2	23625918	23637624	0.240534	LOC100703415	la-related protein 4	BC
NC_031970.2	23637756	23644865	0.240534	LOC100703691	cyclic AMP-dependent transcription factor ATF-7	BC
NC_031970.2	23646930	23689971	0.240534	LOC100703959	disco-interacting protein 2 homolog B-A	BC
NC_031970.2	23694138	23699897	0.240534	LOC100702880	cyclic AMP-dependent transcription factor ATF-1	BC
NC_031970.2	23699477	23702799	0.240534	LOC100703146	methyltransferase-like protein 7A	BC
NC_031970.2	23703087	23705952	0.240534	LOC100703416	methyltransferase-like protein 7A	BC
NC_031970.2	23713022	23760040	0.240534	LOC100704230	sodium channel protein type 8 subunit alpha	BC
NC_031970.2	23764347	23777882	0.240534	LOC100704499	putative fidgetin-like protein 2	BC
NC_031970.2	23785508	23816992	0.240534	LOC100704768	electroneutral sodium bicarbonate exchanger 1	BC
NC_031970.2	23818421	23825972	0.240534	LOC100705039	tubulin monoglycylase TTLL3	BC
NC_031970.2	23827487	23831213	0.240534	LOC100703692	class E basic helix-loop-helix protein 40	BC
NC_031970.2	23834724	23875000	0.240534	LOC100704142	inositol 1%2C4%2C5-trisphosphate receptor type 1	BC
NC_031970.2	24050001	24122680	0.245159	LOC100706902	low-density lipoprotein receptor-related protein 1	BC
NC_031970.2	24135307	24161279	0.245159	LOC100707164	RNA-binding motif%2C single-stranded-interacting protein 2	BC
NC_031970.2	24165978	24169656	0.242985	LOC100707431	protein lifeguard 2	BC
NC_031970.2	24174918	24186085	0.242985	LOC100707699	nuclear receptor subfamily 1 group D member 1	BC
NC_031970.2	24188689	24237248	0.242985	LOC100707970	retinoic acid receptor gamma	BC
NC_031970.2	24238460	24244847	0.242985	LOC100708241	calcium-binding and coiled-coil domain-containing protein 1	BC

NC_031970.2	24245593	24248939	0.242985	smug1	single-strand selective monofunctional uracil DNA glycosylase	BC
NC_031970.2	24249852	24274074	0.242985	LOC100709046	gastrula zinc finger protein XlCGF57.1	BC
NC_031970.2	24278005	24291577	0.242985	LOC100709320	LIM domain and actin-binding protein 1	BC
NC_031970.2	24292280	24300000	0.245159	LOC100705130	SPRY domain-containing protein 3	BC
NC_031970.2	24315568	24318012	0.242985	LOC100709587	insulin-like growth factor-binding protein 5	BC
NC_031970.2	24320058	24330900	0.242985	LOC100705399	phosphatidylinositol 5-phosphate 4-kinase type-2 gamma	BC
NC_031970.2	24334591	24358990	0.242985	LOC100709858	arf-GAP with GTPase%2C ANK repeat and PH domain-containing protein 1	BC
NC_031970.2	24361636	24374088	0.242985	cdk4	cyclin dependent kinase 4%2C	BC
NC_031970.2	24383563	24393343	0.242985	LOC100705835	E3 ubiquitin-protein ligase MARCH9	BC
NC_031970.2	24394052	24400000	0.242985	mettl1	methyltransferase like 1%2C	BC
NC_031970.2	25150001	25153503	0.242945	rassf5	Ras association domain family member 5%2C	BC
NC_031970.2	25155447	25168577	0.242945	ikbke	inhibitor of nuclear factor kappa B kinase subunit epsilon%2C	BC
NC_031970.2	25169799	25222831	0.242945	srgap2	SLIT-ROBO Rho GTPase activating protein 2%2C	BC
NC_031970.2	25223279	25228222	0.241418	fam72a	family with sequence similarity 72 member A	BC
NC_031970.2	25363459	25400000	0.242945	LOC100712178	forkhead box protein P4	BC
NC_031970.2	25462435	25475000	0.263788	LOC102079463	myoD family inhibitor domain-containing protein	BC
NC_031970.2	25533889	25550000	0.267455	LOC100696335	transcription factor EB	BC
NC_031970.2	25565138	25571583	0.241395	tmem183a	transmembrane protein 183A	BC
NC_031970.2	25574874	25575000	0.280868	LOC100689731	liprin-alpha-2	BC
NC_031970.2	25640809	25643049	0.241395	myog	myogenin	BC
NC_031970.2	25651277	25658755	0.241395	mdm4	MDM4%2C p53 regulator%2C	BC
NC_031970.2	25659719	25675000	0.262928	LOC100696864	sortilin	BC
NC_031970.2	25679275	25683697	0.241395	psma5	proteasome subunit alpha 5	BC
NC_031970.2	25684685	25698070	0.241395	LOC100697129	matrix remodeling-associated protein 8	BC
NC_031970.2	25698352	25700000	0.251016	LOC100690808	period circadian protein homolog 3	BC
NC_031970.2	25715160	25724427	0.241395	vamp3	vesicle associated membrane protein 3	BC
NC_031970.2	25747139	25750000	0.255567	LOC100697400	coiled-coil domain-containing protein 3	BC
NC_031970.2	25769603	25775000	0.263237	LOC100697666	calcium/calmodulin-dependent protein kinase type 1	BC
NC_031970.2	25811085	25822144	0.243377	nek4	NIMA related kinase 4%2C	BC
NC_031970.2	25822412	25823871	0.243377	spcs1	signal peptidase complex subunit 1	BC
NC_031970.2	25824008	25825000	0.247157	glt8d1	glycosyltransferase 8 domain containing 1	BC
NC_031970.2	25829219	25834430	0.243377	gnl3	G protein nucleolar 3	BC
NC_031970.2	25832503	25832586	0.243377	LOC112847018	small nucleolar RNA SNORA47	BC
NC_031970.2	25836329	25841730	0.243377	LOC102076585	zinc finger and SCAN domain-containing protein 22	BC
NC_031971.2	19762034	19762888	0.259217	LOC109202638	spore coat protein SP96-like	BC
NC_031971.2	19765509	19766395	0.259217	LOC109202646	spore coat protein SP96-like	BC
NC_031971.2	19769904	19770824	0.259217	LOC102080116	spore coat protein SP96-like	BC

NC_031971.2	19834545	19836898	0.259217	LOC100709362	resuscitation-promoting factor RpfA	BC
NC_031971.2	19845659	19848292	0.259217	LOC100709631	nischarin	BC
NC_031971.2	19849045	19854092	0.259217	LOC100709902	cyclic nucleotide-gated channel rod photoreceptor subunit alpha	BC
NC_031971.2	19867280	19888355	0.259217	tacr3a	neuromedin-K receptor-like	BC
NC_031971.2	19927523	19936639	0.259217	bdh2	3-hydroxybutyrate dehydrogenase 2%2C	BC
NC_031971.2	19936918	19950502	0.259217	LOC100695067	sodium/hydrogen exchanger 9B2	BC
NC_031971.2	19950478	19954439	0.259217	cisd2	CDGSH iron sulfur domain 2	BC
NC_031971.2	19956390	19976480	0.259217	suclg1	succinate--CoA ligase [ADP/GDP-forming] subunit alpha%2C mitochondrial	BC
NC_031971.2	20260717	20262821	0.290969	LOC106098902	zinc finger BED domain-containing protein 4	BC
NC_031971.2	20352880	20375000	0.301232	ctnna2	catenin alpha 2%2C	BC
NC_031971.2	20474025	20475000	0.304628	lrrtm1	leucine rich repeat transmembrane neuronal 1	BC
NC_031971.2	20785493	20795315	0.284015	adra1d	adrenoceptor alpha 1D%2C	BC
NC_031971.2	20795490	20800000	0.350094	LOC100695334	5-hydroxytryptamine receptor 4-like	BC
NC_031971.2	20844300	20850000	0.323476	mettl14	N6-adenosine-methyltransferase non-catalytic subunit	BC
NC_031971.2	20859537	20875000	0.314748	prss12	neurotrypsin	BC
NC_031971.2	20884433	20900000	0.328308	LOC100695867	bifunctional heparan sulfate N-deacetylase/N-sulfotransferase 4	BC
NC_031971.2	21015585	21025000	0.284015	ugt8	2-hydroxyacylsphingosine 1-beta-galactosyltransferase	BC
NC_031971.2	21051108	21067997	0.246743	spock3	SPARC (osteonectin)%2C cwcv and kazal like domains proteoglycan 3%2C	BC
NC_031971.2	21073685	21075000	0.280745	LOC100689789	choline transporter-like protein 1	BC
NC_031971.2	21093704	21100000	0.280868	LOC100696392	rho GTPase-activating protein 7	BC
NC_031971.2	21186101	21198674	0.24193	lonrf1	LON peptidase N-terminal domain and RING finger protein 1	BC
NC_031971.2	21197768	21200000	0.249326	LOC100696923	calcium-binding protein 2	BC
NC_031971.2	21227195	21250000	0.250465	LOC100690058	double C2-like domain-containing protein beta	BC
NC_031971.2	21282453	21287967	0.24193	serping1	C1 inhibitor precursor	BC
NC_031971.2	21290835	21299147	0.24193	tmem134	transmembrane protein 134	BC
NC_031971.2	21302754	21307181	0.24193	aip	AH receptor-interacting protein	BC
NC_031971.2	21307514	21312310	0.24193	cdk2ap2	cyclin dependent kinase 2 associated protein 2	BC
NC_031971.2	21355114	21358601	0.240639	LOC109202441	serine/threonine-protein kinase pim-2-like	BC
NC_031971.2	21365778	21368852	0.240639	LOC109202442	serine/threonine-protein kinase pim-1-like	BC
NC_031971.2	21376425	21384812	0.240639	LOC106098749	serine/threonine-protein kinase pim-1	BC
NC_031971.2	21385836	21391881	0.240639	LOC102080289	serine/threonine-protein kinase pim-1	BC
NC_031971.2	21394596	21430135	0.240639	gpr83	G protein-coupled receptor 83	BC
NC_031971.2	21442605	21452098	0.240639	ankrd49	ankyrin repeat domain 49%2C	BC
NC_031971.2	21481064	21503762	0.240605	LOC109194342	nucleoporin NUP159	BC
NC_031971.2	21530812	21538855	0.240605	LOC102079525	E3 ubiquitin-protein ligase ZFP91	BC
NC_031971.2	21542912	21579480	0.240605	rbm47	RNA-binding protein 47	BC
NC_031971.2	21594744	21600000	0.240639	LOC102081304	putative methyltransferase NSUN7	BC

NC_031971.2	21616038	21625000	0.241204	apbb2	amyloid beta precursor protein binding family B member 2%2C	BC
NC_031971.2	21624239	21625000	0.241204	LOC102078215	trichohyalin-like	BC
NC_031971.2	21674917	21675000	0.242685	haspin	histone H3 associated protein kinase%2C	BC
NC_031971.2	21687943	21700000	0.240605	LOC100695321	protein FAM184B	BC
NC_031971.2	21723740	21725000	0.241486	ncapg	condensin complex subunit 3	BC
NC_031971.2	22200001	22227871	0.256085	klhl5	kelch like family member 5%2C	BC
NC_031971.2	22236220	22248803	0.256085	fam114a1	family with sequence similarity 114 member A1	BC
NC_031971.2	22253504	22260203	0.248754	klf3	Krueppel-like factor 3	BC
NC_031971.2	22300061	22346230	0.248754	LOC100690481	TBC1 domain family member 1	BC
NC_031971.2	22346708	22358780	0.248754	pgm2	phosphoglucomutase 2%2C	BC
NC_031971.2	22360647	22412826	0.248754	zcchc7	zinc finger CCHC domain-containing protein 7	BC
NC_031971.2	22422003	22450000	0.256085	pax5	paired box 5%2C	BC
NC_031971.2	22488720	22498199	0.248754	melk	maternal embryonic leucine zipper kinase	BC
NC_031974.2	1358045	1363779	0.253034	LOC102083260	zinc finger protein 708-like	BC
NC_031974.2	1448802	1498361	0.253034	LOC102080698	zinc finger protein 100	BC
NC_031974.2	1488863	1500687	0.253034	LOC112847916	zinc finger protein 271-like	BC
NC_031974.2	1513817	1523236	0.253034	LOC109197793	zinc finger protein 271	BC
NC_031974.2	1546591	1559309	0.253034	LOC109197792	zinc finger protein 239-like	BC
NC_031974.2	1564373	1575000	0.253034	LOC102076992	zinc finger protein 726-like	BC
NC_031974.2	1594876	1600000	0.266301	LOC102080789	zinc finger protein 708	BC
NC_031974.2	1612019	1625000	0.294183	LOC100701940	zinc finger protein 729	BC
NC_031974.2	22903440	22909200	0.248155	LOC100691259	beta-1%2C4-galactosyltransferase 1	BC
NC_031974.2	22910240	22918304	0.248155	amer2	APC membrane recruitment protein 2	BC
NC_031974.2	22924985	22935649	0.248155	mtmr6	myotubularin related protein 6	BC
NC_031974.2	22937582	22951047	0.248155	nup58	nucleoporin 58%2C	BC
NC_031974.2	22952193	22961207	0.248155	ropn1l	rhophilin associated tail protein 1 like%2C	BC
NC_031974.2	22966444	22980997	0.248155	LOC100691622	ankyrin repeat domain-containing protein 33B	BC
NC_031974.2	22985975	22997733	0.248155	dap	death associated protein	BC
NC_031974.2	23009899	23050000	0.253424	LOC100692066	catenin delta-2	BC
NC_031974.2	29754311	29802734	0.249732	znf438	zinc finger protein 438	BC
NC_031974.2	29819884	29868139	0.245406	zeb1	zinc finger E-box-binding homeobox 1	BC
NC_031974.2	29890456	29950000	0.259209	arhgap12	Rho GTPase activating protein 12%2C	BC
NC_031974.2	29966499	29975000	0.269262	LOC100706139	kinesin-1 heavy chain	BC
NC_031974.2	29994614	29999500	0.245406	LOC106098166	protein ZBED8-like	BC
NC_031974.2	30008103	30025000	0.245406	LOC100711398	glucose-fructose oxidoreductase domain-containing protein 1	BC
NC_031974.2	32950001	32984972	0.250245	sspo	SCO-spondin	BC
NC_031974.2	32985254	33200000	0.250245	LOC102075990	potassium voltage-gated channel subfamily H member 2	BC

NC_031974.2	33229962	33275000	0.241848	LOC100711676	microtubule-associated protein 4	BC
NC_031974.2	33475001	33486303	0.265389	LOC106098735	NACHT%2C LRR and PYD domains-containing protein 4E-like	BC
NC_031974.2	33475001	33725000	0.265389	LOC112847800	syncytin-A-like	BC
NC_031974.2	33510576	33565936	0.265389	LOC100690049	phospholipid phosphatase-related protein type 4	BC
NC_031974.2	33587517	33713298	0.254598	plppr5	phospholipid phosphatase related 5%2C	BC
NC_031974.2	33763526	33775000	0.281274	snx7	sorting nexin 7	BC
NC_031975.2	33573486	33584467	0.253112	mat2b	methionine adenosyltransferase 2 subunit beta	BC
NC_031975.2	33605730	33613444	0.248001	slc23a1	solute carrier family 23 member 1	BC
NC_031975.2	33620686	33624325	0.248001	pwwp2a	PWWP domain-containing protein 2A	BC
NC_031975.2	33636814	33662177	0.248001	LOC100695173	solute carrier family 22 member 6	BC
NC_031975.2	33665716	33670340	0.248001	LOC100710536	deoxycytidine kinase	BC
NC_031975.2	33674436	33677392	0.248001	pfdn1	prefoldin subunit 1	BC
NC_031975.2	33679689	33696210	0.248001	ttc1	tetratricopeptide repeat domain 1%2C	BC
NC_031975.2	33707754	33750768	0.245382	tmprss15	enteropeptidase	BC
NC_031975.2	33752065	33775000	0.253112	LOC100710555	macrophage colony-stimulating factor 1 receptor 2	BC
NC_031975.2	33787354	33800000	0.267157	LOC100709470	platelet-derived growth factor receptor beta	BC
NC_031975.2	33867756	33875000	0.251166	LOC100699870	gap junction Cx32.2 protein	BC
NC_031975.2	33964404	33967131	0.241599	LOC100692936	complement C1q tumor necrosis factor-related protein 3	BC
NC_031975.2	33971602	33974578	0.241599	LOC106097028	complement C1q tumor necrosis factor-related protein 3	BC
NC_031975.2	34016527	34020462	0.241599	LOC100693484	complement C1q-like protein 4	BC
NC_031979.2	33151954	33155346	0.245643	LOC109194655	serine/threonine-protein phosphatase 6 regulatory ankyrin repeat subunit A-like	BC
NC_031979.2	33156196	33157228	0.245643	LOC109204954	biotinidase-like	BC
NC_031979.2	33162143	33164612	0.245643	LOC109204953	sodium/calcium exchanger 2-like	BC
NC_031979.2	33189981	33191688	0.245643	LOC109204952	sodium/calcium exchanger 2-like	BC
NC_031979.2	33196198	33198438	0.245643	LOC112842087	biotinidase-like	BC
NC_031979.2	33231119	33235663	0.245643	LOC109194398	tubulin alpha chain-like	BC
NC_031979.2	33237936	33257534	0.245643	LOC109204951	echinoderm microtubule-associated protein-like 1	BC
NC_031979.2	33269086	33280460	0.245643	prdm10	PR domain zinc finger protein 10	BC
NC_031979.2	33288899	33340975	0.245643	aplp2	amyloid beta precursor like protein 2%2C	BC
NC_031979.2	33341696	33353878	0.245643	st14	suppression of tumorigenicity 14	BC
NC_031979.2	33354888	33367842	0.245643	dhx34	DExH-box helicase 34	BC
NC_031979.2	33367978	33375000	0.245643	spred3	sprouty related EVH1 domain containing 3	BC
NC_031981.2	11250001	11363157	0.244749	plxna4	plexin A4%2C	BC
NC_031981.2	11398064	11473565	0.244749	LOC100697147	MICOS complex subunit MIC19	BC
NC_031981.2	11478811	11500000	0.244749	exoc4	exocyst complex component 4	BC
NC_031981.2	11616278	11631435	0.251779	lrguk	leucine rich repeats and guanylate kinase domain containing%2C	BC
NC_031981.2	11657440	11675000	0.251779	LOC100708618	collagen alpha-1(VII) chain	BC

NC_031981.2	11723046	11725000	0.263686	LOC100696616	urocortin-3	BC
NC_031981.2	11727178	11734889	0.260732	copg2	coatomer protein complex subunit gamma 2	BC
NC_031981.2	11735031	11739385	0.260732	mest	mesoderm specific transcript%2C	BC
NC_031981.2	11741721	11750000	0.272363	atp2b1	ATPase plasma membrane Ca2+ transporting 1%2C	BC
NC_031981.2	11932802	11950000	0.267201	st8sia1	ST8 alpha-N-acetyl-neuraminide alpha-2%2C8-sialyltransferase 1	BC
NC_031981.2	11959972	11962771	0.244046	LOC100694855	troponin I%2C slow skeletal muscle	BC
NC_031981.2	11980775	12000000	0.249129	iqsec3	IQ motif and SEC7 domain-containing protein 3	BC
NC_031981.2	12101533	12115389	0.245592	LOC100708075	sodium- and chloride-dependent GABA transporter 1	BC
NC_031981.2	12124052	12140707	0.245592	LOC100707806	sodium- and chloride-dependent GABA transporter 2	BC
NC_031981.2	12141861	12148840	0.245592	LOC100707536	poly [ADP-ribose] polymerase 11	BC
NC_031981.2	12149226	12151210	0.245592	LOC100707277	poly [ADP-ribose] polymerase 11-like	BC
NC_031981.2	12157866	12221803	0.245592	LOC100707010	transmembrane and TPR repeat-containing protein 2	BC
NC_031981.2	12229148	12245919	0.245592	mettl25	methyltransferase like 25%2C	BC
NC_031981.2	12246216	12248242	0.245592	ccdc59	coiled-coil domain containing 59	BC
NC_031981.2	12248484	12250000	0.25972	LOC100706208	ras and EF-hand domain-containing protein homolog	BC
NC_031981.2	12259428	12273058	0.245592	LOC100705938	protein arginine N-methyltransferase 8-B	BC
NC_031981.2	12276501	12278892	0.245592	LOC100705677	E3 ubiquitin-protein ligase TRIM39	BC
NC_031981.2	12281855	12300000	0.253333	LOC100694590	tetraspanin-11	BC
NC_031981.2	12387088	12400000	0.255072	LOC100694323	semaphorin-3A	BC
NC_031981.2	12436350	12450000	0.266095	LOC100705413	protein piccolo	BC
NC_031981.2	12493877	12575000	0.242322	LOC100705144	voltage-dependent calcium channel subunit alpha-2/delta-1	BC
NC_031981.2	12825001	12871937	0.250122	snd1	staphylococcal nuclease and tudor domain containing 1%2C	BC
NC_031981.2	13120147	13125000	0.259444	LOC100704338	leptin-B-like	BC
NC_031981.2	13143536	13150000	0.266983	prrt4	proline rich transmembrane protein 4	BC
NC_031981.2	13164831	13175000	0.246541	LOC100693341	inosine-5'-monophosphate dehydrogenase 1b	BC
NC_031981.2	13177483	13215380	0.240527	LOC109194165	sortilin	BC
NC_031981.2	13215883	13222605	0.240527	fam3c	family with sequence similarity 3 member C	BC
NC_031981.2	13281265	13289952	0.240527	btg1	BTG anti-proliferation factor 1	BC
NC_031981.2	13390993	13410890	0.240527	dcn	decorin	BC
NC_031981.2	13421991	13425000	0.240527	lum	lumican	BC
NC_031981.2	13431667	13437420	0.241728	kera	keratocan	BC
NC_031981.2	13472927	13475000	0.247704	LOC106098228	probable E3 ubiquitin-protein ligase DTX2	BC
NC_031981.2	13476798	13490643	0.241728	scyl2	SCY1 like pseudokinase 2%2C	BC
NC_031981.2	13491420	13496336	0.241728	depdc4	DEP domain containing 4	BC
NC_031981.2	13496649	13500000	0.241728	tcp11l2	T-complex protein 11-like protein 2	BC
NC_031981.2	13505750	13525000	0.242409	apaf1	apoptotic peptidase activating factor 1	BC
NC_031981.2	13575087	13600000	0.24605	anks1b	ankyrin repeat and sterile alpha motif domain containing 1B%2C	BC

NC_031983.2	6144111	6266139	0.243854	LOC100698875	transcription factor IIIB 90 kDa subunit	BC
NC_031983.2	6233175	6237557	0.243854	LOC100694622	BTB/POZ domain-containing protein 6-B	BC
NC_031983.2	6286840	6297551	0.243854	LOC109196000	transcription factor IIIB 90 kDa subunit	BC
NC_031983.2	6851676	6865280	0.249764	LOC100691460	protein LBH	BC
NC_031983.2	6866677	6894204	0.249764	LOC100691190	protein AHNAK2	BC
NC_031983.2	6905527	6910380	0.247149	sertad1	SERTA domain containing 1%2C	BC
NC_031983.2	6915335	6921744	0.247149	cdca4	cell division cycle associated 4%2C	BC
NC_031983.2	6927520	6932522	0.247149	LOC100693443	activator of 90 kDa heat shock protein ATPase homolog 1	BC
NC_031983.2	6932144	6938851	0.247149	LOC102077187	dr1-associated corepressor homolog	BC
NC_031983.2	6942574	6967751	0.247149	LOC100690925	la-related protein 1B	BC
NC_031983.2	6972302	6980075	0.247149	pgrmc2	membrane-associated progesterone receptor component 2	BC
NC_031983.2	6981947	6990344	0.247149	LOC100692897	acetylserotonin O-methyltransferase	BC
NC_031983.2	6991156	7005586	0.247149	LOC100690654	protein Jade-1	BC
NC_031983.2	7007189	7021874	0.247149	nsd2	histone-lysine N-methyltransferase NSD2	BC
NC_031983.2	7023979	7048379	0.247149	letm1	leucine zipper and EF-hand containing transmembrane protein 1%2C	BC
NC_031983.2	7108812	7125000	0.247149	fgfr3	fibroblast growth factor receptor 3	BC
NC_031983.2	8125001	8134376	0.257545	LOC100691274	homeobox protein EMX1	BC
NC_031983.2	8136960	8162853	0.257545	sfxn5	sideroflexin 5%2C	BC
NC_031983.2	8163809	8202789	0.257545	LOC100710586	rab11 family-interacting protein 1	BC
NC_031983.2	8230297	8231343	0.257545	LOC102082380	probable G-protein coupled receptor 151	BC
NC_031983.2	8254099	8261535	0.257545	mavs	mitochondrial antiviral signaling protein	BC
NC_031983.2	8262300	8268435	0.257545	LOC100710057	pantothenate kinase 3	BC
NC_031983.2	8269860	8299958	0.257545	loxl3	lysyl oxidase homolog 3	BC
NC_031983.2	8302601	8304329	0.257545	mrps26	28S ribosomal protein S26%2C mitochondrial	BC
NC_031983.2	8305903	8308928	0.257545	LOC102080515	neurofilament light polypeptide	BC
NC_031983.2	8312083	8313897	0.257545	LOC100690744	nanos homolog 1	BC
NC_031983.2	8350364	8370547	0.257545	rab3gap2	rab3 GTPase-activating protein non-catalytic subunit	BC
NC_031966.2	27475001	27532538	0.25981	frmpd3	FERM and PDZ domain containing 3%2C	CA
NC_031966.2	27543111	27546975	0.254147	prps1	phosphoribosyl pyrophosphate synthetase 1%2C	CA
NC_031966.2	27549757	27553073	0.254147	slc25a53	solute carrier family 25 member 53	CA
NC_031966.2	27561471	27571751	0.254147	LOC100690556	SLAIN motif-containing protein-like	CA
NC_031966.2	27572162	27578728	0.254147	znf711	zinc finger protein 711	CA
NC_031966.2	27584022	27587733	0.254147	LOC106096447	BTB/POZ domain-containing protein KCTD12	CA
NC_031966.2	27593581	27601295	0.254147	LOC100690288	proline-rich receptor-like protein kinase PERK13	CA
NC_031966.2	27640196	27643956	0.254147	LOC100698667	rho-related GTP-binding protein RhoG	CA
NC_031966.2	27649260	27659385	0.254147	LOC102076457	FH2 domain-containing protein 1	CA
NC_031966.2	27681739	27694605	0.254147	ogt	O-linked N-acetylglucosamine (GlcNAc) transferase%2C	CA

NC_031966.2	27695706	27705835	0.254147	LOC100690020	acidic repeat-containing protein	CA
NC_031966.2	27709051	27712606	0.254147	LOC112841944	olfactory receptor 2T3-like	CA
NC_031966.2	27712726	27715748	0.254147	LOC100689752	caltractin	CA
NC_031966.2	27717292	27723423	0.254147	nsdhl	NAD(P) dependent steroid dehydrogenase-like	CA
NC_031966.2	27742012	27748387	0.254147	fut11	alpha-(1%2C3)-fucosyltransferase 11	CA
NC_031966.2	27747535	27750000	0.254147	LOC100697336	ras-related protein Rab-9B	CA
NC_031966.2	27752465	27763025	0.290179	LOC100697062	myelin proteolipid protein	CA
NC_031966.2	27764513	27767799	0.290179	LOC102077653	protocadherin-20	CA
NC_031966.2	27768903	27772663	0.290179	LOC100696796	regulator of cell cycle RGCC	CA
NC_031966.2	27787345	27800000	0.306803	nlgn3	neuroligin 3%2C	CA
NC_031966.2	28028735	28050000	0.268526	LOC100695922	glutamate receptor 3	CA
NC_031966.2	28157911	28170303	0.286976	eda	ectodysplasin A%2C	CA
NC_031966.2	28170578	28173314	0.286976	LOC100695394	tumor necrosis factor ligand superfamily member 13B	CA
NC_031966.2	28173428	28175000	0.287349	LOC100695129	leucine-rich repeat-containing protein 32	CA
NC_031966.2	28184872	28188597	0.286976	LOC102079731	gap junction beta-1 protein	CA
NC_031966.2	28188961	28192214	0.286976	LOC100694863	gap junction alpha-3 protein	CA
NC_031966.2	28197602	28199689	0.286976	vma21	VMA21%2C vacuolar ATPase assembly factor	CA
NC_031966.2	28213049	28223814	0.306785	LOC100694598	melatonin receptor type 1B	CA
NC_031966.2	28231079	28250000	0.328164	neurl1b	E3 ubiquitin-protein ligase NEURL1B	CA
NC_031966.2	28269649	28272971	0.257527	dusp1	dual specificity phosphatase 1	CA
NC_031966.2	28277554	28297275	0.257527	ergic1	endoplasmic reticulum-Golgi intermediate compartment protein 1	CA
NC_031966.2	28297202	28300000	0.31738	flt4	fms related tyrosine kinase 4%2C	CA
NC_031966.2	28313194	28325000	0.317868	LOC100693249	neuropeptide Y receptor type 2	CA
NC_031966.2	28351204	28357304	0.257527	LOC100693524	zinc-binding protein A33	CA
NC_031966.2	28366450	28371467	0.257527	prelid1	PRELI domain containing 1	CA
NC_031966.2	28371657	28375000	0.318794	mxd3	MAX dimerization protein 3%2C	CA
NC_031966.2	28376692	28391315	0.257527	fam193b	family with sequence similarity 193 member B%2C	CA
NC_031966.2	28392335	28400000	0.319912	ddx41	DEAD-box helicase 41	CA
NC_031966.2	28400739	28406143	0.257527	dok3	docking protein 3	CA
NC_031966.2	28406149	28407761	0.257527	LOC100710497	neurogenin-2	CA
NC_031966.2	32050001	32051735	0.26795	LOC100710804	integrator complex subunit 6	CA
NC_031966.2	32051904	32055552	0.26795	mospd1	motile sperm domain containing 1	CA
NC_031966.2	32058613	32068458	0.26795	fam122b	family with sequence similarity 122B%2C	CA
NC_031966.2	32065590	32065746	0.26795	LOC112842510	small nucleolar RNA U109	CA
NC_031966.2	32070599	32079075	0.26795	hprt1	hypoxanthine phosphoribosyltransferase 1	CA
NC_031966.2	32083543	32094483	0.26795	phf6	PHD finger protein 6	CA
NC_031966.2	32093973	32102942	0.26795	LOC100696750	calcium-binding protein 39	CA

NC_031966.2	32109179	32300000	0.26795	LOC102075906	glypican-3	CA
NC_031966.2	32320256	32350000	0.2634	LOC100696204	glypican-6	CA
NC_031966.2	32353321	32369172	0.259654	LOC100696469	bromodomain-containing protein 8	CA
NC_031966.2	32369414	32371079	0.259654	LOC100696738	DNA damage-inducible transcript 4-like protein	CA
NC_031966.2	32375470	32387439	0.259654	tmem173	stimulator of interferon genes protein	CA
NC_031966.2	32393456	32400000	0.259654	LOC100697276	serine/threonine-protein phosphatase 2B catalytic subunit alpha	CA
NC_031970.2	6500001	6552158	0.251409	ppp1r16b	protein phosphatase 1 regulatory inhibitor subunit 16B	CA
NC_031970.2	6569782	6593841	0.251409	arhgap40	Rho GTPase activating protein 40%2C	CA
NC_031970.2	6624201	6628934	0.251409	LOC100693191	vesicular inhibitory amino acid transporter	CA
NC_031970.2	6629890	6637093	0.251409	LOC100694808	serine/threonine-protein kinase Aurora-2	CA
NC_031970.2	6639297	6645719	0.251409	prelid3b	PRELI domain containing 3B	CA
NC_031970.2	6646943	6650751	0.251409	LOC100698256	tubulin beta chain	CA
NC_031970.2	6657447	6693349	0.251409	LOC100710082	guanine nucleotide-binding protein G(s) subunit alpha	CA
NC_031970.2	6697149	6709814	0.251409	ipo9	importin 9	CA
NC_031970.2	6712285	6722509	0.251409	shisa4	protein shisa-4	CA
NC_031970.2	6724410	6731984	0.251409	lmod1	leiomodin 1	CA
NC_031970.2	6732852	6738327	0.251409	timm17a	mitochondrial import inner membrane translocase subunit Tim17-A	CA
NC_031970.2	6739382	6750000	0.251409	LOC100692376	ras-related protein Rap-1A	CA
NC_031970.2	6794196	6800000	0.26446	inka2	PAK4-inhibitor INKA2	CA
NC_031970.2	8492310	8496182	0.258601	LOC109202229	N-acetyltransferase 8	CA
NC_031970.2	8528116	8529486	0.258601	LOC100693555	N-acetyltransferase 8-like	CA
NC_031970.2	8540426	8543267	0.258601	LOC100703648	adenosine receptor A3	CA
NC_031970.2	8570390	8700000	0.258601	LOC100704187	solute carrier family 12 member 5	CA
NC_031970.2	8746596	8750000	0.276614	ncoa5	nuclear receptor coactivator 5	CA
NC_031970.2	8757252	8762496	0.26732	LOC100694535	F-actin-capping protein subunit alpha-1	CA
NC_031970.2	8764201	8775000	0.270177	LOC100704454	putative helicase mov-10-B.1	CA
NC_031970.2	8779079	8795510	0.26732	LOC100694803	rho-related GTP-binding protein RhoA-D	CA
NC_031970.2	8798499	8800000	0.287793	LOC100704721	protein phosphatase 1H	CA
NC_031970.2	8827772	8850000	0.282391	LOC100704992	protein FAM19A2	CA
NC_031971.2	19100001	19106317	0.26719	LOC100698506	N-acyl-aromatic-L-amino acid amidohydrolase (carboxylate-forming) B	CA
NC_031971.2	19112139	19117047	0.26719	tacr3b	neuromedin-K receptor-like	CA
NC_031971.2	19119421	19121908	0.26719	LOC100709532	ecto-ADP-ribosyltransferase 4	CA
NC_031971.2	19123000	19128461	0.26719	primpol	DNA-directed primase/polymerase protein	CA
NC_031971.2	19128542	19147977	0.26719	LOC100709803	long-chain-fatty-acid--CoA ligase 1	CA
NC_031971.2	19153935	19156329	0.26052	helt	hairy and enhancer of split-related protein HELT	CA
NC_031971.2	19182706	19184955	0.257826	slc25a4	ADP/ATP translocase 1	CA
NC_031971.2	19185343	19194057	0.257826	cfap97	cilia and flagella associated protein 97%2C	CA

NC_031971.2	19195822	19201851	0.257826	ufsp2	UFM1 specific peptidase 2%2C	CA
NC_031971.2	19210632	19221243	0.257826	gucy1a1	guanylate cyclase 1 soluble subunit alpha 1	CA
NC_031971.2	19222091	19241452	0.257826	gucy1b1	guanylate cyclase 1 soluble subunit beta 1	CA
NC_031971.2	19245641	19246781	0.257826	fabp2	fatty acid binding protein 2	CA
NC_031971.2	19246879	19273124	0.257826	LOC100699853	inactive ubiquitin carboxyl-terminal hydrolase 53	CA
NC_031971.2	19276647	19280883	0.257826	LOC100711322	myozenin-2	CA
NC_031971.2	19281766	19291401	0.257826	LOC102079281	synaptopodin-2	CA
NC_031971.2	19298783	19303338	0.257826	LOC100700121	solute carrier family 22 member 7	CA
NC_031971.2	19303405	19314624	0.257826	LOC100700394	solute carrier family 22 member 7	CA
NC_031971.2	19316471	19321073	0.257826	LOC102078086	solute carrier family 22 member 7	CA
NC_031971.2	19321323	19326566	0.257826	LOC109202510	pre-mRNA-splicing factor ATP-dependent RNA helicase DHX15	CA
NC_031971.2	19327076	19328073	0.257826	LOC109202456	solute carrier family 22 member 7-like	CA
NC_031971.2	19329911	19335004	0.257826	LOC106098904	solute carrier family 22 member 7	CA
NC_031971.2	19335142	19345204	0.257826	LOC100706682	pre-mRNA-splicing factor ATP-dependent RNA helicase DHX15	CA
NC_031971.2	19346822	19348350	0.257826	sod3	extracellular superoxide dismutase [Cu-Zn]	CA
NC_031971.2	19347872	19350000	0.26719	LOC100691566	coiled-coil domain-containing protein 149	CA
NC_031971.2	19358931	19364958	0.25709	LOC100691835	leucine-rich repeat LGI family member 2	CA
NC_031971.2	19393293	19400000	0.26052	mttp	microsomal triglyceride transfer protein	CA
NC_031971.2	19413256	19424483	0.251163	LOC100707215	tetraspanin-5	CA
NC_031971.2	19426821	19442596	0.251163	acer2	alkaline ceramidase 2	CA
NC_031971.2	19444583	19449023	0.251163	rps6	40S ribosomal protein S6	CA
NC_031971.2	19454131	19456861	0.251163	LOC100692918	perilipin-2	CA
NC_031971.2	19456977	19470318	0.251163	LOC100707927	WD40 repeat-containing protein SMU1	CA
NC_031971.2	19470146	19475000	0.274657	LOC100708200	dnaJ homolog subfamily A member 1	CA
NC_031971.2	19478657	19482546	0.251163	aptx	aprataxin	CA
NC_031971.2	19484825	19487834	0.251163	LOC100693190	dentin sialophosphoprotein	CA
NC_031971.2	19491147	19498409	0.251163	LOC100693466	SPARC-like protein 1	CA
NC_031971.2	19503630	19506471	0.251163	LOC100693995	class E vacuolar protein-sorting machinery protein HSE1-like	CA
NC_031971.2	19614181	19615065	0.251163	LOC109202643	spore coat protein SP96-like	CA
NC_031971.2	19620281	19621195	0.251163	LOC102079162	spore coat protein SP65-like	CA
NC_031971.2	19748878	19749901	0.251667	LOC109202652	spore coat protein SP96-like	CA
NC_031971.2	19762034	19762888	0.251667	LOC109202638	spore coat protein SP96-like	CA
NC_031971.2	19765509	19766395	0.251667	LOC109202646	spore coat protein SP96-like	CA
NC_031971.2	19769904	19770824	0.251667	LOC102080116	spore coat protein SP96-like	CA
NC_031971.2	19834545	19836898	0.251667	LOC100709362	resuscitation-promoting factor RpfA	CA
NC_031971.2	19845659	19848292	0.251667	LOC100709631	nischarin	CA
NC_031971.2	19849045	19854092	0.251667	LOC100709902	cyclic nucleotide-gated channel rod photoreceptor subunit alpha	CA

NC_031971.2	19867280	19888355	0.251667	tacr3a	neuromedin-K receptor-like	CA
NC_031971.2	19927523	19936639	0.274695	bdh2	3-hydroxybutyrate dehydrogenase 2%2C	CA
NC_031971.2	19936918	19950000	0.274695	LOC100695067	sodium/hydrogen exchanger 9B2	CA
NC_031971.2	19950478	19954439	0.286152	cisd2	CDGSH iron sulfur domain 2	CA
NC_031971.2	19956390	19975000	0.286152	suclg1	succinate--CoA ligase [ADP/GDP-forming] subunit alpha%2C mitochondrial	CA
NC_031971.2	20260717	20262821	0.373816	LOC106098902	zinc finger BED domain-containing protein 4	CA
NC_031971.2	20352880	20375000	0.385564	ctnna2	catenin alpha 2%2C	CA
NC_031971.2	20474025	20475000	0.398559	lrrtm1	leucine rich repeat transmembrane neuronal 1	CA
NC_031971.2	20785493	20795315	0.348205	adra1d	adrenoceptor alpha 1D%2C	CA
NC_031971.2	20795490	20800000	0.373742	LOC100695334	5-hydroxytryptamine receptor 4-like	CA
NC_031971.2	20844300	20850000	0.348205	mettl14	N6-adenosine-methyltransferase non-catalytic subunit	CA
NC_031971.2	20859537	20875000	0.364436	prss12	neurotrypsin	CA
NC_031971.2	20884433	20900000	0.374249	LOC100695867	bifunctional heparan sulfate N-deacetylase/N-sulfotransferase 4	CA
NC_031971.2	21015585	21025000	0.368492	ugt8	2-hydroxyacylsphingosine 1-beta-galactosyltransferase	CA
NC_031971.2	21051108	21067997	0.373033	spock3	SPARC (osteonectin)%2C cwcv and kazal like domains proteoglycan 3%2C	CA
NC_031971.2	21073685	21075000	0.379108	LOC100689789	choline transporter-like protein 1	CA
NC_031971.2	21093704	21100000	0.381779	LOC100696392	rho GTPase-activating protein 7	CA
NC_031971.2	21186101	21198674	0.355566	lonrf1	LON peptidase N-terminal domain and RING finger protein 1	CA
NC_031971.2	21197768	21200000	0.373491	LOC100696923	calcium-binding protein 2	CA
NC_031971.2	21227195	21250000	0.374988	LOC100690058	double C2-like domain-containing protein beta	CA
NC_031971.2	21282453	21287967	0.355566	serping1	C1 inhibitor precursor	CA
NC_031971.2	21290835	21299147	0.355566	tmem134	transmembrane protein 134	CA
NC_031971.2	21302754	21307181	0.355566	aip	AH receptor-interacting protein	CA
NC_031971.2	21307514	21312310	0.355566	cdk2ap2	cyclin dependent kinase 2 associated protein 2	CA
NC_031971.2	21342210	21345677	0.355566	LOC109202440	serine/threonine-protein kinase pim-1	CA
NC_031971.2	21355114	21358601	0.355566	LOC109202441	serine/threonine-protein kinase pim-2-like	CA
NC_031971.2	21365778	21368852	0.355566	LOC109202442	serine/threonine-protein kinase pim-1-like	CA
NC_031971.2	21376425	21384812	0.355566	LOC106098749	serine/threonine-protein kinase pim-1	CA
NC_031971.2	21385836	21391881	0.355566	LOC102080289	serine/threonine-protein kinase pim-1	CA
NC_031971.2	21394596	21400000	0.355566	gpr83	G protein-coupled receptor 83	CA
NC_031971.2	21442605	21450000	0.361299	ankrd49	ankyrin repeat domain 49%2C	CA
NC_031971.2	21481064	21500000	0.368137	LOC109194342	nucleoporin NUP159	CA
NC_031971.2	21530812	21538855	0.373114	LOC102079525	E3 ubiquitin-protein ligase ZFP91	CA
NC_031971.2	21542912	21550000	0.373114	rbm47	RNA-binding protein 47	CA
NC_031971.2	21594744	21600000	0.382515	LOC102081304	putative methyltransferase NSUN7	CA
NC_031971.2	21616038	21625000	0.385407	apbb2	amyloid beta precursor protein binding family B member 2%2C	CA
NC_031971.2	21624239	21625000	0.385407	LOC102078215	trichohyalin-like	CA

NC_031971.2	21674917	21675000	0.386938	haspin	histone H3 associated protein kinase%2C	CA
NC_031971.2	21687943	21700000	0.385883	LOC100695321	protein FAM184B	CA
NC_031971.2	21723740	21725000	0.386643	ncapg	condensin complex subunit 3	CA
NC_031971.2	21742358	21750000	0.384357	lcorl	ligand dependent nuclear receptor corepressor like%2C	CA
NC_031971.2	21883711	21900000	0.382075	slit2	slit homolog 2 protein	CA
NC_031971.2	21983356	21995190	0.261327	LOC100694526	intelectin	CA
NC_031971.2	21997670	22000000	0.330956	LOC100693983	intelectin	CA
NC_031971.2	22010686	22013722	0.261327	LOC100693716	D(1A) dopamine receptor-like	CA
NC_031971.2	22021056	22025000	0.32287	LOC100693455	neuronal acetylcholine receptor subunit alpha-9-I	CA
NC_031971.2	22032577	22038104	0.261327	rhoh	ras homolog family member H	CA
NC_031971.2	22040317	22050000	0.309867	n4bp2	NEDD4 binding protein 2%2C	CA
NC_031971.2	22054687	22075000	0.300942	pds5a	PDS5 cohesin associated factor A%2C	CA
NC_031971.2	22081734	22088520	0.261327	ube2k	ubiquitin conjugating enzyme E2 K%2C	CA
NC_031971.2	22087903	22100000	0.288772	smim14	small integral membrane protein 14	CA
NC_031971.2	22102343	22110003	0.261327	map9	microtubule associated protein 9%2C	CA
NC_031971.2	22113114	22120147	0.261327	ugdh	UDP-glucose 6-dehydrogenase	CA
NC_031971.2	22120200	22125000	0.272584	lias	lipoic acid synthetase	CA
NC_031971.2	22127440	22133221	0.261327	rpl9	60S ribosomal protein L9	CA
NC_031971.2	22134879	22145004	0.261327	rfc1	replication factor C subunit 1	CA
NC_031971.2	22146022	22150000	0.261327	wdr19	WD repeat domain 19%2C	CA
NC_031971.2	22200001	22227871	0.251063	klhl5	kelch like family member 5%2C	CA
NC_031971.2	22236220	22248803	0.251063	fam114a1	family with sequence similarity 114 member A1	CA
NC_031971.2	22253504	22260203	0.251063	klf3	Krueppel-like factor 3	CA
NC_031971.2	22300061	22346230	0.251063	LOC100690481	TBC1 domain family member 1	CA
NC_031971.2	22346708	22358780	0.251063	pgm2	phosphoglucomutase 2%2C	CA
NC_031971.2	22360647	22412826	0.251063	zcchc7	zinc finger CCHC domain-containing protein 7	CA
NC_031971.2	22422003	22450000	0.251063	pax5	paired box 5%2C	CA
NC_031973.2	5874717	5882454	0.257603	LOC100707365	ORM1-like protein 3	CA
NC_031973.2	5961356	6100000	0.257603	LOC100702192	thyroid hormone receptor alpha-B	CA
NC_031973.2	6137935	6147793	0.252763	dhx8	ATP-dependent RNA helicase DHX8	CA
NC_031973.2	6155433	6175000	0.261392	etv4	ETS translocation variant 4	CA
NC_031973.2	6260821	6271043	0.253867	meox1	homeobox protein MOX-1	CA
NC_031980.2	37800001	37836769	0.251458	LOC100694711	receptor-type tyrosine-protein phosphatase F	CA
NC_031980.2	37901403	37915605	0.251458	hyi	hydroxypyruvate isomerase (putative)%2C	CA
NC_031980.2	37920137	37929753	0.251458	LOC109194886	KICSTOR complex protein SZT2	CA
NC_031980.2	37981140	37983299	0.251458	LOC109199849	coiled-coil domain-containing protein 106-like	CA
NC_031980.2	37996761	38006331	0.251458	LOC100703129	sorting nexin-9	CA

NC_031980.2	38047974	38050000	0.251458	LOC109199852	NLR family CARD domain-containing protein 3-like	CA
NC_031980.2	38105991	38116678	0.270709	LOC100703676	atypical chemokine receptor 3	CA
NC_031980.2	38131112	38141971	0.283569	LOC100702862	melanophilin	CA
NC_031981.2	13500001	13504821	0.253049	tcp11l2	T-complex protein 11-like protein 2	CA
NC_031981.2	13505750	13553800	0.253049	apaf1	apoptotic peptidase activating factor 1	CA
NC_031981.2	13575087	13750000	0.253049	anks1b	ankyrin repeat and sterile alpha motif domain containing 1B%2C	CA
NC_031981.2	13837618	13870172	0.251126	uhrf1bp1l	UHRF1 binding protein 1 like%2C	CA
NC_031981.2	13872810	13875786	0.251126	ckap4	cytoskeleton associated protein 4	CA
NC_031981.2	13877108	13883463	0.251126	ikbip	IKBKB interacting protein%2C	CA
NC_031981.2	13884618	13896939	0.251126	arfgap3	ADP ribosylation factor GTPase activating protein 3%2C	CA
NC_031981.2	13898412	13919434	0.251126	pacsin2	protein kinase C and casein kinase substrate in neurons 2%2C	CA
NC_031981.2	13921483	13925000	0.251126	ttll1	probable tubulin polyglutamylase TTLL1	CA
NC_031981.2	35226398	35233993	0.255845	pfkfb3	6-phosphofructo-2-kinase/fructose-2%2C6-biphosphatase 3%2C	CA
NC_031981.2	35245614	35261911	0.255845	rbm17	RNA binding motif protein 17%2C	CA
NC_031981.2	35261940	35273651	0.255845	il15ra	interleukin 15 receptor subunit alpha%2C	CA
NC_031981.2	35278687	35305539	0.252236	LOC100696399	interleukin-15 receptor subunit alpha	CA
NC_031981.2	35311528	35342572	0.252236	fbh1	F-box DNA helicase 1	CA
NC_031981.2	35343139	35352812	0.252236	nudt5	ADP-sugar pyrophosphatase	CA
NC_031981.2	35351683	35365465	0.252236	cdc123	cell division cycle 123%2C	CA
NC_031981.2	35368251	35400000	0.268307	camk1d	calcium/calmodulin dependent protein kinase ID	CA
NC_031981.2	35462612	35472896	0.252236	ccdc3	coiled-coil domain containing 3	CA
NC_031981.2	35475279	35482056	0.252236	optn	optineurin	CA
NC_031981.2	35482523	35494353	0.252236	mcm10	minichromosome maintenance 10 replication initiation factor%2C	CA
NC_031981.2	35494821	35500000	0.271054	ucma	unique cartilage matrix-associated protein	CA
NC_031981.2	35509896	35516113	0.252236	phyh	phytanoyl-CoA 2-hydroxylase	CA
NC_031981.2	35517049	35525000	0.252236	LOC100690600	cyclin-dependent kinase 17	CA

LG: Linkage group
initial pos: initial position
final pos: final position
Fst pair: pairs of strains compared in the Fst method (AB, BC and CA)
