# Supplementary material for: Whole genome re-sequencing reveals recent signatures of selection in three strains of farmed Nile tilapia (Oreochromis niloticus)
Source: Sci Rep. 2020 Jul 13;10:11514. doi: 10.1038/s41598-020-68064-5 (PMC7359307; doi:10.1038/s41598-020-68064-5)
Supplement: Supplementary file 9 — Supplementary table S7 [file 41598_2020_68064_MOESM9_ESM.pdf]

Supplementary information

Whole genome re-sequencing reveals recent signatures of selection in three strains of farmed Nile tilapia (*Oreochromis niloticus*)

María I. Cádiz[12], María E. López[31], Diego Díaz-Domínguez[4], Giovanna Cáceres[12], Grazyella M. Yoshida[1], Daniel Gomez-Uchida[5.6], José M. Yáñez[1,6]*.

[1] Facultad de Ciencias Veterinarias y Pecuarias, Universidad de Chile, Avenida Santa Rosa 11735, 8820808, La Pintana, Santiago, Chile

[2] Programa de Doctorado en Ciencias Silvoagropecuarias y Veterinarias, Campus Sur, Universidad de Chile, Santa Rosa 11315, La Pintana, Santiago, Chile. CP: 8820808.

[3] Department of Animal Breeding and Genetics, Swedish University of Agricultural Sciences, Uppsala, Sweden.

[4] Departamento de Ciencias de la Computación, Universidad de Chile.

[5] Facultad de Ciencias Naturales y Oceanográficas, Universidad de Concepción, Concepción, Chile.

[6] Núcleo Milenio INVASAL, Concepción, Chile

*jmayanez@uchile.cl +56-2 29785533 (Corresponding Author).

Supplementary Table S7 . List of terms and definitions of enrichment analysis to each strain (A, B and C)					
		A			
Code	***Term***	***Definition***	***#g***	***P-val***	***T***
		Biological process 57.0% (369 genes)			
GO:0021546	rhombomere development	The process whose specific outcome is the progression of the rhombomere over time, from its formation to the mature structure. Rhombomeres are transverse segments of the developing rhombencephalon. Rhombomeres are lineage restricted, express different genes from one another, and adopt different developmental fates.	5	0.001	E
GO:0048468	cell development	The process whose specific outcome is the progression of the cell over time, from its formation to the mature structure. Cell development does not include the steps involved in committing a cell to a specific fate.	46	0.0044	E
GO:0050890	cognition	The operation of the mind by which an organism becomes aware of objects of thought or perception; it includes the mental activities associated with thinking, learning, and memory.	4	0.0055	B
GO:0007399	nervous system development	The process whose specific outcome is the progression of nervous tissue over time, from its formation to its mature state.	52	0.0073	E
GO:0021593	rhombomere morphogenesis	The process in which the anatomical structure of the rhombomere is generated and organized. Rhombomeres are transverse segments of the developing rhombencephalon. Rhombomeres are lineage restricted, express different genes from one another, and adopt different developmental fates.	4	0.0096	E
GO:0048469	cell maturation	A developmental process, independent of morphogenetic (shape) change, that is required for a cell to attain its fully functional state.	5	0.015	E
GO:0051225	spindle assembly	The aggregation, arrangement and bonding together of a set of components to form the spindle, the array of microtubules and associated molecules that serves to move duplicated chromosomes apart.	5	0.016	
GO:0008038	neuron recognition	The process in which a neuronal cell in a multicellular organism interprets its surroundings.	4	0.022	B
GO:0010628	positive regulation of gene expression	Any process that increases the frequency, rate or extent of gene expression. Gene expression is the process in which a gene's coding sequence is converted into a mature gene product or products (proteins or RNA). This includes the production of an RNA transcript as well as any processing to produce a mature RNA product or an mRNA or circRNA (for protein-coding genes) and the translation of that mRNA or circRNA into protein. Protein maturation is included when required to form an active form of a product from an inactive precursor form.	16	0.023	
GO:0030154	cell differentiation	The process in which relatively unspecialized cells, e.g. embryonic or regenerative cells, acquire specialized structural and/or functional features that characterize the cells, tissues, or organs of the mature organism or some other relatively stable phase of the organism's life history. Differentiation includes the processes involved in commitment of a cell to a specific fate and its subsequent development to the mature state.	61	0.023	E
GO:0007417	central nervous system development	The process whose specific outcome is the progression of the central nervous system over time, from its formation to the mature structure. The central nervous system is the core nervous system that serves an integrating and coordinating function. In vertebrates it consists of the brain and spinal cord. In those invertebrates with a central nervous system it typically consists of a brain, cerebral ganglia and a nerve cord.	25	0.024	E

GO:0021654	rhombomere boundary formation	The process that gives rise to a rhombomere boundary. This process pertains to the initial formation of a boundary delimiting a rhombomere. Rhombomeres are transverse segments of the developing rhombencephalon that are lineage restricted, express different genes from one another, and adopt different developmental fates. Rhombomeres are numbered in anterior to posterior order.	3	0.028	E
GO:0030510	regulation of BMP signaling pathway	Any process that modulates the frequency, rate or extent of the activity of any BMP receptor signaling pathway.	6	0.029	
GO:0001501	skeletal system development	The process whose specific outcome is the progression of the skeleton over time, from its formation to the mature structure. The skeleton is the bony framework of the body in vertebrates (endoskeleton) or the hard outer envelope of insects (exoskeleton or dermoskeleton).	14	0.03	G
GO:0021594	rhombomere formation	The process that gives rise to the rhombomere. This process pertains to the initial formation of a structure from unspecified parts. Rhombomeres are transverse segments of the developing rhombencephalon. Rhombomeres are lineage restricted, express different genes from one another, and adopt different developmental fates.	3	0.039	E
GO:0051216	cartilage development	The process whose specific outcome is the progression of a cartilage element over time, from its formation to the mature structure. Cartilage elements are skeletal elements that consist of connective tissue dominated by extracellular matrix containing collagen type II and large amounts of proteoglycan, particularly chondroitin sulfate.	8	0.043	G
GO:0048666	neuron development	The process whose specific outcome is the progression of a neuron over time, from initial commitment of the cell to a specific fate, to the fully functional differentiated cell	24	0.043	E
GO:0048859	formation of anatomical boundary	The process in which the limits of an anatomical structure are generated. An anatomical structure is any biological entity that occupies space and is distinguished from its surroundings. Anatomical structures can be macroscopic such as a carpel, or microscopic such as an acrosome.	3	0.045	E
GO:0007612	learning	Any process in an organism in which a relatively long-lasting adaptive behavioral change occurs as the result of experience.	3	0.045	B
GO:0051254	positive regulation of RNA metabolic process	Any process that activates or increases the frequency, rate or extent of the chemical reactions and pathways involving RNA.	14	0.046	
GO:0061448	connective tissue development	The progression of a connective tissue over time, from its formation to the mature structure.	8	0.051	E
GO:0060113	inner ear receptor cell differentiation	The process in which relatively unspecialized cells, acquire specialized structural and/or functional features of inner ear receptor cells. Inner ear receptor cells are mechanorecptors found in the inner ear responsible for transducing signals involved in balance and sensory perception of sound.	4	0.052	E
GO:0048731	system development	The process whose specific outcome is the progression of an organismal system over time, from its formation to the mature structure. A system is a regularly interacting or interdependent group of organs or tissues that work together to carry out a given biological process.	90	0.053	E
GO:0048514	blood vessel morphogenesis	The process in which the anatomical structures of blood vessels are generated and organized. The blood vessel is the vasculature carrying blood.	15	0.06	E
GO:0008366	axon ensheathment	Any process in which the axon of a neuron is insulated, and that insulation maintained, thereby preventing dispersion of the electrical signal.	4	0.061	

GO:0010557	positive regulation of macromolecule biosynthetic process	Any process that increases the rate, frequency or extent of the chemical reactions and pathways resulting in the formation of a macromolecule, any molecule of high relative molecular mass, the structure of which essentially comprises the multiple repetition of units derived, actually or conceptually, from molecules of low relative molecular mass.	14	0.065	
GO:0051173	positive regulation of nitrogen compound metabolic process	Any process that activates or increases the frequency, rate or extent of the chemical reactions and pathways involving nitrogen or nitrogenous compounds.	15	0.066	
GO:0002040	sprouting angiogenesis	The extension of new blood vessels from existing vessels into avascular tissues, this process includes the specialization of endothelial cells into leading tip and stalk cells, proliferation and migration of the endothelial cells and cell adhesion resulting in angiogenic sprout fusion or lumen formation	6	0.067	E
GO:0048285	organelle fission	The creation of two or more organelles by division of one organelle.	12	0.068	
GO:0007422	peripheral nervous system development	The process whose specific outcome is the progression of the peripheral nervous system over time, from its formation to the mature structure. The peripheral nervous system is one of the two major divisions of the nervous system. Nerves in the PNS connect the central nervous system (CNS) with sensory organs, other organs, muscles, blood vessels and glands.	5	0.068	E
GO:0051321	meiotic cell cycle	Progression through the phases of the meiotic cell cycle, in which canonically a cell replicates to produce four offspring with half the chromosomal content of the progenitor cell via two nuclear divisions.	6	0.076	
GO:0030902	hindbrain development	The process whose specific outcome is the progression of the hindbrain over time, from its formation to the mature structure. The hindbrain is the posterior of the three primary divisions of the developing chordate brain, or the corresponding part of the adult brain (in vertebrates, includes the cerebellum, pons, and medulla oblongata and controls the autonomic functions and equilibrium).	7	0.081	E
GO:0048599	oocyte development	The process whose specific outcome is the progression of an oocyte over time, from initial commitment of the cell to its specific fate, to the fully functional differentiated cell.	3	0.082	R
GO:0022402	cell cycle process	The cellular process that ensures successive accurate and complete genome replication and chromosome segregation.	16	0.083	
GO:0048477	oogenesis	The complete process of formation and maturation of an ovum or female gamete from a primordial female germ cell. Examples of this process are found in Mus musculus and Drosophila melanogaster.	4	0.085	R
GO:0098609	single organismal cell-cell adhesion	The attachment of one cell to another cell via adhesion molecules.	8	0.085	
GO:0045935	positive regulation of nucleobase-containing compound metabolic process	Any cellular process that activates or increases the frequency, rate or extent of the chemical reactions and pathways involving nucleobases, nucleosides, nucleotides and nucleic acids.	14	0.085	
GO:0009887	organ morphogenesis	Morphogenesis of an animal organ. An organ is defined as a tissue or set of tissues that work together to perform a specific function or functions. Morphogenesis is the process in which anatomical structures are generated and organized. Organs are commonly observed as visibly distinct structures, but may also exist as loosely associated clusters of cells that work together to perform a specific function or functions.	24	0.089	E
GO:0070925	organelle assembly	The aggregation, arrangement and bonding together of a set of components to form an organelle. An organelle is an organized structure of distinctive morphology and function. Includes the nucleus, mitochondria, plastids, vacuoles, vesicles, ribosomes and the cytoskeleton. Excludes the plasma membrane.	16	0.089	

GO:0009994	oocyte differentiation	The process in which a relatively unspecialized immature germ cell acquires the specialized features of a mature female gamete.	3	0.09	R
Celular Component 38.0% (246 genes)					
GO:0005759	mitochondrial matrix	The gel-like material, with considerable fine structure, that lies in the matrix space, or lumen, of a mitochondrion. It contains the enzymes of the tricarboxylic acid cycle and, in some organisms, the enzymes concerned with fatty acid oxidation.	8	0.026	
GO:0030018	Z disc	Platelike region of a muscle sarcomere to which the plus ends of actin filaments are attached.	4	0.051	G
GO:0030016	myofibril	The contractile element of skeletal and cardiac muscle; a long, highly organized bundle of actin, myosin, and other proteins that contracts by a sliding filament mechanism.	6	0.059	G
GO:0043292	contractile fiber	Fibers, composed of actin, myosin, and associated proteins, found in cells of smooth or striated muscle.	6	0.064	G
GO:0031674	I band	A region of a sarcomere that appears as a light band on each side of the Z disc, comprising a region of the sarcomere where thin (actin) filaments are not overlapped by thick (myosin) filaments; contains actin, troponin, and tropomyosin; each sarcomere includes half of an I band at each end.	4	0.068	G
Molecular function 58.9% (381 genes)					
GO:0046983	protein dimerization activity	The formation of a protein dimer, a macromolecular structure consists of two noncovalently associated identical or nonidentical subunits.	18	0.026	
GO:0008092	cytoskeletal protein binding	Interacting selectively and non-covalently with any protein component of any cytoskeleton (actin, microtubule, or intermediate filament cytoskeleton).	20	0.071	G
KEGG 23.6% (153 genes)					
dre04020	Calcium signaling pathway	Ca2+ that enters the cell from the outside is a principal source of signal Ca2+. Entry of Ca2+ is driven by the presence of a large electrochemical gradient across the plasma membrane. Cells use this external source of signal Ca2+ by activating various entry channels with widely different properties. The voltage-operated channels (VOCs) are found in excitable cells and generate the rapid Ca2+ fluxes that control fast cellular processes. There are many other Ca2+-entry channels, such as the receptor-operated channels (ROCs), for example the NMDA (N-methyl-D-aspartate) receptors (NMDARs) that respond to glutamate. There also are second-messenger-operated channels (SMOCs) and store-operated channels (SOCs).	13	0.061	G
dre00052	Galactose metabolism	Metabolism; Carbohydrate metabolism	4	0.064	
dre03013	RNA transport	RNA transport from the nucleus to the cytoplasm is fundamental for gene expression. The different RNA species that are produced in the nucleus are exported through the nuclear pore complexes (NPCs) via mobile export receptors.	9	0.074	
dre04512	ECM-receptor interaction	The extracellular matrix (ECM) consists of a complex mixture of structural and functional macromolecules and serves an important role in tissue and organ morphogenesis and in the maintenance of cell and tissue structure and function. Specific interactions between cells and the ECM are mediated by transmembrane molecules, mainly integrins and perhaps also proteoglycans, CD36, or other cell-surface-associated components. These interactions lead to a direct or indirect control of cellular activities such as adhesion, migration, differentiation, proliferation, and apoptosis. In addition, integrins function as mechanoreceptors and provide a force-transmitting physical link between the ECM and the cytoskeleton.	6	0.077	G
B					

Biological process (BP) 44.8% 315 (genes)					
GO:0001666	response to hypoxia	Any process that results in a change in state or activity of a cell or an organism (in terms of movement, secretion, enzyme production, gene expression, etc.) as a result of a stimulus indicating lowered oxygen tension. Hypoxia, defined as a decline in O2 levels below normoxic levels of 20.8 - 20.95%, results in metabolic adaptation at both the cellular and organismal level.	5	0.042	A
GO:0060847	endothelial cell fate specification	The process involved in the specification of identity of an endothelial cell. Once specification has taken place, a cell will be committed to differentiate down a specific pathway if left in its normal environment.	2	0.046	
GO:0006535	cysteine biosynthetic process from serine	The chemical reactions and pathways resulting in the formation of cysteine from other compounds, including serine.	2	0.046	
GO:0030168	platelet activation	A series of progressive, overlapping events triggered by exposure of the platelets to subendothelial tissue. These events include shape change, adhesiveness, aggregation, and release reactions. When carried through to completion, these events lead to the formation of a stable hemostatic plug.	3	0.059	
GO:0006887	exocytosis	A process of secretion by a cell that results in the release of intracellular molecules (e.g. hormones, matrix proteins) contained within a membrane-bounded vesicle. Exocytosis can occur either by full fusion, when the vesicle collapses into the plasma membrane, or by a kiss-and-run mechanism that involves the formation of a transient contact, a pore, between a granule (for exemple of chromaffin cells) and the plasma membrane. The latter process most of the time leads to only partial secretion of the granule content. Exocytosis begins with steps that prepare vesicles for fusion with the membrane (tethering and docking) and ends when molecules are secreted from the cell.	8	0.065	
GO:0008361	regulation of cell size	Any process that modulates the size of a cell.	6	0.068	G
GO:0006259	DNA metabolic process	Any cellular metabolic process involving deoxyribonucleic acid. This is one of the two main types of nucleic acid, consisting of a long, unbranched macromolecule formed from one, or more commonly, two, strands of linked deoxyribonucleotides.	16	0.068	G
GO:0030258	lipid modification	The covalent alteration of one or more fatty acids in a lipid, resulting in a change in the properties of the lipid.	7	0.072	
GO:0003158	endothelium development	The process whose specific outcome is the progression of an endothelium over time, from its formation to the mature structure. Endothelium refers to the layer of cells lining blood vessels, lymphatics, the heart, and serous cavities, and is derived from bone marrow or mesoderm. Corneal endothelium is a special case, derived from neural crest cells.	4	0.084	E
GO:0072080	nephron tubule development	The progression of a nephron tubule over time, from its initial formation to the mature structure. A nephron tubule is an epithelial tube that is part of the nephron, the functional part of the kidney.	3	0.086	E
GO:0098656	anion transmembrane transport	The process in which an anion is transported across a membrane.	5	0.089	
GO:0005984	disaccharide metabolic process	The chemical reactions and pathways involving any disaccharide, sugars composed of two monosaccharide units.	2	0.091	
GO:0044003	modification by symbiont of host morphology or physiology	The process in which a symbiont organism effects a change in the structure or processes of its host organism.	2	0.091	

GO:0006281	DNA repair	The process of restoring DNA after damage. Genomes are subject to damage by chemical and physical agents in the environment (e.g. UV and ionizing radiations, chemical mutagens, fungal and bacterial toxins, etc.) and by free radicals or alkylating agents endogenously generated in metabolism. DNA is also damaged because of errors during its replication. A variety of different DNA repair pathways have been reported that include direct reversal, base excision repair, nucleotide excision repair, photoreactivation, bypass, double-strand break repair pathway, and mismatch repair pathway	11	0.092	
GO:0061326	renal tubule development	The progression of the renal tubule over time from its formation to the mature form. A renal tubule is a tube that filters, re-absorbs and secretes substances to rid an organism of waste and to play a role in fluid homeostasis.	3	0.097	E
Celular Component 32.9% (231 genes)					
GO:1990904	intracellular ribonucleoprotein complex	A macromolecular complex containing both protein and RNA molecules.	19	0.073	
Molecular function 43.0% (302 genes)					
GO:0004175	endopeptidase activity	Catalysis of the hydrolysis of internal, alpha-peptide bonds in a polypeptide chain.	22	0.014	
GO:0008236	serine-type peptidase activity	Catalysis of the hydrolysis of peptide bonds in a polypeptide chain by a catalytic mechanism that involves a catalytic triad consisting of a serine nucleophile that is activated by a proton relay involving an acidic residue (e.g. aspartate or glutamate) and a basic residue (usually histidine).	13	0.016	
GO:0004252	serine-type endopeptidase activity	Catalysis of the hydrolysis of internal, alpha-peptide bonds in a polypeptide chain by a catalytic mechanism that involves a catalytic triad consisting of a serine nucleophile that is activated by a proton relay involving an acidic residue (e.g. aspartate or glutamate) and a basic residue (usually histidine).	12	0.017	
GO:0046914	transition metal ion binding	Interacting selectively and non-covalently with a transition metal ions; a transition metal is an element whose atom has an incomplete d-subshell of extranuclear electrons, or which gives rise to a cation or cations with an incomplete d-subshell. Transition metals often have more than one valency state. Biologically relevant transition metals include vanadium, manganese, iron, copper, cobalt, nickel, molybdenum and silver.	55	0.025	
GO:0015297	ion antiporter activity	Enables the active transport of a solute across a membrane by a mechanism whereby two or more species are transported in opposite directions in a tightly coupled process not directly linked to a form of energy other than chemiosmotic energy. The reaction is: solute A(out) + solute B(in) = solute A(in) + solute B(out)	5	0.029	
GO:0015297	antiporter activity	Enables the active transport of a solute across a membrane by a mechanism whereby two or more species are transported in opposite directions in a tightly coupled process not directly linked to a form of energy other than chemiosmotic energy. The reaction is: solute A(out) + solute B(in) = solute A(in) + solute B(out)	6	0.042	
GO:0004122	cystathionine beta-synthase activity	Catalysis of the reaction: L-serine + L-homocysteine = cystathionine + H2O.	2	0.054	
GO:0016160	amylase activity	Catalysis of the hydrolysis of amylose or an amylose derivative.	3	0.077	
KEGG 19.1% (134 genes)					
dre03450	Non-homologous end-joining	Nonhomologous end joining (NHEJ) eliminates DNA double-strand breaks (DSBs) by direct ligation. NHEJ involves binding of the KU heterodimer to double-stranded DNA ends, recruitment of DNA-PKcs (MRX complex in yeast), processing of ends, and recruitment of the DNA ligase IV (LIG4)-XRCC4 complex, which brings about ligation.	3	0.045	

dre04514	Cell adhesion molecules (CAMs)	Cell adhesion molecules are (glyco)proteins expressed on the cell surface and play a critical role in a wide array of biologic processes that include hemostasis, the immune response, inflammation, embryogenesis, and development of neuronal tissue. There are four main groups: the integrin family, the immunoglobulin superfamily, selectins, and cadherins. Membrane proteins that mediate immune cell–cell interactions fall into different categories, namely those involved in antigen recognition, costimulation and cellular adhesion. Furthermore cell-cell adhesions are important for brain morphology and highly coordinated brain functions such as memory and learning. During early development of the nervous system, neurons elongate their axons towards their targets and establish and maintain synapses through formation of cell-cell adhesions. Cell-cell adhesions also underpin axon-axon contacts and link neurons with supporting schwann cells and oligodendrocytes.	8	0.05	E
dre04810	Regulation of actin cytoskeleton	Regulation of actin cytoskeleton	12	0.069	G
dre04510	Focal adhesion	Cell-matrix adhesions play essential roles in important biological processes including cell motility, cell proliferation, cell differentiation, regulation of gene expression and cell survival. At the cell-extracellular matrix contact points, specialized structures are formed and termed focal adhesions, where bundles of actin filaments are anchored to transmembrane receptors o the integrin family through a multi-molecular complex of junctional plaque proteins. Some of the constituents of focal adhesions participate in the structural link between membrane receptors and the actin cytoskeleton, while others are signalling molecules, including different protein kinases and phosphatases, their substrates, and various adapter proteins. Integrin signaling is dependent upon the non-receptor tyrosine kinase activities of the FAK and src proteins as well as the adaptor protein functions of FAK, src and Shc to initiate downstream signaling events. These signalling events culminate in reorganization of the actin cytoskeleton; a prerequisite for changes in cell shape and motility, and gene expression. Similar morphological alterations and modulation of gene expression are initiated by the binding of growth factors to their respective receptors, emphasizing the considerable crosstalk between adhesion- and growth factor-mediated signalling.	11	0.084	E
C					
Biological process 42.8% (221 genes)					
GO:0007156	homophilic cell adhesion via plasma membrane adhesion molecules	The attachment of a plasma membrane adhesion molecule in one cell to an identical molecule in an adjacent cell.	11	0.0005 6	
GO:0009311	oligosaccharide metabolic process	The chemical reactions and pathways involving oligosaccharides, molecules with between two and (about) 20 monosaccharide residues connected by glycosidic linkages.	6	0.0016	
GO:0030149	sphingolipid catabolic process	The chemical reactions and pathways resulting in the breakdown of sphingolipids, any of a class of lipids containing the long-chain amine diol sphingosine or a closely related base (a sphingoid).	4	0.0021	
GO:0046466	membrane lipid catabolic process	The chemical reactions and pathways resulting in the breakdown of membrane lipids, any lipid found in or associated with a biological membrane.	4	0.0021	

GO:0007422	peripheral nervous system development	The process whose specific outcome is the progression of the peripheral nervous system over time, from its formation to the mature structure. The peripheral nervous system is one of the two major divisions of the nervous system. Nerves in the PNS connect the central nervous system (CNS) with sensory organs, other organs, muscles, blood vessels and glands.	6	0.0026	E
GO:0002934	desmosome organization	A process that is carried out at the cellular level which results in the assembly, arrangement of constituent parts, or disassembly of a desmosome. A desmosome is a patch-like intercellular junction found in vertebrate tissues, consisting of parallel zones of two cell membranes, separated by an space of 25-35 nm, and having dense fibrillar plaques in the subjacent cytoplasm.	3	0.0026	E
GO:0009313	oligosaccharide catabolic process	The chemical reactions and pathways resulting in the breakdown of oligosaccharides, molecules with between two and (about) 20 monosaccharide residues connected by glycosidic linkages.	3	0.0054	
GO:0001666	response to hypoxia	Any process that results in a change in state or activity of a cell or an organism (in terms of movement, secretion, enzyme production, gene expression, etc.) as a result of a stimulus indicating lowered oxygen tension. Hypoxia, defined as a decline in O2 levels below normoxic levels of 20.8 - 20.95%, results in metabolic adaptation at both the cellular and organismal level.	5	0.013	A
GO:0019377	glycolipid catabolic process	The chemical reactions and pathways resulting in the breakdown of glycolipid, a class of 1,2-di-O-acylglycerols joined at oxygen 3 by a glycosidic linkage to a carbohydrate part (usually a mono-, di- or tri-saccharide).	3	0.013	
GO:0035270	endocrine system development	Progression of the endocrine system over time, from its formation to a mature structure. The endocrine system is a system of hormones and ductless glands, where the glands release hormones directly into the blood, lymph or other intercellular fluid, and the hormones circulate within the body to affect distant organs. The major glands that make up the human endocrine system are the hypothalamus, pituitary, thyroid, parathryoids, adrenals, pineal body, and the reproductive glands which include the ovaries and testes.	6	0.014	E
GO:0007411	axon guidance	The chemotaxis process that directs the migration of an axon growth cone to a specific target site in response to a combination of attractive and repulsive cues.	9	0.015	B
GO:0097485	neuron projection guidance	The process in which the migration of a neuron projection is directed to a specific target site in response to a combination of attractive and repulsive cues.	9	0.016	B
GO:0006606	protein import into nucleus	The directed movement of a protein from the cytoplasm to the nucleus.	5	0.016	
GO:0019318	hexose metabolic process	The chemical reactions and pathways involving a hexose, any monosaccharide with a chain of six carbon atoms in the molecule.	5	0.016	
GO:0051170	single-organism nuclear import	The directed movement of substances into the nucleus.	5	0.016	
GO:0034504	protein targeting to nucleus	A process in which a protein transports or maintains the localization of another protein to the nucleus.	5	0.016	
GO:0017038	protein import	The targeting and directed movement of proteins into a cell or organelle. Not all import involves an initial targeting event.	6	0.017	
GO:0005996	monosaccharide metabolic process	The chemical reactions and pathways involving monosaccharides, the simplest carbohydrates. They are polyhydric alcohols containing either an aldehyde or a keto group and between three to ten or more carbon atoms. They form the constitutional repeating units of oligo- and polysaccharides.	5	0.022	
GO:0010001	glial cell differentiation	The process in which a relatively unspecialized cell acquires the specialized features of a glial cell.	5	0.026	E

GO:0072594	establishment of protein localization to organelle	The directed movement of a protein to a specific location on or in an organelle. Encompasses establishment of localization in the membrane or lumen of a membrane-bounded organelle.	7	0.026	
GO:0042478	regulation of eye photoreceptor cell development	Any process that modulates the frequency, rate or extent of eye photoreceptor development.	2	0.033	E
GO:0006535	cysteine biosynthetic process from serine	The chemical reactions and pathways resulting in the formation of cysteine from other compounds, including serine.	2	0.033	
GO:0061564	axon development	The progression of an axon over time. Covers axonogenesis (de novo generation of an axon) and axon regeneration (regrowth), as well as processes pertaining to the progression of the axon over time (fasciculation and defasciculation).	12	0.035	E
GO:0060027	convergent extension involved in gastrulation	The morphogenetic process in which an epithelium narrows along one axis and lengthens in a perpendicular axis usually resulting in the formation of the three primary germ layers, ectoderm, mesoderm and endoderm	5	0.041	E
GO:0007043	cell-cell junction assembly	The aggregation, arrangement and bonding together of a set of components to form a junction between cells.	3	0.046	
GO:0043010	camera-type eye development	The process whose specific outcome is the progression of the camera-type eye over time, from its formation to the mature structure. The camera-type eye is an organ of sight that receives light through an aperture and focuses it through a lens, projecting it on a photoreceptor field.	10	0.046	E
GO:0061162	establishment of monopolar cell polarity	The specification and formation of monopolar intracellular organization or cell growth patterns. Monopolar cell organization is directional organization along an axis.	2	0.049	
GO:0044242	cellular lipid catabolic process	The chemical reactions and pathways resulting in the breakdown of lipids, as carried out by individual cells.	5	0.053	
GO:0006605	protein targeting	The process of targeting specific proteins to particular regions of the cell, typically membrane-bounded subcellular organelles. Usually requires an organelle specific protein sequence motif.	7	0.058	
GO:0048667	cell morphogenesis involved in neuron differentiation	The process in which the structures of a neuron are generated and organized. This process occurs while the initially relatively unspecialized cell is acquiring the specialized features of a neuron.	11	0.058	E
GO:0090162	establishment of epithelial cell polarity	The specification and formation of anisotropic intracellular organization of an epithelial cell.	2	0.064	
GO:2000178	negative regulation of neural precursor cell proliferation	Any process that stops, prevents, or reduces the frequency, rate or extent of neural precursor cell proliferation.	2	0.064	E
GO:0070646	protein modification by small protein removal	A protein modification process in which one or more covalently attached groups of a small protein, such as ubiquitin or a ubiquitin-like protein, are removed from a target protein.	5	0.067	
GO:0009069	serine family amino acid metabolic process	The chemical reactions and pathways involving amino acids of the serine family, comprising cysteine, glycine, homoserine, selenocysteine and serine.	3	0.068	
GO:0033365	protein localization to organelle	A process in which a protein is transported to, or maintained in, a location within an organelle.	7	0.071	
GO:0060059	embryonic retina morphogenesis in camera-type eye	The process in which the anatomical structure of the retina is generated and organized in a camera-type eye during the embryonic life stage.	3	0.072	E

GO:0000904	cell morphogenesis involved in differentiation	The change in form (cell shape and size) that occurs when relatively unspecialized cells, e.g. embryonic or regenerative cells, acquire specialized structural and/or functional features that characterize the cells, tissues, or organs of the mature organism or some other relatively stable phase of the organism's life history.	12	0.075	E
GO:0031175	neuron projection development	The process whose specific outcome is the progression of a neuron projection over time, from its formation to the mature structure. A neuron projection is any process extending from a neural cell, such as axons or dendrites (collectively called neurites).	13	0.075	E
GO:0016052	single-organism carbohydrate catabolic process	The chemical reactions and pathways resulting in the breakdown of carbohydrates, any of a group of organic compounds based of the general formula Cx(H2O)y.	4	0.076	
GO:0048812	neuron projection morphogenesis	The process in which the anatomical structures of a neuron projection are generated and organized. A neuron projection is any process extending from a neural cell, such as axons or dendrites.	11	0.08	E
GO:0060026	convergent extension	The morphogenetic process in which an epithelium narrows along one axis and lengthens in a perpendicular axis.	6	0.085	
GO:0002009	morphogenesis of an epithelium	The process in which the anatomical structures of epithelia are generated and organized. An epithelium consists of closely packed cells arranged in one or more layers, that covers the outer surfaces of the body or lines any internal cavity or tube.	12	0.086	E
GO:0048592	eye morphogenesis	The process in which the anatomical structures of the eye are generated and organized.	7	0.089	E
GO:0036211	protein modification process	The covalent alteration of one or more amino acids occurring in proteins, peptides and nascent polypeptides (co-translational, post-translational modifications). Includes the modification of charged tRNAs that are destined to occur in a protein (pre-translation modification).	45	0.094	
GO:0006464	cellular protein modification process	The covalent alteration of one or more amino acids occurring in proteins, peptides and nascent polypeptides (co-translational, post-translational modifications) occurring at the level of an individual cell. Includes the modification of charged tRNAs that are destined to occur in a protein (pre-translation modification).	45	0.094	
GO:0006508	proteolysis	The hydrolysis of proteins into smaller polypeptides and/or amino acids by cleavage of their peptide bonds.	22	0.098	
Celular Component 33.3% (172 genes)					
GO:0005681	spliceosomal complex	Any of a series of ribonucleoprotein complexes that contain snRNA(s) and small nuclear ribonucleoproteins (snRNPs), and are formed sequentially during the spliceosomal splicing of one or more substrate RNAs, and which also contain the RNA substrate(s) from the initial target RNAs of splicing, the splicing intermediate RNA(s), to the final RNA products.	6	0.031	
GO:0001750	photoreceptor outer segment	The outer segment of a vertebrate photoreceptor that contains a stack of membrane discs embedded with photoreceptor proteins	3	0.073	
Molecular function 54.3% (280 genes)					
GO:0050839	cell adhesion molecule binding	Interacting selectively and non-covalently with a cell adhesion molecule.	6	0.012	
GO:0046983	protein dimerization activity	The formation of a protein dimer, a macromolecular structure consists of two noncovalently associated identical or nonidentical subunits.	15	0.018	
GO:0005516	calmodulin binding	Interacting selectively and non-covalently with calmodulin, a calcium-binding protein with many roles, both in the calcium-bound and calcium-free states	5	0.023	

GO:0001882	nucleoside binding	Interacting selectively and non-covalently with a nucleoside, a compound consisting of a purine or pyrimidine nitrogenous base linked either to ribose or deoxyribose.	53	0.046	
GO:0016798	hydrolase activity, acting on glycosyl bonds	Catalysis of the hydrolysis of any glycosyl bond.	8	0.056	
GO:0032553	ribonucleotide binding	Interacting selectively and non-covalently with a ribonucleotide, any compound consisting of a ribonucleoside that is esterified with (ortho)phosphate or an oligophosphate at any hydroxyl group on the ribose moiety.	53	0.059	
GO:0000166	nucleotide binding	Interacting selectively and non-covalently with a nucleotide, any compound consisting of a nucleoside that is esterified with (ortho)phosphate or an oligophosphate at any hydroxyl group on the ribose or deoxyribose.	62	0.064	
GO:1901265	nucleoside phosphate binding	Interacting selectively and non-covalently with nucleoside phosphate.	62	0.064	
GO:0046906	tetrapyrrole binding	Interacting selectively and non-covalently with a tetrapyrrole, a compound containing four pyrrole nuclei variously substituted and linked to each other through carbons at the alpha position.	7	0.081	
GO:0042802	identical protein binding	Interacting selectively and non-covalently with an identical protein or proteins.	7	0.087	
KEGG 18.0% (93 genes)					
dre04115	p53 signaling pathway	p53 activation is induced by a number of stress signals, including DNA damage, oxidative stress and activated oncogenes. The p53 protein is employed as a transcriptional activator of p53-regulated genes. This results in three major outputs; cell cycle arrest, cellular senescence or apoptosis. Other p53-regulated gene functions communicate with adjacent cells, repair the damaged DNA or set up positive and negative feedback loops that enhance or attenuate the functions of the p53 protein and integrate these stress responses with other signal transduction pathways.	5	0.034	
dre00511	Other glycan degradation	Metabolism; Glycan biosynthesis and metabolism	3	0.062	
dre04110	Cell cycle	Mitotic cell cycle progression is accomplished through a reproducible sequence of events, DNA replication (S phase) and mitosis (M phase) separated temporally by gaps known as G1 and G2 phases. Cyclin-dependent kinases (CDKs) are key regulatory enzymes, each consisting of a catalytic CDK subunit and an activating cyclin subunit. CDKs regulate the cell's progression through the phases of the cell cycle by modulating the activity of key substrates. Downstream targets of CDKs include transcription factor E2F and its regulator Rb. Precise activation and inactivation of CDKs at specific points in the cell cycle are required for orderly cell division. Cyclin-CDK inhibitors (CKIs), such as p16Ink4a, p15Ink4b, p27Kip1, and p21Cip1, are involved in the negative regulation of CDK activities, thus providing a pathway through which the cell cycle is negatively regulated.<br>Eukaryotic cells respond to DNA damage by activating signaling pathways that promote cell cycle arrest and DNA repair. In response to DNA damage, the checkpoint kinase ATM phosphorylates and activates Chk2, which in turn directly phosphorylates and activates p53 tumor suppressor protein. p53 and its transcriptional targets play an important role in both G1 and G2 checkpoints. ATR-Chk1-mediated protein degradation of Cdc25A protein phosphatase is also a mechanism conferring intra-S-phase checkpoint activation.	6	0.085	

A, B and C: strains
Code: GO and KEGG code
Term: name of enrichment term
Definition: definition of the term

#g: number of genes
P-val: P value
T: Terms associated to traits such as: Growth (G), early development (E), behavior (B), adaptation to enviroment (A)
